# Effect of Broncho-Vaxom (OM-85) on the frequency of chronic obstructive pulmonary disease (COPD) exacerbations

**DOI:** 10.1186/s12890-023-02665-4

**Published:** 2023-10-07

**Authors:** Joon Young Choi, Yong Bum Park, Tai Joon An, Kwang Ha Yoo, Chin Kook Rhee

**Affiliations:** 1 Division of Pulmonary and Critical Care Medicine, Department of Internal Medicine, Incheon St. Mary’s Hospital, College of Medicine, The Catholic University of Korea, Seoul, Republic of Korea; 2 Division of Pulmonary, Allergy, and Critical Care Medicine, Department of Internal Medicine, Hallym University Kangdong Sacred Heart Hospital, Seoul, Korea; 3 Division of Pulmonology and Critical Care, Department of Internal Medicine, Yeouido St. Mary’s Hospital, College of Medicine, The Catholic University of Korea, Seoul, Republic of Korea; 4 Division of Pulmonary, Allergy and Critical Care Medicine, Department of Internal Medicine, Konkuk University School of Medicine, 120 Neungdong-Ro, Gwangjin-Gu, Seoul, 05030 Republic of Korea; 5 Division of Pulmonary and Critical Care Medicine, Department of Internal Medicine, Seoul St. Mary’s Hospital, College of Medicine, The Catholic University of Korea, 222 Banpodaero, Seochogu, Seoul, 06591 Republic of Korea

**Keywords:** Broncho-Vaxom, Chronic obstructive pulmonary disease, HIRA database, Oral vaccine, OM-85, Exacerbation

## Abstract

**Background:**

Efforts have been made to reduce the risk of chronic obstructive pulmonary disease (COPD) exacerbations using a variety of measures. Broncho-Vaxom (BV) is an immunomodulating agent that has shown potential benefit by balancing between immune stimulation and regulation in patients with COPD. In this study, we evaluated the clinical efficacy of BV for reducing the risk of COPD exacerbations.

**Methods:**

This study was based on the Korean National Health Insurance database, which contains reimbursement information for almost the entire population of South Korea. We extracted data from 2016 to 2019 for patients started on BV during 2017–2018. We collected baseline data on demographics, comorbidities, inhaler use, hospital type, and insurance type 1 year before starting BV. We also analyzed exacerbation history, starting from the year before BV initiation.

**Results:**

In total, 238 patients were enrolled in this study. Their mean age was 69.2 ± 9.14 years, 79.8% were male, and 45% experienced at least one exacerbation. BV reduced the risk of moderate (odds ratio [OR] = 0.59, 95% confidence interval [CI]: 0.38–0.91) and moderate-to-severe exacerbations compared to pre- and post-BV (OR = 0.571, 95% CI: 0.37–0.89). BV use also reduced the incidence of moderate and moderate-to-severe exacerbations (incidence rate ratio [IRR] = 0.75, *p* = 0.03; and IRR = 0.77, *p* = 0.03, respectively). The use of BV was significantly delayed moderate exacerbations (hazard ratio = 0.68, *p* = 0.02), but not with moderate-to-severe or severe exacerbations.

**Conclusion:**

The use of BV was associated with fewer moderate and moderate-to-severe exacerbations. Additionally, BV was associated with a delay in moderate COPD exacerbations.

**Supplementary Information:**

The online version contains supplementary material available at 10.1186/s12890-023-02665-4.

## Introduction

Chronic obstructive pulmonary disease (COPD) is a major public health problem affecting > 300 million patients worldwide [1]. It is associated with high morbidity and mortality, and was recently ranked as the third leading cause of death worldwide [1, 2]. Among patients with COPD, those who experience frequent exacerbations have poor clinical outcomes, including reduced quality of life, accelerated decline in lung function, and increased mortality [3–6]. Additionally, healthcare costs are significantly higher in frequent exacerbators [3]. Efforts have been made to reduce exacerbation risk through pharmacological and non-pharmacological measures.

Various drugs, including bronchodilators, inhaled corticosteroids (ICS), phosphodiesterase E4 inhibitors, long-term macrolides, and mucoregulators, are effective for reducing the frequency of COPD exacerbations [7]. However, a large proportion of patients are categorized as frequent exacerbators, and are at high risk of morbidity and mortality [8, 9]. Various drugs have been investigated in terms of their efficacy for preventing exacerbations, including immunoregulators (so-called “oral vaccines” [10–13]).

Broncho-Vaxom (BV) is an immunoregulator that has been investigated for decades, mostly in patients with chronic inflammatory airway disease. BV is an oral bacterial lysate derived from eight major species of respiratory pathogens [14]. BV modulates the host immune system in various ways by balancing between immune stimulation and regulation [15–19]. It is known that immune stimulation involves both innate immunity (TLR-dependent synergism, polynuclear cell recruitment [CXCL1, 6, 8], proinflammatory response [IL-1, IL-6, TNF-α], secretion of antiviral cytokine [IL-12, IFN-α, IFN-γ], NK cell activation [CCL2, 3]) and adaptive immunity (B-cell activation [CCL2,3,20,22, BAFF, IL-6, APRIL]) [15]. Additionally, immune regulation is associated with maturation in dendritic cells (DC), specifically in pDC and moDC (CD80/CD86), and airway mucosal DC migration (CCR-7) [15]. Immune regulatory effect of BV is also known to involve the induction of regulatory T cells and local aggregation [15]. It was classified as a “grade A” treatment for chronic rhinosinusitis without nasal polyps in the European Position Paper on Rhinosinusitis and Nasal Polyps (EPOS) 2012 [20]. Previous studies have investigated the role of BV in COPD treatment, and showed potential benefits in terms of exacerbation risk, hospitalization duration, and antibiotic prescriptions [12, 13, 21, 22]. However, additional investigations using large databases with more comprehensive analysis are needed to reveal the beneficial effects of BV [7]. Moreover, there has been no real-world study that evaluate the effect of BV in real clinical practice.

In this study, we utilized national health insurance reimbursement data which collects medical data in almost all patients in South Korea, Our objective was to conduct a comprehensive analysis of the effects of BV on COPD exacerbations. We compared exacerbation risk, frequency, and incidence, and the time to first exacerbation, between the pre- and post-BV periods in COPD patients who used the drug. We also performed subgroup analyses to identify patients most likely to benefit from BV.

## Material and methods

### Study population and data collection

**Fig. 1:**
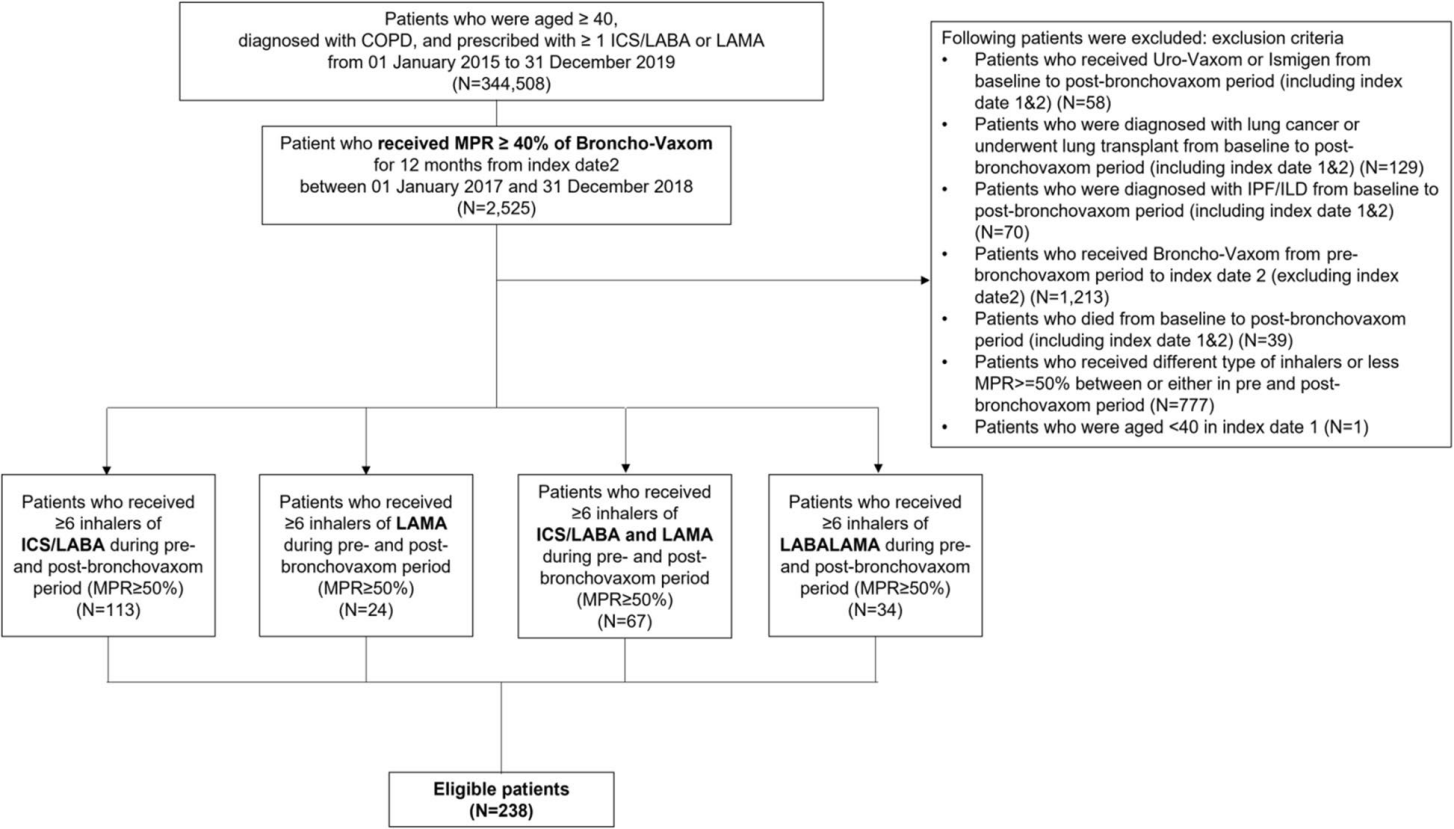
Flow chart of patient selection

The Health Insurance Review & Assessment Service (HIRA) reviews the adequacy of medical cost coverage and verifies insurance claims for almost the entire population of South Korea. The HIRA collects clinical and medical patient data through medical personnel for insurance reimbursement assessment. In this study, we extracted the data of COPD patients registered in the HIRA database from January 2016 to December 2019 (Fig. 1).

The COPD patients included in this study were: 1) aged ≥ 40 years at the index date; 2) prescribed one or more medication for COPD at least twice per year during the study period [long-acting muscarinic antagonist (LAMA), long-acting beta-2 agonist (LABA), inhaled corticosteroids (ICS) plus LABA (ICS + LABA), LABA plus LAMA (LABA + LAMA), short-acting muscarinic antagonist (SAMA), short-acting beta-2 agonist (SABA), SABA plus SAMA (SABA + SAMA), phosphodiesterase-4 inhibitor (PDE4-I), systemic bronchodilator, or theophylline];; 3) had one or more inpatient or outpatient claim with the International Classification of Disease-10 (ICD-10) code for COPD (J43–J44, except J43.0), i.e., any diagnosis for an inpatient claim, or a primary or 4^th^ secondary diagnosis for an outpatient claim, during the study period. We defined patients with COPD based on criteria 1), 2) and 3). To further select patients who are actively treated, we added a criterion of ICS/LABA or LAMA usage. The exclusion criteria were use of Uro-Vaxom® or Ismigen® during the study period; diagnosis of lung cancer, idiopathic pulmonary fibrosis, or interstitial lung disease during the study period; lung transplantation during the study period; prescription of BV during the pre-BV period or before the index date 2; death during the study period; and receiving a different type of inhaler or having a medication possession ratio < 50% for the same type of inhaler between or during the pre- and post-BV periods.

### Study design

**Fig. 2:**
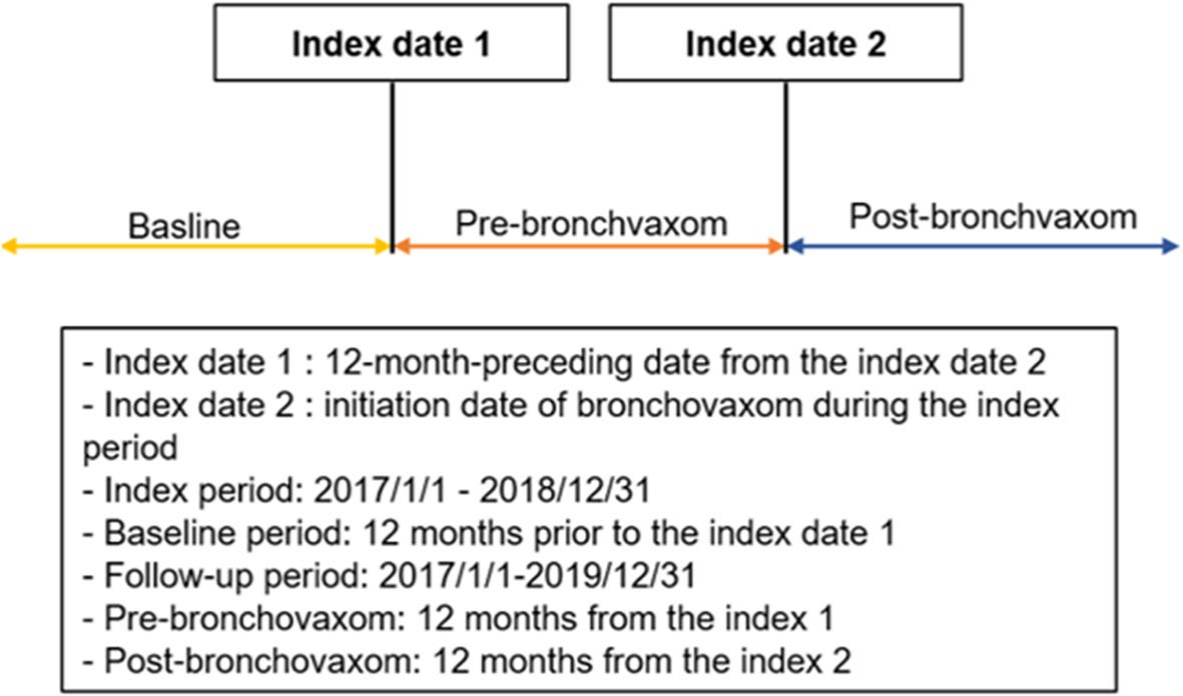
Overview of study design

We selected COPD patients started on BV during 2017–2018, which we defined as the index period (Fig. 2). The date of BV initiation during the index period was called index date 2, and the 12-month date preceding index date 2 was index date 1. We collected data on demographic characteristics during the 12-month period before index date 1, defined as the baseline. We compared outcomes between the 12 months before index date 2 (pre-BV period) and 12 months after index date 2 (post-BV period).

### Clinical parameters

Baseline characteristics of interest included age, sex, insurance type, hospital type, exacerbation history, history of asthma and pneumonia, modified Charlson Comorbidity Index (mCCI) score, and COPD medication use. Data on exacerbation history during the pre- and post-BV periods were also collected.

COPD exacerbations were identified based on the presence of the relevant diagnostic codes and systemic steroid and/or antibiotic prescriptions during the observation period (Supplemental Material 2.2.1.1). Moderate exacerbations were defined as events that required an outpatient visit and prescription of systemic corticosteroids and/or antibiotics. Severe exacerbations were defined as those that required an inpatient or emergency room visit for patients prescribed systemic corticosteroids and/or antibiotics. Exacerbations were also categorized according to antibiotic or oral corticosteroid (OCS) use.

### Statistical analysis

All statistical analyses were performed using SAS 9.4 software (SAS Institute, Cary, NC, USA) using SAS Enterprise Guide version 6.1. Continuous variables are expressed as means c ± standard deviations, and categorical values as numbers and percentages. Exacerbation risk was compared between the pre- and post-BV periods using a binomial mixed model. We also used the Poisson distribution to compare exacerbation frequency, relative frequency, and incidence rate ratio (IRR) between the pre- and post-BV periods. Data on the time to first exacerbation were subjected to survival analysis. All analyses were adjusted for potential confounding variables including age, sex, insurance type, hospital type, exacerbation history, history of asthma or pneumonia, mCCI score, COPD medication use, and index year.

## Results

### Baseline characteristics

Among the 344,508 COPD patients in the database, 2,525 were BV users during the study period (Fig. 1) and 238 were included in the analysis. The mean age of the BV users was 69.2 ± 9.14 years, and 79.8% were male (Table 1). BV was primarily prescribed in tertiary and general hospitals. About 45% of the patients prescribed BV had a history of exacerbations during the previous year, and 9.2% had ≥ 2 moderate or ≥ 1 severe exacerbation. About half of the patients were using ICS + LABA inhalers, and 28.2% were using triple therapy.

**Table 1:** Baseline characteristics of BV users

Variables	Numbers (*n* = 238)
Age	69.2 ± 9.14
Sex (male)	190 (79.8%)
Insurance type	
Health insurance	207 (87.0%)
Medical aid	31 (13.0%)
Hospital type	
Tertiary and general hospital	196 (82.4%)
Others	42 (17.6%)
History of COPD AE	
None	131 (55.0%)
1 moderate	85 (35.7%)
≥ 2 moderate or ≥ 1 severe	22 (9.2%)
History of pneumonia	63 (26.5%)
mCCI	2.62 ± 2.12
COPD medication	
LAMA	24 (10.1%)
LABA + LAMA	34 (14.3%)
ICS + LABA	113 (47.5%)
Triple	67 (28.2%)

Data are presented as n (%) or mean ± SD

*BV* Broncho-Vaxom, *COPD* Chronic obstructive pulmonary disease, *AE* Acute exacerbation, *mCCI* Modified Carlson Comorbidity Index, *LAMA* Long-acting muscarinic antagonist, *LABA* Long-acting beta2-agonist, *ICS* Inhaled corticosteroids

### Differences in exacerbation risk between the pre- and post-BV periods

A comparison of the risk of exacerbation between the pre- and post-BV periods is shown in Table 2. The risk of moderate and moderate-to-severe exacerbations decreased significantly after using BV (odds ratio [OR] = 0.59, 95% confidence interval [CI]: 0.38–0.91]; OR = 0.57, 95% CI: 0.37–0.89, respectively). However, the reduction in the risk of severe exacerbations did not reach statistical significance. BV did not significantly modify the risk of any of the three types of exacerbations (antibiotic-, OCS-, and antibiotic/OCS-related exacerbations), except moderate OCS-related exacerbations (OR = 0.58, 95% CI: 0.34–0.99) (Supplementary Material 6.1).

**Table 2:** Differences of exacerbation risk between pre- and post-BV period

	**Number of patient with events (%) (*n* = 238)**		**Crude OR (95% CI)**	**Adjusted OR (95% CI)**
	**pre-BV**	**post-BV**		
**COPD exacerbation**				
Moderate	127 (0.53)	104 (0.44)	0.678 (0.471–0.976)	0.591 (0.383–0.914)
Severe	91 (0.38)	81 (0.34)	0.833 (0.572–1.215)	0.818 (0.545–1.230)
Moderate to Severe	165 (0.69)	141 (0.59)	0.643 (0.440–0.941)	0.571 (0.368–0.886)

*BV* Broncho-Vaxom, *COPD* Chronic obstructive pulmonary disease, *OR* Odd ratio

Subgroup analysis revealed that the risk of moderate exacerbation decreased in patients aged > 75 years, and in those with one previous moderate exacerbation, no history of pneumonia, a history of asthma, or an mCCI score ≥ 2 (Supplementary Material 6.1.1.1). None of the subgroups benefitted from BV regarding severe exacerbation (Supplementary Material 6.2.1.2). Patients who used triple therapy, those aged > 75 years, females, and those with no history of exacerbations or pneumonia, a history of asthma, or an mCCI score ≥ 2 had a lower moderate-to-severe exacerbation risk after using BV (Supplementary Material 6.2.1.3).

### Differences in exacerbation frequency and incidence

No significant difference in overall exacerbation frequency was observed between the pre-BV and post-BV periods, regardless of exacerbation severity (Supplementary Material 6.2.2). Subgroup analysis revealed that an age ≥ 75 years and mCCI score ≥ 2 were associated with a significantly lower frequency of moderate and moderate-to-severe exacerbations during BV use.

The use of BV was associated with lower incidence rates of moderate and moderate-to-severe exacerbations (IRR = 0.75, *p* = 0.03; and IRR = 0.77, *p* = 0.03, respectively), but not of severe exacerbations (Table 3). In the subgroup analysis, patients aged > 75 years, and those with an mCCI score ≥ 2, benefitted from BV in terms of moderate and moderate-to-severe exacerbations (Supplementary Material 6.2.3).

**Table 3:** Incidence rate of COPD exacerbation between pre- and post-BV period

	**Incidence rate per 1,000 PYs**		**Incidence risk ratio (IRR)**	***P*-value**
	**pre-BV**	**post-BV**		
**COPD exacerbation**				
Moderate	804.75	604.64	0.75	0.03
Severe	473.19	423.73	0.90	0.47
Moderate to Severe	1247.18	965.18	0.77	0.03

*PY* Person-year, *BV* Broncho-Vaxom, *COPD* Chronic obstructive pulmonary disease, *IRR* Incidence rate ratio

### Differences in time to first exacerbation between the pre- and post-BV periods

**Fig. 3:**
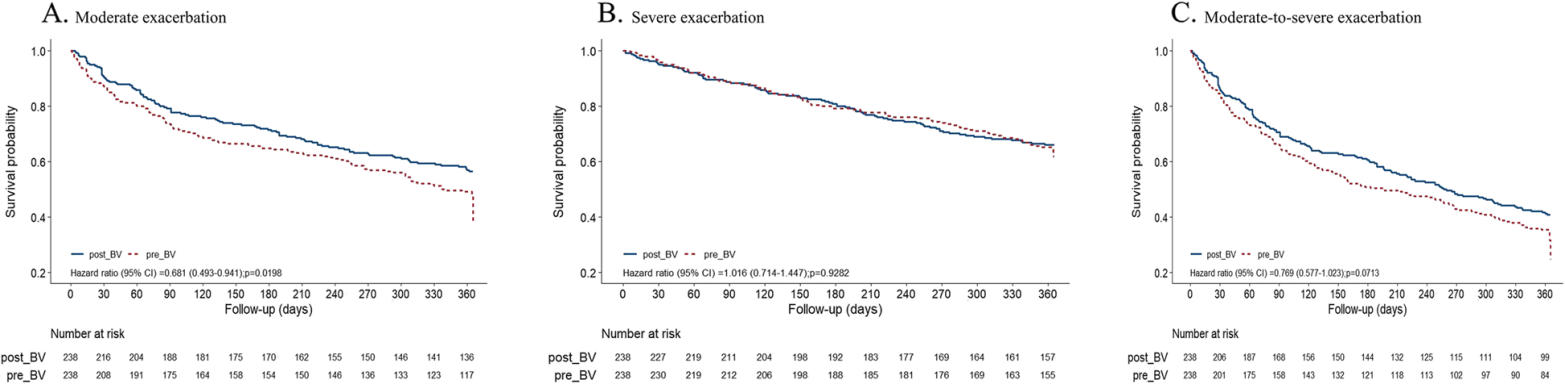
Time to first COPD exacerbation. (**A** Moderate exacerbation; **B** Severe exacerbation; **C** Moderate to severe exacerbation)

A comparison of the time to first exacerbation between the pre- and post-BV periods is shown in Fig. 3. The use of BV delayed moderate exacerbations (hazard ratio [HR] = 0.68, *p* = 0.02), but not moderate-to-severe (HR = 0.77, *p* = 0.07) or severe exacerbations (HR = 1.02, *p* = 0.03).

## Discussion

In this nationwide study, we compared pre- and post-BV exacerbations in BV users. Our results showed that the risk of moderate and moderate-to-severe exacerbations was significantly reduced by BV. Additionally, BV reduced the incidence rates of moderate and moderate-to-severe exacerbations, and delayed the time to the first moderate exacerbation. However, BV did not have a significant effect on severe exacerbations.

Various immunomodulatory substances, including BV, polyvalent mechanical bacterial lysate, pidotimod, probiotics, non-typeable Haemophilus influenza protein vaccine, and MV-130 have been introduced to treat chronic inflammatory airway diseases [10, 11, 23]. These agents are believed to exert their effects through the so-called “gut-lung axis”, where the mucosal immune systems of the gut and lungs function as an integrated unit defending against pathogens that can breach either mucosa [10, 24]. Pathogens are detected by the pattern recognition receptors of dendritic cells (DCs) in mucosal membranes. DCs function as immune sentinels, activating the immune cascade and promoting the migration of innate and adaptive immune cells through the lymphatic system. During this process, not only proinflammatory cells, but also immune regulatory cells, migrate to remote mucosal membranes, which reduces inflammation. A good balance between immunostimulation and immunoregulation may optimize the response to various insults.

BV (OM-85) is an oral bacterial lysate consisting of lyophilized lysates from 21 different bacterial strains that mostly affect the human respiratory tract; they include Streptococcus pneumonia, Haemophilus influenza, Moraxella catarrhalis. Staphylococcus aureus, and Klebsiella pneumonia. The mechanism of action of BV has been widely studied in various experimental models. Parola et al. showed that BV selectively activates NF-kB and the MAPK-dependent pathway in human DCs, which induces chemokines (i.e., CXCL8, CXCL6, CCL3, CCL20, and CCL22) to recruit immune effector cells and enhances humoral immunity by releasing B-cell activating cytokines (i.e., IL-6, IL-10, and BAFF) [18]. Additionally, BV increases IFNα, which has an important role in defending against viral infections. In a murine model, Pasquali et al. reported a substantial decrease in viral load after 5 days in a BV group after infection with the influenza virus [19]. Moreover, the proportion of neutrophils in bronchoalveolar lavage fluid decreased, while the number of CD8 + T-lymphocytes increased, on days 5 and 10 post-infection, respectively, reflecting activation of the innate and adaptive cellular immune responses by BV. These results indicate that BV has a multi-faceted mechanism of action.

COPD exacerbations are associated with a greater disease burden, including reduced quality of life, increased healthcare costs, an accelerated decline of lung function, and increased mortality [3–6]. Among the various methods used to reduce the risk of exacerbations, BV administration has shown promising outcomes, consistent with our results. Soler et al. performed a double-blind randomized placebo-controlled trial in patients with chronic bronchitis or mild COPD [21]. The BV group had a lower cumulative rate of exacerbations (relative risk [RR] = 0.71, *p* = 0.03) and greater proportion of patients free from exacerbations (*p* = 0.01) compared to the control group at the 6-month follow-up. Li et al. performed a double-blind randomized controlled trial of patients with chronic bronchitis and mild COPD. At the 1-year follow-up, the BV group had fewer, shorter-duration and less severe exacerbations, and a shorter duration of antibiotic use [25]. Pan et al. performed a meta-analysis showing that BV decreased the risk of exacerbations (RR = 0.80, 95% CI: 0.65–0.97) [13]. Our study showed that the risk of moderate-to-severe exacerbations decreased in response to BV (OR = 0.57).

Our results indicated that BV may be more effective in reducing moderate than severe exacerbations, and thus may be more effective in those with a lower disease burden. Most of the RCTs that showed favorable effects of BV in terms of reducing the risk of exacerbations included patients with mild COPD; RCTs that included only severe COPD patients failed to show significant reductions in exacerbation risk [21, 22, 25]. Furthermore, the duration of hospitalization did not differ between BV users and non-users in the subgroup analysis of the meta-analysis by Pan et al., which accorded with our result that BV had no beneficial effect on severe exacerbations [13]. Additionally, our study showed that BV had a more significant effect on exacerbations in those aged ≥ 75 years with a high comorbidity burden. As no study has definitively identified subgroups that may benefit from BV, further investigations are needed.

This study had some limitations. First, it was a retrospective observational study, and several types of bias may thus be present. However, because the HIRA database includes almost all patients in South Korea and has minimal missing data, selection and observer biases were probably well controlled. Nevertheless, the single-arm design and inclusion only of patients who used BV may have led to some selection bias. Second, we analyzed 1-year follow-up data of patients who used BV; longer-term outcomes should be investigated in a future study. Third, since COPD was defined by the ICD-10 code and drug usage, there is a possibility that some patients might have been misdiagnosed with COPD. We relied on our operational definition for COPD due to the absence of lung function data in the HIRA database. Nevertheless, this definition has been frequently utilized in prior research [26–40].Strengths of our study included the careful selection of patients from a database including almost all patients in South Korea, based on inclusion criteria that have been used in previous studies; we excluded subjects whose data may have confounded the results. Second, we analyzed the effects of BV on the risk of exacerbations according to severity and type. We also performed a subgroup analysis to identify patients most likely to benefit from BV.

In conclusion, we analyzed the nationwide HIRA database to determine the efficacy of BV for reducing exacerbations in COPD patients. BV use was associated with a significantly lower risk of moderate and moderate-to-severe exacerbations, and a delay in the first moderate exacerbation. However, no effect on severe exacerbations was detected. Given the need to prevent exacerbations in COPD patients, our data are important in showing that BV may reduce the exacerbation risk if used as an adjunct to standard therapy.

### Supplementary Information

**Additional file 1.** 

## Data Availability

The data that support the findings of this study are available from the Health Insurance Review & Assessment Service in Korea, but restrictions apply to the availability of these data, which were used under license for the current study, and so are not publicly available. However, the data are available from the corresponding author on reasonable request.

## Abbreviations

COPD: Chronic obstructive pulmonary disease
BV: Broncho-Vaxom
ICS: Inhaled corticosteroid
EPOS: European Position Paper on Rhinosinusitis and Nasal Polyps
HIRA: Health Insurance Review & Assessment Service
LABA: Long-acting beta-agonist
LAMA: Long-acting muscarinic antagonist
ICD-10: International Classification of Disease-10
mCCI: Modified Charlson Comorbidity Index
IRR: Incidence rate ratio
OCS: Oral corticosteroid
OR: Odd ratio
DC: Dendritic cell
