# Supplementary material for: Effect of Broncho-Vaxom (OM-85) on the frequency of chronic obstructive pulmonary disease (COPD) exacerbations
Source: BMC Pulm Med. 2023 Oct 7;23:378. doi: 10.1186/s12890-023-02665-4 (PMC10559651; doi:10.1186/s12890-023-02665-4)
Supplement: Supplementary file 1 — Additional file 1. [file 12890_2023_2665_MOESM1_ESM.docx]

**Effect of OM-85 on frequency of chronic obstructive pulmonary disease (COPD) exacerbation**

**1.Study designs**

**1.1. Study population**

**1.1.1. Inclusion criteria**

Patients who met the following criteria will be included in this study:

- Patients who were aged ≥ 40 years as of the index date
- Patients who had been diagnosed with COPD and prescribed with ≥ 1 ICS/LABA or LAMA during the study period
  - Defined as patients who had ≥1 inpatient or outpatient claim of ICS/LABA or LAMA
    - World Health Organization-anatomical classification codes (WHO-ATC) for ICS/LABA: R03AK06, R03AK07, R03AK08, R03AK10, R03AK11, R03AK12
    - WHO-ATC for LAMA: R03BB04, R03BB05, R03BB06, R03BB07
  - Defined as patients who had ≥1 inpatient or outpatient claims with International Classification of Disease-10 code (ICD-10 code) for COPD recorded as any diagnosis in inpatient claims or primary ~ 4^th^ secondary diagnosis in outpatient calim during the selection period
    - ICD-10 codes for COPD: J43.x-J44.x (except J43.0)

The list of Korean Classification of Diseases (KCD) codes for the COPD and the Health Insurance Review and Assessment (HIRA) molecule codes for ICS/LABA and LAMA are provided in Appendix 7.1, Table 7-1.

**1.1.2. Exclusion criteria**

Among the user group, patients who meet the following criteria will were excluded from the analysis population.

- Patients who had been prescribed with Uro-Vaxom® or Ismigen® anytime during the period from the baseline period to the post-bronchovaxom period (including index date 1&2)
  - Defined as patients who had ≥1 inpatient or outpatient claims with Uro-Vaxom® (standardized lyophilized bacterial lysates of e.coli) or Ismigen® (standardized lyophilized mixed bacterial lysate, sublingual tablet) during the period from the baseline period to the post-bronchovaxom period (detailed codes listed in Appendix 7.1, Table 7-2)
    - WHO-ATC code for Uro-Vaxom®: L03AX
    - WHO-ATC code for Ismigen®: L03AX
- Patients who had been diagnosed with lung cancer or had undergone lung transplantation during the period from the baseline period to the post-bronchovaxom period (including index date 1&2)
  - Lung cancer (detailed codes listed in Appendix 7.1, Table 7-2)

Defined as patients who had ≥1 inpatient or outpatient claims with ICD-10 codes for lung cancer as any diagnosis in inpatient claims or primary ~ 4th secondary diagnosis in outpatient claims during the period from the baseline period to the post-bronchovaxom period

- - - ICD-10 codes for lung cancer: C34.x (detailed codes listed in Appendix 7.1, Table 7-2)
  - Lung transplantation (detailed codes listed in Appendix 7.1, Table 7-2)

Defined as patients who had ≥1 inpatient or outpatient claims with ICD-10 codes for lung transplantation recorded as any diagnosis in inpatient claims or primary ~ 4th secondary diagnosis in outpatient claims during the period from the baseline period to the post-bronchovaxom period, OR

- - - ICD-10 codes for lung transplantation: Z94.2

Defined as patients who had ≥1 inpatient or outpatient claims with procedure codes for lung transplantation during the period from the baseline period to the post-bronchovaxom period

- - - Procedure codes for lung transplantation: Q8091.x, Q8092.x, Q8101.x, Q8102.x, Q8103.x
- Patients who had been diagnosed with IPF or ILD during the period from the baseline period to the post-bronchovaxom period (including index date 1&2)
  - IPF and ILD (detailed codes listed in Appendix 7.1, Table 7-2)

Defined as patients who had ≥1 inpatient or outpatient claims with ICD-10 codes for IPF or ILD recorded as any diagnosis in inpatient claims or primary ~ 4th secondary diagnosis in outpatient claims during the period from the baseline period to the post-bronchovaxom period

- - - ICD-10 codes for IPF: J84.x
    - ICD-10 codes for ILD: J70.2-J70.4
- Pateints who had been prescribed with Broncho-Vaxom® during the pre-bronchovaxom period or the index date 2 (excluding index date 2)
  - Defined as patients who had ≥1 inpatient or outpatient claims with Broncho-Vaxom® during the pre-bronchovaxom period or the index date 2 (detailed codes listed in Appendix 7.1, Table 7-2)
    - WHO-ATC code for Broncho-Vaxom®: L03AX
- Patients who had died during the period from the baseline period to the post-bronchovaxom period (including index date 1&2)
  - Defined as the date of death recorded as any diagnosis OR with death recorded as diagnosis result type
    - ICD-10 codes for death: I46.1, R96.x, R98.x, R99.x
    - Diagnosis result type code: 04 (dead)
- Patients who received different types of inhalers or less MPR ≥ 50% of same type of inhaler between or either in pre- and post- bronchovaxom period
  - ICS/LABA (detailed codes listed in Appendix 7.1, Table 7-2)

Defined as patients who received MPR ≥ 50% ICS/LABA in the pre-bronchovaxom period and the post-bronchovaxom period

- - - WHO-ATC for ICS/LABA: R03AK06, R03AK07, R03AK08, R03AK09, R03AK10, R03AK11, 03AK12
  - LAMA (detailed codes listed in Appendix 7.1, Table 7-2)

Defined as patients who received MPR ≥ 50% LAMA in the pre-bronchovaxom period and the post-bronchovaxom period

- - - WHO-ATC for LAMA: R03BB04, R03BB05, R03BB06, R03BB07
  - Triple combination (detailed codes listed in Appendix 7.1, Table 7-2)

Defined as patients who received MPR ≥ 50% the combination of ICS/LABA + LAMA issued on the same date in the same prescription in the pre-bronchovaxom period and the post-bronchovaxom period

- - - WHO-ATC for ICS/LABA: R03AK06, R03AK07, R03AK08, R03AK10, R03AK11, R03AK12
    - WHO-ATC for LAMA: R03BB04, R03BB05, R03BB06, R03BB07
  - LABA/LAMA (detailed codes listed in Appendix 7.1, Table 7-2)

Defined as patients who received MPR ≥ 50% LABA/LAMA or the combination of LABA + LAMA issued on the same date in the same prescription in the pre-bronchovaxom period and the post-bronchovaxom period

- - - WHO-ATC for LABA/LAMA: R03AL03, R03AL04, R03AL05, R03AL06
    - WHO-ATC for LABA: R03AC12, R03AC13, R03AC18
    - WHO-ATC for LAMA: R03BB04, R03BB05, R03BB06, R03BB07

**2. Variables**

**2.1 Exposure variables**

**2.1.1 Exposure drug**

The exposure drug is Broncho-Vaxom® in this study. Eligible patients who had MPR ≥ 40% for the exposure drug during the selection period will be considered as exposed. Among the exposed group, the first prescription date will be defined as the index date.

The relevant HIRA molecule codes are listed in the Appendix 7.2, Table 7-3

WHO ATC Code for Broncho-Vaxom®: R07AX

**2.1.2 Exposure period**

The following assumptions are employed to define the exposure period for this study:

- Adherence rate will be defined using the medication possession ratio (MPR) for the 12-month period following the index date (i.e. follow-up period)
  - MPR is defined as the total sum of days’ supply for all fills during the 12-month period following the index date

$$MPR=\frac{Sum of \mathrm{days}^{'}supply during the follow-up period (12 months)}{Number of days (12 months) from the index date}$$

- - Sum of days’ supply refers to the total prescribed amount
- Good adherence will be defined as having an MPR of ≥ 40% for Broncho-Vaxom®
  - According to the treatment gudelines, Broncho-Vaxom® are generally prescribed for 3 months followed by 3 months of withdrawal period (e.g. a 3-month prescription will require a 3-month treatment break, which results in a 6-month treatment regimen). Since this study has a 12-month follow-up duration, Broncho-Vaxom® users will be required to have MPRs ≥ 50% of the follow-up duration (12 months) to be eligible for analysis.
  - Since MPRs ≥ 80% are considered adherent, MPRs ≥ 40% (= 50% of the 80%) will be used to assess good adherence for Broncho-Vaxom®. In other words, good adherence will be defined as having an MPR ≥ 40% of the follow-up duration (i.e. 40% of 12 months).

Since eligibility of the study for the user group is limited to the patients with good adherence, the exposure period will be defined as the 12-month follow-up period from the initiation of Broncho-Vaxom® for user group. The exposure end date will be defined as the end date of the follow-up period of 12 months.

**2.2 Outcome(s)**

The primary outcome is incidence, frequency, and time to COPD exacerbation.

**2.2.1 Primary Outcomes**

**2.2.1.1. COPD Exacerbation**

COPD exacerbation will be defined by relevant diagnosis codes and systemic steroid and/or antibiotics prescription data obtained during the observational period. The pre-defined conditions (i.e. relevant diagnosis codes, systemic steroids and/or antibiotics) MUST be recorded in a same claim issued on same date. Events observed on the index date will not be considered. Detailed codes and definitions are listed in Appendix 7.3, Table 7-4.

COPD exacerbation will be classified as moderate, severe, or moderate to severe exacerbation. Moderate, severe, and moderate to severe exacerbations will further be categorized according to anti-biotics use and oral corticosteroid use.

The events occurred on the index date will not be considered as COPD exacerbation events.

**2.2.1.1.1 Moderate COPD exacerbation**

Moderate COPD exacerbation will be defined as ≥ 1 outpatient claim(s) with 1) ICD-10 codes for COPD recorded as primary ~ 4^th^ secondary diagnosis AND 2) systemic steroids and/or antibiotics prescriptions observed during the 12-month follow-up after the index date.

- ICD-10 codes for COPD: J43.x-J44.x (except J43.0)
- WHO-ATC for systemic steroids: H02AB01, H02AB02, H02AB04, H02AB06, H02AB08, H02AB09, H02AB13
- WHO-ATC for antibiotics: J01.x, J02A.x, J04A.x

Date of moderate COPD exacerbation event will be defined as the starting date of an outpatient claim with the above 2 conditions used to define moderate COPD exacerbation event

To assess the incidence of moderate COPD exacerbation event, the first moderate exacerbation event observed during the follow-period will be considered. The follow-up will end when event occurs or the 12 month-period ends, whichever occurs first.

To assess the frequency of moderate COPD exacerbation event episodes, any moderate exacerbation events observed during follow-up period (i.e. 12 months after the index date) will be considered.

The following events will be considered as a single episode of moderate COPD exacerbation:

- Consecutive outpatient claims with the above 2 pre-defined conditions of moderate COPD exacerbation that are less than 14 days apart
  - Outpatient visits due to moderate COPD exacerbation will be defined as ≥ 1 outpatient claim(s) with 1) ICD-10 codes for COPD recorded as primary ~ 4th secondary diagnosis AND 2) systemic steroids and/or antibiotics prescriptions observed during the 12-month follow-up after the index date

The following events will not be considered as episodes of moderate COPD exacerbation:

- Outpatient claims with the above 2 pre-defined conditions of moderate COPD exacerbation that had been issued within 14 days of being discharged from a previous hospital admission or emergency room (ER) visit due to severe COPD exacerbation
  - Hospital admission or ER visit due to severe COPD exacerbation will be defined as ≥ 1 inpatient claim(s) or ER visit(s) with 1) ICD-10 codes for COPD or diseases due to COPD worsening as any diagnosis AND 2) systemic steroids and/or antibiotics prescriptions observed during the 12-month after the index date.

**2.2.1.1.2 Severe COPD exacerbation**

Severe COPD exacerbation will be defined as ≥ 1 inpatient claim(s) or ER visit(s) with 1) ICD-10 codes for COPD or diseases due to COPD worsening as any diagnosis AND 2) systemic steroids and/or antibiotics prescriptions observed during the 12-month follow-up after the index date.

- ICD-10 codes for COPD: J43.x-J44.x (except J43.0)
- ICD-10 codes for diseases due to COPD worsening
  - Pneumonia (J12.x-J18.x, J69.0, J84.0, J84.9, J85.1, B96.0)
  - Pulmonary thromboembolism (I26, I26.0, and I26.9)
  - Dyspnea (R06.0), Or
  - Acute respiratory distress syndrome (J80)
- WHO-ATC for systemic steroids: H02AB01, H02AB02, H02AB04, H02AB06, H02AB08, H02AB09, H02AB13
- WHO-ATC for antibiotics: J01.x, J02A.x, J04A.x

ER visit will be identified by procedure codes or specialty code for ER.

- Procedure codes for ER
  - Before 2015: AC101.x, AC103.x, AC105.x,
  - After 2016: V1.x, V2.x, V3.x, V4.x, V5.x
- Specialty code for ER: 24 (department of emergency medicine)

Date of severe COPD exacerbation event will be defined as the starting date of ER claims or admission date of inpatient claims with the above 2 conditions to define severe COPD exacerbation event

To assess the incidence of severe COPD exacerbation event, the first severe exacerbation event observed during the follow-period will be considered. The follow-up will end when event occurs or the 12 month-period ends, whichever occurs first.

To assess the frequency of severe COPD exacerbation event episodes, any severe exacerbation events observed during follow-up period (i.e. 12 months after the index date) will be considered.

The following events will be considered as separate severe COPD exacerbation event episodes:

- Inpatient claims with the above 2 pre-defined conditions of severe COPD exacerbation occurred after discharging from a previous inpatient visit due to severe COPD exacerbation
  - Inpatient visit due to severe COPD exacerbation will be defined as ≥ 1 inpatient claims 1) with ICD-10 codes for COPD or diseases due to COPD worsening as any diagnosis, AND 2) with systemic steroids and/or antibiotics prescription within 12 months after the index date
- ER claims with the above 2 pre-defined conditions of severe COPD exacerbation occurred after a previous ER visit due to severe COPD exacerbation
  - ER visit due to severe COPD exacerbation will be defined as ≥ 1 ER claims 1) with ICD-10 codes for COPD or diseases due to COPD worsening as any diagnosis, AND 2) with systemic steroids and/or antibiotics prescription within 12 months after the index date

**2.2.1.1.3 Moderate to severe COPD exacerbation**

Moderate to severe COPD exacerbation will be defined as a composition event of moderate and severe COPD exacerbation. The same definition on moderate and severe COPD exacerbation described in Section 2.2.1.1.1 and Section 2.2.1.1.2 will be used.

To assess the incidence of moderate to severe COPD exacerbation event, the first COPD exacerbation event among pre-defined moderate COPD exacerbation and severe COPD exacerbation event observed during the 12-month period following the index date will be considered. The follow-up will end when event occurs or the 12 month-period ends, whichever occurs first.

To assess the frequency of moderate to severe COPD exacerbation events, any events observed during the follow-up period (i.e. 12 months after the index date) will be considered.

**2.2.1.1.4 Antibiotics-used COPD exacerbation**

Antibiotics-used moderate COPD exacerbation will be defined as ≥ 1 outpatient claim(s) with 1) ICD-10 codes for COPD recorded as primary ~ 4^th^ secondary diagnosis AND 2) antibiotics prescriptions observed during the 12-month follow-up after the index date.

Antibiotics-used severe COPD exacerbation will be defined as as ≥ 1 inpatient claim(s) or ER visit(s) with 1) ICD-10 codes for COPD or diseases due to COPD worsening as any diagnosis AND 2) antibiotics prescriptions observed during the 12-month follow-up after the index date.

Same definitions used in Section 2.2.1.1.1 and 2.2.1.1.2 will be used for separating and combining of event episodes

- Antibiotics-used moderate COPD exacerbation

To assess the incidence of antibiotics-used moderate COPD exacerbation event, the first antibiotics-used moderate exacerbation event observed during the follow-period will be considered. The follow-up will end when event occurs or the 12 month-period ends, whichever occurs first.

To assess the frequency of antibiotics-used moderate COPD exacerbation event episodes, any antibiotics-used moderate exacerbation events observed during follow-up period (i.e. 12 months after the index date) will be considered.

- Antibiotics-used severe COPD exacerbation

To assess the incidence of severe COPD exacerbation event, the first antibiotics-used severe exacerbation event observed during the follow-period will be considered. The follow-up will end when event occurs or the 12 month-period ends, whichever occurs first.

To assess the frequency of antibiotics-used severe COPD exacerbation event episodes, any severe exacerbation events observed during follow-up period (i.e. 12 months after the index date) will be considered.

- Antibiotics-used moderate to severe COPD exacerbation

To assess the incidence of antibiotics-used moderate to severe COPD exacerbation event, the first antibiotics-used COPD exacerbation event among pre-defined antibiotics-used moderate COPD exacerbation and antibiotics-used severe COPD exacerbation event observed during the 12-month period following the index date will be considered. The follow-up will end when event occurs or the 12 month-period ends, whichever occurs first.

To assess the frequency of antibiotics-used moderate to severe COPD exacerbation events, any events observed during the follow-up period (i.e. 12 months after the index date) will be considered.

**2.2.1.1.5. Oral corticosteroid(OCS)-used COPD exacerbation**

OCS-used moderate COPD exacerbation will be defined as ≥ 1 outpatient claim(s) with 1) ICD-10 codes for COPD recorded as primary ~ 4^th^ secondary diagnosis AND 2) OCS prescriptions observed during the 12-month follow-up after the index date.

OCS-used severe COPD exacerbation will be defined as ≥ 1 inpatient claim(s) or ER visit(s) with 1) ICD-10 codes for COPD or diseases due to COPD worsening as any diagnosis AND 2) OCS prescriptions observed during the 12-month follow-up after the index date.

Same definitions used in Section 2.2.1.1.1 and 2.2.1.1.2 will be used for separating and combining of event episodes

- OCS-used moderate COPD exacerbation

To assess the incidence of OCS-used moderate COPD exacerbation event, the first OCS-used moderate exacerbation event observed during the follow-period will be considered. The follow-up will end when event occurs or the 12 month-period ends, whichever occurs first.

To assess the frequency of OCS-used moderate COPD exacerbation event episodes, any OCS-used moderate exacerbation events observed during follow-up period (i.e. 12 months after the index date) will be considered.

- OCS-used severe COPD exacerbation

To assess the incidence of severe COPD exacerbation event, the first OCS-used severe exacerbation event observed during the follow-period will be considered. The follow-up will end when event occurs or the 12 month-period ends, whichever occurs first.

To assess the frequency of OCS-used severe COPD exacerbation event episodes, any severe exacerbation events observed during follow-up period (i.e. 12 months after the index date) will be considered.

- OCS-used moderate to severe COPD exacerbation

To assess the incidence of OCS-used moderate to severe COPD exacerbation event, the first OCS-used COPD exacerbation event among pre-defined OCS-used moderate COPD exacerbation and OCS-used severe COPD exacerbation event observed during the 12-month period following the index date will be considered. The follow-up will end when event occurs or the 12 month-period ends, whichever occurs first.

To assess the frequency of OCS-used moderate to severe COPD exacerbation events, any events observed during the follow-up period (i.e. 12 months after the index date) will be considered.

**2.2.1.1.6. Antibiotics and OCS-used COPD exacerbation**

Antibiotics and OCS-used moderate COPD exacerbation will be defined as ≥ 1 outpatient claim(s) with 1) ICD-10 codes for COPD recorded as primary ~ 4^th^ secondary diagnosis AND 2) OCS and antibiotics prescriptions observed during the 12-month follow-up after the index date.

Antibiotics and OCS-used severe COPD exacerbation will be defined as as ≥ 1 inpatient claim(s) or ER visit(s) with 1) ICD-10 codes for COPD or diseases due to COPD worsening as any diagnosis AND 2) OCS and antibiotics prescriptions observed during the 12-month follow-up after the index date.

Same definitions used in Section 2.2.1.1.1 and 2.2.1.1.2 will be used for separating and combining of event episodes

- Antibiotics and OCS-used moderate COPD exacerbation

To assess the incidence of antibiotics and OCS-used moderate COPD exacerbation event, the first antibiotics and OCS-used moderate exacerbation event observed during the follow-period will be considered. The follow-up will end when event occurs or the 12 month-period ends, whichever occurs first.

To assess the frequency of antibiotics and OCS-used moderate COPD exacerbation event episodes, any antibiotics and OCS-used moderate exacerbation events observed during follow-up period (i.e. 12 months after the index date) will be considered.

- Antibiotics and OCS-used severe COPD exacerbation

To assess the incidence of severe COPD exacerbation event, the first antibiotics and OCS-used severe exacerbation event observed during the follow-period will be considered. The follow-up will end when event occurs or the 12 month-period ends, whichever occurs first.

To assess the frequency of antibiotics and OCS-used severe COPD exacerbation event episodes, any severe exacerbation events observed during follow-up period (i.e. 12 months after the index date) will be considered.

- Antibiotics and OCS-used moderate to severe COPD exacerbation

To assess the incidence of antibiotics and OCS-used moderate to severe COPD exacerbation event, the first antibiotics and OCS-used COPD exacerbation event among pre-defined antibiotics and OCS-used moderate COPD exacerbation and antibiotics and OCS-used severe COPD exacerbation event observed during the 12-month period following the index date will be considered. The follow-up will end when event occurs or the 12 month-period ends, whichever occurs first.

To assess the frequency of antibiotics and OCS-used moderate to severe COPD exacerbation events, any events observed during the follow-up period (i.e. 12 months after the index date) will be considered.

**2.2.1.2 Time to COPD exacerbation**

Time to first COPD exacerbation events will be analyzed, classified and categorized as following:

- Time to moderate COPD exacerbation
- Time to severe COPD exacerbation,
- Time to moderate to severe COPD exacerbation
- Time to antibiotics-used COPD exacerbation (as moderate, severe, and moderate to severe)
- Time to OCS-used COPD exacerbation (as moderate, severe, and moderate to severe)
- Time to antibiotics and OCS-used COPD exacerbation (as moderate, severe, and moderate to severe)

Same definitions used in Section 2.2.1.1 will be used for defining the respective events.

**2.3 Covariates**

The demographic characteristics and potential confounders will be included as covariables.

2.3.1. Demographic characteristics

The demographic characteristics will include age, sex, and economic status as of the index date and detailed definitions are as follows:

- Age: patient’s age as of the index date
- Sex: patient’s sex on the index date (male or female)
- Economic status: patient’s insurance type as of the year of the index date
- Index year: the year of the index date

2.3.2. Clinical characteristics

The clinical characteristics will include history of COPD exacerbation, history of asthma, and comorbidity observed during the baseline period (12-month period prior to index date). Events observed on the index date will not be considered. Detailed definitions are as follows:

- History of COPD exacerbation
  - Defined in the same way as for COPD exacerbation of primary outcomes observed during the 12-month baseline period prior to the index date (detailed codes listed in Appendix 7.3, Table 7-4)
  - Categorized as those with no COPD exacerbation, 1 moderate COPD exacerbation, and 2≥ moderate or 1≥ severe COPD exacerbation
- History of pneumonia
  - Defined as ≥1 inpatient or outpatient claims 1) with ICD-10 codes for pneumonia recorded as any diagnosis in inpatient claims or primary ~ 4th secondary diagnosis in outpatient claims, AND 2) with diagnostic test code for chest-X ray or chest-CT, AND 3) with antibiotics prescription during the 12-month baseline period prior to the index date (detailed codes listed in Appendix7.4, Table 7-6)
  - ICD-10 codes for pneumonia: J12.x-J18.x, J69.0, J84.0, J84.9, J85.1, B96.0
  - Diagnostic test codes for chest-x ray: G2101.x-G2105.x
  - Diagnostic test codes for chest-CT: HA424.x, HA434.x, HA454.x, HA464.x, HA474.x, HA484.x, HA834.x
  - World health organization-anatomical classification codes [WHO-ATC] for antibiotics: J01.x, J02A.x, J04A.x
- History of asthma
  - Defined as ≥ 1 outpatient claim(s) with ICD-10 codes for asthma recorded as primary ~ 4^th^ secondary diagnosis OR ≥ 1 inpatient claim(s) with ICD-10 codes for asthma recorded as any diagnosis type during the 12-month baseline period prior to the index date (detailed codes listed in Appendix 7.4, Table 7-7)
  - ICD-10 codes for asthma: J45.x-J46.x
- Comorbidity
  - Defined by the ICD-10 codes of relevant diseases recorded as primary ~ 4^th^ secondary diagnosis during the 12-month baseline period prior to the index date
  - modified Charlson Comorbidity Index (mCCI) will be determined using the ICD-10 codes of relevant diagnoses recorded during the 12-month baseline period prior to the index date
  - ICD-10 codes for mCCI calculation are listed in Appendix 7.4, Table 7-8.

**3. Data source and management**

**3.1 Data Source**

For statistical analyses, patient data will be retrieved from the National Health Insurance claims data of HIRA from 01 January 2015 to 31 December 2019.

The HIRA national claims database contains the demographic and medical claim information of more than 50 million Korean population. South Korea has a single-payer, universal and compulsory health coverage system, which covers approximately 98% of the entire Korean population. As of 2011, the HIRA claims database contains approximately 46 million patients per year, which accounts for 90% of the entire South Korean population and includes healthcare insurance claims from almost 80,000 healthcare service providers.

In South Korea, healthcare service providers electronically submit claims to HIRA which then reviews and makes reimbursement decisions based on coverage criteria of National Health Insurance Service (NHIS). In addition, the claims from patients with medical aid program, government expenditures, and veteran patients are also included in the claims data.

The database consists of five tables:

- Data-table 20 that includes general information such as gender, age and insurance type, indicators for inpatient and outpatient services
- Data-table 30 that includes specific information on the provided healthcare services
- Data-table 40 that includes specific information on diagnosis
- Data-table 53 that includes specific information on outpatient prescriptions
- Data-table of providers that includes specific information on the healthcare service providers

Personal identifying information of patients and providers is coded and de-identified to protect patient privacy and all information was linked using de-identified patient and provider code.

**3.2 Data management**

Data is extracted from the HIRA and stored on a secured, shared server for statistical analyses. The original data will be stored in its own, read-only directory to protect the integrity of the extracted data. After obtaining an approval from the HIRA, data analysts can access the data and conduct analysis in a secured computer. HIRA’s policy strictly forbids exportation of the original data, however, results obtained from statistical analyses can be exported after an internal review.

**3.3 Missing Data**

This study will rely on secondary data collected for the purpose of providing medical care and payment for care provided, which are known to have a high degree of completeness. However, the amount of missing values for data elements will be reported. No imputation method for missing data will be used.

When performing multivariable analysis, patients with missing data on any covariate will be excluded and this will be reported in the corresponding table.

**4. Statistical analysis**

All statistical tests will be two-sided with a significance level of 0.05. The statistical analysis will be performed using SAS® 9.4 software (SAS Institute, North Carolina, US) via SAS Enterprise Guide version 6.1. The statistical results will be presented as tables, and/or graphs. Figures will be produced using SAS®.

4.1 Demographics and baseline characteristic analysis

A descriptive analysis will be performed to evaluate demographic and baseline characteristics of the patients. Variables will include age, sex, economic status (i.e. insurance type), inhaler, history of COPD exacerbation, history of asthma, comorbidities.

Continuous variables will be summarized using the mean, standard deviation (SD), median, minimum and maximum, and 25 and 75 percentiles. Categorical variables will be summarized using frequencies and proportion (%). Proportion will not include missing category and will be calculated over the number of subjects with available (non-missing) data. If there is no observation, a hyphen ‘-‘ will be displayed with no other statistics.

- Continuous variables
  - Mean and standard deviation (SD)
  - Median
  - Minimum and maximum
  - 25th and 75th percentile
- Categorical variables
  - Numbers
  - Percentages

Chi-square test for categorical variables and t-test or Wilcoxon rank sum test for continuous variables will be conducted to compare the baseline characteristics of study groups.

Patient baseline characteristics and a standardized difference before and after matching will be presented.

**4.2 Outcome analysis**

For analysis, the primary outcomes on COPD exacerbation will be presented as the incidence rate per person-years (PYs) and Odds ratio (OR) with corresponding 95% confidence intervals (CI). The frequencies of event episodes will be presented as the number of events per person per year. Time to event will be presented as mean and standard deviation (SD) with descriptive statistics and median and standard error (SE) with Kaplan-Meier analysis.

- COPD exacerbation
  - Incidence rate per PYs for the first event
  - Frequency per PYs
  - Hazard ratio and corresponding CI
- Time to COPD exacerbation
  - Mean and SD
  - Median and SE

The incidence rate for outcomes will be calculated in the same way as for the main analysis. The incidence rate ratio will be further calculated for the subgroup analysis.

- Number of patients with event (cases)
- Sum of event-free period during the follow-up period (person years)
- Incidence rate per person-years (IR/PYs)

= Number of patients with event (cases) / sum of event-free period during the follow-up period (person-years)

- Incidence rate per person-years

= incidence rate for post-bronchovaxom period / incidence rate for pre-bronchovaxom period

- Time to the first event occurrence from the index date (days; mean SD, median, minimum, maximum, 25th percentile, 75th percentile)

Frequencies of events will be calculated in the same way as for the main analysis. The relative frequency will be further calculated for the subgroup analysis.

- Number of events per person per year

= Number of total events (cases) / sum of follow-up duration (person-years)

- Relative frequency

= number of events per person per year for post-bronchovaxom period / number of events per person per year for pre-bronchovaxom period

- Number of total events
- Follow-up duration from the index date

**5. Quality control**

All operational definitions used for this study will be reviewed and confirmed by the study investigators. The principal investigator, the co-investigators and the sponsors of the study must approve all revisions to the statistical analysis plan (SAP). All changes must be documented as SAP amendments.

IQVIA will be responsible for the management and monitoring of the responsible investigators throughout this database study. IQVIA will oversee the participating investigators by means of project management. This includes assessing the study quality and integrity.

The study was approved by the Institutional Review Board of Konkuk university hospital.

Data analyses will be designed and pre-programmed before commencing statistical analyses. Programming logic used in this study will be reviewed and cross-checked by two programmers.

The interim analysis results will be reviewed and discussed by the study investigators. The operational definitions and statistical modelling will be further clarified and amended if necessary.

All analyses will be performed in a manner consistent with the Strengthening the Reporting of Observational Studies in Epidemiology (STROBE) guidelines and applicable sections of the Consolidated Standards of Reporting Trials (CONSORT) guidelines.

**6. Outcomes**

6.1 Differences in various type of exacerbation risk between pre- and post-BV period

|  | **Number of patient with events (%) (n=238)** | | **Crude OR (95% CI)** | **Adjusted OR (95% CI)** |
| --- | --- | --- | --- | --- |
|  | **pre-BV** | **post-BV** |  |  |
| **Antibiotics-used COPD exacerbation** | | | | |
| Moderate | 83 (0.35) | 73 (0.31) | 0.826 (0.562-1.216) | 0.788 (0.507-1.225) |
| Severe | 49 (0.21) | 40 (0.17) | 0.779 (0.489-1.242) | 0.769 (0.465-1.273) |
| Moderate to Severe | 113 (0.47) | 101 (0.42) | 0.816 (0.567-1.174) | 0.790 (0.530-1.178) |
| **OCS-used COPD exacerbation** | | | | |
| Moderate | 40 (0.17) | 26 (0.11) | 0.607 (0.356-1.036) | 0.577 (0.336-0.992) |
| Severe | 12 (0.05) | 17 (0.07) | 1.449 (0.673-3.121) | 1.523 (0.728-3.189) |
| Moderate to Severe | 46 (0.19) | 39 (0.16) | 0.818 (0.509-1.314) | 0.803 (0.488-1.321) |
| **Antibiotics and OCS-used COPD exacerbation** | | | | |
| Moderate | 44 (0.18) | 36 (0.15) | 0.786 (0.483-1.278) | 0.754 (0.431-1.318) |
| Severe | 44 (0.18) | 43 (0.18) | 0.972 (0.609-1.553) | 0.969 (0.577-1.626) |
| Moderate to Severe | 81 (0.34) | 69 (0.29) | 0.791 (0.536-1.169) | 0.761 (0.491-1.179) |

6.2 Subgroup analysis of all COPD AE

6.2.1 Odd ratio of all COPD AE

6.2.1.1 Moderate exacerbation

| **MODERATE COPD EXACERBATION** |  | | | | | | | | | | | | | | |
| --- | --- | --- | --- | --- | --- | --- | --- | --- | --- | --- | --- | --- | --- | --- | --- |
|  | **Pre-BV** | | | | **Post-BV** | | | | **Mcnemar p-value** | **Crude OR** | | | **Adjusted OR** | | |
|  | **Event** | | **No Event** | | **Event** | | **No Event** | |  |  |  |  |  |  |  |
|  | n | % | n | % | n | % | n | % |  | OR | 95% CI | p-value | OR | 95% CI | p-value |
| **Overall** | 127 | 0.53 | 111 | 0.47 | 104 | 0.44 | 134 | 0.56 | 0.0079 | 0.678 | (0.471-0.976) | 0.0366 | 0.591 | (0.383-0.914) | 0.0182 |
| **COPD medication during pre and post-BV period** |  |  |  |  |  |  |  |  |  |  |  |  |  |  |  |
| *ICS/LABA* | 46 | 0.41 | 67 | 0.59 | 42 | 0.37 | 71 | 0.63 | 0.4927 | 0.862 | (0.500-1.484) | 0.5881 | 0.816 | (0.420-1.586) | 0.5452 |
| *LAMA* | 13 | 0.54 | 11 | 0.46 | 8 | 0.33 | 16 | 0.67 | 0.0956 | 0.423 | (0.120-1.491) | 0.1711 | 0.435 | (0.135-1.396) | 0.1532 |
| *Triple* | 48 | 0.72 | 19 | 0.28 | 40 | 0.60 | 27 | 0.40 | 0.0736 | 0.586 | (0.280-1.230) | 0.1549 | 0.430 | (0.163-1.130) | 0.0857 |
| *LABA/LAMA* | 20 | 0.59 | 14 | 0.41 | 14 | 0.41 | 20 | 0.59 | 0.0833 | 0.490 | (0.177-1.356) | 0.1632 | 0.321 | (0.081-1.280) | 0.1041 |
| **Age at index date 1** |  |  |  |  |  |  |  |  |  |  |  |  |  |  |  |
| *40 to < 65* | 41 | 0.61 | 26 | 0.39 | 36 | 0.54 | 31 | 0.46 | 0.2253 | 0.736 | (0.364-1.491) | 0.3895 | 0.643 | (0.265-1.559) | 0.3235 |
| *65 to < 75* | 53 | 0.49 | 56 | 0.51 | 50 | 0.46 | 59 | 0.54 | 0.5900 | 0.895 | (0.522-1.537) | 0.6862 | 0.847 | (0.422-1.700) | 0.6375 |
| *75+* | 33 | 0.53 | 29 | 0.47 | 18 | 0.29 | 44 | 0.71 | 0.0039 | 0.360 | (0.168-0.771) | 0.0094 | 0.229 | (0.082-0.636) | 0.0054 |
| **Sex** |  |  |  |  |  |  |  |  |  |  |  |  |  |  |  |
| *Male* | 101 | 0.53 | 89 | 0.47 | 85 | 0.45 | 105 | 0.55 | 0.0356 | 0.713 | (0.475-1.072) | 0.1036 | 0.636 | (0.391-1.035) | 0.0682 |
| *Female* | 26 | 0.54 | 22 | 0.46 | 19 | 0.40 | 29 | 0.60 | 0.0896 | 0.554 | (0.239-1.285) | 0.1647 | NA | NA | NA |
| **Insurance type at index date2** |  |  |  |  |  |  |  |  |  |  |  |  |  |  |  |
| *Health insurance* | 105 | 0.51 | 102 | 0.49 | 88 | 0.43 | 119 | 0.57 | 0.0322 | 0.718 | (0.486-1.062) | 0.0967 | 0.635 | (0.394-1.022) | 0.0611 |
| *Medical aid* | 22 | 0.71 | 9 | 0.29 | 16 | 0.52 | 15 | 0.48 | 0.0833 | 0.436 | (0.144-1.324) | 0.1374 | 0.446 | (0.123-1.611) | 0.2089 |
| **Hospital type at index date2** |  |  |  |  |  |  |  |  |  |  |  |  |  |  |  |
| *Tertiary and general hospital* | 101 | 0.52 | 95 | 0.48 | 85 | 0.43 | 111 | 0.57 | 0.0422 | 0.720 | (0.482-1.076) | 0.1085 | 0.631 | (0.384-1.037) | 0.0694 |
| *Others* | 26 | 0.62 | 16 | 0.38 | 19 | 0.45 | 23 | 0.55 | 0.0522 | 0.508 | (0.205-1.260) | 0.1398 | 0.291 | (0.080-1.056) | 0.0599 |
| **History of COPD exacerbation** |  |  |  |  |  |  |  |  |  |  |  |  |  |  |  |
| *none* | 43 | 0.33 | 88 | 0.67 | 37 | 0.28 | 94 | 0.72 | 0.3173 | 0.806 | (0.472-1.374) | 0.4245 | 0.770 | (0.394-1.503) | 0.4406 |
| *1 moderate* | 65 | 0.76 | 20 | 0.24 | 48 | 0.56 | 37 | 0.44 | 0.0041 | 0.399 | (0.204-0.783) | 0.0081 | 0.351 | (0.164-0.755) | 0.0079 |
| *≥ 2 moderate OR ≥ 1 severe* | 19 | 0.86 | 3 | 0.14 | 19 | 0.86 | 3 | 0.14 | 1.0000 | 1.000 | (0.154-6.489) | 1.0000 | 1.000 | (0.456-2.192) | 1.0000 |
| **History of Pneumonia** |  |  |  |  |  |  |  |  |  |  |  |  |  |  |  |
| *No* | 83 | 0.47 | 92 | 0.53 | 63 | 0.36 | 112 | 0.64 | 0.0065 | 0.623 | (0.405-0.961) | 0.0324 | 0.540 | (0.320-0.912) | 0.0214 |
| *Yes* | 44 | 0.70 | 19 | 0.30 | 41 | 0.65 | 22 | 0.35 | 0.5127 | 0.805 | (0.373-1.734) | 0.5738 | 0.745 | (0.293-1.896) | 0.5315 |
| **History of Asthma** |  |  |  |  |  |  |  |  |  |  |  |  |  |  |  |
| *No* | 17 | 0.45 | 21 | 0.55 | 13 | 0.34 | 25 | 0.66 | 0.2482 | 0.642 | (0.243-1.695) | 0.3614 | 0.501 | (0.143-1.755) | 0.2710 |
| *Yes* | 110 | 0.55 | 90 | 0.45 | 91 | 0.46 | 109 | 0.55 | 0.0167 | 0.683 | (0.459-1.016) | 0.0599 | 0.589 | (0.365-0.951) | 0.0306 |
| **mCCI** |  |  |  |  |  |  |  |  |  |  |  |  |  |  |  |
| *0, 1* | 68 | 0.59 | 48 | 0.41 | 62 | 0.53 | 54 | 0.47 | 0.2733 | 0.810 | (0.478-1.373) | 0.4313 | 0.740 | (0.391-1.401) | 0.3520 |
| *2 +* | 59 | 0.48 | 63 | 0.52 | 42 | 0.34 | 80 | 0.66 | 0.0113 | 0.561 | (0.332-0.945) | 0.0303 | 0.449 | (0.229-0.880) | 0.0200 |
| **mCCI category** |  |  |  |  |  |  |  |  |  |  |  |  |  |  |  |
| *Congestive heart failure* |  |  |  |  |  |  |  |  |  |  |  |  |  |  |  |
| *No* | 105 | 0.52 | 98 | 0.48 | 93 | 0.46 | 110 | 0.54 | 0.1275 | 0.789 | (0.533-1.169) | 0.2363 | 0.725 | (0.452-1.163) | 0.1809 |
| *Yes* | 22 | 0.63 | 13 | 0.37 | 11 | 0.31 | 24 | 0.69 | 0.0023 | 0.271 | (0.096-0.767) | 0.0155 | 0.064 | (0.011-0.376) | 0.0033 |
| *Chronic pulmonary disease* |  |  |  |  |  |  |  |  |  |  |  |  |  |  |  |
| *No* | 4 | 0.36 | 7 | 0.64 | 4 | 0.36 | 7 | 0.64 | 1.0000 | 1.000 | (0.126-7.936) | 1.0000 | 1.000 | (0.030-33.514) | 1.0000 |
| *Yes* | 123 | 0.54 | 104 | 0.46 | 100 | 0.44 | 127 | 0.56 | 0.0071 | 0.666 | (0.459-0.966) | 0.0326 | 0.574 | (0.367-0.898) | 0.0152 |
| *Mild liver disease* |  |  |  |  |  |  |  |  |  |  |  |  |  |  |  |
| *No* | 88 | 0.55 | 73 | 0.45 | 72 | 0.45 | 89 | 0.55 | 0.0159 | 0.671 | (0.431-1.046) | 0.0778 | 0.556 | (0.320-0.964) | 0.0368 |
| *Yes* | 39 | 0.51 | 38 | 0.49 | 32 | 0.42 | 45 | 0.58 | 0.2087 | 0.693 | (0.361-1.328) | 0.2651 | 0.612 | (0.265-1.413) | 0.2459 |
| *Diabetes with chronic complications* |  |  |  |  |  |  |  |  |  |  |  |  |  |  |  |
| *No* | 113 | 0.58 | 82 | 0.42 | 94 | 0.48 | 101 | 0.52 | 0.0150 | 0.675 | (0.451-1.011) | 0.0564 | 0.585 | (0.362-0.946) | 0.0289 |
| *Yes* | 14 | 0.33 | 29 | 0.67 | 10 | 0.23 | 33 | 0.77 | 0.2850 | 0.628 | (0.233-1.693) | 0.3491 | 0.501 | (0.135-1.859) | 0.2936 |
| *Any malignancy, including lymphoma and leukemia* |  |  |  |  |  |  |  |  |  |  |  |  |  |  |  |
| *No* | 112 | 0.56 | 88 | 0.44 | 94 | 0.47 | 106 | 0.53 | 0.0181 | 0.697 | (0.468-1.037) | 0.0744 | 0.610 | (0.380-0.981) | 0.0417 |
| *Yes* | 15 | 0.39 | 23 | 0.61 | 10 | 0.26 | 28 | 0.74 | 0.2253 | 0.548 | (0.198-1.516) | 0.2384 | 0.402 | (0.108-1.495) | 0.1680 |
| **Index year** |  |  |  |  |  |  |  |  |  |  |  |  |  |  |  |
| *2017* | 66 | 0.49 | 68 | 0.51 | 54 | 0.40 | 80 | 0.60 | 0.0704 | 0.695 | (0.426-1.135) | 0.1449 | 0.618 | (0.344-1.110) | 0.1063 |
| *2018* | 61 | 0.59 | 43 | 0.41 | 50 | 0.48 | 54 | 0.52 | 0.0482 | 0.653 | (0.374-1.139) | 0.1319 | 0.516 | (0.235-1.133) | 0.0983 |

6.2.1.2 Severe exacerbation

| **SEVERE COPD EXACERBATION** |  | | | | | | | | | | | | | | |
| --- | --- | --- | --- | --- | --- | --- | --- | --- | --- | --- | --- | --- | --- | --- | --- |
|  | **Pre-BV** | | | | **Post-BV** | | | | **Mcnemar p-value** | **Crude OR** | | | **Adjusted OR** | | |
|  | **Event** | | **No Event** | | **Event** | | **No Event** | |  |  |  |  |  |  |  |
|  | n | % | n | % | n | % | n | % |  | OR | 95% CI | p-value | OR | 95% CI | p-value |
| **Overall** | 91 | 0.38 | 147 | 0.62 | 81 | 0.34 | 157 | 0.66 | 0.2450 | 0.833 | (0.572-1.215) | 0.3422 | 0.818 | (0.545-1.230) | 0.3330 |
| **COPD medication during pre and post-BV period** |  |  |  |  |  |  |  |  |  |  |  |  |  |  |  |
| *ICS/LABA* | 42 | 0.37 | 71 | 0.63 | 35 | 0.31 | 78 | 0.69 | 0.2623 | 0.759 | (0.433-1.328) | 0.3306 | 0.730 | (0.389-1.368) | 0.3222 |
| *LAMA* | 4 | 0.17 | 20 | 0.83 | 5 | 0.21 | 19 | 0.79 | 0.7055 | 1.316 | (0.273-6.330) | 0.7211 | NA | NA | NA |
| *Triple* | 31 | 0.46 | 36 | 0.54 | 25 | 0.37 | 42 | 0.63 | 0.1797 | 0.691 | (0.341-1.403) | 0.3015 | 0.625 | (0.239-1.633) | 0.3322 |
| *LABA/LAMA* | 14 | 0.41 | 20 | 0.59 | 16 | 0.47 | 18 | 0.53 | 0.4795 | 1.270 | (0.462-3.489) | 0.6337 | 1.406 | (0.399-4.948) | 0.5857 |
| **Age at index date 1** |  |  |  |  |  |  |  |  |  |  |  |  |  |  |  |
| *40 to < 65* | 22 | 0.33 | 45 | 0.67 | 20 | 0.30 | 47 | 0.70 | 0.6374 | 0.870 | (0.411-1.842) | 0.7129 | 0.832 | (0.318-2.177) | 0.7033 |
| *65 to < 75* | 39 | 0.36 | 70 | 0.64 | 39 | 0.36 | 70 | 0.64 | 1.0000 | 1.000 | (0.570-1.755) | 1.0000 | 1.000 | (0.538-1.858) | 1.0000 |
| *75+* | 30 | 0.48 | 32 | 0.52 | 22 | 0.35 | 40 | 0.65 | 0.0881 | 0.587 | (0.280-1.231) | 0.1551 | 0.500 | (0.203-1.229) | 0.1282 |
| **Sex** |  |  |  |  |  |  |  |  |  |  |  |  |  |  |  |
| *Male* | 74 | 0.39 | 116 | 0.61 | 68 | 0.36 | 122 | 0.64 | 0.4142 | 0.874 | (0.574-1.329) | 0.5266 | 0.858 | (0.542-1.360) | 0.5134 |
| *Female* | 17 | 0.35 | 31 | 0.65 | 13 | 0.27 | 35 | 0.73 | 0.3711 | 0.677 | (0.275-1.668) | 0.3889 | 0.597 | (0.185-1.924) | 0.3796 |
| **Insurance type at index date2** |  |  |  |  |  |  |  |  |  |  |  |  |  |  |  |
| *Health insurance* | 79 | 0.38 | 128 | 0.62 | 69 | 0.33 | 138 | 0.67 | 0.2184 | 0.810 | (0.540-1.216) | 0.3078 | 0.790 | (0.508-1.229) | 0.2938 |
| *Medical aid* | 12 | 0.39 | 19 | 0.61 | 12 | 0.39 | 19 | 0.61 | 1.0000 | 1.000 | (0.339-2.952) | 1.0000 | 1.000 | (0.313-3.197) | 1.0000 |
| **Hospital type at index date2** |  |  |  |  |  |  |  |  |  |  |  |  |  |  |  |
| *Tertiary and general hospital* | 81 | 0.41 | 115 | 0.59 | 72 | 0.37 | 124 | 0.63 | 0.2568 | 0.824 | (0.547-1.242) | 0.3540 | 0.810 | (0.520-1.261) | 0.3482 |
| *Others* | 10 | 0.24 | 32 | 0.76 | 9 | 0.21 | 33 | 0.79 | 0.7630 | 0.873 | (0.300-2.538) | 0.7980 | 0.827 | (0.206-3.309) | 0.7830 |
| **History of COPD exacerbation** |  |  |  |  |  |  |  |  |  |  |  |  |  |  |  |
| *none* | 51 | 0.39 | 80 | 0.61 | 37 | 0.28 | 94 | 0.72 | 0.0308 | 0.617 | (0.365-1.043) | 0.0713 | 0.580 | (0.323-1.041) | 0.0677 |
| *1 moderate* | 27 | 0.32 | 58 | 0.68 | 32 | 0.38 | 53 | 0.62 | 0.2971 | 1.297 | (0.680-2.475) | 0.4259 | 1.372 | (0.641-2.936) | 0.4103 |
| *≥ 2 moderate OR ≥ 1 severe* | 13 | 0.59 | 9 | 0.41 | 12 | 0.55 | 10 | 0.45 | 0.7389 | 0.831 | (0.227-3.040) | 0.7692 | 0.727 | (0.106-4.974) | 0.7339 |
| **History of Pneumonia** |  |  |  |  |  |  |  |  |  |  |  |  |  |  |  |
| *No* | 57 | 0.33 | 118 | 0.67 | 52 | 0.30 | 123 | 0.70 | 0.4658 | 0.875 | (0.554-1.383) | 0.5658 | 0.866 | (0.529-1.418) | 0.5655 |
| *Yes* | 34 | 0.54 | 29 | 0.46 | 29 | 0.46 | 34 | 0.54 | 0.3359 | 0.728 | (0.354-1.495) | 0.3807 | 0.693 | (0.304-1.577) | 0.3758 |
| **History of Asthma** |  |  |  |  |  |  |  |  |  |  |  |  |  |  |  |
| *No* | 10 | 0.26 | 28 | 0.74 | 8 | 0.21 | 30 | 0.79 | 0.5930 | 0.747 | (0.245-2.274) | 0.5982 | 0.681 | (0.196-2.360) | 0.5346 |
| *Yes* | 81 | 0.41 | 119 | 0.60 | 73 | 0.37 | 127 | 0.64 | 0.3017 | 0.844 | (0.562-1.268) | 0.4134 | 0.830 | (0.536-1.285) | 0.4010 |
| **mCCI** |  |  |  |  |  |  |  |  |  |  |  |  |  |  |  |
| *0, 1* | 38 | 0.33 | 78 | 0.67 | 39 | 0.34 | 77 | 0.66 | 0.8618 | 1.040 | (0.597-1.811) | 0.8898 | 1.045 | (0.566-1.930) | 0.8876 |
| *2 +* | 53 | 0.43 | 69 | 0.57 | 42 | 0.34 | 80 | 0.66 | 0.0858 | 0.683 | (0.404-1.155) | 0.1536 | 0.657 | (0.369-1.168) | 0.1506 |
| **mCCI category** |  |  |  |  |  |  |  |  |  |  |  |  |  |  |  |
| *Congestive heart failure* |  |  |  |  |  |  |  |  |  |  |  |  |  |  |  |
| *No* | 76 | 0.37 | 127 | 0.63 | 69 | 0.34 | 134 | 0.66 | 0.3853 | 0.860 | (0.571-1.296) | 0.4705 | 0.845 | (0.538-1.327) | 0.4628 |
| *Yes* | 15 | 0.43 | 20 | 0.57 | 12 | 0.34 | 23 | 0.66 | 0.3173 | 0.696 | (0.251-1.924) | 0.4735 | 0.626 | (0.168-2.329) | 0.4737 |
| *Chronic pulmonary disease* |  |  |  |  |  |  |  |  |  |  |  |  |  |  |  |
| *No* | 3 | 0.27 | 8 | 0.73 | 2 | 0.18 | 9 | 0.82 | 0.6547 | 0.593 | (0.053-6.642) | 0.6399 | 0.527 | (0.021-13.527) | 0.6127 |
| *Yes* | 88 | 0.39 | 139 | 0.61 | 79 | 0.35 | 148 | 0.65 | 0.2786 | 0.843 | (0.574-1.239) | 0.3832 | 0.829 | (0.548-1.256) | 0.3748 |
| *Mild liver disease* |  |  |  |  |  |  |  |  |  |  |  |  |  |  |  |
| *No* | 59 | 0.37 | 102 | 0.63 | 52 | 0.32 | 109 | 0.68 | 0.3270 | 0.825 | (0.518-1.313) | 0.4147 | 0.807 | (0.485-1.344) | 0.4086 |
| *Yes* | 32 | 0.42 | 45 | 0.58 | 29 | 0.38 | 48 | 0.62 | 0.5316 | 0.850 | (0.439-1.646) | 0.6249 | 0.819 | (0.375-1.786) | 0.6113 |
| *Diabetes with chronic complications* |  |  |  |  |  |  |  |  |  |  |  |  |  |  |  |
| *No* | 73 | 0.37 | 122 | 0.63 | 69 | 0.35 | 126 | 0.65 | 0.5930 | 0.915 | (0.604-1.388) | 0.6751 | 0.905 | (0.573-1.428) | 0.6654 |
| *Yes* | 18 | 0.42 | 25 | 0.58 | 12 | 0.28 | 31 | 0.72 | 0.1573 | 0.538 | (0.210-1.374) | 0.1892 | 0.443 | (0.131-1.504) | 0.1860 |
| *Any malignancy, including lymphoma and leukemia* |  |  |  |  |  |  |  |  |  |  |  |  |  |  |  |
| *No* | 77 | 0.39 | 123 | 0.62 | 67 | 0.34 | 133 | 0.67 | 0.2113 | 0.805 | (0.533-1.216) | 0.3003 | 0.785 | (0.501-1.230) | 0.2893 |
| *Yes* | 14 | 0.37 | 24 | 0.63 | 14 | 0.37 | 24 | 0.63 | 1.0000 | 1.000 | (0.377-2.655) | 1.0000 | 1.000 | (0.286-3.494) | 1.0000 |
| **Index year** |  |  |  |  |  |  |  |  |  |  |  |  |  |  |  |
| *2017* | 44 | 0.33 | 90 | 0.67 | 43 | 0.32 | 91 | 0.68 | 0.8815 | 0.967 | (0.576-1.623) | 0.8968 | 0.962 | (0.539-1.716) | 0.8941 |
| *2018* | 47 | 0.45 | 57 | 0.55 | 38 | 0.37 | 66 | 0.63 | 0.0947 | 0.698 | (0.397-1.228) | 0.2100 | 0.639 | (0.333-1.226) | 0.1757 |

6.2.1.3 Moderate-to-severe exacerbation

| **MODERATE TO SEVERE COPD EXACERBATION** |  | | | | | | | | | | | | | | |
| --- | --- | --- | --- | --- | --- | --- | --- | --- | --- | --- | --- | --- | --- | --- | --- |
|  | **Pre-BV** | | | | **Post-BV** | | | | **Mcnemar p-value** | **Crude OR** | | | **Adjusted OR** | | |
|  | **Event** | | **No Event** | | **Event** | | **No Event** | |  |  |  |  |  |  |  |
|  | n | % | n | % | n | % | n | % |  | OR | 95% CI | p-value | OR | 95% CI | p-value |
| **Overall** | 165 | 0.69 | 73 | 0.31 | 141 | 0.59 | 97 | 0.41 | 0.0059 | 0.643 | (0.440-0.941) | 0.0232 | 0.571 | (0.368-0.886) | 0.0126 |
| **COPD medication during pre and post-BV period** |  |  |  |  |  |  |  |  |  |  |  |  |  |  |  |
| *ICS/LABA* | 70 | 0.62 | 43 | 0.38 | 57 | 0.50 | 56 | 0.50 | 0.0474 | 0.625 | (0.365-1.070) | 0.0862 | 0.563 | (0.299-1.059) | 0.0743 |
| *LAMA* | 13 | 0.54 | 11 | 0.46 | 11 | 0.46 | 13 | 0.54 | 0.4795 | 0.716 | (0.210-2.436) | 0.5779 | NA | NA | NA |
| *Triple* | 57 | 0.85 | 10 | 0.15 | 50 | 0.75 | 17 | 0.25 | 0.0707 | 0.516 | (0.212-1.258) | 0.1431 | 0.280 | (0.082-0.952) | 0.0417 |
| *LABA/LAMA* | 25 | 0.74 | 9 | 0.26 | 23 | 0.68 | 11 | 0.32 | 0.5271 | 0.753 | (0.250-2.269) | 0.6040 | NA | NA | NA |
| **Age at index date 1** |  |  |  |  |  |  |  |  |  |  |  |  |  |  |  |
| *40 to < 65* | 48 | 0.72 | 19 | 0.28 | 43 | 0.64 | 24 | 0.36 | 0.2513 | 0.709 | (0.336-1.499) | 0.3626 | 1.000 | (0.567-1.764) | 1.0000 |
| *65 to < 75* | 69 | 0.63 | 40 | 0.37 | 66 | 0.61 | 43 | 0.39 | 0.6121 | 0.890 | (0.510-1.551) | 0.6779 | 0.862 | (0.445-1.667) | 0.6556 |
| *75+* | 48 | 0.77 | 14 | 0.23 | 32 | 0.52 | 30 | 0.48 | 0.0006 | 0.311 | (0.140-0.691) | 0.0048 | 0.187 | (0.064-0.547) | 0.0027 |
| **Sex** |  |  |  |  |  |  |  |  |  |  |  |  |  |  |  |
| *Male* | 132 | 0.69 | 58 | 0.31 | 117 | 0.62 | 73 | 0.38 | 0.0431 | 0.704 | (0.459-1.082) | 0.1086 | 0.641 | (0.390-1.052) | 0.0783 |
| *Female* | 33 | 0.69 | 15 | 0.31 | 24 | 0.50 | 24 | 0.50 | 0.0495 | 0.455 | (0.192-1.078) | 0.0725 | 0.320 | (0.106-0.969) | 0.0441 |
| **Insurance type at index date2** |  |  |  |  |  |  |  |  |  |  |  |  |  |  |  |
| *Health insurance* | 140 | 0.68 | 67 | 0.32 | 121 | 0.58 | 86 | 0.42 | 0.0222 | 0.673 | (0.449-1.009) | 0.0555 | 0.608 | (0.381-0.970) | 0.0371 |
| *Medical aid* | 25 | 0.81 | 6 | 0.19 | 20 | 0.65 | 11 | 0.35 | 0.0588 | 0.436 | (0.128-1.484) | 0.1766 | 1.000 | (0.419-2.385) | 1.0000 |
| **Hospital type at index date2** |  |  |  |  |  |  |  |  |  |  |  |  |  |  |  |
| *Tertiary and general hospital* | 136 | 0.69 | 60 | 0.31 | 117 | 0.60 | 79 | 0.40 | 0.0167 | 0.653 | (0.429-0.995) | 0.0473 | 0.577 | (0.354-0.941) | 0.0277 |
| *Others* | 29 | 0.69 | 13 | 0.31 | 24 | 0.57 | 18 | 0.43 | 0.1655 | 0.598 | (0.235-1.520) | 0.2721 | 0.455 | (0.125-1.661) | 0.2264 |
| **History of COPD exacerbation** |  |  |  |  |  |  |  |  |  |  |  |  |  |  |  |
| *none* | 74 | 0.56 | 57 | 0.44 | 58 | 0.44 | 73 | 0.56 | 0.0183 | 0.612 | (0.373-1.003) | 0.0516 | 0.560 | (0.320-0.981) | 0.0429 |
| *1 moderate* | 70 | 0.82 | 15 | 0.18 | 62 | 0.73 | 23 | 0.27 | 0.1306 | 0.578 | (0.273-1.223) | 0.1493 | 0.531 | (0.229-1.230) | 0.1380 |
| *≥ 2 moderate OR ≥ 1 severe* | 21 | 0.95 | 1 | 0.05 | 21 | 0.95 | 1 | 0.05 | 1.0000 | 1.000 | (0.046-21.782) | 1.0000 | 1.000 | (0.202-4.957) | 1.0000 |
| **History of Pneumonia** |  |  |  |  |  |  |  |  |  |  |  |  |  |  |  |
| *No* | 111 | 0.63 | 64 | 0.37 | 92 | 0.53 | 83 | 0.47 | 0.0118 | 0.639 | (0.415-0.984) | 0.0421 | 0.576 | (0.354-0.937) | 0.0265 |
| *Yes* | 54 | 0.86 | 9 | 0.14 | 49 | 0.78 | 14 | 0.22 | 0.2513 | 0.583 | (0.226-1.506) | 0.2602 | 0.530 | (0.184-1.532) | 0.2366 |
| **History of Asthma** |  |  |  |  |  |  |  |  |  |  |  |  |  |  |  |
| *No* | 22 | 0.58 | 16 | 0.42 | 18 | 0.47 | 20 | 0.53 | 0.3458 | 0.655 | (0.253-1.690) | 0.3713 | 0.571 | (0.171-1.907) | 0.3528 |
| *Yes* | 143 | 0.72 | 57 | 0.29 | 123 | 0.62 | 77 | 0.39 | 0.0086 | 0.637 | (0.417-0.971) | 0.0363 | 0.556 | (0.341-0.905) | 0.0186 |
| **mCCI** |  |  |  |  |  |  |  |  |  |  |  |  |  |  |  |
| *0, 1* | 83 | 0.72 | 33 | 0.28 | 79 | 0.68 | 37 | 0.32 | 0.4927 | 0.849 | (0.480-1.501) | 0.5702 | 0.802 | (0.415-1.550) | 0.5079 |
| *2 +* | 82 | 0.67 | 40 | 0.33 | 62 | 0.51 | 60 | 0.49 | 0.0020 | 0.504 | (0.298-0.853) | 0.0111 | 0.431 | (0.234-0.795) | 0.0074 |
| **mCCI category** |  |  |  |  |  |  |  |  |  |  |  |  |  |  |  |
| *Congestive heart failure* |  |  |  |  |  |  |  |  |  |  |  |  |  |  |  |
| *No* | 137 | 0.67 | 66 | 0.33 | 121 | 0.60 | 82 | 0.40 | 0.0489 | 0.711 | (0.472-1.071) | 0.1019 | 0.642 | (0.395-1.044) | 0.0741 |
| *Yes* | 28 | 0.80 | 7 | 0.20 | 20 | 0.57 | 15 | 0.43 | 0.0114 | 0.333 | (0.109-1.022) | 0.0544 | 0.272 | (0.077-0.958) | 0.0430 |
| *Chronic pulmonary disease* |  |  |  |  |  |  |  |  |  |  |  |  |  |  |  |
| *No* | 6 | 0.55 | 5 | 0.45 | 6 | 0.55 | 5 | 0.45 | 1.0000 | 1.000 | (0.135-7.398) | 1.0000 | 1.000 | (0.030-33.514) | 1.0000 |
| *Yes* | 159 | 0.70 | 68 | 0.30 | 135 | 0.59 | 92 | 0.41 | 0.0047 | 0.628 | (0.424-0.928) | 0.0198 | 0.553 | (0.352-0.867) | 0.0101 |
| *Mild liver disease* |  |  |  |  |  |  |  |  |  |  |  |  |  |  |  |
| *No* | 112 | 0.70 | 49 | 0.30 | 98 | 0.61 | 63 | 0.39 | 0.0568 | 0.681 | (0.427-1.085) | 0.1051 | 0.618 | (0.371-1.031) | 0.0649 |
| *Yes* | 53 | 0.69 | 24 | 0.31 | 43 | 0.56 | 34 | 0.44 | 0.0330 | 0.573 | (0.292-1.124) | 0.1039 | 0.497 | (0.217-1.141) | 0.0981 |
| *Diabetes with chronic complications* |  |  |  |  |  |  |  |  |  |  |  |  |  |  |  |
| *No* | 141 | 0.72 | 54 | 0.28 | 125 | 0.64 | 70 | 0.36 | 0.0356 | 0.684 | (0.444-1.054) | 0.0849 | 0.605 | (0.357-1.024) | 0.0614 |
| *Yes* | 24 | 0.56 | 19 | 0.44 | 16 | 0.37 | 27 | 0.63 | 0.0593 | 0.469 | (0.191-1.153) | 0.0967 | 0.375 | (0.122-1.152) | 0.0850 |
| *Any malignancy, including lymphoma and leukemia* |  |  |  |  |  |  |  |  |  |  |  |  |  |  |  |
| *No* | 143 | 0.72 | 57 | 0.29 | 123 | 0.62 | 77 | 0.39 | 0.0098 | 0.637 | (0.417-0.971) | 0.0363 | 0.555 | (0.336-0.917) | 0.0218 |
| *Yes* | 22 | 0.58 | 16 | 0.42 | 18 | 0.47 | 20 | 0.53 | 0.3173 | 0.655 | (0.253-1.690) | 0.3713 | 0.558 | (0.178-1.748) | 0.3073 |
| **Index year** |  |  |  |  |  |  |  |  |  |  |  |  |  |  |  |
| *2017* | 85 | 0.63 | 49 | 0.37 | 77 | 0.57 | 57 | 0.43 | 0.2482 | 0.779 | (0.474-1.280) | 0.3216 | 0.728 | (0.401-1.320) | 0.2933 |
| *2018* | 80 | 0.77 | 24 | 0.23 | 64 | 0.62 | 40 | 0.38 | 0.0025 | 0.480 | (0.260-0.887) | 0.0195 | 0.373 | (0.175-0.796) | 0.0113 |

6.2.2. Frequency of all COPD exacerbation

6.2.2.1 Moderate Exacerbation

| **MODERATE COPD EXACERBATION** | **All** | **Pre-BV** | **Post-BV** | **Relative frequency** | **p-value** |
| --- | --- | --- | --- | --- | --- |
|  |  |  |  |  |  |
| ***Number of event per  person per year*** | 1.43 | 1.60 | 1.26 | 0.78 | 0.1246 |
| *Total event* | 680 | 381 | 299 |  |  |
| *Patients with event* | 231 | 127 | 104 |  |  |
| *observational period (year)* |  |  |  |  |  |
| *Mean* | 365.04 | 365.08 | 365.00 |  |  |
| *SD* | 0.20 | 0.28 | 0.00 |  |  |
| *Median* | 365 | 365 | 365 |  |  |
| *Min* | 365 | 365 | 365 |  |  |
| *Max* | 366 | 366 | 365 |  |  |
| *P25* | 365 | 365 | 365 |  |  |
| *P75* | 365 | 365 | 365 |  |  |
| **COPD medication during pre and post-BV period** |  |  |  |  |  |
| *ICS/LABA* | 1.03 | 1.08 | 0.97 | 0.90 | 0.7047 |
| *LAMA* | 1.06 | 1.38 | 0.75 | 0.55 | 0.2750 |
| *Triple* | 2.26 | 2.46 | 2.06 | 0.84 | 0.4327 |
| *LABA/LAMA* | 1.38 | 1.80 | 0.97 | 0.54 | 0.1591 |
| **Age at index date 1** |  |  |  |  |  |
| *40 to < 65* | 2.09 | 2.43 | 1.75 | 0.72 | 0.2334 |
| *65 to < 75* | 1.30 | 1.25 | 1.35 | 1.08 | 0.7066 |
| *75+* | 0.94 | 1.32 | 0.56 | 0.43 | 0.0262 |
| **Sex** |  |  |  |  |  |
| *Male* | 1.30 | 1.46 | 1.14 | 0.78 | 0.1724 |
| *Female* | 1.94 | 2.17 | 1.71 | 0.79 | 0.4661 |
| **Insurance type at index date2** |  |  |  |  |  |
| *Health insurance* | 1.35 | 1.50 | 1.21 | 0.81 | 0.2091 |
| *Medical aid* | 1.94 | 2.29 | 1.58 | 0.69 | 0.3646 |
| **Hospital type at index date2** |  |  |  |  |  |
| *Tertiary and general hospital* | 1.32 | 1.47 | 1.17 | 0.80 | 0.1667 |
| *Others* | 1.93 | 2.21 | 1.64 | 0.74 | 0.4697 |
| **History of COPD exacerbation** |  |  |  |  |  |
| *none* | 0.60 | 0.58 | 0.62 | 1.07 | 0.8073 |
| *1 moderate* | 2.09 | 2.53 | 1.65 | 0.65 | 0.0374 |
| *≥ 2 moderate OR ≥ 1 severe* | 3.82 | 4.09 | 3.55 | 0.87 | 0.6453 |
| **History of Pneumonia** |  |  |  |  |  |
| *no* | 1.17 | 1.35 | 0.99 | 0.73 | 0.1443 |
| *yes* | 2.15 | 2.30 | 2.00 | 0.87 | 0.5313 |
| **History of Asthma** |  |  |  |  |  |
| *no* | 0.80 | 0.90 | 0.71 | 0.79 | 0.5662 |
| *yes* | 1.55 | 1.74 | 1.36 | 0.78 | 0.1470 |
| **mCCI** |  |  |  |  |  |
| *0, 1* | 1.97 | 2.10 | 1.83 | 0.87 | 0.4850 |
| *2 +* | 0.92 | 1.12 | 0.71 | 0.64 | 0.0471 |
| **mCCI category** |  |  |  |  |  |
| *Congestive heart failure* |  |  |  |  |  |
| *No* | 1.49 | 1.62 | 1.35 | 0.83 | 0.2860 |
| *Yes* | 1.10 | 1.49 | 0.71 | 0.48 | 0.0541 |
| *Dementia* |  |  |  |  |  |
| *No* | 1.44 | 1.61 | 1.27 | 0.79 | 0.1377 |
| *Yes* | 0.92 | 1.17 | 0.67 | 0.57 | 0.4887 |
| *Chronic pulmonary disease* |  |  |  |  |  |
| *No* | 0.50 | 0.45 | 0.55 | 1.20 | 0.7834 |
| *Yes* | 1.47 | 1.66 | 1.29 | 0.78 | 0.1182 |
| *Rheumatologic disease* |  |  |  |  |  |
| *No* | 1.46 | 1.65 | 1.26 | 0.76 | 0.0953 |
| *Yes* | 0.92 | 0.67 | 1.17 | 1.75 | 0.4707 |
| *Mild liver disease* |  |  |  |  |  |
| *No* | 1.61 | 1.80 | 1.43 | 0.80 | 0.2251 |
| *Yes* | 1.05 | 1.20 | 0.90 | 0.75 | 0.2854 |
| *Diabetes with chronic complications* |  |  |  |  |  |
| *No* | 1.63 | 1.81 | 1.44 | 0.80 | 0.1611 |
| *Yes* | 0.54 | 0.65 | 0.42 | 0.64 | 0.3854 |
| *Hemiplegia or paraplegia* |  |  |  |  |  |
| *No* | 1.44 | 1.62 | 1.26 | 0.77 | 0.1071 |
| *Yes* | 0.67 | NA | 1.33 | NA | NA |
| *Renal disease* |  |  |  |  |  |
| *No* | 1.46 | 1.64 | 1.28 | 0.78 | 0.1162 |
| *Yes* | 0.77 | 0.73 | 0.82 | 1.12 | 0.8944 |
| *Any malignancy, including lymphoma and leukemia* |  |  |  |  |  |
| *No* | 1.57 | 1.73 | 1.40 | 0.81 | 0.1988 |
| *Yes* | 0.71 | 0.92 | 0.50 | 0.54 | 0.2280 |
| *Moderate or severe liver disease* |  |  |  |  |  |
| *No* | 1.43 | 1.60 | 1.26 | 0.79 | 0.1338 |
| *Yes* | 1.00 | 2.00 | NA | NA | NA |
| *Metastatic solid tumor* |  |  |  |  |  |
| *No* | 1.43 | 1.61 | 1.26 | 0.78 | 0.1200 |
| *Yes* | 0.50 | NA | 1.00 | NA | NA |
| *HIV* |  |  |  |  |  |
| *No* | 1.43 | 1.60 | 1.26 | 0.78 | 0.1246 |
| *Yes* | NA | NA | NA | NA | NA |
| **Index year** |  |  |  |  |  |
| *2017* | 1.40 | 1.66 | 1.15 | 0.69 | 0.1014 |
| *2018* | 1.46 | 1.53 | 1.40 | 0.91 | 0.6776 |

6.2.2.2 Severe exacerbation

| **SEVERE COPD EXACERBATION** | **All** | **Pre-BV** | **Post-BV** | **Relative frequency** | **p-value** |
| --- | --- | --- | --- | --- | --- |
|  |  |  |  |  |  |
| ***Number of event per  person per year*** | 0.65 | 0.72 | 0.58 | 0.81 | 0.2366 |
| *Total event* | 310 | 171 | 139 |  |  |
| *Patients with event* | 172 | 91 | 81 |  |  |
| *observational period (year)* |  |  |  |  |  |
| *Mean* | 365.04 | 365.08 | 365.00 |  |  |
| *SD* | 0.20 | 0.28 | 0.00 |  |  |
| *Median* | 365 | 365 | 365 |  |  |
| *Min* | 365 | 365 | 365 |  |  |
| *Max* | 366 | 366 | 365 |  |  |
| *P25* | 365 | 365 | 365 |  |  |
| *P75* | 365 | 365 | 365 |  |  |
| **COPD medication during pre and post-BV period** |  |  |  |  |  |
| *ICS/LABA* | 0.57 | 0.66 | 0.47 | 0.71 | 0.1794 |
| *LAMA* | 0.23 | 0.25 | 0.21 | 0.83 | 0.7974 |
| *Triple* | 0.75 | 0.79 | 0.72 | 0.91 | 0.7395 |
| *LABA/LAMA* | 1.03 | 1.09 | 0.97 | 0.89 | 0.7853 |
| **Age at index date 1** |  |  |  |  |  |
| *40 to < 65* | 0.69 | 0.82 | 0.55 | 0.67 | 0.3329 |
| *65 to < 75* | 0.55 | 0.56 | 0.54 | 0.97 | 0.8823 |
| *75+* | 0.79 | 0.89 | 0.69 | 0.78 | 0.4011 |
| **Sex** |  |  |  |  |  |
| *Male* | 0.65 | 0.70 | 0.61 | 0.88 | 0.4976 |
| *Female* | 0.65 | 0.81 | 0.48 | 0.59 | 0.2412 |
| **Insurance type at index date2** |  |  |  |  |  |
| *Health insurance* | 0.63 | 0.71 | 0.56 | 0.78 | 0.1820 |
| *Medical aid* | 0.77 | 0.77 | 0.77 | 1.00 | 1.0000 |
| **Hospital type at index date2** |  |  |  |  |  |
| *Tertiary and general hospital* | 0.70 | 0.75 | 0.65 | 0.86 | 0.4151 |
| *Others* | 0.43 | 0.57 | 0.29 | 0.50 | 0.2692 |
| **History of COPD exacerbation** |  |  |  |  |  |
| *none* | 0.49 | 0.55 | 0.44 | 0.79 | 0.2710 |
| *1 moderate* | 0.55 | 0.54 | 0.57 | 1.04 | 0.8647 |
| *≥ 2 moderate OR ≥ 1 severe* | 1.98 | 2.41 | 1.55 | 0.64 | 0.3072 |
| **History of Pneumonia** |  |  |  |  |  |
| *no* | 0.45 | 0.46 | 0.44 | 0.96 | 0.8377 |
| *yes* | 1.22 | 1.45 | 0.98 | 0.68 | 0.1815 |
| **History of Asthma** |  |  |  |  |  |
| *no* | 0.34 | 0.39 | 0.29 | 0.73 | 0.5223 |
| *yes* | 0.71 | 0.78 | 0.64 | 0.82 | 0.2849 |
| **mCCI** |  |  |  |  |  |
| *0, 1* | 0.63 | 0.66 | 0.60 | 0.90 | 0.6843 |
| *2 +* | 0.67 | 0.77 | 0.57 | 0.74 | 0.2001 |
| **mCCI category** |  |  |  |  |  |
| *Congestive heart failure* |  |  |  |  |  |
| *No* | 0.62 | 0.67 | 0.57 | 0.85 | 0.3834 |
| *Yes* | 0.84 | 1.00 | 0.69 | 0.69 | 0.3787 |
| *Dementia* |  |  |  |  |  |
| *No* | 0.66 | 0.73 | 0.58 | 0.79 | 0.1814 |
| *Yes* | 0.50 | 0.17 | 0.83 | 5.00 | 0.1698 |
| *Chronic pulmonary disease* |  |  |  |  |  |
| *No* | 0.27 | 0.27 | 0.27 | 1.00 | 1.0000 |
| *Yes* | 0.67 | 0.74 | 0.60 | 0.81 | 0.2334 |
| *Rheumatologic disease* |  |  |  |  |  |
| *No* | 0.66 | 0.74 | 0.59 | 0.80 | 0.2049 |
| *Yes* | 0.42 | 0.33 | 0.50 | 1.50 | 0.5427 |
| *Mild liver disease* |  |  |  |  |  |
| *No* | 0.60 | 0.65 | 0.55 | 0.86 | 0.4753 |
| *Yes* | 0.76 | 0.87 | 0.65 | 0.75 | 0.3200 |
| *Diabetes with chronic complications* |  |  |  |  |  |
| *No* | 0.69 | 0.75 | 0.62 | 0.83 | 0.3340 |
| *Yes* | 0.50 | 0.58 | 0.42 | 0.72 | 0.3771 |
| *Hemiplegia or paraplegia* |  |  |  |  |  |
| *No* | 0.64 | 0.71 | 0.57 | 0.80 | 0.2184 |
| *Yes* | 1.50 | 1.33 | 1.67 | 1.25 | 0.7961 |
| *Renal disease* |  |  |  |  |  |
| *No* | 0.66 | 0.72 | 0.59 | 0.82 | 0.2607 |
| *Yes* | 0.55 | 0.64 | 0.45 | 0.71 | 0.6765 |
| *Any malignancy, including lymphoma and leukemia* |  |  |  |  |  |
| *No* | 0.67 | 0.74 | 0.60 | 0.81 | 0.2810 |
| *Yes* | 0.55 | 0.61 | 0.50 | 0.83 | 0.6003 |
| *Moderate or severe liver disease* |  |  |  |  |  |
| *No* | 0.65 | 0.72 | 0.59 | 0.81 | 0.2363 |
| *Yes* | NA | NA | NA | NA | NA |
| *Metastatic solid tumor* |  |  |  |  |  |
| *No* | 0.65 | 0.72 | 0.59 | 0.81 | 0.2363 |
| *Yes* | NA | NA | NA | NA | NA |
| *HIV* |  |  |  |  |  |
| *No* | 0.65 | 0.72 | 0.58 | 0.81 | 0.2366 |
| *Yes* | NA | NA | NA | NA | NA |
| **Index year** |  |  |  |  |  |
| *2017* | 0.55 | 0.56 | 0.53 | 0.95 | 0.8210 |
| *2018* | 0.79 | 0.92 | 0.65 | 0.71 | 0.1704 |

6.2.2.3 Moderate-to-severe exacerbation

| **MODERATE TO SEVERE COPD EXACERBATION** | **All** | **Pre-BV** | **Post-BV** | **Relative frequency** | **p-value** |
| --- | --- | --- | --- | --- | --- |
|  |  |  |  |  |  |
| ***Number of event per  person per year*** | 2.08 | 2.32 | 1.84 | 0.79 | 0.0575 |
| *Total event* | 990 | 552 | 438 |  |  |
| *Patients with event* | 306 | 165 | 141 |  |  |
| *observational period (year)* |  |  |  |  |  |
| *Mean* | 365.04 | 365.08 | 365.00 |  |  |
| *SD* | 0.20 | 0.28 | 0.00 |  |  |
| *Median* | 365 | 365 | 365 |  |  |
| *Min* | 365 | 365 | 365 |  |  |
| *Max* | 366 | 366 | 365 |  |  |
| *P25* | 365 | 365 | 365 |  |  |
| *P75* | 365 | 365 | 365 |  |  |
| **COPD medication during pre and post-BV period** |  |  |  |  |  |
| *ICS/LABA* | 1.59 | 1.74 | 1.44 | 0.83 | 0.3410 |
| *LAMA* | 1.29 | 1.63 | 0.96 | 0.59 | 0.2685 |
| *Triple* | 3.02 | 3.25 | 2.78 | 0.85 | 0.3862 |
| *LABA/LAMA* | 2.41 | 2.88 | 1.94 | 0.67 | 0.1932 |
| **Age at index date 1** |  |  |  |  |  |
| *40 to < 65* | 2.78 | 3.26 | 2.30 | 0.71 | 0.1401 |
| *65 to < 75* | 1.85 | 1.81 | 1.89 | 1.05 | 0.7800 |
| *75+* | 1.73 | 2.21 | 1.26 | 0.57 | 0.0194 |
| **Sex** |  |  |  |  |  |
| *Male* | 1.95 | 2.15 | 1.75 | 0.81 | 0.1241 |
| *Female* | 2.58 | 2.98 | 2.19 | 0.73 | 0.2668 |
| **Insurance type at index date2** |  |  |  |  |  |
| *Health insurance* | 1.99 | 2.21 | 1.76 | 0.80 | 0.0843 |
| *Medical aid* | 2.71 | 3.07 | 2.36 | 0.77 | 0.4277 |
| **Hospital type at index date2** |  |  |  |  |  |
| *Tertiary and general hospital* | 2.02 | 2.22 | 1.82 | 0.82 | 0.1145 |
| *Others* | 2.36 | 2.79 | 1.93 | 0.69 | 0.2962 |
| **History of COPD exacerbation** |  |  |  |  |  |
| *none* | 1.09 | 1.13 | 1.05 | 0.93 | 0.6927 |
| *1 moderate* | 2.64 | 3.07 | 2.21 | 0.72 | 0.0460 |
| *≥ 2 moderate OR ≥ 1 severe* | 5.80 | 6.50 | 5.09 | 0.78 | 0.2636 |
| **History of Pneumonia** |  |  |  |  |  |
| *no* | 1.62 | 1.81 | 1.43 | 0.79 | 0.1446 |
| *yes* | 3.37 | 3.75 | 2.99 | 0.80 | 0.1831 |
| **History of Asthma** |  |  |  |  |  |
| *no* | 1.15 | 1.29 | 1.00 | 0.78 | 0.4120 |
| *yes* | 2.26 | 2.52 | 2.00 | 0.80 | 0.0753 |
| **mCCI** |  |  |  |  |  |
| *0, 1* | 2.60 | 2.77 | 2.42 | 0.88 | 0.4127 |
| *2 +* | 1.59 | 1.89 | 1.29 | 0.68 | 0.0276 |
| **mCCI category** |  |  |  |  |  |
| *Congestive heart failure* |  |  |  |  |  |
| *No* | 2.10 | 2.29 | 1.92 | 0.84 | 0.1835 |
| *Yes* | 1.94 | 2.49 | 1.40 | 0.56 | 0.0453 |
| *Dementia* |  |  |  |  |  |
| *No* | 2.10 | 2.35 | 1.85 | 0.79 | 0.0548 |
| *Yes* | 1.42 | 1.33 | 1.50 | 1.12 | 0.8119 |
| *Chronic pulmonary disease* |  |  |  |  |  |
| *No* | 0.77 | 0.73 | 0.82 | 1.12 | 0.8160 |
| *Yes* | 2.14 | 2.40 | 1.89 | 0.79 | 0.0536 |
| *Rheumatologic disease* |  |  |  |  |  |
| *No* | 2.12 | 2.39 | 1.85 | 0.77 | 0.0397 |
| *Yes* | 1.33 | 1.00 | 1.67 | 1.67 | 0.3639 |
| *Mild liver disease* |  |  |  |  |  |
| *No* | 2.21 | 2.44 | 1.98 | 0.81 | 0.1572 |
| *Yes* | 1.81 | 2.07 | 1.55 | 0.75 | 0.1747 |
| *Diabetes with chronic complications* |  |  |  |  |  |
| *No* | 2.31 | 2.56 | 2.06 | 0.81 | 0.0897 |
| *Yes* | 1.04 | 1.23 | 0.84 | 0.68 | 0.2663 |
| *Hemiplegia or paraplegia* |  |  |  |  |  |
| *No* | 2.08 | 2.33 | 1.83 | 0.78 | 0.0470 |
| *Yes* | 2.17 | 1.33 | 3.00 | 2.25 | 0.3688 |
| *Renal disease* |  |  |  |  |  |
| *No* | 2.12 | 2.37 | 1.87 | 0.79 | 0.0562 |
| *Yes* | 1.32 | 1.36 | 1.27 | 0.93 | 0.9192 |
| *Any malignancy, including lymphoma and leukemia* |  |  |  |  |  |
| *No* | 2.24 | 2.47 | 2.00 | 0.81 | 0.0996 |
| *Yes* | 1.26 | 1.53 | 1.00 | 0.66 | 0.2444 |
| *Moderate or severe liver disease* |  |  |  |  |  |
| *No* | 2.09 | 2.32 | 1.85 | 0.80 | 0.0618 |
| *Yes* | 1.00 | 2.00 | NA | NA | NA |
| *Metastatic solid tumor* |  |  |  |  |  |
| *No* | 2.09 | 2.33 | 1.85 | 0.79 | 0.0552 |
| *Yes* | 0.50 | NA | 1.00 | NA | NA |
| *HIV* |  |  |  |  |  |
| *No* | 2.08 | 2.32 | 1.84 | 0.79 | 0.0575 |
| *Yes* | NA | NA | NA | NA | NA |
| **Index year** |  |  |  |  |  |
| *2017* | 1.95 | 2.22 | 1.68 | 0.76 | 0.1151 |
| *2018* | 2.25 | 2.45 | 2.05 | 0.84 | 0.2812 |

6.2.3 Incidence rate of all COPD exacerbation

6.2.3.1 Moderate exacerbation

| **MODERATE COPD EXACERBATION** | **All** | **Pre-BV** | **Post-BV** | **p-value** | **IRR** | **IRR-pvalue** |
| --- | --- | --- | --- | --- | --- | --- |
|  |  |  |  |  |  |  |
| ***Incidence rate per 1,000 PYs*** | 700.39 | 804.75 | 604.64 | 0.0301 | 0.75 | 0.0306 |
| *Patients with event* | 231 | 127 | 104 |  |  |  |
| *Sum of person years (PYs)* | 329.82 | 157.81 | 172.00 |  |  |  |
| *Time to event (KM estimated)* |  |  |  |  |  |  |
| *Median* | NA | 338.50 | NA |  |  |  |
| *SE* | NA | 9.36 | NA |  |  |  |
| *Time to event (descriptive)* |  |  |  |  |  |  |
| *Mean* | 134.82 | 135.66 | 133.79 |  |  |  |
| *SD* | 114.31 | 119.94 | 107.61 |  |  |  |
| *Median* | 91.00 | 91.00 | 91.00 |  |  |  |
| *Min* | 1.00 | 1.00 | 4.00 |  |  |  |
| *Max* | 365.00 | 365.00 | 362.00 |  |  |  |
| *P25* | 33.00 | 31.00 | 34.00 |  |  |  |
| *P75* | 234.00 | 252.00 | 216.00 |  |  |  |
| **COPD medication during pre and post-BV period** |  |  |  |  |  |  |
| *ICS/LABA* | 511.60 | 541.13 | 482.76 | 0.5925 | 0.89 | 0.5928 |
| *LAMA* | 597.37 | 810.28 | 418.63 | 0.1348 | 0.52 | 0.1417 |
| *Triple* | 1190.00 | 1367.44 | 1029.67 | 0.1840 | 0.75 | 0.1851 |
| *LABA/LAMA* | 698.10 | 923.40 | 517.67 | 0.0934 | 0.56 | 0.0968 |
| **Age at index date 1** |  |  |  |  |  |  |
| *40 to < 65* | 926.69 | 1002.29 | 853.39 | 0.4808 | 0.85 | 0.4813 |
| *65 to < 75* | 673.01 | 705.81 | 641.42 | 0.6274 | 0.91 | 0.6275 |
| *75+* | 544.40 | 789.19 | 347.05 | 0.0040 | 0.44 | 0.0051 |
| **Sex** |  |  |  |  |  |  |
| *Male* | 698.89 | 782.17 | 620.39 | 0.1146 | 0.79 | 0.1154 |
| *Female* | 706.69 | 906.41 | 542.97 | 0.0870 | 0.60 | 0.0895 |
| **Insurance type at index date2** |  |  |  |  |  |  |
| *Health insurance* | 657.03 | 738.50 | 580.60 | 0.0952 | 0.79 | 0.0960 |
| *Medical aid* | 1053.55 | 1407.27 | 782.96 | 0.0719 | 0.56 | 0.0743 |
| **Hospital type at index date2** |  |  |  |  |  |  |
| *Tertiary and general hospital* | 678.17 | 768.68 | 594.93 | 0.0810 | 0.77 | 0.0817 |
| *Others* | 810.15 | 984.20 | 652.29 | 0.1698 | 0.66 | 0.1729 |
| **History of COPD exacerbation** |  |  |  |  |  |  |
| *none* | 364.41 | 396.20 | 333.32 | 0.4402 | 0.84 | 0.4409 |
| *1 moderate* | 1186.80 | 1549.08 | 901.34 | 0.0041 | 0.58 | 0.0044 |
| *≥ 2 moderate OR ≥ 1 severe* | 2522.17 | 2595.27 | 2453.08 | 0.8621 | 0.95 | 0.8621 |
| **History of Pneumonia** |  |  |  |  |  |  |
| *no* | 559.74 | 668.50 | 460.94 | 0.0253 | 0.69 | 0.0261 |
| *yes* | 1232.24 | 1307.44 | 1160.60 | 0.5830 | 0.89 | 0.5831 |
| **History of Asthma** |  |  |  |  |  |  |
| *no* | 517.35 | 604.31 | 435.42 | 0.3712 | 0.72 | 0.3737 |
| *yes* | 739.44 | 848.24 | 640.19 | 0.0464 | 0.75 | 0.0471 |
| **mCCI** |  |  |  |  |  |  |
| *0, 1* | 909.98 | 969.59 | 852.49 | 0.4633 | 0.88 | 0.4636 |
| *2 +* | 540.24 | 672.90 | 423.07 | 0.0204 | 0.63 | 0.0215 |
| **mCCI category** |  |  |  |  |  |  |
| *Congestive heart failure* |  |  |  |  |  |  |
| *No* | 705.89 | 765.43 | 648.91 | 0.2456 | 0.85 | 0.2461 |
| *Yes* | 669.11 | 1066.14 | 383.48 | 0.0041 | 0.36 | 0.0056 |
| *Dementia* |  |  |  |  |  |  |
| *No* | 694.91 | 794.09 | 603.17 | 0.0400 | 0.76 | 0.0406 |
| *Yes* | 936.88 | 1370.54 | 658.90 | 0.3345 | 0.48 | 0.3376 |
| *Chronic pulmonary disease* |  |  |  |  |  |  |
| *No* | 424.09 | 406.74 | 443.00 | 0.9039 | 1.09 | 0.9039 |
| *Yes* | 717.15 | 831.20 | 613.60 | 0.0237 | 0.74 | 0.0242 |
| *Rheumatologic disease* |  |  |  |  |  |  |
| *No* | 715.41 | 831.17 | 609.88 | 0.0214 | 0.73 | 0.0219 |
| *Yes* | 461.43 | 406.96 | 516.77 | 0.7209 | 1.27 | 0.7218 |
| *Mild liver disease* |  |  |  |  |  |  |
| *No* | 746.42 | 867.04 | 637.95 | 0.0527 | 0.74 | 0.0535 |
| *Yes* | 614.94 | 692.50 | 541.09 | 0.2996 | 0.78 | 0.3009 |
| *Diabetes with chronic complications* |  |  |  |  |  |  |
| *No* | 804.87 | 921.20 | 698.79 | 0.0472 | 0.76 | 0.0478 |
| *Yes* | 330.44 | 398.34 | 266.78 | 0.3291 | 0.67 | 0.3329 |
| *Hemiplegia or paraplegia* |  |  |  |  |  |  |
| *No* | 700.38 | 820.29 | 591.64 | 0.0139 | 0.72 | 0.0142 |
| *Yes* | 701.06 | NA | 2326.43 | NA | NA | NA |
| *Renal disease* |  |  |  |  |  |  |
| *No* | 719.05 | 828.06 | 619.70 | 0.0304 | 0.75 | 0.0309 |
| *Yes* | 382.63 | 431.36 | 332.55 | 0.7322 | 0.77 | 0.7334 |
| *Any malignancy, including lymphoma and leukemia* |  |  |  |  |  |  |
| *No* | 776.17 | 876.46 | 683.05 | 0.0740 | 0.78 | 0.0747 |
| *Yes* | 388.13 | 499.57 | 290.83 | 0.1798 | 0.58 | 0.1851 |
| *Moderate or severe liver disease* |  |  |  |  |  |  |
| *No* | 699.89 | 799.40 | 608.18 | 0.0385 | 0.76 | 0.0391 |
| *Yes* | 837.73 | 5144.37 | NA | NA | NA | NA |
| *Metastatic solid tumor* |  |  |  |  |  |  |
| *No* | 700.75 | 809.87 | 600.92 | 0.0239 | 0.74 | 0.0244 |
| *Yes* | 626.50 | NA | 1667.81 | NA | NA | NA |
| *HIV* |  |  |  |  |  |  |
| *No* | 700.39 | 804.75 | 604.64 | 0.0301 | 0.75 | 0.0306 |
| *Yes* | NA | NA | NA | NA | NA | NA |
| **Index year** |  |  |  |  |  |  |
| *2017* | 630.69 | 719.94 | 547.71 | 0.1350 | 0.76 | 0.1362 |
| *2018* | 795.42 | 922.31 | 681.11 | 0.1109 | 0.74 | 0.1120 |

6.2.3.2 Severe exacerbation

| **SEVERE COPD EXACERBATION** | **All** | **Pre-BV** | **Post-BV** | **p-value** | **IRR** | **IRR-pvalue** |
| --- | --- | --- | --- | --- | --- | --- |
|  |  |  |  |  |  |  |
| ***Incidence rate per 1,000 PYs*** | 448.53 | 473.19 | 423.73 | 0.4695 | 0.90 | 0.4699 |
| *Patients with event* | 172 | 91 | 81 |  |  |  |
| *Sum of person years (PYs)* | 383.47 | 192.31 | 191.16 |  |  |  |
| *Time to event (KM estimated)* |  |  |  |  |  |  |
| *Median* | NA | NA | NA |  |  |  |
| *SE* | NA | NA | NA |  |  |  |
| *Time to event (descriptive)* |  |  |  |  |  |  |
| *Mean* | 169.96 | 183.70 | 154.52 |  |  |  |
| *SD* | 111.61 | 118.54 | 101.80 |  |  |  |
| *Median* | 156.50 | 157.00 | 150.00 |  |  |  |
| *Min* | 2.00 | 6.00 | 2.00 |  |  |  |
| *Max* | 364.00 | 364.00 | 357.00 |  |  |  |
| *P25* | 68.00 | 72.00 | 64.00 |  |  |  |
| *P75* | 268.00 | 298.00 | 238.00 |  |  |  |
| **COPD medication during pre and post-BV period** |  |  |  |  |  |  |
| *ICS/LABA* | 424.01 | 471.58 | 378.23 | 0.3340 | 0.80 | 0.3351 |
| *LAMA* | 209.57 | 182.99 | 237.11 | 0.6983 | 1.30 | 0.6993 |
| *Triple* | 524.01 | 570.07 | 476.28 | 0.5026 | 0.84 | 0.5037 |
| *LABA/LAMA* | 576.29 | 518.30 | 638.83 | 0.5672 | 1.23 | 0.5678 |
| **Age at index date 1** |  |  |  |  |  |  |
| *40 to < 65* | 381.56 | 399.66 | 363.45 | 0.7584 | 0.91 | 0.7586 |
| *65 to < 75* | 432.20 | 430.82 | 433.59 | 0.9774 | 1.01 | 0.9774 |
| *75+* | 559.59 | 641.84 | 476.35 | 0.2853 | 0.74 | 0.2881 |
| **Sex** |  |  |  |  |  |  |
| *Male* | 464.79 | 479.40 | 449.87 | 0.7050 | 0.94 | 0.7051 |
| *Female* | 384.83 | 447.93 | 324.96 | 0.3813 | 0.73 | 0.3837 |
| **Insurance type at index date2** |  |  |  |  |  |  |
| *Health insurance* | 442.92 | 474.98 | 411.15 | 0.3805 | 0.87 | 0.3811 |
| *Medical aid* | 486.54 | 461.71 | 514.20 | 0.7920 | 1.11 | 0.7920 |
| **Hospital type at index date2** |  |  |  |  |  |  |
| *Tertiary and general hospital* | 491.88 | 518.15 | 465.34 | 0.5066 | 0.90 | 0.5069 |
| *Others* | 262.35 | 277.88 | 247.01 | 0.7976 | 0.89 | 0.7977 |
| **History of COPD exacerbation** |  |  |  |  |  |  |
| *none* | 401.97 | 476.57 | 330.63 | 0.0882 | 0.69 | 0.0904 |
| *1 moderate* | 430.56 | 377.83 | 488.04 | 0.3263 | 1.29 | 0.3274 |
| *≥ 2 moderate OR ≥ 1 severe* | 908.40 | 939.50 | 876.95 | 0.8633 | 0.93 | 0.8633 |
| **History of Pneumonia** |  |  |  |  |  |  |
| *no* | 366.90 | 379.87 | 353.67 | 0.7094 | 0.93 | 0.7095 |
| *yes* | 729.25 | 804.57 | 657.13 | 0.4224 | 0.82 | 0.4232 |
| **History of Asthma** |  |  |  |  |  |  |
| *no* | 262.94 | 297.85 | 229.34 | 0.5803 | 0.77 | 0.5816 |
| *yes* | 488.87 | 510.28 | 467.12 | 0.5838 | 0.92 | 0.5840 |
| **mCCI** |  |  |  |  |  |  |
| *0, 1* | 403.57 | 390.32 | 417.37 | 0.7688 | 1.07 | 0.7688 |
| *2 +* | 493.06 | 558.15 | 429.81 | 0.2043 | 0.77 | 0.2060 |
| **mCCI category** |  |  |  |  |  |  |
| *Congestive heart failure* |  |  |  |  |  |  |
| *No* | 438.53 | 453.55 | 423.11 | 0.6759 | 0.93 | 0.6761 |
| *Yes* | 511.13 | 606.19 | 427.36 | 0.3649 | 0.70 | 0.3667 |
| *Dementia* |  |  |  |  |  |  |
| *No* | 444.73 | 480.56 | 409.09 | 0.2989 | 0.85 | 0.2996 |
| *Yes* | 627.79 | 198.83 | 1362.87 | 0.0482 | 6.85 | 0.0851 |
| *Chronic pulmonary disease* |  |  |  |  |  |  |
| *No* | 240.11 | 293.61 | 188.57 | 0.6239 | 0.64 | 0.6276 |
| *Yes* | 460.50 | 483.27 | 437.55 | 0.5211 | 0.91 | 0.5214 |
| *Rheumatologic disease* |  |  |  |  |  |  |
| *No* | 452.17 | 478.90 | 425.34 | 0.4480 | 0.89 | 0.4484 |
| *Yes* | 385.08 | 375.68 | 394.97 | 0.9435 | 1.05 | 0.9435 |
| *Mild liver disease* |  |  |  |  |  |  |
| *No* | 421.16 | 447.03 | 395.22 | 0.5168 | 0.88 | 0.5173 |
| *Yes* | 508.69 | 530.43 | 486.69 | 0.7370 | 0.92 | 0.7371 |
| *Diabetes with chronic complications* |  |  |  |  |  |  |
| *No* | 454.98 | 463.76 | 446.05 | 0.8166 | 0.96 | 0.8166 |
| *Yes* | 420.34 | 515.73 | 329.05 | 0.2228 | 0.64 | 0.2279 |
| *Hemiplegia or paraplegia* |  |  |  |  |  |  |
| *No* | 439.68 | 468.24 | 411.06 | 0.4006 | 0.88 | 0.4011 |
| *Yes* | 1371.06 | 893.03 | 2131.81 | 0.3340 | 2.39 | 0.3405 |
| *Renal disease* |  |  |  |  |  |  |
| *No* | 451.91 | 475.59 | 428.14 | 0.4999 | 0.90 | 0.5002 |
| *Yes* | 381.32 | 426.32 | 334.27 | 0.7490 | 0.78 | 0.7501 |
| *Any malignancy, including lymphoma and leukemia* |  |  |  |  |  |  |
| *No* | 448.88 | 478.54 | 419.02 | 0.4261 | 0.88 | 0.4266 |
| *Yes* | 446.79 | 445.78 | 447.81 | 0.9904 | 1.00 | 0.9904 |
| *Moderate or severe liver disease* |  |  |  |  |  |  |
| *No* | 450.88 | 475.65 | 425.96 | 0.4697 | 0.90 | 0.4700 |
| *Yes* | NA | NA | NA | NA | NA | NA |
| *Metastatic solid tumor* |  |  |  |  |  |  |
| *No* | 450.88 | 475.65 | 425.96 | 0.4697 | 0.90 | 0.4700 |
| *Yes* | NA | NA | NA | NA | NA | NA |
| *HIV* |  |  |  |  |  |  |
| *No* | 448.53 | 473.19 | 423.73 | 0.4695 | 0.90 | 0.4699 |
| *Yes* | NA | NA | NA | NA | NA | NA |
| **Index year** |  |  |  |  |  |  |
| *2017* | 391.97 | 395.02 | 388.90 | 0.9420 | 0.98 | 0.9420 |
| *2018* | 526.26 | 580.78 | 471.51 | 0.3380 | 0.81 | 0.3394 |

6.2.3.3 Moderate-to-severe exacerbation

| **MODERATE TO SEVERE COPD EXACERBATION** | **All** | **Pre-BV** | **Post-BV** | **p-value** | **IRR** | **IRR-pvalue** |
| --- | --- | --- | --- | --- | --- | --- |
|  |  |  |  |  |  |  |
| ***Incidence rate per 1,000 PYs*** | 1099.20 | 1247.18 | 965.18 | 0.0251 | 0.77 | 0.0254 |
| *Patients with event* | 306 | 165 | 141 |  |  |  |
| *Sum of person years (PYs)* | 278.38 | 132.30 | 146.09 |  |  |  |
| *Time to event (KM estimated)* |  |  |  |  |  |  |
| *Median* | 239.00 | 196.00 | 258.50 |  |  |  |
| *SE* | 6.61 | 9.44 | 9.17 |  |  |  |
| *Time to event (descriptive)* |  |  |  |  |  |  |
| *Mean* | 129.73 | 131.78 | 127.33 |  |  |  |
| *SD* | 111.96 | 117.36 | 105.65 |  |  |  |
| *Median* | 91.00 | 91.00 | 91.00 |  |  |  |
| *Min* | 1.00 | 1.00 | 2.00 |  |  |  |
| *Max* | 365.00 | 365.00 | 362.00 |  |  |  |
| *P25* | 32.00 | 33.00 | 32.00 |  |  |  |
| *P75* | 219.00 | 222.00 | 209.00 |  |  |  |
| **COPD medication during pre and post-BV period** |  |  |  |  |  |  |
| *ICS/LABA* | 878.47 | 1020.13 | 750.49 | 0.0843 | 0.74 | 0.0853 |
| *LAMA* | 723.27 | 816.13 | 637.54 | 0.5458 | 0.78 | 0.5466 |
| *Triple* | 1730.97 | 1872.40 | 1593.73 | 0.4050 | 0.85 | 0.4056 |
| *LABA/LAMA* | 1236.56 | 1444.36 | 1069.34 | 0.2980 | 0.74 | 0.2981 |
| **Age at index date 1** |  |  |  |  |  |  |
| *40 to < 65* | 1247.24 | 1356.86 | 1144.07 | 0.4161 | 0.84 | 0.4166 |
| *65 to < 75* | 1011.03 | 1052.86 | 970.71 | 0.6370 | 0.92 | 0.6370 |
| *75+* | 1112.72 | 1529.31 | 789.94 | 0.0034 | 0.52 | 0.0038 |
| **Sex** |  |  |  |  |  |  |
| *Male* | 1114.85 | 1218.27 | 1017.41 | 0.1554 | 0.84 | 0.1559 |
| *Female* | 1035.68 | 1377.99 | 772.00 | 0.0294 | 0.56 | 0.0308 |
| **Insurance type at index date2** |  |  |  |  |  |  |
| *Health insurance* | 1054.91 | 1188.25 | 933.69 | 0.0517 | 0.79 | 0.0521 |
| *Medical aid* | 1452.99 | 1726.79 | 1212.65 | 0.2369 | 0.70 | 0.2387 |
| **Hospital type at index date2** |  |  |  |  |  |  |
| *Tertiary and general hospital* | 1102.86 | 1251.11 | 969.34 | 0.0426 | 0.77 | 0.0430 |
| *Others* | 1082.07 | 1229.08 | 945.43 | 0.3404 | 0.77 | 0.3417 |
| **History of COPD exacerbation** |  |  |  |  |  |  |
| *none* | 699.41 | 813.13 | 593.50 | 0.0714 | 0.73 | 0.0726 |
| *1 moderate* | 1660.06 | 1888.57 | 1460.53 | 0.1401 | 0.77 | 0.1405 |
| *≥ 2 moderate OR ≥ 1 severe* | 4142.72 | 4967.78 | 3552.69 | 0.2784 | 0.72 | 0.2773 |
| **History of Pneumonia** |  |  |  |  |  |  |
| *no* | 892.22 | 1022.95 | 773.02 | 0.0463 | 0.76 | 0.0469 |
| *yes* | 2025.13 | 2269.94 | 1810.00 | 0.2508 | 0.80 | 0.2511 |
| **History of Asthma** |  |  |  |  |  |  |
| *no* | 746.06 | 864.68 | 638.92 | 0.3396 | 0.74 | 0.3411 |
| *yes* | 1183.44 | 1338.26 | 1043.14 | 0.0424 | 0.78 | 0.0428 |
| **mCCI** |  |  |  |  |  |  |
| *0, 1* | 1316.45 | 1348.87 | 1284.03 | 0.7539 | 0.95 | 0.7540 |
| *2 +* | 927.08 | 1158.76 | 733.20 | 0.0062 | 0.63 | 0.0065 |
| **mCCI category** |  |  |  |  |  |  |
| *Congestive heart failure* |  |  |  |  |  |  |
| *No* | 1071.52 | 1162.60 | 984.22 | 0.1813 | 0.85 | 0.1818 |
| *Yes* | 1276.45 | 1936.57 | 864.09 | 0.0054 | 0.45 | 0.0058 |
| *Dementia* |  |  |  |  |  |  |
| *No* | 1075.36 | 1227.65 | 937.52 | 0.0208 | 0.76 | 0.0210 |
| *Yes* | 2711.03 | 2539.99 | 2872.22 | 0.8389 | 1.13 | 0.8391 |
| *Chronic pulmonary disease* |  |  |  |  |  |  |
| *No* | 670.59 | 648.57 | 694.17 | 0.9063 | 1.07 | 0.9063 |
| *Yes* | 1128.64 | 1292.19 | 982.23 | 0.0188 | 0.76 | 0.0191 |
| *Rheumatologic disease* |  |  |  |  |  |  |
| *No* | 1117.76 | 1277.92 | 973.85 | 0.0204 | 0.76 | 0.0207 |
| *Yes* | 816.46 | 808.33 | 824.76 | 0.9700 | 1.02 | 0.9700 |
| *Mild liver disease* |  |  |  |  |  |  |
| *No* | 1146.20 | 1286.90 | 1018.89 | 0.0909 | 0.79 | 0.0914 |
| *Yes* | 1008.72 | 1170.82 | 861.68 | 0.1339 | 0.74 | 0.1352 |
| *Diabetes with chronic complications* |  |  |  |  |  |  |
| *No* | 1237.84 | 1363.81 | 1121.03 | 0.1101 | 0.82 | 0.1106 |
| *Yes* | 629.99 | 830.11 | 462.67 | 0.0666 | 0.56 | 0.0701 |
| *Hemiplegia or paraplegia* |  |  |  |  |  |  |
| *No* | 1094.52 | 1253.28 | 952.07 | 0.0172 | 0.76 | 0.0175 |
| *Yes* | 1479.94 | 893.03 | 2634.01 | 0.2304 | 2.95 | 0.2361 |
| *Renal disease* |  |  |  |  |  |  |
| *No* | 1129.44 | 1278.67 | 994.70 | 0.0309 | 0.78 | 0.0312 |
| *Yes* | 613.25 | 754.65 | 478.70 | 0.4759 | 0.63 | 0.4807 |
| *Any malignancy, including lymphoma and leukemia* |  |  |  |  |  |  |
| *No* | 1193.06 | 1345.01 | 1054.55 | 0.0475 | 0.78 | 0.0479 |
| *Yes* | 721.66 | 846.82 | 611.24 | 0.3035 | 0.72 | 0.3050 |
| *Moderate or severe liver disease* |  |  |  |  |  |  |
| *No* | 1100.32 | 1241.45 | 971.83 | 0.0327 | 0.78 | 0.0330 |
| *Yes* | 837.73 | 5144.37 | NA | NA | NA | NA |
| *Metastatic solid tumor* |  |  |  |  |  |  |
| *No* | 1101.92 | 1256.65 | 962.29 | 0.0199 | 0.77 | 0.0202 |
| *Yes* | 626.50 | NA | 1667.81 | NA | NA | NA |
| *HIV* |  |  |  |  |  |  |
| *No* | 1099.20 | 1247.18 | 965.18 | 0.0251 | 0.77 | 0.0254 |
| *Yes* | NA | NA | NA | NA | NA | NA |
| **Index year** |  |  |  |  |  |  |
| *2017* | 982.88 | 1055.46 | 913.54 | 0.3583 | 0.87 | 0.3587 |
| *2018* | 1268.02 | 1545.46 | 1035.62 | 0.0165 | 0.67 | 0.0170 |

6.3 Subgroup analysis of antibiotics-used COPD AE

6.3.1. Odd ratio of antibiotics-used COPD AE

6.3.1.1 Moderate exacerbation

| **MODERATE COPD EXACERBATION** |  | | | | | | | | | | | | | | | | |
| --- | --- | --- | --- | --- | --- | --- | --- | --- | --- | --- | --- | --- | --- | --- | --- | --- | --- |
|  | **Pre-BV** | | | | **Post-BV** | | | | **Mcnemar p-value** | **Crude OR** | | | | **Adjusted OR** | | | |
|  | **Event** | | **No Event** | | **Event** | | **No Event** | |  |  |  |  |  |  |  |  |  |
|  | n | % | n | % | n | % | n | % |  | OR | 95% CI | p-value | OR | | 95% CI | p-value |  |
| **Overall** | 83 | 0.35 | 155 | 0.65 | 73 | 0.31 | 165 | 0.69 | 0.2253 | 0.826 | (0.562-1.216) | 0.3311 | 0.788 | | (0.507-1.225) | 0.2886 |  |
| **COPD medication during pre and post-BV period** |  |  |  |  |  |  |  |  |  |  |  |  |  | |  |  |  |
| *ICS/LABA* | 27 | 0.24 | 86 | 0.76 | 26 | 0.23 | 87 | 0.77 | 0.8415 | 0.952 | (0.510-1.778) | 0.8761 | 0.931 | | (0.345-2.510) | 0.8869 |  |
| *LAMA* | 11 | 0.46 | 13 | 0.54 | 8 | 0.33 | 16 | 0.67 | 0.2568 | 0.591 | (0.168-2.082) | 0.3965 | NA | | NA | NA |  |
| *Triple* | 31 | 0.46 | 36 | 0.54 | 30 | 0.45 | 37 | 0.55 | 0.8415 | 0.942 | (0.469-1.892) | 0.8638 | 0.925 | | (0.408-2.098) | 0.8503 |  |
| *LABA/LAMA* | 14 | 0.41 | 20 | 0.59 | 9 | 0.26 | 25 | 0.74 | 0.1317 | 0.514 | (0.175-1.512) | 0.2183 | 0.413 | | (0.109-1.562) | 0.1853 |  |
| **Age at index date 1** |  |  |  |  |  |  |  |  |  |  |  |  |  | |  |  |  |
| *40 to < 65* | 26 | 0.39 | 41 | 0.61 | 25 | 0.37 | 42 | 0.63 | 0.8084 | 0.939 | (0.459-1.921) | 0.8604 | 0.923 | | (0.399-2.138) | 0.8504 |  |
| *65 to < 75* | 34 | 0.31 | 75 | 0.69 | 36 | 0.33 | 73 | 0.67 | 0.6949 | 1.088 | (0.610-1.939) | 0.7733 | 1.124 | | (0.558-2.265) | 0.7414 |  |
| *75+* | 23 | 0.37 | 39 | 0.63 | 12 | 0.19 | 50 | 0.81 | 0.0278 | 0.407 | (0.176-0.940) | 0.0357 | 0.294 | | (0.084-1.024) | 0.0545 |  |
| **Sex** |  |  |  |  |  |  |  |  |  |  |  |  |  | |  |  |  |
| *Male* | 68 | 0.36 | 122 | 0.64 | 60 | 0.32 | 130 | 0.68 | 0.2850 | 0.828 | (0.539-1.273) | 0.3878 | 0.793 | | (0.484-1.300) | 0.3555 |  |
| *Female* | 15 | 0.31 | 33 | 0.69 | 13 | 0.27 | 35 | 0.73 | 0.5637 | 0.817 | (0.327-2.040) | 0.6590 | 0.693 | | (0.192-2.505) | 0.5683 |  |
| **Insurance type at index date2** |  |  |  |  |  |  |  |  |  |  |  |  |  | |  |  |  |
| *Health insurance* | 67 | 0.32 | 140 | 0.68 | 63 | 0.30 | 144 | 0.70 | 0.5930 | 0.914 | (0.601-1.390) | 0.6731 | 0.894 | | (0.552-1.450) | 0.6492 |  |
| *Medical aid* | 16 | 0.52 | 15 | 0.48 | 10 | 0.32 | 21 | 0.68 | 0.0833 | 0.446 | (0.150-1.331) | 0.1420 | 0.212 | | (0.036-1.259) | 0.0855 |  |
| **Hospital type at index date2** |  |  |  |  |  |  |  |  |  |  |  |  |  | |  |  |  |
| *Tertiary and general hospital* | 72 | 0.37 | 124 | 0.63 | 62 | 0.32 | 134 | 0.68 | 0.1967 | 0.797 | (0.523-1.215) | 0.2898 | 0.750 | | (0.457-1.230) | 0.2526 |  |
| *Others* | 11 | 0.26 | 31 | 0.74 | 11 | 0.26 | 31 | 0.74 | 1.0000 | 1.000 | (0.363-2.758) | 1.0000 | 1.000 | | (0.305-3.279) | 1.0000 |  |
| **History of COPD exacerbation** |  |  |  |  |  |  |  |  |  |  |  |  |  | |  |  |  |
| *none* | 27 | 0.21 | 104 | 0.79 | 28 | 0.21 | 103 | 0.79 | 0.8474 | 1.047 | (0.573-1.913) | 0.8801 | 1.063 | | (0.386-2.930) | 0.9051 |  |
| *1 moderate* | 42 | 0.49 | 43 | 0.51 | 31 | 0.36 | 54 | 0.64 | 0.0630 | 0.588 | (0.314-1.099) | 0.0949 | 0.527 | | (0.256-1.083) | 0.0805 |  |
| *≥ 2 moderate OR ≥ 1 severe* | 14 | 0.64 | 8 | 0.36 | 14 | 0.64 | 8 | 0.36 | 1.0000 | 1.000 | (0.263-3.797) | 1.0000 | 1.199 | | (0.473-3.041) | 0.8409 |  |
| **History of Pneumonia** |  |  |  |  |  |  |  |  |  |  |  |  |  | |  |  |  |
| *No* | 51 | 0.29 | 124 | 0.71 | 42 | 0.24 | 133 | 0.76 | 0.1699 | 0.768 | (0.475-1.242) | 0.2796 | 0.715 | | (0.396-1.291) | 0.2635 |  |
| *Yes* | 32 | 0.51 | 31 | 0.49 | 31 | 0.49 | 32 | 0.51 | 0.8415 | 0.938 | (0.458-1.924) | 0.8603 | 0.927 | | (0.402-2.140) | 0.8570 |  |
| **History of Asthma** |  |  |  |  |  |  |  |  |  |  |  |  |  | |  |  |  |
| *No* | 12 | 0.32 | 26 | 0.68 | 10 | 0.26 | 28 | 0.74 | 0.5271 | 0.774 | (0.273-2.194) | 0.6210 | NA | | NA | NA |  |
| *Yes* | 71 | 0.36 | 129 | 0.65 | 63 | 0.32 | 137 | 0.69 | 0.2935 | 0.836 | (0.549-1.271) | 0.3991 | 0.795 | | (0.492-1.286) | 0.3484 |  |
| **mCCI** |  |  |  |  |  |  |  |  |  |  |  |  |  | |  |  |  |
| *0, 1* | 46 | 0.40 | 70 | 0.60 | 44 | 0.38 | 72 | 0.62 | 0.7237 | 0.930 | (0.544-1.590) | 0.7890 | 0.911 | | (0.498-1.667) | 0.7604 |  |
| *2 +* | 37 | 0.30 | 85 | 0.70 | 29 | 0.24 | 93 | 0.76 | 0.1824 | 0.716 | (0.403-1.275) | 0.2541 | 0.650 | | (0.312-1.352) | 0.2462 |  |
| **mCCI category** |  |  |  |  |  |  |  |  |  |  |  |  |  | |  |  |  |
| *Congestive heart failure* |  |  |  |  |  |  |  |  |  |  |  |  |  | |  |  |  |
| *No* | 68 | 0.33 | 135 | 0.67 | 64 | 0.32 | 139 | 0.68 | 0.5994 | 0.914 | (0.601-1.390) | 0.6730 | 0.893 | | (0.547-1.459) | 0.6503 |  |
| *Yes* | 15 | 0.43 | 20 | 0.57 | 9 | 0.26 | 26 | 0.74 | 0.0578 | 0.462 | (0.159-1.337) | 0.1489 | 0.311 | | (0.054-1.779) | 0.1823 |  |
| *Chronic pulmonary disease* |  |  |  |  |  |  |  |  |  |  |  |  |  | |  |  |  |
| *No* | 2 | 0.18 | 9 | 0.82 | 3 | 0.27 | 8 | 0.73 | 0.3173 | 1.687 | (0.151-18.914) | 0.6399 | NA | | NA | NA |  |
| *Yes* | 81 | 0.36 | 146 | 0.64 | 70 | 0.31 | 157 | 0.69 | 0.1790 | 0.804 | (0.542-1.192) | 0.2757 | 0.762 | | (0.487-1.192) | 0.2322 |  |
| *Mild liver disease* |  |  |  |  |  |  |  |  |  |  |  |  |  | |  |  |  |
| *No* | 59 | 0.37 | 102 | 0.63 | 51 | 0.32 | 110 | 0.68 | 0.2278 | 0.802 | (0.503-1.278) | 0.3504 | 0.740 | | (0.425-1.288) | 0.2845 |  |
| *Yes* | 24 | 0.31 | 53 | 0.69 | 22 | 0.29 | 55 | 0.71 | 0.6831 | 0.883 | (0.436-1.790) | 0.7275 | 0.849 | | (0.371-1.941) | 0.6942 |  |
| *Diabetes with chronic complications* |  |  |  |  |  |  |  |  |  |  |  |  |  | |  |  |  |
| *No* | 78 | 0.40 | 117 | 0.60 | 69 | 0.35 | 126 | 0.65 | 0.2492 | 0.821 | (0.543-1.242) | 0.3496 | 0.789 | | (0.500-1.246) | 0.3081 |  |
| *Yes* | 5 | 0.12 | 38 | 0.88 | 4 | 0.09 | 39 | 0.91 | 0.7055 | 0.779 | (0.183-3.312) | 0.7299 | 0.656 | | (0.160-2.682) | 0.5486 |  |
| *Any malignancy, including lymphoma and leukemia* |  |  |  |  |  |  |  |  |  |  |  |  |  | |  |  |  |
| *No* | 72 | 0.36 | 128 | 0.64 | 68 | 0.34 | 132 | 0.66 | 0.5862 | 0.916 | (0.605-1.386) | 0.6762 | 0.896 | | (0.565-1.421) | 0.6390 |  |
| *Yes* | 11 | 0.29 | 27 | 0.71 | 5 | 0.13 | 33 | 0.87 | 0.1088 | 0.372 | (0.109-1.271) | 0.1114 | 0.250 | | (0.037-1.717) | 0.1535 |  |
| **Index year** |  |  |  |  |  |  |  |  |  |  |  |  |  | |  |  |  |
| *2017* | 43 | 0.32 | 91 | 0.68 | 38 | 0.28 | 96 | 0.72 | 0.4111 | 0.838 | (0.494-1.422) | 0.5089 | 0.784 | | (0.420-1.464) | 0.4427 |  |
| *2018* | 40 | 0.38 | 64 | 0.62 | 35 | 0.34 | 69 | 0.66 | 0.3692 | 0.812 | (0.456-1.445) | 0.4743 | 0.768 | | (0.390-1.515) | 0.4430 |  |

6.3.1.2 Severe exacerbation

| **SEVERE COPD EXACERBATION** |  | | | | | | | | | | | | | | |
| --- | --- | --- | --- | --- | --- | --- | --- | --- | --- | --- | --- | --- | --- | --- | --- |
|  | **Pre-BV** | | | | **Post-BV** | | | | **Mcnemar p-value** | **Crude OR** | | | **Adjusted OR** | | |
|  | **Event** | | **No Event** | | **Event** | | **No Event** | |  |  |  |  |  |  |  |
|  | n | % | n | % | n | % | n | % |  | OR | 95% CI | p-value | OR | 95% CI | p-value |
| **Overall** | 49 | 0.21 | 189 | 0.79 | 40 | 0.17 | 198 | 0.83 | 0.2492 | 0.779 | (0.489-1.242) | 0.2928 | 0.769 | (0.465-1.273) | 0.3060 |
| **COPD medication during pre and post-BV period** |  |  |  |  |  |  |  |  |  |  |  |  |  |  |  |
| *ICS/LABA* | 21 | 0.19 | 92 | 0.81 | 16 | 0.14 | 97 | 0.86 | 0.3532 | 0.723 | (0.351-1.486) | 0.3740 | 0.709 | (0.317-1.587) | 0.3996 |
| *LAMA* | 4 | 0.17 | 20 | 0.83 | 3 | 0.13 | 21 | 0.88 | 0.6547 | 0.714 | (0.125-4.085) | 0.6935 | 1.000 | (0.408-2.453) | 1.0000 |
| *Triple* | 17 | 0.25 | 50 | 0.75 | 13 | 0.19 | 54 | 0.81 | 0.3711 | 0.708 | (0.306-1.640) | 0.4147 | 0.690 | (0.310-1.537) | 0.3578 |
| *LABA/LAMA* | 7 | 0.21 | 27 | 0.79 | 8 | 0.24 | 26 | 0.76 | 0.7055 | 1.187 | (0.354-3.980) | 0.7752 | 1.339 | (0.366-4.892) | 0.6498 |
| **Age at index date 1** |  |  |  |  |  |  |  |  |  |  |  |  |  |  |  |
| *40 to < 65* | 7 | 0.10 | 60 | 0.90 | 8 | 0.12 | 59 | 0.88 | 0.7630 | 1.162 | (0.385-3.507) | 0.7867 | 1.182 | (0.406-3.443) | 0.7561 |
| *65 to < 75* | 27 | 0.25 | 82 | 0.75 | 20 | 0.18 | 89 | 0.82 | 0.1936 | 0.682 | (0.352-1.323) | 0.2551 | 0.648 | (0.314-1.339) | 0.2392 |
| *75+* | 15 | 0.24 | 47 | 0.76 | 12 | 0.19 | 50 | 0.81 | 0.5127 | 0.752 | (0.311-1.816) | 0.5204 | 0.729 | (0.281-1.888) | 0.5087 |
| **Sex** |  |  |  |  |  |  |  |  |  |  |  |  |  |  |  |
| *Male* | 43 | 0.23 | 147 | 0.77 | 34 | 0.18 | 156 | 0.82 | 0.1985 | 0.745 | (0.448-1.238) | 0.2543 | 0.726 | (0.429-1.229) | 0.2316 |
| *Female* | 6 | 0.13 | 42 | 0.88 | 6 | 0.13 | 42 | 0.88 | 1.0000 | 1.000 | (0.285-3.507) | 1.0000 | 1.000 | (0.304-3.290) | 1.0000 |
| **Insurance type at index date2** |  |  |  |  |  |  |  |  |  |  |  |  |  |  |  |
| *Health insurance* | 45 | 0.22 | 162 | 0.78 | 35 | 0.17 | 172 | 0.83 | 0.1892 | 0.733 | (0.446-1.202) | 0.2167 | 0.720 | (0.425-1.220) | 0.2214 |
| *Medical aid* | 4 | 0.13 | 27 | 0.87 | 5 | 0.16 | 26 | 0.84 | 0.5637 | 1.298 | (0.288-5.846) | 0.7258 | 1.492 | (0.338-6.592) | 0.5863 |
| **Hospital type at index date2** |  |  |  |  |  |  |  |  |  |  |  |  |  |  |  |
| *Tertiary and general hospital* | 45 | 0.23 | 151 | 0.77 | 36 | 0.18 | 160 | 0.82 | 0.2164 | 0.755 | (0.460-1.240) | 0.2650 | 0.742 | (0.441-1.249) | 0.2604 |
| *Others* | 4 | 0.10 | 38 | 0.90 | 4 | 0.10 | 38 | 0.90 | 1.0000 | 1.000 | (0.219-4.570) | 1.0000 | 1.000 | (0.210-4.754) | 1.0000 |
| **History of COPD exacerbation** |  |  |  |  |  |  |  |  |  |  |  |  |  |  |  |
| *none* | 26 | 0.20 | 105 | 0.80 | 22 | 0.17 | 109 | 0.83 | 0.4652 | 0.815 | (0.431-1.540) | 0.5260 | 0.795 | (0.383-1.648) | 0.5347 |
| *1 moderate* | 18 | 0.21 | 67 | 0.79 | 15 | 0.18 | 70 | 0.82 | 0.5485 | 0.798 | (0.366-1.737) | 0.5650 | 0.797 | (0.354-1.795) | 0.5793 |
| *≥ 2 moderate OR ≥ 1 severe* | 5 | 0.23 | 17 | 0.77 | 3 | 0.14 | 19 | 0.86 | 0.4142 | 0.537 | (0.097-2.966) | 0.4575 | 1.000 | (0.406-2.466) | 1.0000 |
| **History of Pneumonia** |  |  |  |  |  |  |  |  |  |  |  |  |  |  |  |
| *No* | 32 | 0.18 | 143 | 0.82 | 29 | 0.17 | 146 | 0.83 | 0.6310 | 0.888 | (0.508-1.551) | 0.6740 | 0.882 | (0.481-1.615) | 0.6814 |
| *Yes* | 17 | 0.27 | 46 | 0.73 | 11 | 0.17 | 52 | 0.83 | 0.2008 | 0.572 | (0.237-1.380) | 0.2097 | 0.524 | (0.193-1.424) | 0.2012 |
| **History of Asthma** |  |  |  |  |  |  |  |  |  |  |  |  |  |  |  |
| *No* | 7 | 0.18 | 31 | 0.82 | 4 | 0.11 | 34 | 0.89 | 0.3173 | 0.521 | (0.131-2.080) | 0.3461 | 0.505 | (0.179-1.425) | 0.1902 |
| *Yes* | 42 | 0.21 | 158 | 0.79 | 36 | 0.18 | 164 | 0.82 | 0.4054 | 0.826 | (0.501-1.362) | 0.4513 | 0.817 | (0.473-1.412) | 0.4679 |
| **mCCI** |  |  |  |  |  |  |  |  |  |  |  |  |  |  |  |
| *0, 1* | 25 | 0.22 | 91 | 0.78 | 19 | 0.16 | 97 | 0.84 | 0.2568 | 0.713 | (0.364-1.395) | 0.3204 | 0.691 | (0.310-1.542) | 0.3634 |
| *2 +* | 24 | 0.20 | 98 | 0.80 | 21 | 0.17 | 101 | 0.83 | 0.6015 | 0.849 | (0.440-1.639) | 0.6230 | 0.843 | (0.415-1.710) | 0.6330 |
| **mCCI category** |  |  |  |  |  |  |  |  |  |  |  |  |  |  |  |
| *Congestive heart failure* |  |  |  |  |  |  |  |  |  |  |  |  |  |  |  |
| *No* | 42 | 0.21 | 161 | 0.79 | 36 | 0.18 | 167 | 0.82 | 0.4054 | 0.826 | (0.502-1.361) | 0.4521 | 0.817 | (0.474-1.408) | 0.4644 |
| *Yes* | 7 | 0.20 | 28 | 0.80 | 4 | 0.11 | 31 | 0.89 | 0.3173 | 0.516 | (0.127-2.092) | 0.3437 | 0.414 | (0.089-1.928) | 0.2520 |
| *Chronic pulmonary disease* |  |  |  |  |  |  |  |  |  |  |  |  |  |  |  |
| *No* | 2 | 0.18 | 9 | 0.82 | 1 | 0.09 | 10 | 0.91 | 0.5637 | 0.450 | (0.021-9.567) | 0.5734 | 0.449 | (0.024-8.349) | 0.4894 |
| *Yes* | 47 | 0.21 | 180 | 0.79 | 39 | 0.17 | 188 | 0.83 | 0.2935 | 0.794 | (0.494-1.277) | 0.3406 | 0.786 | (0.470-1.312) | 0.3546 |
| *Mild liver disease* |  |  |  |  |  |  |  |  |  |  |  |  |  |  |  |
| *No* | 36 | 0.22 | 125 | 0.78 | 24 | 0.15 | 137 | 0.85 | 0.0641 | 0.608 | (0.342-1.083) | 0.0906 | 0.581 | (0.301-1.123) | 0.1056 |
| *Yes* | 13 | 0.17 | 64 | 0.83 | 16 | 0.21 | 61 | 0.79 | 0.4913 | 1.291 | (0.563-2.962) | 0.5414 | 1.318 | (0.529-3.282) | 0.5489 |
| *Diabetes with chronic complications* |  |  |  |  |  |  |  |  |  |  |  |  |  |  |  |
| *No* | 37 | 0.19 | 158 | 0.81 | 34 | 0.17 | 161 | 0.83 | 0.6682 | 0.902 | (0.536-1.516) | 0.6951 | 0.896 | (0.506-1.587) | 0.7054 |
| *Yes* | 12 | 0.28 | 31 | 0.72 | 6 | 0.14 | 37 | 0.86 | 0.0833 | 0.419 | (0.135-1.304) | 0.1296 | 0.265 | (0.077-0.914) | 0.0362 |
| *Any malignancy, including lymphoma and leukemia* |  |  |  |  |  |  |  |  |  |  |  |  |  |  |  |
| *No* | 44 | 0.22 | 156 | 0.78 | 33 | 0.17 | 167 | 0.84 | 0.1235 | 0.701 | (0.423-1.162) | 0.1668 | 0.685 | (0.390-1.206) | 0.1887 |
| *Yes* | 5 | 0.13 | 33 | 0.87 | 7 | 0.18 | 31 | 0.82 | 0.5271 | 1.490 | (0.403-5.509) | 0.5401 | 1.833 | (0.492-6.832) | 0.3567 |
| **Index year** |  |  |  |  |  |  |  |  |  |  |  |  |  |  |  |
| *2017* | 30 | 0.22 | 104 | 0.78 | 24 | 0.18 | 110 | 0.82 | 0.3304 | 0.756 | (0.412-1.389) | 0.3651 | 0.737 | (0.387-1.402) | 0.3493 |
| *2018* | 19 | 0.18 | 85 | 0.82 | 16 | 0.15 | 88 | 0.85 | 0.5316 | 0.813 | (0.388-1.707) | 0.5816 | 0.790 | (0.302-2.064) | 0.6269 |

6.3.2.3. Moderate-to-severe exacerbation

| **MODERATE TO SEVERE COPD EXACERBATION** |  | | | | | | | | | | | | | | | | |
| --- | --- | --- | --- | --- | --- | --- | --- | --- | --- | --- | --- | --- | --- | --- | --- | --- | --- |
|  | **Pre-BV** | | | | **Post-BV** | | | | **Mcnemar p-value** | **Crude OR** | | | | **Adjusted OR** | | | |
|  | **Event** | | **No Event** | | **Event** | | **No Event** | |  |  |  |  |  |  |  |  |  |
|  | n | % | n | % | n | % | n | % |  | OR | 95% CI | p-value | OR | | 95% CI | p-value |  |
| **Overall** | 113 | 0.47 | 125 | 0.53 | 101 | 0.42 | 137 | 0.58 | 0.1904 | 0.816 | (0.567-1.174) | 0.2712 | 0.790 | | (0.530-1.178) | 0.2458 |  |
| **COPD medication during pre and post-BV period** |  |  |  |  |  |  |  |  |  |  |  |  |  | |  |  |  |
| *ICS/LABA* | 41 | 0.36 | 72 | 0.64 | 39 | 0.35 | 74 | 0.65 | 0.7456 | 0.926 | (0.532-1.610) | 0.7823 | 0.905 | | (0.452-1.810) | 0.7753 |  |
| *LAMA* | 13 | 0.54 | 11 | 0.46 | 10 | 0.42 | 14 | 0.58 | 0.2568 | 0.604 | (0.177-2.069) | 0.4061 | 1.000 | | (0.348-2.873) | 1.0000 |  |
| *Triple* | 39 | 0.58 | 28 | 0.42 | 37 | 0.55 | 30 | 0.45 | 0.7055 | 0.885 | (0.439-1.786) | 0.7304 | 0.858 | | (0.374-1.967) | 0.7129 |  |
| *LABA/LAMA* | 20 | 0.59 | 14 | 0.41 | 15 | 0.44 | 19 | 0.56 | 0.1317 | 0.553 | (0.201-1.522) | 0.2422 | 0.417 | | (0.106-1.643) | 0.2034 |  |
| **Age at index date 1** |  |  |  |  |  |  |  |  |  |  |  |  |  | |  |  |  |
| *40 to < 65* | 28 | 0.42 | 39 | 0.58 | 32 | 0.48 | 35 | 0.52 | 0.3458 | 1.273 | (0.632-2.565) | 0.4931 | 1.351 | | (0.538-3.392) | 0.5166 |  |
| *65 to < 75* | 52 | 0.48 | 57 | 0.52 | 49 | 0.45 | 60 | 0.55 | 0.6394 | 0.895 | (0.521-1.538) | 0.6859 | 0.871 | | (0.456-1.664) | 0.6723 |  |
| *75+* | 33 | 0.53 | 29 | 0.47 | 20 | 0.32 | 42 | 0.68 | 0.0093 | 0.418 | (0.198-0.886) | 0.0236 | 0.326 | | (0.131-0.809) | 0.0165 |  |
| **Sex** |  |  |  |  |  |  |  |  |  |  |  |  |  | |  |  |  |
| *Male* | 95 | 0.50 | 95 | 0.50 | 83 | 0.44 | 107 | 0.56 | 0.1456 | 0.776 | (0.516-1.166) | 0.2204 | 0.746 | | (0.476-1.170) | 0.2005 |  |
| *Female* | 18 | 0.38 | 30 | 0.63 | 18 | 0.38 | 30 | 0.63 | 1.0000 | 1.000 | (0.424-2.357) | 1.0000 | 1.000 | | (0.327-3.058) | 0.9993 |  |
| **Insurance type at index date2** |  |  |  |  |  |  |  |  |  |  |  |  |  | |  |  |  |
| *Health insurance* | 94 | 0.45 | 113 | 0.55 | 87 | 0.42 | 120 | 0.58 | 0.4126 | 0.872 | (0.589-1.290) | 0.4899 | 0.854 | | (0.557-1.310) | 0.4683 |  |
| *Medical aid* | 19 | 0.61 | 12 | 0.39 | 14 | 0.45 | 17 | 0.55 | 0.1317 | 0.520 | (0.178-1.518) | 0.2223 | 0.298 | | (0.039-2.248) | 0.2304 |  |
| **Hospital type at index date2** |  |  |  |  |  |  |  |  |  |  |  |  |  | |  |  |  |
| *Tertiary and general hospital* | 99 | 0.51 | 97 | 0.49 | 86 | 0.44 | 110 | 0.56 | 0.1281 | 0.766 | (0.513-1.144) | 0.1914 | 0.733 | | (0.470-1.144) | 0.1704 |  |
| *Others* | 14 | 0.33 | 28 | 0.67 | 15 | 0.36 | 27 | 0.64 | 0.7630 | 1.111 | (0.435-2.840) | 0.8218 | 1.142 | | (0.357-3.652) | 0.8187 |  |
| **History of COPD exacerbation** |  |  |  |  |  |  |  |  |  |  |  |  |  | |  |  |  |
| *none* | 48 | 0.37 | 83 | 0.63 | 45 | 0.34 | 86 | 0.66 | 0.6473 | 0.905 | (0.542-1.511) | 0.7003 | 0.883 | | (0.477-1.634) | 0.6890 |  |
| *1 moderate* | 49 | 0.58 | 36 | 0.42 | 42 | 0.49 | 43 | 0.51 | 0.2367 | 0.718 | (0.387-1.330) | 0.2882 | 0.684 | | (0.342-1.369) | 0.2797 |  |
| *≥ 2 moderate OR ≥ 1 severe* | 16 | 0.73 | 6 | 0.27 | 14 | 0.64 | 8 | 0.36 | 0.4142 | 0.656 | (0.164-2.631) | 0.5350 | 1.000 | | (0.375-2.665) | 1.0000 |  |
| **History of Pneumonia** |  |  |  |  |  |  |  |  |  |  |  |  |  | |  |  |  |
| *No* | 75 | 0.43 | 100 | 0.57 | 65 | 0.37 | 110 | 0.63 | 0.1655 | 0.788 | (0.511-1.215) | 0.2784 | 0.752 | | (0.461-1.227) | 0.2523 |  |
| *Yes* | 38 | 0.60 | 25 | 0.40 | 36 | 0.57 | 27 | 0.43 | 0.7237 | 0.877 | (0.423-1.820) | 0.7208 | 0.862 | | (0.376-1.976) | 0.7223 |  |
| **History of Asthma** |  |  |  |  |  |  |  |  |  |  |  |  |  | |  |  |  |
| *No* | 17 | 0.45 | 21 | 0.55 | 13 | 0.34 | 25 | 0.66 | 0.2850 | 0.642 | (0.243-1.695) | 0.3614 | 0.539 | | (0.151-1.916) | 0.3295 |  |
| *Yes* | 96 | 0.48 | 104 | 0.52 | 88 | 0.44 | 112 | 0.56 | 0.3390 | 0.851 | (0.572-1.266) | 0.4245 | 0.827 | | (0.533-1.284) | 0.3953 |  |
| **mCCI** |  |  |  |  |  |  |  |  |  |  |  |  |  | |  |  |  |
| *0, 1* | 59 | 0.51 | 57 | 0.49 | 58 | 0.50 | 58 | 0.50 | 0.8759 | 0.966 | (0.573-1.629) | 0.8962 | 0.959 | | (0.533-1.727) | 0.8881 |  |
| *2 +* | 54 | 0.44 | 68 | 0.56 | 43 | 0.35 | 79 | 0.65 | 0.0934 | 0.685 | (0.406-1.156) | 0.1551 | 0.644 | | (0.357-1.161) | 0.1415 |  |
| **mCCI category** |  |  |  |  |  |  |  |  |  |  |  |  |  | |  |  |  |
| *Congestive heart failure* |  |  |  |  |  |  |  |  |  |  |  |  |  | |  |  |  |
| *No* | 93 | 0.46 | 110 | 0.54 | 89 | 0.44 | 114 | 0.56 | 0.6374 | 0.923 | (0.622-1.370) | 0.6909 | 0.910 | | (0.586-1.414) | 0.6746 |  |
| *Yes* | 20 | 0.57 | 15 | 0.43 | 12 | 0.34 | 23 | 0.66 | 0.0209 | 0.391 | (0.141-1.082) | 0.0695 | 0.265 | | (0.067-1.051) | 0.0583 |  |
| *Chronic pulmonary disease* |  |  |  |  |  |  |  |  |  |  |  |  |  | |  |  |  |
| *No* | 3 | 0.27 | 8 | 0.73 | 4 | 0.36 | 7 | 0.64 | 0.3173 | 1.524 | (0.176-13.161) | 0.6726 | NA | | NA | NA |  |
| *Yes* | 110 | 0.48 | 117 | 0.52 | 97 | 0.43 | 130 | 0.57 | 0.1536 | 0.794 | (0.547-1.152) | 0.2231 | 0.767 | | (0.511-1.152) | 0.2007 |  |
| *Mild liver disease* |  |  |  |  |  |  |  |  |  |  |  |  |  | |  |  |  |
| *No* | 81 | 0.50 | 80 | 0.50 | 69 | 0.43 | 92 | 0.57 | 0.1213 | 0.741 | (0.475-1.155) | 0.1837 | 0.690 | | (0.418-1.142) | 0.1477 |  |
| *Yes* | 32 | 0.42 | 45 | 0.58 | 32 | 0.42 | 45 | 0.58 | 1.0000 | 1.000 | (0.519-1.926) | 1.0000 | 1.000 | | (0.474-2.111) | 1.0000 |  |
| *Diabetes with chronic complications* |  |  |  |  |  |  |  |  |  |  |  |  |  | |  |  |  |
| *No* | 97 | 0.50 | 98 | 0.50 | 94 | 0.48 | 101 | 0.52 | 0.7218 | 0.940 | (0.630-1.404) | 0.7621 | 0.932 | | (0.601-1.445) | 0.7518 |  |
| *Yes* | 16 | 0.37 | 27 | 0.63 | 7 | 0.16 | 36 | 0.84 | 0.0126 | 0.328 | (0.114-0.948) | 0.0401 | 0.145 | | (0.037-0.567) | 0.0066 |  |
| *Any malignancy, including lymphoma and leukemia* |  |  |  |  |  |  |  |  |  |  |  |  |  | |  |  |  |
| *No* | 99 | 0.50 | 101 | 0.51 | 91 | 0.46 | 109 | 0.55 | 0.3458 | 0.852 | (0.573-1.266) | 0.4254 | 0.832 | | (0.539-1.284) | 0.4040 |  |
| *Yes* | 14 | 0.37 | 24 | 0.63 | 10 | 0.26 | 28 | 0.74 | 0.2482 | 0.612 | (0.220-1.705) | 0.3381 | 0.498 | | (0.127-1.945) | 0.3062 |  |
| **Index year** |  |  |  |  |  |  |  |  |  |  |  |  |  | |  |  |  |
| *2017* | 60 | 0.45 | 74 | 0.55 | 55 | 0.41 | 79 | 0.59 | 0.4751 | 0.859 | (0.526-1.402) | 0.5399 | 0.830 | | (0.475-1.449) | 0.5091 |  |
| *2018* | 53 | 0.51 | 51 | 0.49 | 46 | 0.44 | 58 | 0.56 | 0.2367 | 0.763 | (0.438-1.329) | 0.3361 | 0.720 | | (0.381-1.360) | 0.3082 |  |

6.3.2. Frequency of antibiotics-used COPD AE

6.3.2.1 Moderate exacerbation

| **MODERATE COPD EXACERBATION** | **All** | **Pre-BV** | **Post-BV** | **Relative frequency** | **p-value** |
| --- | --- | --- | --- | --- | --- |
|  |  |  |  |  |  |
| ***Number of event per  person per year*** | 0.70 | 0.81 | 0.59 | 0.73 | 0.0876 |
| *Total event* | 333 | 193 | 140 |  |  |
| *Patients with event* | 156 | 83 | 73 |  |  |
| *observational period (year)* |  |  |  |  |  |
| *Mean* | 365.04 | 365.08 | 365.00 |  |  |
| *SD* | 0.20 | 0.28 | 0.00 |  |  |
| *Median* | 365 | 365 | 365 |  |  |
| *Min* | 365 | 365 | 365 |  |  |
| *Max* | 366 | 366 | 365 |  |  |
| *P25* | 365 | 365 | 365 |  |  |
| *P75* | 365 | 365 | 365 |  |  |
| **COPD medication during pre and post-BV period** |  |  |  |  |  |
| *ICS/LABA* | 0.46 | 0.47 | 0.44 | 0.94 | 0.8476 |
| *LAMA* | 0.94 | 1.17 | 0.71 | 0.61 | 0.3976 |
| *Triple* | 0.98 | 1.06 | 0.90 | 0.85 | 0.5535 |
| *LABA/LAMA* | 0.79 | 1.21 | 0.38 | 0.32 | 0.0407 |
| **Age at index date 1** |  |  |  |  |  |
| *40 to < 65* | 0.89 | 1.16 | 0.61 | 0.53 | 0.0625 |
| *65 to < 75* | 0.72 | 0.68 | 0.75 | 1.11 | 0.6997 |
| *75+* | 0.47 | 0.66 | 0.27 | 0.41 | 0.0260 |
| **Sex** |  |  |  |  |  |
| *Male* | 0.66 | 0.74 | 0.57 | 0.77 | 0.2111 |
| *Female* | 0.87 | 1.08 | 0.65 | 0.60 | 0.2342 |
| **Insurance type at index date2** |  |  |  |  |  |
| *Health insurance* | 0.69 | 0.76 | 0.61 | 0.81 | 0.2966 |
| *Medical aid* | 0.79 | 1.16 | 0.42 | 0.36 | 0.0460 |
| **Hospital type at index date2** |  |  |  |  |  |
| *Tertiary and general hospital* | 0.76 | 0.90 | 0.61 | 0.68 | 0.0587 |
| *Others* | 0.44 | 0.40 | 0.48 | 1.18 | 0.7329 |
| **History of COPD exacerbation** |  |  |  |  |  |
| *none* | 0.32 | 0.32 | 0.32 | 1.00 | 0.9998 |
| *1 moderate* | 0.98 | 1.25 | 0.71 | 0.57 | 0.0406 |
| *≥ 2 moderate OR ≥ 1 severe* | 1.89 | 2.05 | 1.73 | 0.84 | 0.6369 |
| **History of Pneumonia** |  |  |  |  |  |
| *No* | 0.51 | 0.62 | 0.39 | 0.64 | 0.0664 |
| *Yes* | 1.24 | 1.35 | 1.13 | 0.84 | 0.5147 |
| **History of Asthma** |  |  |  |  |  |
| *No* | 0.46 | 0.50 | 0.42 | 0.84 | 0.6937 |
| *Yes* | 0.75 | 0.87 | 0.62 | 0.71 | 0.0960 |
| **mCCI** |  |  |  |  |  |
| *0, 1* | 0.91 | 0.98 | 0.83 | 0.84 | 0.4703 |
| *2 +* | 0.50 | 0.65 | 0.36 | 0.56 | 0.0525 |
| **mCCI category** |  |  |  |  |  |
| *Congestive heart failure* |  |  |  |  |  |
| *No* | 0.70 | 0.80 | 0.61 | 0.76 | 0.1838 |
| *Yes* | 0.69 | 0.89 | 0.49 | 0.55 | 0.1815 |
| *Dementia* |  |  |  |  |  |
| *No* | 0.70 | 0.81 | 0.60 | 0.73 | 0.1060 |
| *Yes* | 0.58 | 0.83 | 0.33 | 0.40 | 0.2590 |
| *Chronic pulmonary disease* |  |  |  |  |  |
| *No* | 0.32 | 0.27 | 0.36 | 1.33 | 0.7561 |
| *Yes* | 0.72 | 0.84 | 0.60 | 0.72 | 0.0800 |
| *Rheumatologic disease* |  |  |  |  |  |
| *No* | 0.72 | 0.84 | 0.60 | 0.71 | 0.0739 |
| *Yes* | 0.33 | 0.25 | 0.42 | 1.67 | 0.6070 |
| *Mild liver disease* |  |  |  |  |  |
| *No* | 0.77 | 0.87 | 0.68 | 0.78 | 0.2520 |
| *Yes* | 0.55 | 0.69 | 0.40 | 0.58 | 0.1430 |
| *Diabetes with chronic complications* |  |  |  |  |  |
| *No* | 0.82 | 0.93 | 0.70 | 0.75 | 0.1250 |
| *Yes* | 0.17 | 0.26 | 0.09 | 0.36 | 0.2230 |
| *Hemiplegia or paraplegia* |  |  |  |  |  |
| *No* | 0.70 | 0.82 | 0.59 | 0.72 | 0.0762 |
| *Yes* | 0.33 | NA | 0.67 | NA | NA |
| *Renal disease* |  |  |  |  |  |
| *No* | 0.72 | 0.83 | 0.61 | 0.74 | 0.1046 |
| *Yes* | 0.23 | 0.36 | 0.09 | 0.25 | 0.3193 |
| *Any malignancy, including lymphoma and leukemia* |  |  |  |  |  |
| *No* | 0.77 | 0.87 | 0.67 | 0.77 | 0.1818 |
| *Yes* | 0.36 | 0.53 | 0.18 | 0.35 | 0.0890 |
| *Moderate or severe liver disease* |  |  |  |  |  |
| *No* | 0.70 | 0.81 | 0.59 | 0.73 | 0.0997 |
| *Yes* | 1.00 | 2.00 | NA | NA | NA |
| *Metastatic solid tumor* |  |  |  |  |  |
| *No* | 0.70 | 0.81 | 0.59 | 0.73 | 0.0874 |
| *Yes* | NA | NA | NA | NA | NA |
| *HIV* |  |  |  |  |  |
| *No* | 0.70 | 0.81 | 0.59 | 0.73 | 0.0876 |
| *Yes* | NA | NA | NA | NA | NA |
| **Index year** |  |  |  |  |  |
| *2017* | 0.68 | 0.81 | 0.55 | 0.67 | 0.1104 |
| *2018* | 0.73 | 0.81 | 0.64 | 0.80 | 0.4273 |

6.3.2.2 Severe exacerbation

| **SEVERE COPD EXACERBATION** | **All** | **Pre-BV** | **Post-BV** | **Relative frequency** | **p-value** |
| --- | --- | --- | --- | --- | --- |
|  |  |  |  |  |  |
| ***Number of event per  person per year*** | 0.24 | 0.28 | 0.21 | 0.73 | 0.1598 |
| *Total event* | 116 | 67 | 49 |  |  |
| *Patients with event* | 89 | 49 | 40 |  |  |
| *observational period (year)* |  |  |  |  |  |
| *Mean* | 365.04 | 365.08 | 365.00 |  |  |
| *SD* | 0.20 | 0.28 | 0.00 |  |  |
| *Median* | 365 | 365 | 365 |  |  |
| *Min* | 365 | 365 | 365 |  |  |
| *Max* | 366 | 366 | 365 |  |  |
| *P25* | 365 | 365 | 365 |  |  |
| *P75* | 365 | 365 | 365 |  |  |
| **COPD medication during pre and post-BV period** |  |  |  |  |  |
| *ICS/LABA* | 0.21 | 0.26 | 0.17 | 0.66 | 0.2386 |
| *LAMA* | 0.19 | 0.25 | 0.13 | 0.50 | 0.4114 |
| *Triple* | 0.29 | 0.36 | 0.22 | 0.63 | 0.2036 |
| *LABA/LAMA* | 0.29 | 0.24 | 0.35 | 1.50 | 0.4535 |
| **Age at index date 1** |  |  |  |  |  |
| *40 to < 65* | 0.15 | 0.16 | 0.13 | 0.82 | 0.7202 |
| *65 to < 75* | 0.28 | 0.32 | 0.24 | 0.74 | 0.3395 |
| *75+* | 0.28 | 0.34 | 0.23 | 0.67 | 0.2956 |
| **Sex** |  |  |  |  |  |
| *Male* | 0.26 | 0.29 | 0.22 | 0.75 | 0.2258 |
| *Female* | 0.19 | 0.23 | 0.15 | 0.64 | 0.4682 |
| **Insurance type at index date2** |  |  |  |  |  |
| *Health insurance* | 0.25 | 0.29 | 0.21 | 0.72 | 0.1645 |
| *Medical aid* | 0.18 | 0.19 | 0.16 | 0.83 | 0.7985 |
| **Hospital type at index date2** |  |  |  |  |  |
| *Tertiary and general hospital* | 0.27 | 0.31 | 0.23 | 0.74 | 0.1887 |
| *Others* | 0.12 | 0.14 | 0.10 | 0.67 | 0.6012 |
| **History of COPD exacerbation** |  |  |  |  |  |
| *none* | 0.24 | 0.24 | 0.23 | 0.94 | 0.8324 |
| *1 moderate* | 0.25 | 0.31 | 0.19 | 0.62 | 0.1694 |
| *≥ 2 moderate OR ≥ 1 severe* | 0.27 | 0.41 | 0.14 | 0.33 | 0.1873 |
| **History of Pneumonia** |  |  |  |  |  |
| *No* | 0.21 | 0.23 | 0.18 | 0.80 | 0.3822 |
| *Yes* | 0.35 | 0.43 | 0.27 | 0.63 | 0.2681 |
| **History of Asthma** |  |  |  |  |  |
| *No* | 0.17 | 0.24 | 0.11 | 0.44 | 0.2023 |
| *Yes* | 0.26 | 0.29 | 0.23 | 0.78 | 0.2859 |
| **mCCI** |  |  |  |  |  |
| *0, 1* | 0.24 | 0.29 | 0.18 | 0.62 | 0.1218 |
| *2 +* | 0.25 | 0.27 | 0.23 | 0.85 | 0.6057 |
| **mCCI category** |  |  |  |  |  |
| *Congestive heart failure* |  |  |  |  |  |
| *No* | 0.24 | 0.28 | 0.21 | 0.74 | 0.1983 |
| *Yes* | 0.24 | 0.29 | 0.20 | 0.70 | 0.5804 |
| *Dementia* |  |  |  |  |  |
| *No* | 0.24 | 0.29 | 0.19 | 0.67 | 0.0821 |
| *Yes* | 0.33 | NA | 0.67 | NA | NA |
| *Chronic pulmonary disease* |  |  |  |  |  |
| *No* | 0.14 | 0.18 | 0.09 | 0.50 | 0.5743 |
| *Yes* | 0.25 | 0.29 | 0.21 | 0.74 | 0.1803 |
| *Rheumatologic disease* |  |  |  |  |  |
| *No* | 0.25 | 0.29 | 0.21 | 0.72 | 0.1556 |
| *Yes* | 0.17 | 0.17 | 0.17 | 1.00 | 1.0000 |
| *Mild liver disease* |  |  |  |  |  |
| *No* | 0.23 | 0.29 | 0.16 | 0.55 | 0.0267 |
| *Yes* | 0.28 | 0.26 | 0.30 | 1.15 | 0.7277 |
| *Diabetes with chronic complications* |  |  |  |  |  |
| *No* | 0.24 | 0.28 | 0.21 | 0.74 | 0.2299 |
| *Yes* | 0.26 | 0.30 | 0.21 | 0.69 | 0.4604 |
| *Hemiplegia or paraplegia* |  |  |  |  |  |
| *No* | 0.24 | 0.28 | 0.19 | 0.68 | 0.0912 |
| *Yes* | 0.83 | 0.33 | 1.33 | 4.00 | 0.4012 |
| *Renal disease* |  |  |  |  |  |
| *No* | 0.24 | 0.29 | 0.20 | 0.71 | 0.1248 |
| *Yes* | 0.23 | 0.18 | 0.27 | 1.50 | 0.7570 |
| *Any malignancy, including lymphoma and leukemia* |  |  |  |  |  |
| *No* | 0.25 | 0.30 | 0.21 | 0.70 | 0.1316 |
| *Yes* | 0.21 | 0.21 | 0.21 | 1.00 | 0.9991 |
| *Moderate or severe liver disease* |  |  |  |  |  |
| *No* | 0.24 | 0.28 | 0.21 | 0.73 | 0.1597 |
| *Yes* | NA | NA | NA | NA | NA |
| *Metastatic solid tumor* |  |  |  |  |  |
| *No* | 0.24 | 0.28 | 0.21 | 0.73 | 0.1597 |
| *Yes* | NA | NA | NA | NA | NA |
| *HIV* |  |  |  |  |  |
| *No* | 0.24 | 0.28 | 0.21 | 0.73 | 0.1598 |
| *Yes* | NA | NA | NA | NA | NA |
| **Index year** |  |  |  |  |  |
| *2017* | 0.28 | 0.34 | 0.22 | 0.67 | 0.1642 |
| *2018* | 0.20 | 0.21 | 0.18 | 0.86 | 0.6649 |

6.3.2.3 Moderate-to-severe exacerbation

| **MODERATE TO SEVERE COPD EXACERBATION** | **All** | **Pre-BV** | **Post-BV** | **Relative frequency** | **p-value** |
| --- | --- | --- | --- | --- | --- |
|  |  |  |  |  |  |
| ***Number of event per  person per year*** | 0.94 | 1.09 | 0.79 | 0.73 | 0.0351 |
| *Total event* | 449 | 260 | 189 |  |  |
| *Patients with event* | 214 | 113 | 101 |  |  |
| *observational period (year)* |  |  |  |  |  |
| *Mean* | 365.04 | 365.08 | 365.00 |  |  |
| *SD* | 0.20 | 0.28 | 0.00 |  |  |
| *Median* | 365 | 365 | 365 |  |  |
| *Min* | 365 | 365 | 365 |  |  |
| *Max* | 366 | 366 | 365 |  |  |
| *P25* | 365 | 365 | 365 |  |  |
| *P75* | 365 | 365 | 365 |  |  |
| **COPD medication during pre and post-BV period** |  |  |  |  |  |
| *ICS/LABA* | 0.67 | 0.73 | 0.61 | 0.84 | 0.4636 |
| *LAMA* | 1.13 | 1.42 | 0.83 | 0.59 | 0.3121 |
| *Triple* | 1.27 | 1.42 | 1.12 | 0.79 | 0.3217 |
| *LABA/LAMA* | 1.09 | 1.44 | 0.74 | 0.51 | 0.0956 |
| **Age at index date 1** |  |  |  |  |  |
| *40 to < 65* | 1.04 | 1.33 | 0.75 | 0.56 | 0.0599 |
| *65 to < 75* | 1.00 | 1.00 | 0.99 | 0.99 | 0.9654 |
| *75+* | 0.75 | 1.00 | 0.50 | 0.50 | 0.0160 |
| **Sex** |  |  |  |  |  |
| *Male* | 0.92 | 1.04 | 0.80 | 0.77 | 0.1016 |
| *Female* | 1.05 | 1.31 | 0.79 | 0.60 | 0.1816 |
| **Insurance type at index date2** |  |  |  |  |  |
| *Health insurance* | 0.94 | 1.05 | 0.83 | 0.78 | 0.1332 |
| *Medical aid* | 0.97 | 1.36 | 0.58 | 0.43 | 0.0531 |
| **Hospital type at index date2** |  |  |  |  |  |
| *Tertiary and general hospital* | 1.03 | 1.21 | 0.84 | 0.70 | 0.0246 |
| *Others* | 0.56 | 0.55 | 0.57 | 1.04 | 0.9176 |
| **History of COPD exacerbation** |  |  |  |  |  |
| *none* | 0.56 | 0.57 | 0.55 | 0.97 | 0.8968 |
| *1 moderate* | 1.22 | 1.55 | 0.89 | 0.58 | 0.0167 |
| *≥ 2 moderate OR ≥ 1 severe* | 2.16 | 2.46 | 1.86 | 0.76 | 0.4150 |
| **History of Pneumonia** |  |  |  |  |  |
| *No* | 0.71 | 0.85 | 0.58 | 0.68 | 0.0385 |
| *Yes* | 1.59 | 1.78 | 1.40 | 0.79 | 0.3075 |
| **History of Asthma** |  |  |  |  |  |
| *No* | 0.63 | 0.74 | 0.53 | 0.71 | 0.3474 |
| *Yes* | 1.00 | 1.16 | 0.85 | 0.73 | 0.0528 |
| **mCCI** |  |  |  |  |  |
| *0, 1* | 1.14 | 1.28 | 1.01 | 0.79 | 0.2344 |
| *2 +* | 0.75 | 0.92 | 0.59 | 0.64 | 0.0575 |
| **mCCI category** |  |  |  |  |  |
| *Congestive heart failure* |  |  |  |  |  |
| *No* | 0.95 | 1.08 | 0.81 | 0.75 | 0.0900 |
| *Yes* | 0.93 | 1.17 | 0.69 | 0.59 | 0.1371 |
| *Dementia* |  |  |  |  |  |
| *No* | 0.94 | 1.10 | 0.79 | 0.72 | 0.0320 |
| *Yes* | 0.92 | 0.83 | 1.00 | 1.20 | 0.7687 |
| *Chronic pulmonary disease* |  |  |  |  |  |
| *No* | 0.45 | 0.45 | 0.45 | 1.00 | 1.0000 |
| *Yes* | 0.97 | 1.12 | 0.81 | 0.72 | 0.0337 |
| *Rheumatologic disease* |  |  |  |  |  |
| *No* | 0.97 | 1.13 | 0.81 | 0.71 | 0.0290 |
| *Yes* | 0.50 | 0.42 | 0.58 | 1.40 | 0.6148 |
| *Mild liver disease* |  |  |  |  |  |
| *No* | 1.00 | 1.16 | 0.84 | 0.72 | 0.0676 |
| *Yes* | 0.83 | 0.95 | 0.70 | 0.74 | 0.2959 |
| *Diabetes with chronic complications* |  |  |  |  |  |
| *No* | 1.06 | 1.21 | 0.90 | 0.75 | 0.0622 |
| *Yes* | 0.43 | 0.56 | 0.30 | 0.54 | 0.2162 |
| *Hemiplegia or paraplegia* |  |  |  |  |  |
| *No* | 0.94 | 1.10 | 0.78 | 0.71 | 0.0235 |
| *Yes* | 1.17 | 0.33 | 2.00 | 6.00 | 0.2761 |
| *Renal disease* |  |  |  |  |  |
| *No* | 0.97 | 1.12 | 0.82 | 0.73 | 0.0378 |
| *Yes* | 0.45 | 0.55 | 0.36 | 0.67 | 0.7237 |
| *Any malignancy, including lymphoma and leukemia* |  |  |  |  |  |
| *No* | 1.02 | 1.16 | 0.87 | 0.75 | 0.0705 |
| *Yes* | 0.57 | 0.74 | 0.40 | 0.54 | 0.1833 |
| *Moderate or severe liver disease* |  |  |  |  |  |
| *No* | 0.94 | 1.09 | 0.80 | 0.73 | 0.0404 |
| *Yes* | 1.00 | 2.00 | NA | NA | NA |
| *Metastatic solid tumor* |  |  |  |  |  |
| *No* | 0.95 | 1.10 | 0.80 | 0.73 | 0.0350 |
| *Yes* | NA | NA | NA | NA | NA |
| *HIV* |  |  |  |  |  |
| *No* | 0.94 | 1.09 | 0.79 | 0.73 | 0.0351 |
| *Yes* | NA | NA | NA | NA | NA |
| **Index year** |  |  |  |  |  |
| *2017* | 0.96 | 1.15 | 0.77 | 0.67 | 0.0471 |
| *2018* | 0.92 | 1.02 | 0.83 | 0.81 | 0.3619 |

6.3.3 Incidence rate of antibiotics-used COPD AE

6.3.3.1 Moderate exacerbation

| **MODERATE COPD EXACERBATION** | **All** | **Pre-BV** | **Post-BV** | **p-value** | **IRR** | **IRR-pvalue** |
| --- | --- | --- | --- | --- | --- | --- |
|  |  |  |  |  |  |  |
| ***Incidence rate per 1,000 PYs*** | 405.82 | 439.65 | 373.18 | 0.3064 | 0.85 | 0.3070 |
| *Patients with event* | 156 | 83 | 73 |  |  |  |
| *Sum of person years (PYs)* | 384.41 | 188.79 | 195.62 |  |  |  |
| *Time to event (KM estimated)* |  |  |  |  |  |  |
| *Median* | NA | NA | NA |  |  |  |
| *SE* | NA | NA | NA |  |  |  |
| *Time to event (descriptive)* |  |  |  |  |  |  |
| *Mean* | 152.22 | 150.87 | 153.75 |  |  |  |
| *SD* | 113.44 | 119.83 | 106.50 |  |  |  |
| *Median* | 127.00 | 116.00 | 133.00 |  |  |  |
| *Min* | 1.00 | 1.00 | 7.00 |  |  |  |
| *Max* | 365.00 | 365.00 | 362.00 |  |  |  |
| *P25* | 57.00 | 40.00 | 64.00 |  |  |  |
| *P75* | 252.50 | 272.00 | 243.00 |  |  |  |
| **COPD medication during pre and post-BV period** |  |  |  |  |  |  |
| *ICS/LABA* | 272.42 | 279.79 | 265.17 | 0.8451 | 0.95 | 0.8451 |
| *LAMA* | 504.75 | 593.55 | 418.63 | 0.4494 | 0.71 | 0.4524 |
| *Triple* | 640.72 | 659.10 | 622.76 | 0.8247 | 0.94 | 0.8247 |
| *LABA/LAMA* | 403.48 | 523.92 | 297.19 | 0.1783 | 0.57 | 0.1845 |
| **Age at index date 1** |  |  |  |  |  |  |
| *40 to < 65* | 490.06 | 503.79 | 476.55 | 0.8427 | 0.95 | 0.8427 |
| *65 to < 75* | 398.72 | 384.75 | 412.88 | 0.7679 | 1.07 | 0.7679 |
| *75+* | 334.05 | 471.21 | 214.42 | 0.0228 | 0.46 | 0.0270 |
| **Sex** |  |  |  |  |  |  |
| *Male* | 414.65 | 442.16 | 387.33 | 0.4543 | 0.88 | 0.4548 |
| *Female* | 369.85 | 428.60 | 319.34 | 0.4366 | 0.75 | 0.4374 |
| **Insurance type at index date2** |  |  |  |  |  |  |
| *Health insurance* | 384.26 | 398.83 | 369.88 | 0.6676 | 0.93 | 0.6677 |
| *Medical aid* | 564.13 | 769.35 | 395.38 | 0.0931 | 0.51 | 0.0987 |
| **Hospital type at index date2** |  |  |  |  |  |  |
| *Tertiary and general hospital* | 431.34 | 475.83 | 389.10 | 0.2447 | 0.82 | 0.2455 |
| *Others* | 298.32 | 293.55 | 303.25 | 0.9392 | 1.03 | 0.9392 |
| **History of COPD exacerbation** |  |  |  |  |  |  |
| *none* | 234.61 | 230.38 | 238.83 | 0.8938 | 1.04 | 0.8938 |
| *1 moderate* | 585.29 | 702.60 | 477.31 | 0.1002 | 0.68 | 0.1025 |
| *≥ 2 moderate OR ≥ 1 severe* | 1109.10 | 1185.05 | 1042.30 | 0.7342 | 0.88 | 0.7341 |
| **History of Pneumonia** |  |  |  |  |  |  |
| *No* | 310.86 | 346.29 | 276.51 | 0.2789 | 0.80 | 0.2801 |
| *Yes* | 739.16 | 770.88 | 709.05 | 0.7401 | 0.92 | 0.7401 |
| **History of Asthma** |  |  |  |  |  |  |
| *No* | 342.27 | 370.88 | 313.28 | 0.6929 | 0.84 | 0.6935 |
| *Yes* | 418.58 | 453.87 | 384.86 | 0.3401 | 0.85 | 0.3406 |
| **mCCI** |  |  |  |  |  |  |
| *0, 1* | 509.12 | 524.28 | 494.19 | 0.7792 | 0.94 | 0.7793 |
| *2 +* | 317.87 | 366.16 | 272.09 | 0.2293 | 0.74 | 0.2312 |
| **mCCI category** |  |  |  |  |  |  |
| *Congestive heart failure* |  |  |  |  |  |  |
| *No* | 402.81 | 420.41 | 385.65 | 0.6202 | 0.92 | 0.6203 |
| *Yes* | 423.23 | 554.70 | 303.39 | 0.1451 | 0.55 | 0.1524 |
| *Dementia* |  |  |  |  |  |  |
| *No* | 398.39 | 425.03 | 372.42 | 0.4187 | 0.88 | 0.4191 |
| *Yes* | 760.41 | 1370.54 | 402.26 | 0.1411 | 0.29 | 0.1569 |
| *Chronic pulmonary disease* |  |  |  |  |  |  |
| *No* | 241.66 | 186.31 | 301.36 | 0.5941 | 1.62 | 0.5983 |
| *Yes* | 415.16 | 454.92 | 377.03 | 0.2491 | 0.83 | 0.2498 |
| *Rheumatologic disease* |  |  |  |  |  |  |
| *No* | 415.52 | 454.45 | 378.04 | 0.2586 | 0.83 | 0.2593 |
| *Yes* | 238.04 | 189.54 | 287.00 | 0.6461 | 1.51 | 0.6495 |
| *Mild liver disease* |  |  |  |  |  |  |
| *No* | 431.30 | 474.24 | 390.41 | 0.3083 | 0.82 | 0.3090 |
| *Yes* | 355.59 | 372.80 | 338.54 | 0.7438 | 0.91 | 0.7439 |
| *Diabetes with chronic complications* |  |  |  |  |  |  |
| *No* | 483.67 | 524.22 | 444.77 | 0.3195 | 0.85 | 0.3200 |
| *Yes* | 111.83 | 125.01 | 98.81 | 0.7250 | 0.79 | 0.7259 |
| *Hemiplegia or paraplegia* |  |  |  |  |  |  |
| *No* | 405.17 | 446.72 | 365.43 | 0.2132 | 0.82 | 0.2141 |
| *Yes* | 463.52 | NA | 1509.30 | NA | NA | NA |
| *Renal disease* |  |  |  |  |  |  |
| *No* | 419.78 | 452.59 | 388.12 | 0.3423 | 0.86 | 0.3428 |
| *Yes* | 150.56 | 203.71 | 98.93 | 0.5432 | 0.49 | 0.5554 |
| *Any malignancy, including lymphoma and leukemia* |  |  |  |  |  |  |
| *No* | 443.28 | 460.34 | 426.54 | 0.6519 | 0.93 | 0.6520 |
| *Yes* | 233.31 | 339.68 | 138.14 | 0.0821 | 0.41 | 0.0953 |
| *Moderate or severe liver disease* |  |  |  |  |  |  |
| *No* | 404.48 | 434.80 | 375.10 | 0.3582 | 0.86 | 0.3587 |
| *Yes* | 837.73 | 5144.37 | NA | 0.3582 | NA | NA |
| *Metastatic solid tumor* |  |  |  |  |  |  |
| *No* | 407.94 | 441.98 | 375.10 | 0.3059 | 0.85 | 0.3065 |
| *Yes* | NA | NA | NA | NA | NA | NA |
| *HIV* |  |  |  |  |  |  |
| *No* | 405.82 | 439.65 | 373.18 | 0.3064 | 0.85 | 0.3070 |
| *Yes* | NA | NA | NA | NA | NA | NA |
| **Index year** |  |  |  |  |  |  |
| *2017* | 367.53 | 393.33 | 342.13 | 0.5306 | 0.87 | 0.5311 |
| *2018* | 457.28 | 503.36 | 413.97 | 0.3976 | 0.82 | 0.3983 |

6.3.3.2 Severe exacerbation

| **SEVERE COPD EXACERBATION** | **All** | **Pre-BV** | **Post-BV** | **p-value** | **IRR** | **IRR-pvalue** |
| --- | --- | --- | --- | --- | --- | --- |
|  |  |  |  |  |  |  |
| ***Incidence rate per 1,000 PYs*** | 205.76 | 227.20 | 184.44 | 0.3265 | 0.81 | 0.3278 |
| *Patients with event* | 89 | 49 | 40 |  |  |  |
| *Sum of person years (PYs)* | 432.54 | 215.67 | 216.88 |  |  |  |
| *Time to event (KM estimated)* |  |  |  |  |  |  |
| *Median* | NA | NA | NA |  |  |  |
| *SE* | NA | NA | NA |  |  |  |
| *Time to event (descriptive)* |  |  |  |  |  |  |
| *Mean* | 189.92 | 203.24 | 173.60 |  |  |  |
| *SD* | 110.87 | 109.91 | 111.23 |  |  |  |
| *Median* | 181.00 | 177.00 | 186.50 |  |  |  |
| *Min* | 2.00 | 26.00 | 2.00 |  |  |  |
| *Max* | 364.00 | 364.00 | 338.00 |  |  |  |
| *P25* | 80.00 | 120.00 | 65.00 |  |  |  |
| *P75* | 279.00 | 289.00 | 268.00 |  |  |  |
| **COPD medication during pre and post-BV period** |  |  |  |  |  |  |
| *ICS/LABA* | 178.24 | 204.43 | 152.58 | 0.3757 | 0.75 | 0.3780 |
| *LAMA* | 160.27 | 182.99 | 137.50 | 0.7068 | 0.75 | 0.7083 |
| *Triple* | 247.96 | 285.35 | 211.69 | 0.4155 | 0.74 | 0.4177 |
| *LABA/LAMA* | 248.80 | 222.17 | 277.94 | 0.6648 | 1.25 | 0.6652 |
| **Age at index date 1** |  |  |  |  |  |  |
| *40 to < 65* | 118.78 | 109.77 | 127.96 | 0.7668 | 1.17 | 0.7671 |
| *65 to < 75* | 239.46 | 281.14 | 199.52 | 0.2422 | 0.71 | 0.2451 |
| *75+* | 245.51 | 268.53 | 221.74 | 0.6201 | 0.83 | 0.6211 |
| **Sex** |  |  |  |  |  |  |
| *Male* | 224.53 | 253.15 | 196.44 | 0.2674 | 0.78 | 0.2691 |
| *Female* | 133.93 | 130.99 | 137.00 | 0.9381 | 1.05 | 0.9381 |
| **Insurance type at index date2** |  |  |  |  |  |  |
| *Health insurance* | 213.49 | 241.86 | 185.51 | 0.2373 | 0.77 | 0.2392 |
| *Medical aid* | 155.67 | 135.09 | 177.27 | 0.6844 | 1.31 | 0.6854 |
| **Hospital type at index date2** |  |  |  |  |  |  |
| *Tertiary and general hospital* | 229.87 | 256.72 | 203.28 | 0.2950 | 0.79 | 0.2965 |
| *Others* | 99.80 | 99.06 | 100.55 | 0.9831 | 1.02 | 0.9831 |
| **History of COPD exacerbation** |  |  |  |  |  |  |
| *none* | 199.80 | 217.24 | 182.50 | 0.5468 | 0.84 | 0.5475 |
| *1 moderate* | 215.41 | 232.11 | 198.28 | 0.6517 | 0.85 | 0.6523 |
| *≥ 2 moderate OR ≥ 1 severe* | 204.57 | 271.28 | 145.09 | 0.3828 | 0.53 | 0.3915 |
| **History of Pneumonia** |  |  |  |  |  |  |
| *No* | 189.52 | 197.94 | 181.03 | 0.7274 | 0.91 | 0.7275 |
| *Yes* | 252.97 | 314.79 | 194.08 | 0.2056 | 0.62 | 0.2113 |
| **History of Asthma** |  |  |  |  |  |  |
| *No* | 155.67 | 203.26 | 110.42 | 0.3204 | 0.54 | 0.3303 |
| *Yes* | 215.54 | 231.75 | 199.28 | 0.5056 | 0.86 | 0.5062 |
| **mCCI** |  |  |  |  |  |  |
| *0, 1* | 206.78 | 237.34 | 176.83 | 0.3311 | 0.75 | 0.3335 |
| *2 +* | 204.77 | 217.52 | 191.91 | 0.6747 | 0.88 | 0.6750 |
| **mCCI category** |  |  |  |  |  |  |
| *Congestive heart failure* |  |  |  |  |  |  |
| *No* | 211.31 | 227.98 | 194.71 | 0.4867 | 0.85 | 0.4873 |
| *Yes* | 173.44 | 222.66 | 125.06 | 0.3479 | 0.56 | 0.3574 |
| *Dementia* |  |  |  |  |  |  |
| *No* | 203.29 | 233.68 | 173.42 | 0.1687 | 0.74 | 0.1709 |
| *Yes* | 315.69 | NA | 851.40 | NA | NA | NA |
| *Chronic pulmonary disease* |  |  |  |  |  |  |
| *No* | 143.54 | 195.74 | 93.61 | 0.5345 | 0.48 | 0.5470 |
| *Yes* | 208.92 | 228.77 | 189.14 | 0.3788 | 0.83 | 0.3799 |
| *Rheumatologic disease* |  |  |  |  |  |  |
| *No* | 207.02 | 229.61 | 184.57 | 0.3154 | 0.80 | 0.3168 |
| *Yes* | 182.15 | 182.26 | 182.03 | 0.9990 | 1.00 | 0.9990 |
| *Mild liver disease* |  |  |  |  |  |  |
| *No* | 203.43 | 247.60 | 160.49 | 0.0963 | 0.65 | 0.0999 |
| *Yes* | 210.75 | 185.00 | 237.62 | 0.5013 | 1.28 | 0.5026 |
| *Diabetes with chronic complications* |  |  |  |  |  |  |
| *No* | 199.69 | 207.89 | 191.47 | 0.7290 | 0.92 | 0.7291 |
| *Yes* | 233.80 | 318.42 | 152.67 | 0.1295 | 0.48 | 0.1415 |
| *Hemiplegia or paraplegia* |  |  |  |  |  |  |
| *No* | 201.03 | 225.43 | 176.85 | 0.2620 | 0.78 | 0.2637 |
| *Yes* | 633.75 | 365.62 | 1000.69 | 0.3939 | 2.74 | 0.4110 |
| *Renal disease* |  |  |  |  |  |  |
| *No* | 208.63 | 228.69 | 188.68 | 0.3736 | 0.83 | 0.3747 |
| *Yes* | 147.60 | 197.11 | 98.24 | 0.5581 | 0.50 | 0.5696 |
| *Any malignancy, including lymphoma and leukemia* |  |  |  |  |  |  |
| *No* | 211.89 | 243.77 | 180.43 | 0.1890 | 0.74 | 0.1914 |
| *Yes* | 173.56 | 142.19 | 206.02 | 0.5234 | 1.45 | 0.5265 |
| *Moderate or severe liver disease* |  |  |  |  |  |  |
| *No* | 206.71 | 228.26 | 185.29 | 0.3265 | 0.81 | 0.3277 |
| *Yes* | NA | NA | NA | NA | NA | NA |
| *Metastatic solid tumor* |  |  |  |  |  |  |
| *No* | 206.71 | 228.26 | 185.29 | 0.3265 | 0.81 | 0.3277 |
| *Yes* | NA | NA | NA | NA | NA | NA |
| *HIV* |  |  |  |  |  |  |
| *No* | 205.76 | 227.20 | 184.44 | 0.3265 | 0.81 | 0.3278 |
| *Yes* | NA | NA | NA | NA | NA | NA |
| **Index year** |  |  |  |  |  |  |
| *2017* | 223.78 | 251.69 | 196.53 | 0.3648 | 0.78 | 0.3664 |
| *2018* | 183.03 | 196.95 | 168.85 | 0.6495 | 0.86 | 0.6500 |

6.3.3.3 Moderate-to-severe exacerbation

| **MODERATE TO SEVERE COPD EXACERBATION** | **All** | **Pre-BV** | **Post-BV** | **p-value** | **IRR** | **IRR-pvalue** |
| --- | --- | --- | --- | --- | --- | --- |
|  |  |  |  |  |  |  |
| ***Incidence rate per 1,000 PYs*** | 604.10 | 644.39 | 564.60 | 0.3339 | 0.88 | 0.3344 |
| *Patients with event* | 214 | 113 | 101 |  |  |  |
| *Sum of person years (PYs)* | 354.25 | 175.36 | 178.89 |  |  |  |
| *Time to event (KM estimated)* |  |  |  |  |  |  |
| *Median* | NA | NA | NA |  |  |  |
| *SE* | NA | NA | NA |  |  |  |
| *Time to event (descriptive)* |  |  |  |  |  |  |
| *Mean* | 158.28 | 164.05 | 151.82 |  |  |  |
| *SD* | 113.99 | 119.92 | 107.20 |  |  |  |
| *Median* | 139.50 | 152.00 | 131.00 |  |  |  |
| *Min* | 1.00 | 1.00 | 2.00 |  |  |  |
| *Max* | 365.00 | 365.00 | 362.00 |  |  |  |
| *P25* | 57.00 | 53.00 | 63.00 |  |  |  |
| *P75* | 265.00 | 272.00 | 252.00 |  |  |  |
| **COPD medication during pre and post-BV period** |  |  |  |  |  |  |
| *ICS/LABA* | 443.57 | 457.60 | 429.73 | 0.7788 | 0.94 | 0.7788 |
| *LAMA* | 656.98 | 745.64 | 569.01 | 0.5186 | 0.76 | 0.5204 |
| *Triple* | 865.23 | 895.39 | 835.55 | 0.7631 | 0.93 | 0.7631 |
| *LABA/LAMA* | 685.64 | 807.45 | 570.82 | 0.3071 | 0.71 | 0.3099 |
| **Age at index date 1** |  |  |  |  |  |  |
| *40 to < 65* | 607.10 | 554.34 | 662.25 | 0.4913 | 1.19 | 0.4919 |
| *65 to < 75* | 629.96 | 653.17 | 607.06 | 0.7130 | 0.93 | 0.7130 |
| *75+* | 557.38 | 729.48 | 401.20 | 0.0318 | 0.55 | 0.0349 |
| **Sex** |  |  |  |  |  |  |
| *Male* | 629.13 | 674.05 | 584.54 | 0.3424 | 0.87 | 0.3430 |
| *Female* | 504.80 | 522.95 | 487.87 | 0.8350 | 0.93 | 0.8350 |
| **Insurance type at index date2** |  |  |  |  |  |  |
| *Health insurance* | 581.02 | 605.56 | 556.64 | 0.5711 | 0.92 | 0.5712 |
| *Medical aid* | 772.40 | 943.80 | 619.67 | 0.2290 | 0.66 | 0.2323 |
| **Hospital type at index date2** |  |  |  |  |  |  |
| *Tertiary and general hospital* | 652.79 | 714.32 | 593.90 | 0.2097 | 0.83 | 0.2104 |
| *Others* | 409.32 | 380.78 | 440.10 | 0.6967 | 1.16 | 0.6969 |
| **History of COPD exacerbation** |  |  |  |  |  |  |
| *none* | 428.78 | 445.16 | 412.60 | 0.7143 | 0.93 | 0.7143 |
| *1 moderate* | 800.56 | 860.65 | 740.26 | 0.4730 | 0.86 | 0.4736 |
| *≥ 2 moderate OR ≥ 1 severe* | 1266.62 | 1509.69 | 1069.77 | 0.3459 | 0.71 | 0.3466 |
| **History of Pneumonia** |  |  |  |  |  |  |
| *No* | 508.35 | 549.67 | 467.77 | 0.3404 | 0.85 | 0.3411 |
| *Yes* | 938.56 | 976.54 | 901.54 | 0.7311 | 0.92 | 0.7312 |
| **History of Asthma** |  |  |  |  |  |  |
| *No* | 501.72 | 581.23 | 425.59 | 0.3953 | 0.73 | 0.3976 |
| *Yes* | 624.89 | 657.04 | 593.22 | 0.4885 | 0.90 | 0.4888 |
| **mCCI** |  |  |  |  |  |  |
| *0, 1* | 713.68 | 719.98 | 707.38 | 0.9239 | 0.98 | 0.9239 |
| *2 +* | 509.70 | 578.08 | 443.78 | 0.1942 | 0.77 | 0.1958 |
| **mCCI category** |  |  |  |  |  |  |
| *Congestive heart failure* |  |  |  |  |  |  |
| *No* | 602.26 | 616.75 | 587.84 | 0.7460 | 0.95 | 0.7461 |
| *Yes* | 614.74 | 814.02 | 436.60 | 0.0823 | 0.54 | 0.0880 |
| *Dementia* |  |  |  |  |  |  |
| *No* | 591.56 | 632.10 | 551.79 | 0.3299 | 0.87 | 0.3303 |
| *Yes* | 1330.00 | 1370.54 | 1291.78 | 0.9333 | 0.94 | 0.9333 |
| *Chronic pulmonary disease* |  |  |  |  |  |  |
| *No* | 353.58 | 295.51 | 414.70 | 0.6554 | 1.40 | 0.6573 |
| *Yes* | 618.93 | 665.83 | 573.14 | 0.2813 | 0.86 | 0.2818 |
| *Rheumatologic disease* |  |  |  |  |  |  |
| *No* | 611.51 | 657.43 | 566.57 | 0.2874 | 0.86 | 0.2879 |
| *Yes* | 473.39 | 418.27 | 529.19 | 0.7250 | 1.27 | 0.7258 |
| *Mild liver disease* |  |  |  |  |  |  |
| *No* | 635.87 | 705.57 | 569.79 | 0.1911 | 0.81 | 0.1920 |
| *Yes* | 540.77 | 528.41 | 553.72 | 0.8516 | 1.05 | 0.8516 |
| *Diabetes with chronic complications* |  |  |  |  |  |  |
| *No* | 680.59 | 691.09 | 670.09 | 0.8312 | 0.97 | 0.8312 |
| *Yes* | 312.46 | 457.13 | 181.30 | 0.0328 | 0.40 | 0.0413 |
| *Hemiplegia or paraplegia* |  |  |  |  |  |  |
| *No* | 599.36 | 648.81 | 551.34 | 0.2386 | 0.85 | 0.2392 |
| *Yes* | 1032.51 | 365.62 | 2634.01 | 0.0598 | 7.20 | 0.0872 |
| *Renal disease* |  |  |  |  |  |  |
| *No* | 626.93 | 661.90 | 592.49 | 0.4224 | 0.90 | 0.4226 |
| *Yes* | 207.47 | 327.09 | 98.93 | 0.2635 | 0.30 | 0.3004 |
| *Any malignancy, including lymphoma and leukemia* |  |  |  |  |  |  |
| *No* | 653.25 | 683.68 | 623.08 | 0.5226 | 0.91 | 0.5228 |
| *Yes* | 378.58 | 458.20 | 304.50 | 0.3198 | 0.66 | 0.3237 |
| *Moderate or severe liver disease* |  |  |  |  |  |  |
| *No* | 603.31 | 639.40 | 567.77 | 0.3862 | 0.89 | 0.3866 |
| *Yes* | 837.73 | 5144.37 | NA | NA | NA | NA |
| *Metastatic solid tumor* |  |  |  |  |  |  |
| *No* | 607.52 | 648.07 | 567.77 | 0.3336 | 0.88 | 0.3340 |
| *Yes* | NA | NA | NA | NA | NA | NA |
| *HIV* |  |  |  |  |  |  |
| *No* | 604.10 | 644.39 | 564.60 | 0.3339 | 0.88 | 0.3344 |
| *Yes* | NA | NA | NA | NA | NA | NA |
| **Index year** |  |  |  |  |  |  |
| *2017* | 570.41 | 596.28 | 544.63 | 0.6273 | 0.91 | 0.6275 |
| *2018* | 648.59 | 709.17 | 590.48 | 0.3627 | 0.83 | 0.3634 |

6.3.4 Time to first antibiotics-used exacerbation

6.3.4.1 Moderate exacerbation


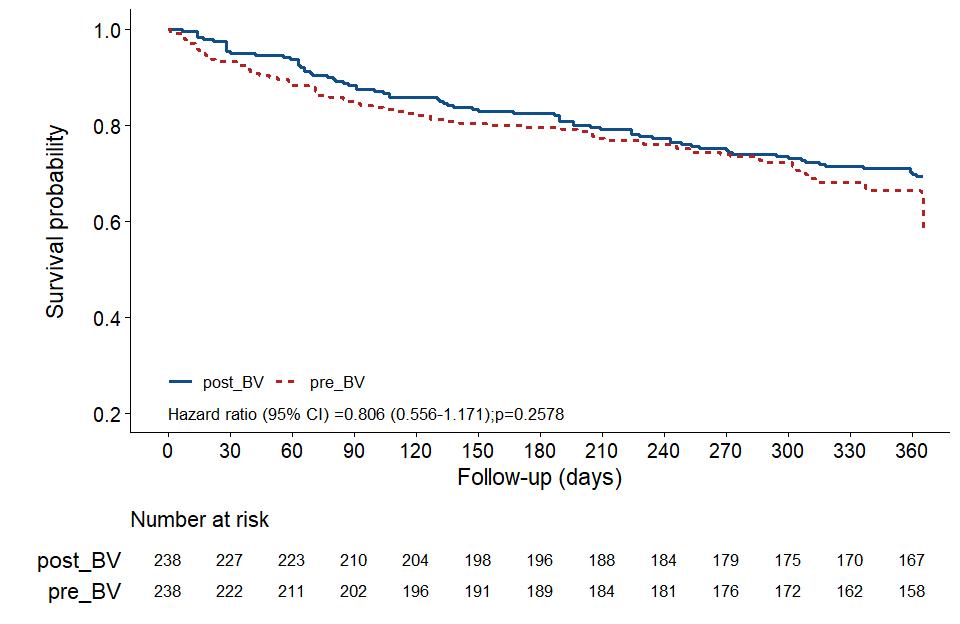


6.2.4.2. Severe exacerbation


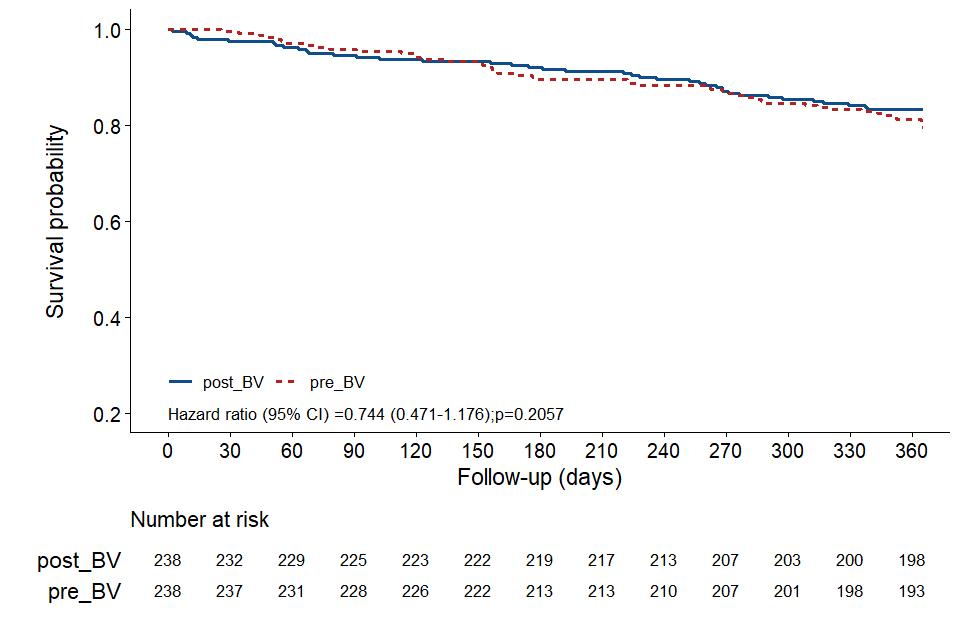


6.3.4.3 Moderate-to-severe exacerbation


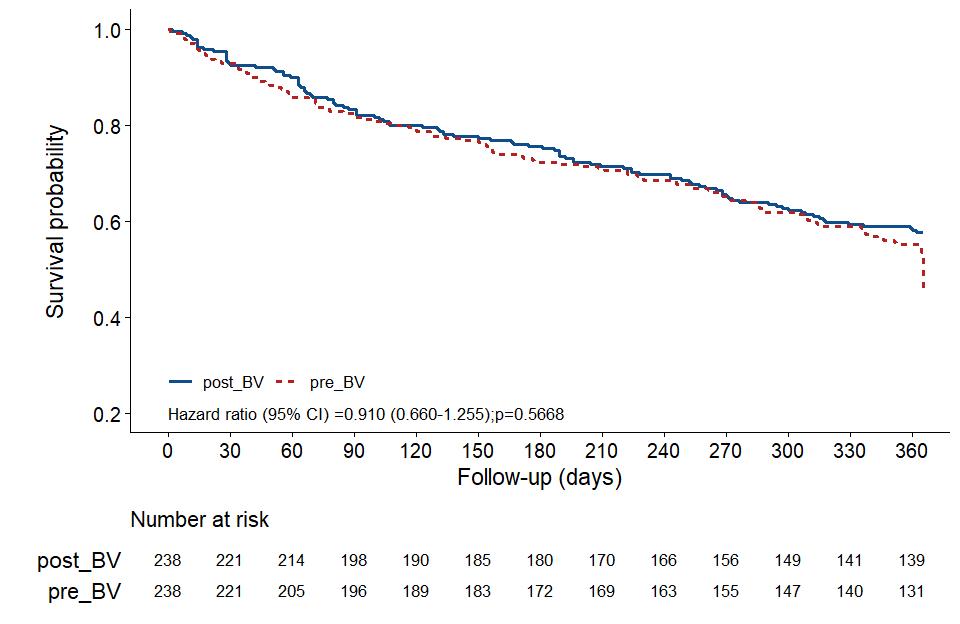


6.4 Subgroup analysis of OCS-used COPD AE

6.4.1 Odd ratio of OCS-used COPD AE

6.4.1.1. Moderate exacerbation

| **MODERATE COPD EXACERBATION** |  | | | | | | | | | | | | | | |
| --- | --- | --- | --- | --- | --- | --- | --- | --- | --- | --- | --- | --- | --- | --- | --- |
|  | **Pre-BV** | | | | **Post-BV** | | | | **Mcnemar p-value** | **Crude OR** | | | **Adjusted OR** | | |
|  | **Event** | | **No Event** | | **Event** | | **No Event** | |  |  |  |  |  |  |  |
|  | n | % | n | % | n | % | n | % |  | OR | 95% CI | p-value | OR | 95% CI | p-value |
| **Overall** | 40 | 0.17 | 198 | 0.83 | 26 | 0.11 | 212 | 0.89 | 0.0308 | 0.607 | (0.356-1.036) | 0.0669 | 0.577 | (0.336-0.992) | 0.0468 |
| **COPD medication during pre and post-BV period** |  |  |  |  |  |  |  |  |  |  |  |  |  |  |  |
| *ICS/LABA* | 17 | 0.15 | 96 | 0.85 | 12 | 0.11 | 101 | 0.89 | 0.2752 | 0.671 | (0.301-1.497) | 0.3264 | 0.707 | (0.362-1.381) | 0.3070 |
| *LAMA* | 3 | 0.13 | 21 | 0.88 | 0 | 0.00 | 24 | 1.00 | NA | NA | NA | NA | 0.604 | (0.321-1.137) | 0.1125 |
| *Triple* | 16 | 0.24 | 51 | 0.76 | 11 | 0.16 | 56 | 0.84 | 0.1317 | 0.626 | (0.260-1.508) | 0.2914 | 0.604 | (0.267-1.368) | 0.2227 |
| *LABA/LAMA* | 4 | 0.12 | 30 | 0.88 | 3 | 0.09 | 31 | 0.91 | 0.7055 | 0.726 | (0.138-3.831) | 0.6976 | 1.066 | (0.448-2.534) | 0.9408 |
| **Age at index date 1** |  |  |  |  |  |  |  |  |  |  |  |  |  |  |  |
| *40 to < 65* | 15 | 0.22 | 52 | 0.78 | 11 | 0.16 | 56 | 0.84 | 0.2059 | 0.681 | (0.280-1.654) | 0.3905 | 0.554 | (0.220-1.392) | 0.2052 |
| *65 to < 75* | 14 | 0.13 | 95 | 0.87 | 13 | 0.12 | 96 | 0.88 | 0.8273 | 0.919 | (0.405-2.085) | 0.8383 | 0.907 | (0.378-2.174) | 0.8254 |
| *75+* | 11 | 0.18 | 51 | 0.82 | 2 | 0.03 | 60 | 0.97 | 0.0067 | 0.155 | (0.031-0.763) | 0.0226 | 0.053 | (0.011-0.241) | 0.0003 |
| **Sex** |  |  |  |  |  |  |  |  |  |  |  |  |  |  |  |
| *Male* | 27 | 0.14 | 163 | 0.86 | 21 | 0.11 | 169 | 0.89 | 0.2888 | 0.750 | (0.406-1.388) | 0.3577 | 0.721 | (0.379-1.373) | 0.3179 |
| *Female* | 13 | 0.27 | 35 | 0.73 | 5 | 0.10 | 43 | 0.90 | 0.0114 | 0.313 | (0.098-1.004) | 0.0508 | NA | NA | NA |
| **Insurance type at index date2** |  |  |  |  |  |  |  |  |  |  |  |  |  |  |  |
| *Health insurance* | 35 | 0.17 | 172 | 0.83 | 18 | 0.09 | 189 | 0.91 | 0.0052 | 0.468 | (0.254-0.861) | 0.0149 | 0.432 | (0.228-0.820) | 0.0104 |
| *Medical aid* | 5 | 0.16 | 26 | 0.84 | 8 | 0.26 | 23 | 0.74 | 0.1797 | 1.809 | (0.481-6.800) | 0.3681 | 1.000 | (0.466-2.147) | 1.0000 |
| **Hospital type at index date2** |  |  |  |  |  |  |  |  |  |  |  |  |  |  |  |
| *Tertiary and general hospital* | 30 | 0.15 | 166 | 0.85 | 20 | 0.10 | 176 | 0.90 | 0.0771 | 0.629 | (0.342-1.157) | 0.1349 | 0.597 | (0.318-1.121) | 0.1080 |
| *Others* | 10 | 0.24 | 32 | 0.76 | 6 | 0.14 | 36 | 0.86 | 0.2059 | 0.533 | (0.166-1.712) | 0.2828 | 1.000 | (0.515-1.943) | 1.0000 |
| **History of COPD exacerbation** |  |  |  |  |  |  |  |  |  |  |  |  |  |  |  |
| *none* | 12 | 0.09 | 119 | 0.91 | 9 | 0.07 | 122 | 0.93 | 0.4669 | 0.732 | (0.294-1.822) | 0.4991 | 0.727 | (0.292-1.806) | 0.4890 |
| *1 moderate* | 19 | 0.22 | 66 | 0.78 | 14 | 0.16 | 71 | 0.84 | 0.2513 | 0.685 | (0.313-1.499) | 0.3394 | 0.654 | (0.282-1.518) | 0.3190 |
| *≥ 2 moderate OR ≥ 1 severe* | 9 | 0.41 | 13 | 0.59 | 3 | 0.14 | 19 | 0.86 | 0.0143 | 0.228 | (0.045-1.144) | 0.0704 | 0.290 | (0.088-0.955) | 0.0425 |
| **History of Pneumonia** |  |  |  |  |  |  |  |  |  |  |  |  |  |  |  |
| *No* | 28 | 0.16 | 147 | 0.84 | 19 | 0.11 | 156 | 0.89 | 0.1060 | 0.639 | (0.340-1.202) | 0.1635 | 0.814 | (0.530-1.251) | 0.3458 |
| *Yes* | 12 | 0.19 | 51 | 0.81 | 7 | 0.11 | 56 | 0.89 | 0.1317 | 0.531 | (0.189-1.495) | 0.2263 | 0.422 | (0.134-1.332) | 0.1385 |
| **History of Asthma** |  |  |  |  |  |  |  |  |  |  |  |  |  |  |  |
| *No* | 4 | 0.11 | 34 | 0.89 | 2 | 0.05 | 36 | 0.95 | 0.4142 | 0.472 | (0.075-2.988) | 0.4152 | 0.592 | (0.251-1.394) | 0.2223 |
| *Yes* | 36 | 0.18 | 164 | 0.82 | 24 | 0.12 | 176 | 0.88 | 0.0455 | 0.621 | (0.354-1.091) | 0.0972 | 0.590 | (0.326-1.070) | 0.0818 |
| **mCCI** |  |  |  |  |  |  |  |  |  |  |  |  |  |  |  |
| *0, 1* | 22 | 0.19 | 94 | 0.81 | 17 | 0.15 | 99 | 0.85 | 0.2752 | 0.734 | (0.363-1.483) | 0.3850 | 0.697 | (0.330-1.470) | 0.3396 |
| *2 +* | 18 | 0.15 | 104 | 0.85 | 9 | 0.07 | 113 | 0.93 | 0.0495 | 0.460 | (0.196-1.082) | 0.0749 | 0.541 | (0.266-1.102) | 0.0898 |
| **mCCI category** |  |  |  |  |  |  |  |  |  |  |  |  |  |  |  |
| *Congestive heart failure* |  |  |  |  |  |  |  |  |  |  |  |  |  |  |  |
| *No* | 35 | 0.17 | 168 | 0.83 | 23 | 0.11 | 180 | 0.89 | 0.0455 | 0.613 | (0.346-1.086) | 0.0932 | 0.643 | (0.386-1.073) | 0.0904 |
| *Yes* | 5 | 0.14 | 30 | 0.86 | 3 | 0.09 | 32 | 0.91 | 0.4142 | 0.563 | (0.114-2.770) | 0.4684 | 0.772 | (0.356-1.673) | 0.5012 |
| *Chronic pulmonary disease* |  |  |  |  |  |  |  |  |  |  |  |  |  |  |  |
| *No* | 0 | 0.00 | 11 | 1.00 | 0 | 0.00 | 11 | 1.00 | NA | NA | NA | NA | NA | NA | NA |
| *Yes* | 40 | 0.18 | 187 | 0.82 | 26 | 0.11 | 201 | 0.89 | 0.0308 | 0.605 | (0.354-1.034) | 0.0660 | 0.577 | (0.331-1.005) | 0.0521 |
| *Mild liver disease* |  |  |  |  |  |  |  |  |  |  |  |  |  |  |  |
| *No* | 28 | 0.17 | 133 | 0.83 | 19 | 0.12 | 142 | 0.88 | 0.0833 | 0.636 | (0.337-1.200) | 0.1609 | 0.592 | (0.303-1.157) | 0.1244 |
| *Yes* | 12 | 0.16 | 65 | 0.84 | 7 | 0.09 | 70 | 0.91 | 0.1967 | 0.542 | (0.196-1.493) | 0.2323 | 0.594 | (0.243-1.451) | 0.2492 |
| *Diabetes with chronic complications* |  |  |  |  |  |  |  |  |  |  |  |  |  |  |  |
| *No* | 32 | 0.16 | 163 | 0.84 | 21 | 0.11 | 174 | 0.89 | 0.0555 | 0.615 | (0.339-1.115) | 0.1088 | 0.650 | (0.388-1.089) | 0.1012 |
| *Yes* | 8 | 0.19 | 35 | 0.81 | 5 | 0.12 | 38 | 0.88 | 0.3173 | 0.576 | (0.164-2.027) | 0.3809 | 0.756 | (0.347-1.649) | 0.4738 |
| *Any malignancy, including lymphoma and leukemia* |  |  |  |  |  |  |  |  |  |  |  |  |  |  |  |
| *No* | 35 | 0.18 | 165 | 0.83 | 23 | 0.12 | 177 | 0.89 | 0.0455 | 0.613 | (0.346-1.086) | 0.0928 | 0.579 | (0.322-1.044) | 0.0689 |
| *Yes* | 5 | 0.13 | 33 | 0.87 | 3 | 0.08 | 35 | 0.92 | 0.4142 | 0.566 | (0.117-2.747) | 0.4697 | 0.477 | (0.186-1.223) | 0.1197 |
| **Index year** |  |  |  |  |  |  |  |  |  |  |  |  |  |  |  |
| *2017* | 18 | 0.13 | 116 | 0.87 | 15 | 0.11 | 119 | 0.89 | 0.5316 | 0.812 | (0.387-1.704) | 0.5799 | 0.779 | (0.360-1.688) | 0.5238 |
| *2018* | 22 | 0.21 | 82 | 0.79 | 11 | 0.11 | 93 | 0.89 | 0.0116 | 0.441 | (0.199-0.977) | 0.0437 | 0.383 | (0.161-0.911) | 0.0304 |

6.4.1.2. Severe exacerbation

| **SEVERE COPD EXACERBATION** |  | | | | | | | | | | | | | | |
| --- | --- | --- | --- | --- | --- | --- | --- | --- | --- | --- | --- | --- | --- | --- | --- |
|  | **Pre-BV** | | | | **Post-BV** | | | | **Mcnemar p-value** | **Crude OR** | | | **Adjusted OR** | | |
|  | **Event** | | **No Event** | | **Event** | | **No Event** | |  |  |  |  |  |  |  |
|  | n | % | n | % | n | % | n | % |  | OR | 95% CI | p-value | OR | 95% CI | p-value |
| **Overall** | 12 | 0.05 | 226 | 0.95 | 17 | 0.07 | 221 | 0.93 | 0.2513 | 1.449 | (0.673-3.121) | 0.3423 | 1.523 | (0.728-3.189) | 0.2629 |
| **COPD medication during pre and post-BV period** |  |  |  |  |  |  |  |  |  |  |  |  |  |  |  |
| *ICS/LABA* | 4 | 0.04 | 109 | 0.96 | 7 | 0.06 | 106 | 0.94 | 0.3173 | 1.800 | (0.502-6.450) | 0.3638 | 1.894 | (0.547-6.560) | 0.3104 |
| *LAMA* | 0 | 0.00 | 24 | 1.00 | 0 | 0.00 | 24 | 1.00 | NA | NA | NA | NA | NA | NA | NA |
| *Triple* | 3 | 0.04 | 64 | 0.96 | 3 | 0.04 | 64 | 0.96 | 1.0000 | 1.000 | (0.186-5.368) | 1.0000 | 1.000 | (0.133-7.526) | 1.0000 |
| *LABA/LAMA* | 5 | 0.15 | 29 | 0.85 | 7 | 0.21 | 27 | 0.79 | 0.4142 | 1.504 | (0.398-5.682) | 0.5367 | 1.747 | (0.388-7.858) | 0.4558 |
| **Age at index date 1** |  |  |  |  |  |  |  |  |  |  |  |  |  |  |  |
| *40 to < 65* | 5 | 0.07 | 62 | 0.93 | 4 | 0.06 | 63 | 0.94 | 0.6547 | 0.787 | (0.195-3.182) | 0.7335 | 0.862 | (0.435-1.708) | 0.6663 |
| *65 to < 75* | 5 | 0.05 | 104 | 0.95 | 10 | 0.09 | 99 | 0.91 | 0.1655 | 2.101 | (0.681-6.478) | 0.1940 | NA | NA | NA |
| *75+* | 2 | 0.03 | 60 | 0.97 | 3 | 0.05 | 59 | 0.95 | 0.3173 | 1.525 | (0.233-9.967) | 0.6544 | 1.007 | (0.729-1.390) | 0.9833 |
| **Sex** |  |  |  |  |  |  |  |  |  |  |  |  |  |  |  |
| *Male* | 10 | 0.05 | 180 | 0.95 | 16 | 0.08 | 174 | 0.92 | 0.1336 | 1.655 | (0.726-3.775) | 0.2295 | 1.803 | (0.826-3.937) | 0.1383 |
| *Female* | 2 | 0.04 | 46 | 0.96 | 1 | 0.02 | 47 | 0.98 | 0.5637 | 0.489 | (0.039-6.115) | 0.5719 | 0.969 | (0.672-1.396) | 0.9310 |
| **Insurance type at index date2** |  |  |  |  |  |  |  |  |  |  |  |  |  |  |  |
| *Health insurance* | 10 | 0.05 | 197 | 0.95 | 13 | 0.06 | 194 | 0.94 | 0.4054 | 1.320 | (0.561-3.104) | 0.5226 | 1.159 | (0.722-1.862) | 0.5387 |
| *Medical aid* | 2 | 0.06 | 29 | 0.94 | 4 | 0.13 | 27 | 0.87 | 0.4142 | 2.148 | (0.327-14.101) | 0.4132 | 1.000 | (0.548-1.823) | 1.0000 |
| **Hospital type at index date2** |  |  |  |  |  |  |  |  |  |  |  |  |  |  |  |
| *Tertiary and general hospital* | 9 | 0.05 | 187 | 0.95 | 15 | 0.08 | 181 | 0.92 | 0.1336 | 1.722 | (0.730-4.064) | 0.2135 | 1.829 | (0.814-4.109) | 0.1430 |
| *Others* | 3 | 0.07 | 39 | 0.93 | 2 | 0.05 | 40 | 0.95 | 0.5637 | 0.650 | (0.095-4.442) | 0.6532 | 1.011 | (0.000-3428103) | 0.9994 |
| **History of COPD exacerbation** |  |  |  |  |  |  |  |  |  |  |  |  |  |  |  |
| *none* | 6 | 0.05 | 125 | 0.95 | 9 | 0.07 | 122 | 0.93 | 0.3657 | 1.537 | (0.524-4.511) | 0.4312 | 1.682 | (0.692-4.090) | 0.2492 |
| *1 moderate* | 2 | 0.02 | 83 | 0.98 | 6 | 0.07 | 79 | 0.93 | 0.1025 | 3.152 | (0.597-16.632) | 0.1736 | 1.207 | (0.749-1.945) | 0.4348 |
| *≥ 2 moderate OR ≥ 1 severe* | 4 | 0.18 | 18 | 0.82 | 2 | 0.09 | 20 | 0.91 | 0.1573 | 0.450 | (0.063-3.223) | 0.4085 | 1.003 | (0.507-1.986) | 0.9962 |
| **History of Pneumonia** |  |  |  |  |  |  |  |  |  |  |  |  |  |  |  |
| *No* | 6 | 0.03 | 169 | 0.97 | 13 | 0.07 | 162 | 0.93 | 0.0707 | 2.260 | (0.831-6.149) | 0.1096 | 1.506 | (0.859-2.640) | 0.1514 |
| *Yes* | 6 | 0.10 | 57 | 0.90 | 4 | 0.06 | 59 | 0.94 | 0.3173 | 0.644 | (0.166-2.493) | 0.5182 | 0.969 | (0.679-1.384) | 0.9308 |
| **History of Asthma** |  |  |  |  |  |  |  |  |  |  |  |  |  |  |  |
| *No* | 1 | 0.03 | 37 | 0.97 | 3 | 0.08 | 35 | 0.92 | 0.1573 | 3.171 | (0.282-35.665) | 0.3401 | 0.987 | (0.210-4.643) | 0.9932 |
| *Yes* | 11 | 0.06 | 189 | 0.95 | 14 | 0.07 | 186 | 0.93 | 0.4669 | 1.293 | (0.568-2.943) | 0.5381 | 1.276 | (0.631-2.583) | 0.4959 |
| **mCCI** |  |  |  |  |  |  |  |  |  |  |  |  |  |  |  |
| *0, 1* | 4 | 0.03 | 112 | 0.97 | 7 | 0.06 | 109 | 0.94 | 0.2568 | 1.798 | (0.502-6.437) | 0.3640 | 2.040 | (0.254-16.363) | 0.4990 |
| *2 +* | 8 | 0.07 | 114 | 0.93 | 10 | 0.08 | 112 | 0.92 | 0.5637 | 1.272 | (0.478-3.388) | 0.6272 | NA | NA | NA |
| **mCCI category** |  |  |  |  |  |  |  |  |  |  |  |  |  |  |  |
| *Congestive heart failure* |  |  |  |  |  |  |  |  |  |  |  |  |  |  |  |
| *No* | 8 | 0.04 | 195 | 0.96 | 13 | 0.06 | 190 | 0.94 | 0.1655 | 1.668 | (0.671-4.147) | 0.2695 | 1.759 | (0.721-4.294) | 0.2133 |
| *Yes* | 4 | 0.11 | 31 | 0.89 | 4 | 0.11 | 31 | 0.89 | 1.0000 | 1.000 | (0.212-4.708) | 1.0000 | 1.020 | (0.419-2.484) | 0.9636 |
| *Chronic pulmonary disease* |  |  |  |  |  |  |  |  |  |  |  |  |  |  |  |
| *No* | 0 | 0.00 | 11 | 1.00 | 1 | 0.09 | 10 | 0.91 | NA | NA | NA | NA | NA | NA | NA |
| *Yes* | 12 | 0.05 | 215 | 0.95 | 16 | 0.07 | 211 | 0.93 | 0.3458 | 1.359 | (0.624-2.958) | 0.4385 | 1.421 | (0.672-3.004) | 0.3555 |
| *Mild liver disease* |  |  |  |  |  |  |  |  |  |  |  |  |  |  |  |
| *No* | 5 | 0.03 | 156 | 0.97 | 12 | 0.07 | 149 | 0.93 | 0.0348 | 2.513 | (0.854-7.389) | 0.0936 | 2.897 | (0.851-9.864) | 0.0883 |
| *Yes* | 7 | 0.09 | 70 | 0.91 | 5 | 0.06 | 72 | 0.94 | 0.4795 | 0.694 | (0.205-2.355) | 0.5538 | 0.611 | (0.124-3.005) | 0.5395 |
| *Diabetes with chronic complications* |  |  |  |  |  |  |  |  |  |  |  |  |  |  |  |
| *No* | 10 | 0.05 | 185 | 0.95 | 13 | 0.07 | 182 | 0.93 | 0.4054 | 1.321 | (0.561-3.113) | 0.5220 | 1.390 | (0.582-3.320) | 0.4565 |
| *Yes* | 2 | 0.05 | 41 | 0.95 | 4 | 0.09 | 39 | 0.91 | 0.4142 | 2.103 | (0.338-13.060) | 0.4162 | NA | NA | NA |
| *Any malignancy, including lymphoma and leukemia* |  |  |  |  |  |  |  |  |  |  |  |  |  |  |  |
| *No* | 10 | 0.05 | 190 | 0.95 | 14 | 0.07 | 186 | 0.93 | 0.3173 | 1.430 | (0.615-3.324) | 0.4040 | 1.222 | (0.737-2.026) | 0.4345 |
| *Yes* | 2 | 0.05 | 36 | 0.95 | 3 | 0.08 | 35 | 0.92 | 0.5637 | 1.543 | (0.222-10.702) | 0.6527 | 1.739 | (0.246-12.317) | 0.5703 |
| **Index year** |  |  |  |  |  |  |  |  |  |  |  |  |  |  |  |
| *2017* | 2 | 0.01 | 132 | 0.99 | 6 | 0.04 | 128 | 0.96 | 0.1573 | 3.094 | (0.600-15.943) | 0.1754 | 1.010 | (0.458-2.228) | 0.9897 |
| *2018* | 10 | 0.10 | 94 | 0.90 | 11 | 0.11 | 93 | 0.89 | 0.7630 | 1.112 | (0.444-2.785) | 0.8194 | 1.143 | (0.426-3.072) | 0.7884 |

6.4.1.3 Moderate-to-severe exacerbation

| **MODERATE TO SEVERE COPD EXACERBATION** |  | | | | | | | | | | | | | | |
| --- | --- | --- | --- | --- | --- | --- | --- | --- | --- | --- | --- | --- | --- | --- | --- |
|  | **Pre-BV** | | | | **Post-BV** | | | | **Mcnemar p-value** | **Crude OR** | | | **Adjusted OR** | | |
|  | **Event** | | **No Event** | | **Event** | | **No Event** | |  |  |  |  |  |  |  |
|  | n | % | n | % | n | % | n | % |  | OR | 95% CI | p-value | OR | 95% CI | p-value |
| **Overall** | 46 | 0.19 | 192 | 0.81 | 39 | 0.16 | 199 | 0.84 | 0.2967 | 0.818 | (0.509-1.314) | 0.4045 | 0.803 | (0.488-1.321) | 0.3856 |
| **COPD medication during pre and post-BV period** |  |  |  |  |  |  |  |  |  |  |  |  |  |  |  |
| *ICS/LABA* | 19 | 0.17 | 94 | 0.83 | 16 | 0.14 | 97 | 0.86 | 0.5316 | 0.816 | (0.392-1.700) | 0.5844 | 0.791 | (0.373-1.679) | 0.5380 |
| *LAMA* | 3 | 0.13 | 21 | 0.88 | 0 | 0.00 | 24 | 1.00 | NA | NA | NA | NA | 0.604 | (0.321-1.137) | 0.1125 |
| *Triple* | 17 | 0.25 | 50 | 0.75 | 13 | 0.19 | 54 | 0.81 | 0.2482 | 0.708 | (0.306-1.640) | 0.4147 | 0.788 | (0.389-1.593) | 0.5007 |
| *LABA/LAMA* | 7 | 0.21 | 27 | 0.79 | 10 | 0.29 | 24 | 0.71 | 0.2568 | 1.607 | (0.498-5.184) | 0.4157 | 1.731 | (0.405-7.396) | 0.4473 |
| **Age at index date 1** |  |  |  |  |  |  |  |  |  |  |  |  |  |  |  |
| *40 to < 65* | 15 | 0.22 | 52 | 0.78 | 14 | 0.21 | 53 | 0.79 | 0.7055 | 0.916 | (0.394-2.130) | 0.8357 | 0.856 | (0.340-2.160) | 0.7390 |
| *65 to < 75* | 19 | 0.17 | 90 | 0.83 | 21 | 0.19 | 88 | 0.81 | 0.7055 | 1.130 | (0.563-2.271) | 0.7283 | 1.149 | (0.547-2.416) | 0.7114 |
| *75+* | 12 | 0.19 | 50 | 0.81 | 4 | 0.06 | 58 | 0.94 | 0.0114 | 0.287 | (0.084-0.980) | 0.0465 | NA | NA | NA |
| **Sex** |  |  |  |  |  |  |  |  |  |  |  |  |  |  |  |
| *Male* | 33 | 0.17 | 157 | 0.83 | 34 | 0.18 | 156 | 0.82 | 0.8658 | 1.037 | (0.609-1.766) | 0.8933 | 1.042 | (0.590-1.840) | 0.8871 |
| *Female* | 13 | 0.27 | 35 | 0.73 | 5 | 0.10 | 43 | 0.90 | 0.0114 | 0.313 | (0.098-1.004) | 0.0508 | NA | NA | NA |
| **Insurance type at index date2** |  |  |  |  |  |  |  |  |  |  |  |  |  |  |  |
| *Health insurance* | 40 | 0.19 | 167 | 0.81 | 29 | 0.14 | 178 | 0.86 | 0.0782 | 0.680 | (0.402-1.152) | 0.1509 | 0.654 | (0.370-1.157) | 0.1441 |
| *Medical aid* | 6 | 0.19 | 25 | 0.81 | 10 | 0.32 | 21 | 0.68 | 0.1025 | 1.984 | (0.577-6.827) | 0.2664 | 2.541 | (0.651-9.920) | 0.1723 |
| **Hospital type at index date2** |  |  |  |  |  |  |  |  |  |  |  |  |  |  |  |
| *Tertiary and general hospital* | 35 | 0.18 | 161 | 0.82 | 31 | 0.16 | 165 | 0.84 | 0.5050 | 0.864 | (0.506-1.475) | 0.5910 | 0.853 | (0.487-1.495) | 0.5765 |
| *Others* | 11 | 0.26 | 31 | 0.74 | 8 | 0.19 | 34 | 0.81 | 0.3173 | 0.663 | (0.226-1.947) | 0.4455 | 0.751 | (0.319-1.769) | 0.5041 |
| **History of COPD exacerbation** |  |  |  |  |  |  |  |  |  |  |  |  |  |  |  |
| *none* | 16 | 0.12 | 115 | 0.88 | 16 | 0.12 | 115 | 0.88 | 1.0000 | 1.000 | (0.473-2.116) | 1.0000 | 1.000 | (0.357-2.802) | 1.0000 |
| *1 moderate* | 21 | 0.25 | 64 | 0.75 | 18 | 0.21 | 67 | 0.79 | 0.5127 | 0.819 | (0.394-1.702) | 0.5882 | 0.800 | (0.370-1.730) | 0.5663 |
| *≥ 2 moderate OR ≥ 1 severe* | 9 | 0.41 | 13 | 0.59 | 5 | 0.23 | 17 | 0.77 | 0.0455 | 0.425 | (0.102-1.763) | 0.2246 | 0.450 | (0.145-1.401) | 0.1585 |
| **History of Pneumonia** |  |  |  |  |  |  |  |  |  |  |  |  |  |  |  |
| *No* | 33 | 0.19 | 142 | 0.81 | 28 | 0.16 | 147 | 0.84 | 0.4233 | 0.820 | (0.468-1.434) | 0.4837 | 0.805 | (0.451-1.438) | 0.4619 |
| *Yes* | 13 | 0.21 | 50 | 0.79 | 11 | 0.17 | 52 | 0.83 | 0.4142 | 0.814 | (0.325-2.035) | 0.6545 | 0.730 | (0.201-2.652) | 0.6271 |
| **History of Asthma** |  |  |  |  |  |  |  |  |  |  |  |  |  |  |  |
| *No* | 5 | 0.13 | 33 | 0.87 | 5 | 0.13 | 33 | 0.87 | 1.0000 | 1.000 | (0.248-4.029) | 1.0000 | 1.000 | (0.543-1.843) | 1.0000 |
| *Yes* | 41 | 0.21 | 159 | 0.80 | 34 | 0.17 | 166 | 0.83 | 0.2623 | 0.794 | (0.478-1.321) | 0.3727 | 0.776 | (0.453-1.329) | 0.3541 |
| **mCCI** |  |  |  |  |  |  |  |  |  |  |  |  |  |  |  |
| *0, 1* | 23 | 0.20 | 93 | 0.80 | 22 | 0.19 | 94 | 0.81 | 0.8273 | 0.946 | (0.489-1.832) | 0.8690 | 0.938 | (0.458-1.923) | 0.8608 |
| *2 +* | 23 | 0.19 | 99 | 0.81 | 17 | 0.14 | 105 | 0.86 | 0.2207 | 0.697 | (0.348-1.395) | 0.3051 | 0.666 | (0.318-1.395) | 0.2792 |
| **mCCI category** |  |  |  |  |  |  |  |  |  |  |  |  |  |  |  |
| *Congestive heart failure* |  |  |  |  |  |  |  |  |  |  |  |  |  |  |  |
| *No* | 38 | 0.19 | 165 | 0.81 | 32 | 0.16 | 171 | 0.84 | 0.3173 | 0.813 | (0.483-1.368) | 0.4330 | 0.795 | (0.457-1.381) | 0.4135 |
| *Yes* | 8 | 0.23 | 27 | 0.77 | 7 | 0.20 | 28 | 0.80 | 0.7389 | 0.844 | (0.253-2.811) | 0.7759 | 1.000 | (0.452-2.211) | 1.0000 |
| *Chronic pulmonary disease* |  |  |  |  |  |  |  |  |  |  |  |  |  |  |  |
| *No* | 0 | 0.00 | 11 | 1.00 | 1 | 0.09 | 10 | 0.91 | NA | NA | NA | NA | NA | NA | NA |
| *Yes* | 46 | 0.20 | 181 | 0.80 | 38 | 0.17 | 189 | 0.83 | 0.2278 | 0.791 | (0.490-1.277) | 0.3363 | 0.775 | (0.470-1.277) | 0.3155 |
| *Mild liver disease* |  |  |  |  |  |  |  |  |  |  |  |  |  |  |  |
| *No* | 29 | 0.18 | 132 | 0.82 | 28 | 0.17 | 133 | 0.83 | 0.8527 | 0.958 | (0.537-1.709) | 0.8845 | 0.954 | (0.505-1.802) | 0.8829 |
| *Yes* | 17 | 0.22 | 60 | 0.78 | 11 | 0.14 | 66 | 0.86 | 0.1336 | 0.588 | (0.250-1.382) | 0.2198 | 0.520 | (0.200-1.355) | 0.1780 |
| *Diabetes with chronic complications* |  |  |  |  |  |  |  |  |  |  |  |  |  |  |  |
| *No* | 36 | 0.18 | 159 | 0.82 | 32 | 0.16 | 163 | 0.84 | 0.5050 | 0.867 | (0.511-1.471) | 0.5952 | 0.852 | (0.484-1.499) | 0.5765 |
| *Yes* | 10 | 0.23 | 33 | 0.77 | 7 | 0.16 | 36 | 0.84 | 0.3173 | 0.642 | (0.209-1.967) | 0.4286 | 0.608 | (0.227-1.624) | 0.3123 |
| *Any malignancy, including lymphoma and leukemia* |  |  |  |  |  |  |  |  |  |  |  |  |  |  |  |
| *No* | 40 | 0.20 | 160 | 0.80 | 33 | 0.17 | 167 | 0.84 | 0.2498 | 0.790 | (0.473-1.321) | 0.3678 | 0.772 | (0.449-1.327) | 0.3474 |
| *Yes* | 6 | 0.16 | 32 | 0.84 | 6 | 0.16 | 32 | 0.84 | 1.0000 | 1.000 | (0.275-3.640) | 1.0000 | 1.000 | (0.512-1.951) | 1.0000 |
| **Index year** |  |  |  |  |  |  |  |  |  |  |  |  |  |  |  |
| *2017* | 19 | 0.14 | 115 | 0.86 | 19 | 0.14 | 115 | 0.86 | 1.0000 | 1.000 | (0.499-2.004) | 1.0000 | 1.000 | (0.469-2.132) | 1.0000 |
| *2018* | 27 | 0.26 | 77 | 0.74 | 20 | 0.19 | 84 | 0.81 | 0.0896 | 0.679 | (0.349-1.323) | 0.2522 | 0.633 | (0.302-1.328) | 0.2239 |

6.4.2 Frequency of OCS-used COPD AE

6.4.2.1. Moderate exacerbation

| **MODERATE COPD EXACERBATION** | **All** | **Pre-BV** | **Post-BV** | **Relative frequency** | **p-value** |
| --- | --- | --- | --- | --- | --- |
|  |  |  |  |  |  |
| ***Number of event per person per year*** | 0.35 | 0.40 | 0.31 | 0.79 | 0.5237 |
| *Total event* | 168 | 94 | 74 |  |  |
| *Patients with event* | 66 | 40 | 26 |  |  |
| *observational period (year)* |  |  |  |  |  |
| *Mean* | 365.04 | 365.08 | 365.00 |  |  |
| *SD* | 0.20 | 0.28 | 0.00 |  |  |
| *Median* | 365 | 365 | 365 |  |  |
| *Min* | 365 | 365 | 365 |  |  |
| *Max* | 366 | 366 | 365 |  |  |
| *P25* | 365 | 365 | 365 |  |  |
| *P75* | 365 | 365 | 365 |  |  |
| **COPD medication during pre and post-BV period** |  |  |  |  |  |
| *ICS/LABA* | 0.35 | 0.44 | 0.26 | 0.58 | 0.3703 |
| *LAMA* | 0.06 | 0.13 | NA | NA | NA |
| *Triple* | 0.58 | 0.54 | 0.63 | 1.17 | 0.7613 |
| *LABA/LAMA* | 0.12 | 0.15 | 0.09 | 0.60 | 0.5123 |
| **Age at index date 1** |  |  |  |  |  |
| *40 to < 65* | 0.66 | 0.67 | 0.66 | 0.98 | 0.9688 |
| *65 to < 75* | 0.25 | 0.26 | 0.25 | 0.96 | 0.9483 |
| *75+* | 0.19 | 0.34 | 0.05 | 0.14 | 0.0407 |
| **Sex** |  |  |  |  |  |
| *Male* | 0.29 | 0.35 | 0.24 | 0.70 | 0.4251 |
| *Female* | 0.58 | 0.58 | 0.58 | 1.00 | 0.9999 |
| **Insurance type at index date2** |  |  |  |  |  |
| *Health insurance* | 0.29 | 0.38 | 0.21 | 0.55 | 0.1734 |
| *Medical aid* | 0.76 | 0.52 | 1.00 | 1.94 | 0.3910 |
| **Hospital type at index date2** |  |  |  |  |  |
| *Tertiary and general hospital* | 0.26 | 0.31 | 0.22 | 0.72 | 0.3917 |
| *Others* | 0.77 | 0.81 | 0.74 | 0.91 | 0.9020 |
| **History of COPD exacerbation** |  |  |  |  |  |
| *none* | 0.16 | 0.14 | 0.18 | 1.28 | 0.7113 |
| *1 moderate* | 0.48 | 0.54 | 0.42 | 0.78 | 0.5792 |
| *≥ 2 moderate OR ≥ 1 severe* | 1.02 | 1.36 | 0.68 | 0.50 | 0.4965 |
| **History of Pneumonia** |  |  |  |  |  |
| *No* | 0.36 | 0.41 | 0.31 | 0.76 | 0.5453 |
| *Yes* | 0.33 | 0.35 | 0.30 | 0.86 | 0.8306 |
| **History of Asthma** |  |  |  |  |  |
| *No* | 0.12 | 0.18 | 0.05 | 0.29 | 0.2498 |
| *Yes* | 0.40 | 0.44 | 0.36 | 0.83 | 0.6271 |
| **mCCI** |  |  |  |  |  |
| *0, 1* | 0.53 | 0.51 | 0.54 | 1.07 | 0.8895 |
| *2 +* | 0.19 | 0.29 | 0.09 | 0.31 | 0.0207 |
| **mCCI category** |  |  |  |  |  |
| *Congestive heart failure* |  |  |  |  |  |
| *No* | 0.38 | 0.41 | 0.35 | 0.85 | 0.6730 |
| *Yes* | 0.19 | 0.29 | 0.09 | 0.30 | 0.1521 |
| *Dementia* |  |  |  |  |  |
| *No* | 0.36 | 0.40 | 0.32 | 0.80 | 0.5440 |
| *Yes* | 0.08 | 0.17 | NA | NA | NA |
| *Chronic pulmonary disease* |  |  |  |  |  |
| *No* | NA | NA | NA | NA | NA |
| *Yes* | 0.37 | 0.41 | 0.33 | 0.79 | 0.5232 |
| *Rheumatologic disease* |  |  |  |  |  |
| *No* | 0.36 | 0.40 | 0.31 | 0.78 | 0.5219 |
| *Yes* | 0.25 | 0.25 | 0.25 | 1.00 | 1.0000 |
| *Mild liver disease* |  |  |  |  |  |
| *No* | 0.43 | 0.45 | 0.41 | 0.92 | 0.8417 |
| *Yes* | 0.19 | 0.29 | 0.10 | 0.36 | 0.0696 |
| *Diabetes with chronic complications* |  |  |  |  |  |
| *No* | 0.38 | 0.41 | 0.35 | 0.86 | 0.7169 |
| *Yes* | 0.24 | 0.35 | 0.14 | 0.40 | 0.2052 |
| *Hemiplegia or paraplegia* |  |  |  |  |  |
| *No* | 0.36 | 0.40 | 0.31 | 0.78 | 0.5035 |
| *Yes* | 0.17 | NA | 0.33 | NA | NA |
| *Renal disease* |  |  |  |  |  |
| *No* | 0.36 | 0.41 | 0.32 | 0.77 | 0.5031 |
| *Yes* | 0.14 | 0.09 | 0.18 | 2.00 | 0.5743 |
| *Any malignancy, including lymphoma and leukemia* |  |  |  |  |  |
| *No* | 0.39 | 0.42 | 0.36 | 0.86 | 0.6959 |
| *Yes* | 0.18 | 0.29 | 0.08 | 0.27 | 0.1321 |
| *Moderate or severe liver disease* |  |  |  |  |  |
| *No* | 0.35 | 0.40 | 0.31 | 0.79 | 0.5237 |
| *Yes* | NA | NA | NA | NA | NA |
| *Metastatic solid tumor* |  |  |  |  |  |
| *No* | 0.35 | 0.40 | 0.31 | 0.79 | 0.5237 |
| *Yes* | NA | NA | NA | NA | NA |
| *HIV* |  |  |  |  |  |
| *No* | 0.35 | 0.40 | 0.31 | 0.79 | 0.5237 |
| *Yes* | NA | NA | NA | NA | NA |
| **Index year** |  |  |  |  |  |
| *2017* | 0.33 | 0.40 | 0.26 | 0.65 | 0.4313 |
| *2018* | 0.38 | 0.38 | 0.38 | 0.98 | 0.9595 |

6.4.2.2 Severe exacerbation

| **SEVERE COPD EXACERBATION** | **All** | **Pre-BV** | **Post-BV** | **Relative frequency** | **p-value** |
| --- | --- | --- | --- | --- | --- |
|  |  |  |  |  |  |
| ***Number of event per person per year*** | 0.12 | 0.15 | 0.10 | 0.64 | 0.3765 |
| *Total event* | 59 | 36 | 23 |  |  |
| *Patients with event* | 29 | 12 | 17 |  |  |
| *observational period (year)* |  |  |  |  |  |
| *Mean* | 365.04 | 365.08 | 365.00 |  |  |
| *SD* | 0.20 | 0.28 | 0.00 |  |  |
| *Median* | 365 | 365 | 365 |  |  |
| *Min* | 365 | 365 | 365 |  |  |
| *Max* | 366 | 366 | 365 |  |  |
| *P25* | 365 | 365 | 365 |  |  |
| *P75* | 365 | 365 | 365 |  |  |
| **COPD medication during pre and post-BV period** |  |  |  |  |  |
| *ICS/LABA* | 0.10 | 0.13 | 0.07 | 0.53 | 0.4847 |
| *LAMA* | NA | NA | NA | NA | NA |
| *Triple* | 0.08 | 0.06 | 0.10 | 1.75 | 0.5493 |
| *LABA/LAMA* | 0.37 | 0.50 | 0.24 | 0.47 | 0.3095 |
| **Age at index date 1** |  |  |  |  |  |
| *40 to < 65* | 0.24 | 0.34 | 0.13 | 0.39 | 0.2891 |
| *65 to < 75* | 0.09 | 0.07 | 0.10 | 1.38 | 0.5791 |
| *75+* | 0.06 | 0.08 | 0.05 | 0.60 | 0.6003 |
| **Sex** |  |  |  |  |  |
| *Male* | 0.14 | 0.17 | 0.12 | 0.69 | 0.4896 |
| *Female* | 0.05 | 0.08 | 0.02 | 0.25 | 0.3131 |
| **Insurance type at index date2** |  |  |  |  |  |
| *Health insurance* | 0.13 | 0.16 | 0.09 | 0.58 | 0.3298 |
| *Medical aid* | 0.11 | 0.10 | 0.13 | 1.33 | 0.7396 |
| **Hospital type at index date2** |  |  |  |  |  |
| *Tertiary and general hospital* | 0.11 | 0.12 | 0.10 | 0.87 | 0.7891 |
| *Others* | 0.19 | 0.31 | 0.07 | 0.23 | 0.3020 |
| **History of COPD exacerbation** |  |  |  |  |  |
| *none* | 0.08 | 0.08 | 0.08 | 1.10 | 0.8639 |
| *1 moderate* | 0.08 | 0.05 | 0.11 | 2.25 | 0.3830 |
| *≥ 2 moderate OR ≥ 1 severe* | 0.57 | 1.00 | 0.14 | 0.14 | 0.1365 |
| **History of Pneumonia** |  |  |  |  |  |
| *No* | 0.08 | 0.07 | 0.10 | 1.42 | 0.5152 |
| *Yes* | 0.24 | 0.38 | 0.10 | 0.25 | 0.1475 |
| **History of Asthma** |  |  |  |  |  |
| *No* | 0.09 | 0.08 | 0.11 | 1.33 | 0.8017 |
| *Yes* | 0.13 | 0.17 | 0.10 | 0.58 | 0.3224 |
| **mCCI** |  |  |  |  |  |
| *0, 1* | 0.12 | 0.15 | 0.09 | 0.65 | 0.5967 |
| *2 +* | 0.13 | 0.16 | 0.10 | 0.63 | 0.4578 |
| **mCCI category** |  |  |  |  |  |
| *Congestive heart failure* |  |  |  |  |  |
| *No* | 0.12 | 0.15 | 0.09 | 0.63 | 0.4411 |
| *Yes* | 0.14 | 0.17 | 0.11 | 0.67 | 0.5742 |
| *Dementia* |  |  |  |  |  |
| *No* | 0.13 | 0.16 | 0.10 | 0.64 | 0.3763 |
| *Yes* | NA | NA | NA | NA | NA |
| *Chronic pulmonary disease* |  |  |  |  |  |
| *No* | 0.05 | NA | 0.09 | NA | NA |
| *Yes* | 0.13 | 0.16 | 0.10 | 0.61 | 0.3387 |
| *Rheumatologic disease* |  |  |  |  |  |
| *No* | 0.12 | 0.15 | 0.09 | 0.60 | 0.3365 |
| *Yes* | 0.13 | 0.08 | 0.17 | 2.00 | 0.5741 |
| *Mild liver disease* |  |  |  |  |  |
| *No* | 0.11 | 0.11 | 0.11 | 0.94 | 0.9341 |
| *Yes* | 0.16 | 0.23 | 0.08 | 0.33 | 0.1738 |
| *Diabetes with chronic complications* |  |  |  |  |  |
| *No* | 0.14 | 0.17 | 0.10 | 0.56 | 0.2985 |
| *Yes* | 0.07 | 0.05 | 0.09 | 2.00 | 0.4168 |
| *Hemiplegia or paraplegia* |  |  |  |  |  |
| *No* | 0.12 | 0.15 | 0.10 | 0.66 | 0.4141 |
| *Yes* | 0.17 | 0.33 | NA | NA | NA |
| *Renal disease* |  |  |  |  |  |
| *No* | 0.13 | 0.15 | 0.10 | 0.66 | 0.4139 |
| *Yes* | 0.05 | 0.09 | NA | NA | NA |
| *Any malignancy, including lymphoma and leukemia* |  |  |  |  |  |
| *No* | 0.13 | 0.17 | 0.10 | 0.56 | 0.2953 |
| *Yes* | 0.08 | 0.05 | 0.11 | 2.00 | 0.4701 |
| *Moderate or severe liver disease* |  |  |  |  |  |
| *No* | 0.12 | 0.15 | 0.10 | 0.64 | 0.3765 |
| *Yes* | NA | NA | NA | NA | NA |
| *Metastatic solid tumor* |  |  |  |  |  |
| *No* | 0.12 | 0.15 | 0.10 | 0.64 | 0.3765 |
| *Yes* | NA | NA | NA | NA | NA |
| *HIV* |  |  |  |  |  |
| *No* | 0.12 | 0.15 | 0.10 | 0.64 | 0.3765 |
| *Yes* | NA | NA | NA | NA | NA |
| **Index year** |  |  |  |  |  |
| *2017* | 0.03 | 0.02 | 0.04 | 2.00 | 0.3932 |
| *2018* | 0.24 | 0.32 | 0.16 | 0.52 | 0.2577 |

6.4.2.3 Moderate-to-severe exacerbation

| **MODERATE TO SEVERE COPD EXACERBATION** | **All** | **Pre-BV** | **Post-BV** | **Relative frequency** | **p-value** |
| --- | --- | --- | --- | --- | --- |
|  |  |  |  |  |  |
| ***Number of event per person per year*** | 0.48 | 0.55 | 0.41 | 0.75 | 0.3541 |
| *Total event* | 227 | 130 | 97 |  |  |
| *Patients with event* | 85 | 46 | 39 |  |  |
| *observational period (year)* |  |  |  |  |  |
| *Mean* | 365.04 | 365.08 | 365.00 |  |  |
| *SD* | 0.20 | 0.28 | 0.00 |  |  |
| *Median* | 365 | 365 | 365 |  |  |
| *Min* | 365 | 365 | 365 |  |  |
| *Max* | 366 | 366 | 365 |  |  |
| *P25* | 365 | 365 | 365 |  |  |
| *P75* | 365 | 365 | 365 |  |  |
| **COPD medication during pre and post-BV period** |  |  |  |  |  |
| *ICS/LABA* | 0.45 | 0.58 | 0.33 | 0.57 | 0.2810 |
| *LAMA* | 0.06 | 0.13 | NA | NA | NA |
| *Triple* | 0.66 | 0.60 | 0.73 | 1.23 | 0.6658 |
| *LABA/LAMA* | 0.49 | 0.65 | 0.32 | 0.50 | 0.2760 |
| **Age at index date 1** |  |  |  |  |  |
| *40 to < 65* | 0.90 | 1.02 | 0.79 | 0.78 | 0.6130 |
| *65 to < 75* | 0.34 | 0.33 | 0.35 | 1.06 | 0.9010 |
| *75+* | 0.26 | 0.42 | 0.10 | 0.23 | 0.0439 |
| **Sex** |  |  |  |  |  |
| *Male* | 0.44 | 0.52 | 0.36 | 0.69 | 0.3138 |
| *Female* | 0.64 | 0.67 | 0.60 | 0.91 | 0.8751 |
| **Insurance type at index date2** |  |  |  |  |  |
| *Health insurance* | 0.42 | 0.54 | 0.30 | 0.56 | 0.1062 |
| *Medical aid* | 0.87 | 0.61 | 1.13 | 1.84 | 0.3719 |
| **Hospital type at index date2** |  |  |  |  |  |
| *Tertiary and general hospital* | 0.37 | 0.42 | 0.32 | 0.76 | 0.3980 |
| *Others* | 0.96 | 1.12 | 0.81 | 0.72 | 0.6251 |
| **History of COPD exacerbation** |  |  |  |  |  |
| *none* | 0.24 | 0.21 | 0.26 | 1.21 | 0.6919 |
| *1 moderate* | 0.56 | 0.59 | 0.53 | 0.90 | 0.7940 |
| *≥ 2 moderate OR ≥ 1 severe* | 1.59 | 2.37 | 0.82 | 0.35 | 0.2019 |
| **History of Pneumonia** |  |  |  |  |  |
| *No* | 0.45 | 0.48 | 0.41 | 0.86 | 0.6825 |
| *Yes* | 0.56 | 0.73 | 0.40 | 0.54 | 0.3029 |
| **History of Asthma** |  |  |  |  |  |
| *No* | 0.21 | 0.26 | 0.16 | 0.60 | 0.4782 |
| *Yes* | 0.53 | 0.60 | 0.46 | 0.76 | 0.4082 |
| **mCCI** |  |  |  |  |  |
| *0, 1* | 0.65 | 0.66 | 0.64 | 0.97 | 0.9502 |
| *2 +* | 0.32 | 0.44 | 0.19 | 0.43 | 0.0331 |
| **mCCI category** |  |  |  |  |  |
| *Congestive heart failure* |  |  |  |  |  |
| *No* | 0.50 | 0.56 | 0.44 | 0.79 | 0.4921 |
| *Yes* | 0.33 | 0.46 | 0.20 | 0.44 | 0.1492 |
| *Dementia* |  |  |  |  |  |
| *No* | 0.49 | 0.56 | 0.42 | 0.75 | 0.3682 |
| *Yes* | 0.08 | 0.17 | NA | NA | NA |
| *Chronic pulmonary disease* |  |  |  |  |  |
| *No* | 0.05 | 0.00 | 0.09 | NA | 0.9941 |
| *Yes* | 0.50 | 0.57 | 0.42 | 0.74 | 0.3392 |
| *Rheumatologic disease* |  |  |  |  |  |
| *No* | 0.48 | 0.56 | 0.41 | 0.73 | 0.3380 |
| *Yes* | 0.38 | 0.33 | 0.42 | 1.25 | 0.8044 |
| *Mild liver disease* |  |  |  |  |  |
| *No* | 0.54 | 0.56 | 0.52 | 0.92 | 0.8320 |
| *Yes* | 0.35 | 0.52 | 0.18 | 0.35 | 0.0347 |
| *Diabetes with chronic complications* |  |  |  |  |  |
| *No* | 0.51 | 0.58 | 0.45 | 0.77 | 0.4533 |
| *Yes* | 0.31 | 0.40 | 0.23 | 0.59 | 0.3740 |
| *Hemiplegia or paraplegia* |  |  |  |  |  |
| *No* | 0.48 | 0.55 | 0.41 | 0.74 | 0.3540 |
| *Yes* | 0.33 | NA | 0.33 | NA | 1.0000 |
| *Renal disease* |  |  |  |  |  |
| *No* | 0.49 | 0.56 | 0.42 | 0.74 | 0.3534 |
| *Yes* | 0.18 | 0.18 | 0.18 | 1.00 | 1.0000 |
| *Any malignancy, including lymphoma and leukemia* |  |  |  |  |  |
| *No* | 0.52 | 0.59 | 0.45 | 0.77 | 0.4406 |
| *Yes* | 0.26 | 0.34 | 0.18 | 0.54 | 0.3168 |
| *Moderate or severe liver disease* |  |  |  |  |  |
| *No* | 0.48 | 0.55 | 0.41 | 0.75 | 0.3541 |
| *Yes* | NA | NA | NA | NA | NA |
| *Metastatic solid tumor* |  |  |  |  |  |
| *No* | 0.48 | 0.55 | 0.41 | 0.75 | 0.3541 |
| *Yes* | NA | NA | NA | NA | NA |
| *HIV* |  |  |  |  |  |
| *No* | 0.48 | 0.55 | 0.41 | 0.75 | 0.3541 |
| *Yes* | NA | NA | NA | NA | NA |
| **Index year** |  |  |  |  |  |
| *2017* | 0.37 | 0.43 | 0.31 | 0.72 | 0.5121 |
| *2018* | 0.62 | 0.70 | 0.54 | 0.77 | 0.5118 |

6.4.3. Incidence rate of OCS-used COPD AE

6.4.3.1 Moderate exacerbation

| **MODERATE COPD EXACERBATION** | **All** | **Pre-BV** | **Post-BV** | **p-value** | **IRR** | **IRR-pvalue** |
| --- | --- | --- | --- | --- | --- | --- |
|  |  |  |  |  |  |  |
| ***Incidence rate per 1,000 PYs*** | 151.37 | 186.28 | 117.50 | 0.0641 | 0.63 | 0.0673 |
| *Patients with event* | 66 | 40 | 26 |  |  |  |
| *Sum of person years (PYs)* | 436.01 | 214.73 | 221.28 |  |  |  |
| *Time to event (KM estimated)* |  |  |  |  |  |  |
| *Median* | NA | NA | NA |  |  |  |
| *SE* | NA | NA | NA |  |  |  |
| *Time to event (descriptive)* |  |  |  |  |  |  |
| *Mean* | 148.23 | 158.50 | 132.42 |  |  |  |
| *SD* | 119.04 | 125.93 | 108.06 |  |  |  |
| *Median* | 135.00 | 139.00 | 130.50 |  |  |  |
| *Min* | 2.00 | 2.00 | 5.00 |  |  |  |
| *Max* | 364.00 | 364.00 | 331.00 |  |  |  |
| *P25* | 33.00 | 34.00 | 32.00 |  |  |  |
| *P75* | 252.00 | 261.50 | 213.00 |  |  |  |
| **COPD medication during pre and post-BV period** |  |  |  |  |  |  |
| *ICS/LABA* | 139.60 | 165.77 | 114.08 | 0.3178 | 0.69 | 0.3217 |
| *LAMA* | 66.47 | 141.83 | NA | NA | NA | NA |
| *Triple* | 228.32 | 270.34 | 186.21 | 0.3370 | 0.69 | 0.3412 |
| *LABA/LAMA* | 107.89 | 125.63 | 90.80 | 0.6690 | 0.72 | 0.6707 |
| **Age at index date 1** |  |  |  |  |  |  |
| *40 to < 65* | 219.21 | 252.83 | 185.57 | 0.4332 | 0.73 | 0.4359 |
| *65 to < 75* | 134.38 | 140.77 | 128.12 | 0.8068 | 0.91 | 0.8069 |
| *75+* | 111.60 | 196.62 | 33.04 | 0.0059 | 0.17 | 0.0203 |
| **Sex** |  |  |  |  |  |  |
| *Male* | 136.60 | 155.29 | 118.30 | 0.3478 | 0.76 | 0.3498 |
| *Female* | 212.72 | 318.21 | 114.25 | 0.0391 | 0.36 | 0.0516 |
| **Insurance type at index date2** |  |  |  |  |  |  |
| *Health insurance* | 138.47 | 187.34 | 91.87 | 0.0115 | 0.49 | 0.0140 |
| *Medical aid* | 244.11 | 179.24 | 315.48 | 0.3139 | 1.76 | 0.3213 |
| **Hospital type at index date2** |  |  |  |  |  |  |
| *Tertiary and general hospital* | 137.94 | 167.85 | 108.84 | 0.1294 | 0.65 | 0.1335 |
| *Others* | 217.61 | 277.82 | 159.87 | 0.2764 | 0.58 | 0.2846 |
| **History of COPD exacerbation** |  |  |  |  |  |  |
| *none* | 84.07 | 96.59 | 71.68 | 0.4965 | 0.74 | 0.4987 |
| *1 moderate* | 219.75 | 256.99 | 183.64 | 0.3370 | 0.71 | 0.3400 |
| *≥ 2 moderate OR ≥ 1 severe* | 333.00 | 543.62 | 154.01 | 0.0406 | 0.28 | 0.0585 |
| **History of Pneumonia** |  |  |  |  |  |  |
| *No* | 146.82 | 177.88 | 116.77 | 0.1527 | 0.66 | 0.1568 |
| *Yes* | 163.94 | 209.35 | 119.51 | 0.2298 | 0.57 | 0.2385 |
| **History of Asthma** |  |  |  |  |  |  |
| *No* | 83.42 | 114.47 | 54.08 | 0.3717 | 0.47 | 0.3866 |
| *Yes* | 164.80 | 200.24 | 130.22 | 0.0989 | 0.65 | 0.1025 |
| **mCCI** |  |  |  |  |  |  |
| *0, 1* | 189.53 | 216.57 | 163.17 | 0.3786 | 0.75 | 0.3807 |
| *2 +* | 117.27 | 159.09 | 76.86 | 0.0662 | 0.48 | 0.0747 |
| **mCCI category** |  |  |  |  |  |  |
| *Congestive heart failure* |  |  |  |  |  |  |
| *No* | 156.79 | 191.71 | 122.76 | 0.0930 | 0.64 | 0.0968 |
| *Yes* | 121.04 | 155.47 | 88.42 | 0.4317 | 0.57 | 0.4397 |
| *Dementia* |  |  |  |  |  |  |
| *No* | 153.29 | 186.83 | 120.77 | 0.0815 | 0.65 | 0.0848 |
| *Yes* | 83.51 | 167.24 | NA | NA | NA | NA |
| *Chronic pulmonary disease* |  |  |  |  |  |  |
| *No* | NA | NA | NA | NA | NAA | NA |
| *Yes* | 159.40 | 196.31 | 123.64 | 0.0632 | 0.63 | 0.0665 |
| *Rheumatologic disease* |  |  |  |  |  |  |
| *No* | 149.78 | 191.91 | 109.16 | 0.0289 | 0.57 | 0.0319 |
| *Yes* | 181.20 | 86.92 | 283.80 | 0.2688 | 3.26 | 0.3055 |
| *Mild liver disease* |  |  |  |  |  |  |
| *No* | 161.34 | 195.52 | 128.28 | 0.1522 | 0.66 | 0.1562 |
| *Yes* | 131.31 | 167.78 | 95.67 | 0.2289 | 0.57 | 0.2375 |
| *Diabetes with chronic complications* |  |  |  |  |  |  |
| *No* | 148.29 | 180.53 | 116.57 | 0.1152 | 0.65 | 0.1194 |
| *Yes* | 165.38 | 213.49 | 121.55 | 0.3157 | 0.57 | 0.3231 |
| *Hemiplegia or paraplegia* |  |  |  |  |  |  |
| *No* | 151.10 | 188.91 | 114.45 | 0.0461 | 0.61 | 0.0493 |
| *Yes* | 171.64 | NA | 352.56 | NA | NA | NA |
| *Renal disease* |  |  |  |  |  |  |
| *No* | 151.83 | 190.91 | 113.93 | 0.0434 | 0.60 | 0.0466 |
| *Yes* | 142.38 | 95.79 | 188.13 | 0.5705 | 1.96 | 0.5816 |
| *Any malignancy, including lymphoma and leukemia* |  |  |  |  |  |  |
| *No* | 159.57 | 195.45 | 124.74 | 0.0906 | 0.64 | 0.0943 |
| *Yes* | 110.29 | 140.25 | 81.32 | 0.4479 | 0.58 | 0.4555 |
| *Moderate or severe liver disease* |  |  |  |  |  |  |
| *No* | 152.07 | 187.15 | 118.03 | 0.0640 | 0.63 | 0.0673 |
| *Yes* | NA | NA | NA | NA | NA | NA |
| *Metastatic solid tumor* |  |  |  |  |  |  |
| *No* | 152.07 | 187.15 | 118.03 | 0.0640 | 0.63 | 0.0673 |
| *Yes* | NA | NA | NA | NA | NA | NA |
| *HIV* |  |  |  |  |  |  |
| *No* | 151.37 | 186.28 | 117.50 | 0.0641 | 0.63 | 0.0673 |
| *Yes* | NA | NA | NA | NA | NA | NA |
| **Index year** |  |  |  |  |  |  |
| *2017* | 134.74 | 149.85 | 120.20 | 0.5274 | 0.80 | 0.5283 |
| *2018* | 172.69 | 232.54 | 114.00 | 0.0466 | 0.49 | 0.0535 |

6.4.3.2 Severe exacerbation

| **SEVERE COPD EXACERBATION** | **All** | **Pre-BV** | **Post-BV** | **p-value** | **IRR** | **IRR-pvalue** |
| --- | --- | --- | --- | --- | --- | --- |
|  |  |  |  |  |  |  |
| ***Incidence rate per 1,000 PYs*** | 62.95 | 52.15 | 73.72 | 0.3548 | 1.41 | 0.3584 |
| *Patients with event* | 29 | 12 | 17 |  |  |  |
| *Sum of person years (PYs)* | 460.72 | 230.13 | 230.59 |  |  |  |
| *Time to event (KM estimated)* |  |  |  |  |  |  |
| *Median* | NA | NA | NA |  |  |  |
| *SE* | NA | NA | NA |  |  |  |
| *Time to event (descriptive)* |  |  |  |  |  |  |
| *Mean* | 183.76 | 147.50 | 209.35 |  |  |  |
| *SD* | 109.88 | 120.47 | 97.31 |  |  |  |
| *Median* | 181.00 | 104.50 | 213.00 |  |  |  |
| *Min* | 7.00 | 11.00 | 7.00 |  |  |  |
| *Max* | 364.00 | 364.00 | 345.00 |  |  |  |
| *P25* | 100.00 | 56.00 | 141.00 |  |  |  |
| *P75* | 283.00 | 254.00 | 286.00 |  |  |  |
| **COPD medication during pre and post-BV period** |  |  |  |  |  |  |
| *ICS/LABA* | 50.02 | 36.43 | 63.56 | 0.3652 | 1.74 | 0.3745 |
| *LAMA* | NA | NA | NA | NA | NA | NA |
| *Triple* | 45.59 | 45.27 | 45.92 | 0.9861 | 1.01 | 0.9861 |
| *LABA/LAMA* | 195.83 | 165.93 | 224.75 | 0.6020 | 1.35 | 0.6043 |
| **Age at index date 1** |  |  |  |  |  |  |
| *40 to < 65* | 70.39 | 78.41 | 62.41 | 0.7328 | 0.80 | 0.7336 |
| *65 to < 75* | 70.89 | 47.07 | 94.91 | 0.1872 | 2.02 | 0.2005 |
| *75+* | 41.23 | 33.25 | 49.08 | 0.6667 | 1.48 | 0.6698 |
| **Sex** |  |  |  |  |  |  |
| *Male* | 70.91 | 54.46 | 87.41 | 0.2341 | 1.61 | 0.2405 |
| *Female* | 31.89 | 43.00 | 21.03 | 0.5472 | 0.49 | 0.5592 |
| **Insurance type at index date2** |  |  |  |  |  |  |
| *Health insurance* | 57.42 | 50.06 | 64.73 | 0.5396 | 1.29 | 0.5413 |
| *Medical aid* | 99.76 | 65.83 | 134.41 | 0.3956 | 2.04 | 0.4098 |
| **Hospital type at index date2** |  |  |  |  |  |  |
| *Tertiary and general hospital* | 63.10 | 47.30 | 78.90 | 0.2175 | 1.67 | 0.2249 |
| *Others* | 62.23 | 75.26 | 49.40 | 0.6412 | 0.66 | 0.6447 |
| **History of COPD exacerbation** |  |  |  |  |  |  |
| *none* | 58.93 | 47.12 | 70.76 | 0.4358 | 1.50 | 0.4405 |
| *1 moderate* | 47.92 | 23.84 | 72.26 | 0.1441 | 3.03 | 0.1744 |
| *≥ 2 moderate OR ≥ 1 severe* | 152.80 | 211.59 | 98.23 | 0.3605 | 0.46 | 0.3756 |
| **History of Pneumonia** |  |  |  |  |  |  |
| *No* | 55.57 | 35.07 | 76.11 | 0.1034 | 2.17 | 0.1164 |
| *Yes* | 84.17 | 101.65 | 66.91 | 0.5127 | 0.66 | 0.5171 |
| **History of Asthma** |  |  |  |  |  |  |
| *No* | 54.25 | 26.81 | 82.32 | 0.2958 | 3.07 | 0.3313 |
| *Yes* | 64.60 | 57.04 | 72.11 | 0.5594 | 1.26 | 0.5608 |
| **mCCI** |  |  |  |  |  |  |
| *0, 1* | 48.73 | 35.50 | 61.92 | 0.3655 | 1.74 | 0.3747 |
| *2 +* | 76.60 | 68.12 | 85.07 | 0.6383 | 1.25 | 0.6393 |
| **mCCI category** |  |  |  |  |  |  |
| *Congestive heart failure* |  |  |  |  |  |  |
| *No* | 53.16 | 40.42 | 65.94 | 0.2691 | 1.63 | 0.2761 |
| *Yes* | 121.81 | 124.13 | 119.58 | 0.9579 | 0.96 | 0.9579 |
| *Dementia* |  |  |  |  |  |  |
| *No* | 64.63 | 53.54 | 75.69 | 0.3547 | 1.41 | 0.3584 |
| *Yes* | NA | NA | NA | NA | NA | NA |
| *Chronic pulmonary disease* |  |  |  |  |  |  |
| *No* | 46.01 | NA | 92.87 | NA | NA | NA |
| *Yes* | 63.78 | 54.75 | 72.79 | 0.4537 | 1.33 | 0.4560 |
| *Rheumatologic disease* |  |  |  |  |  |  |
| *No* | 59.42 | 50.39 | 68.42 | 0.4381 | 1.36 | 0.4408 |
| *Yes* | 129.40 | 84.63 | 175.94 | 0.5378 | 2.08 | 0.5501 |
| *Mild liver disease* |  |  |  |  |  |  |
| *No* | 54.18 | 31.74 | 76.82 | 0.0818 | 2.42 | 0.0968 |
| *Yes* | 81.65 | 96.43 | 67.23 | 0.5348 | 0.70 | 0.5378 |
| *Diabetes with chronic complications* |  |  |  |  |  |  |
| *No* | 60.97 | 53.12 | 68.80 | 0.5369 | 1.30 | 0.5387 |
| *Yes* | 71.85 | 47.76 | 96.08 | 0.4057 | 2.01 | 0.4196 |
| *Hemiplegia or paraplegia* |  |  |  |  |  |  |
| *No* | 61.47 | 48.27 | 74.69 | 0.2536 | 1.55 | 0.2592 |
| *Yes* | 190.93 | 446.52 | NA | NA | NA | NA |
| *Renal disease* |  |  |  |  |  |  |
| *No* | 63.71 | 50.02 | 77.41 | 0.2534 | 1.55 | 0.2590 |
| *Yes* | 47.16 | 97.92 | NA | NA | NA | NA |
| *Any malignancy, including lymphoma and leukemia* |  |  |  |  |  |  |
| *No* | 62.12 | 51.95 | 72.22 | 0.4232 | 1.39 | 0.4264 |
| *Yes* | 67.23 | 53.12 | 81.68 | 0.6339 | 1.54 | 0.6375 |
| *Moderate or severe liver disease* |  |  |  |  |  |  |
| *No* | 63.22 | 52.37 | 74.04 | 0.3548 | 1.41 | 0.3584 |
| *Yes* | NA | NA | NA | NA | NA | NA |
| *Metastatic solid tumor* |  |  |  |  |  |  |
| *No* | 63.22 | 52.37 | 74.04 | 0.3548 | 1.41 | 0.3584 |
| *Yes* | NA | NA | NA | NA | NA | NA |
| *HIV* |  |  |  |  |  |  |
| *No* | 62.95 | 52.15 | 73.72 | 0.3548 | 1.41 | 0.3584 |
| *Yes* | NA | NA | NA | NA | NA | NA |
| **Index year** |  |  |  |  |  |  |
| *2017* | 30.15 | 15.03 | 45.37 | 0.1455 | 3.02 | 0.1760 |
| *2018* | 107.47 | 103.04 | 111.84 | 0.8511 | 1.09 | 0.8512 |

6.4.3.3. Moderate-to-severe exacerbation

| **MODERATE TO SEVERE COPD EXACERBATION** | **All** | **Pre-BV** | **Post-BV** | **p-value** | **IRR** | **IRR-pvalue** |
| --- | --- | --- | --- | --- | --- | --- |
|  |  |  |  |  |  |  |
| ***Incidence rate per 1,000 PYs*** | 200.22 | 219.79 | 181.19 | 0.3741 | 0.82 | 0.3750 |
| *Patients with event* | 85 | 46 | 39 |  |  |  |
| *Sum of person years (PYs)* | 424.53 | 209.29 | 215.24 |  |  |  |
| *Time to event (KM estimated)* |  |  |  |  |  |  |
| *Median* | NA | NA | NA |  |  |  |
| *SE* | NA | NA | NA |  |  |  |
| *Time to event (descriptive)* |  |  |  |  |  |  |
| *Mean* | 147.27 | 142.11 | 153.36 |  |  |  |
| *SD* | 114.51 | 120.10 | 108.79 |  |  |  |
| *Median* | 132.00 | 112.00 | 155.00 |  |  |  |
| *Min* | 2.00 | 2.00 | 5.00 |  |  |  |
| *Max* | 364.00 | 364.00 | 331.00 |  |  |  |
| *P25* | 33.00 | 31.00 | 50.00 |  |  |  |
| *P75* | 254.00 | 252.00 | 267.00 |  |  |  |
| **COPD medication during pre and post-BV period** |  |  |  |  |  |  |
| *ICS/LABA* | 171.95 | 189.10 | 155.22 | 0.5598 | 0.82 | 0.5606 |
| *LAMA* | 66.47 | 141.83 | NA | NA | NA | NA |
| *Triple* | 256.40 | 287.95 | 224.27 | 0.4957 | 0.78 | 0.4976 |
| *LABA/LAMA* | 288.96 | 244.57 | 331.02 | 0.5362 | 1.35 | 0.5391 |
| **Age at index date 1** |  |  |  |  |  |  |
| *40 to < 65* | 252.81 | 259.61 | 245.91 | 0.8840 | 0.95 | 0.8841 |
| *65 to < 75* | 204.53 | 195.82 | 213.11 | 0.7892 | 1.09 | 0.7893 |
| *75+* | 140.05 | 220.26 | 66.93 | 0.0259 | 0.30 | 0.0391 |
| **Sex** |  |  |  |  |  |  |
| *Male* | 196.99 | 195.68 | 198.28 | 0.9568 | 1.01 | 0.9568 |
| *Female* | 213.26 | 319.88 | 114.25 | 0.0381 | 0.36 | 0.0504 |
| **Insurance type at index date2** |  |  |  |  |  |  |
| *Health insurance* | 185.40 | 220.34 | 152.13 | 0.1261 | 0.69 | 0.1288 |
| *Medical aid* | 305.57 | 216.21 | 406.33 | 0.2133 | 1.88 | 0.2218 |
| **Hospital type at index date2** |  |  |  |  |  |  |
| *Tertiary and general hospital* | 186.57 | 200.50 | 173.00 | 0.5493 | 0.86 | 0.5498 |
| *Others* | 268.48 | 316.81 | 221.94 | 0.4406 | 0.70 | 0.4437 |
| **History of COPD exacerbation** |  |  |  |  |  |  |
| *none* | 131.03 | 131.42 | 130.64 | 0.9864 | 0.99 | 0.9864 |
| *1 moderate* | 263.52 | 287.32 | 240.31 | 0.5774 | 0.84 | 0.5780 |
| *≥ 2 moderate OR ≥ 1 severe* | 433.31 | 622.70 | 280.01 | 0.1416 | 0.45 | 0.1519 |
| **History of Pneumonia** |  |  |  |  |  |  |
| *No* | 193.86 | 213.15 | 175.17 | 0.4442 | 0.82 | 0.4450 |
| *Yes* | 218.46 | 238.68 | 198.57 | 0.6528 | 0.83 | 0.6534 |
| **History of Asthma** |  |  |  |  |  |  |
| *No* | 143.24 | 145.51 | 141.03 | 0.9606 | 0.97 | 0.9606 |
| *Yes* | 211.44 | 234.39 | 189.11 | 0.3537 | 0.81 | 0.3548 |
| **mCCI** |  |  |  |  |  |  |
| *0, 1* | 222.99 | 230.56 | 215.60 | 0.8220 | 0.94 | 0.8220 |
| *2 +* | 179.59 | 209.99 | 150.18 | 0.2917 | 0.72 | 0.2946 |
| **mCCI category** |  |  |  |  |  |  |
| *Congestive heart failure* |  |  |  |  |  |  |
| *No* | 193.14 | 211.59 | 175.02 | 0.4281 | 0.83 | 0.4291 |
| *Yes* | 241.55 | 269.41 | 216.02 | 0.6691 | 0.80 | 0.6695 |
| *Dementia* |  |  |  |  |  |  |
| *No* | 203.61 | 221.34 | 186.39 | 0.4314 | 0.84 | 0.4321 |
| *Yes* | 83.51 | 167.24 | NA | NA | NA | NA |
| *Chronic pulmonary disease* |  |  |  |  |  |  |
| *No* | 46.01 | NA | 92.87 | NA | NA | NA |
| *Yes* | 208.54 | 231.95 | 185.85 | 0.3109 | 0.80 | 0.3121 |
| *Rheumatologic disease* |  |  |  |  |  |  |
| *No* | 196.13 | 222.30 | 170.84 | 0.2433 | 0.77 | 0.2449 |
| *Yes* | 276.22 | 176.07 | 386.00 | 0.3493 | 2.19 | 0.3647 |
| *Mild liver disease* |  |  |  |  |  |  |
| *No* | 199.58 | 205.12 | 194.14 | 0.8355 | 0.95 | 0.8355 |
| *Yes* | 201.55 | 250.34 | 154.90 | 0.2090 | 0.62 | 0.2147 |
| *Diabetes with chronic complications* |  |  |  |  |  |  |
| *No* | 195.54 | 208.34 | 182.89 | 0.5914 | 0.88 | 0.5917 |
| *Yes* | 221.46 | 274.01 | 173.83 | 0.3514 | 0.63 | 0.3558 |
| *Hemiplegia or paraplegia* |  |  |  |  |  |  |
| *No* | 197.88 | 217.34 | 178.91 | 0.3761 | 0.82 | 0.3771 |
| *Yes* | 394.01 | 446.52 | 352.56 | 0.8675 | NA | 0.8673 |
| *Renal disease* |  |  |  |  |  |  |
| *No* | 200.39 | 220.44 | 180.83 | 0.3736 | 0.82 | 0.3746 |
| *Yes* | 196.85 | 206.41 | 188.13 | 0.9261 | 0.91 | 0.9261 |
| *Any malignancy, including lymphoma and leukemia* |  |  |  |  |  |  |
| *No* | 206.53 | 230.06 | 183.75 | 0.3379 | 0.80 | 0.3391 |
| *Yes* | 168.86 | 169.39 | 168.33 | 0.9914 | 0.99 | 0.9914 |
| *Moderate or severe liver disease* |  |  |  |  |  |  |
| *No* | 201.17 | 220.85 | 182.04 | 0.3738 | 0.82 | 0.3747 |
| *Yes* | NA | NA | NA | NA | NA | NA |
| *Metastatic solid tumor* |  |  |  |  |  |  |
| *No* | 201.17 | 220.85 | 182.04 | 0.3738 | 0.82 | 0.3747 |
| *Yes* | NA | NA | NA | NA | NA | NA |
| *HIV* |  |  |  |  |  |  |
| *No* | 200.22 | 219.79 | 181.19 | 0.3741 | 0.82 | 0.3750 |
| *Yes* | NA | NA | NA | NA | NA | NA |
| **Index year** |  |  |  |  |  |  |
| *2017* | 156.02 | 158.37 | 153.74 | 0.9271 | 0.97 | 0.9271 |
| *2018* | 259.72 | 302.31 | 218.22 | 0.2664 | 0.72 | 0.2692 |

6.4.4. Time to first OCS-used COPD AE

6.4.4.1. Moderate exacerbation


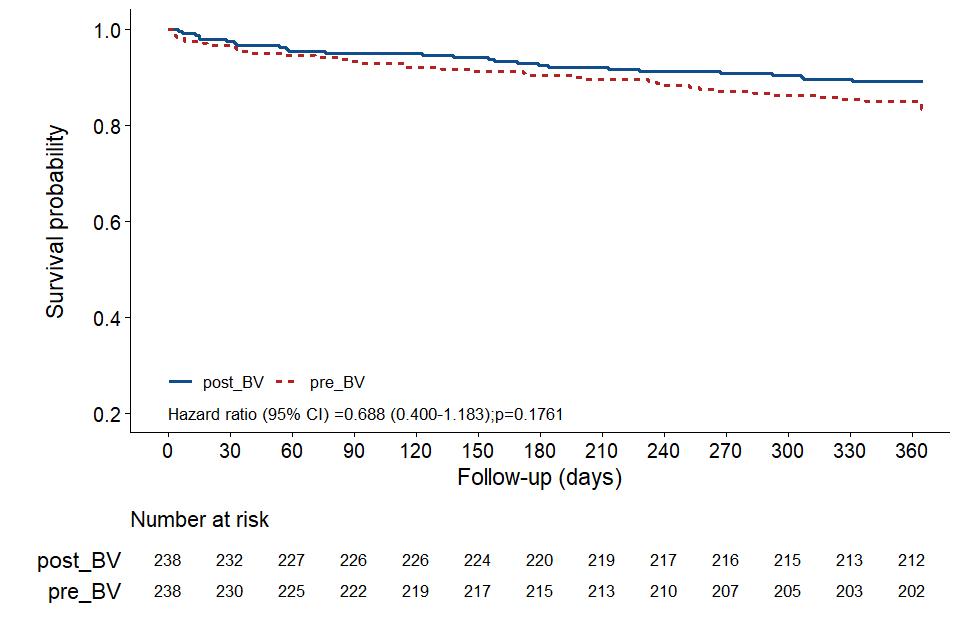


6.4.4.2. Severe exacerbation


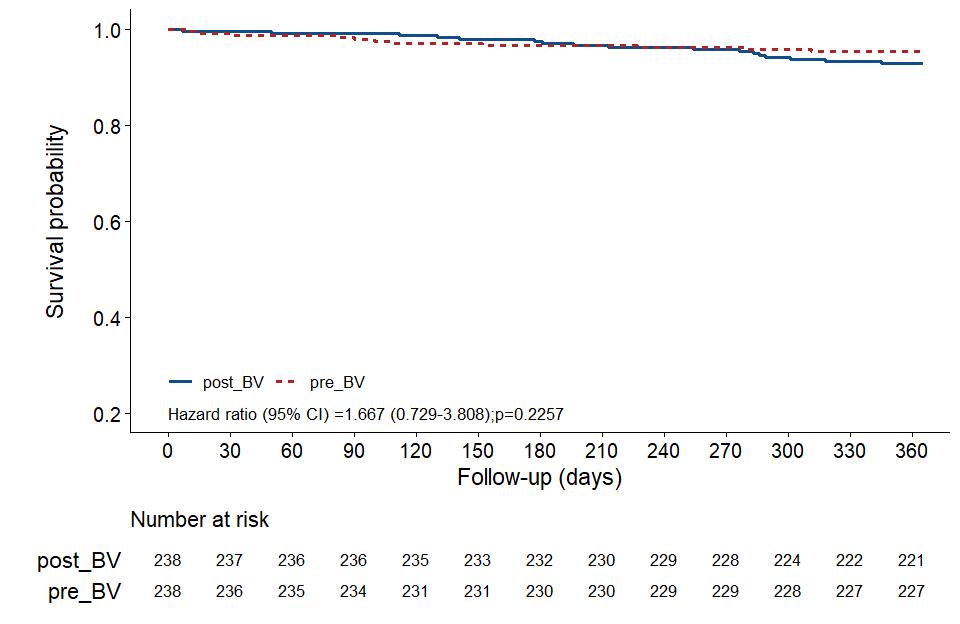


6.3.4.3. Moderate-to-severe exacerbation


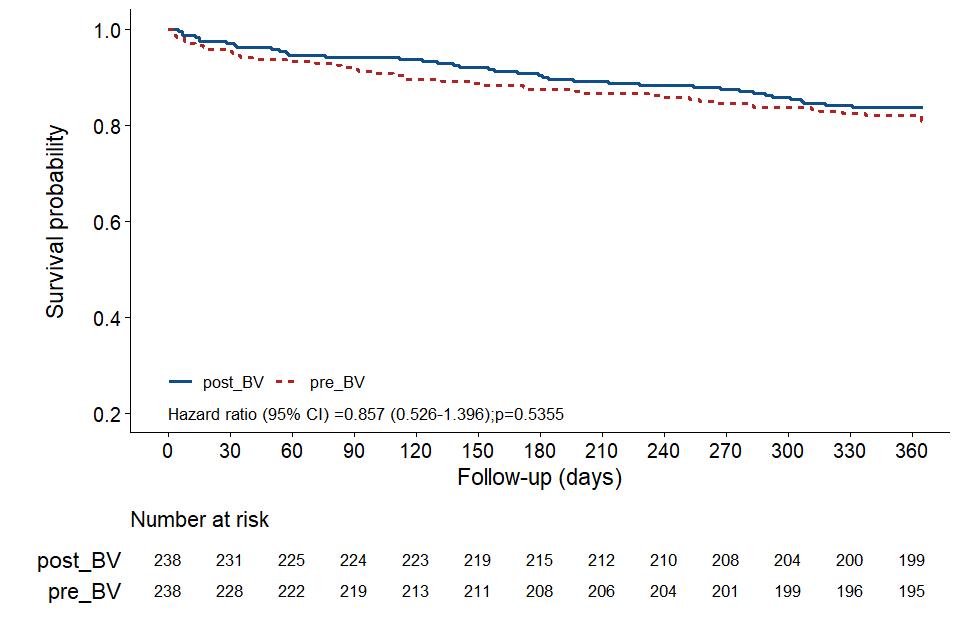


6.5. Subgroup analysis of antibiotics & OCS-used COPD AE

6.5.1. Odd ratio of antibiotics & OCS-used COPD AE

6.5.1.1 Moderate exacerbation

| **MODERATE COPD EXACERBATION** |  | | | | | | | | | | | | | | |
| --- | --- | --- | --- | --- | --- | --- | --- | --- | --- | --- | --- | --- | --- | --- | --- |
|  | **Pre-BV** | | | | **Post-BV** | | | | **Mcnemar p-value** | **Crude OR** | | | **Adjusted OR** | | |
|  | **Event** | | **No Event** | | **Event** | | **No Event** | |  |  |  |  |  |  |  |
|  | n | % | n | % | n | % | n | % |  | OR | 95% CI | p-value | OR | 95% CI | p-value |
| **Overall** | 44 | 0.18 | 194 | 0.82 | 36 | 0.15 | 202 | 0.85 | 0.1824 | 0.786 | (0.483-1.278) | 0.3295 | 0.754 | (0.431-1.318) | 0.3194 |
| **COPD medication during pre and post-BV period** |  |  |  |  |  |  |  |  |  |  |  |  |  |  |  |
| *ICS/LABA* | 12 | 0.11 | 101 | 0.89 | 17 | 0.15 | 96 | 0.85 | 0.1655 | 1.490 | (0.668-3.324) | 0.3264 | 1.423 | (0.719-2.815) | 0.3080 |
| *LAMA* | 1 | 0.04 | 23 | 0.96 | 1 | 0.04 | 23 | 0.96 | NA | 1.000 | (0.047-21.173) | 1.0000 | 1.000 | (0.982-1.018) | 1.0000 |
| *Triple* | 24 | 0.36 | 43 | 0.64 | 13 | 0.19 | 54 | 0.81 | 0.0045 | 0.431 | (0.193-0.965) | 0.0410 | 0.359 | (0.137-0.940) | 0.0374 |
| *LABA/LAMA* | 7 | 0.21 | 27 | 0.79 | 5 | 0.15 | 29 | 0.85 | 0.4795 | 0.665 | (0.176-2.513) | 0.5367 | 0.626 | (0.214-1.830) | 0.3811 |
| **Age at index date 1** |  |  |  |  |  |  |  |  |  |  |  |  |  |  |  |
| *40 to < 65* | 15 | 0.22 | 52 | 0.78 | 11 | 0.16 | 56 | 0.84 | 0.2059 | 0.681 | (0.280-1.654) | 0.3905 | 0.643 | (0.293-1.411) | 0.2655 |
| *65 to < 75* | 21 | 0.19 | 88 | 0.81 | 20 | 0.18 | 89 | 0.82 | 0.8185 | 0.942 | (0.472-1.878) | 0.8634 | 0.926 | (0.362-2.367) | 0.8713 |
| *75+* | 8 | 0.13 | 54 | 0.87 | 5 | 0.08 | 57 | 0.92 | 0.2568 | 0.592 | (0.176-1.988) | 0.3904 | 1.018 | (0.664-1.563) | 0.9661 |
| **Sex** |  |  |  |  |  |  |  |  |  |  |  |  |  |  |  |
| *Male* | 37 | 0.19 | 153 | 0.81 | 30 | 0.16 | 160 | 0.84 | 0.1936 | 0.775 | (0.454-1.324) | 0.3493 | 0.741 | (0.406-1.355) | 0.3292 |
| *Female* | 7 | 0.15 | 41 | 0.85 | 6 | 0.13 | 42 | 0.88 | 0.7055 | 0.837 | (0.248-2.823) | 0.7694 | 0.920 | (0.452-1.872) | 0.8144 |
| **Insurance type at index date2** |  |  |  |  |  |  |  |  |  |  |  |  |  |  |  |
| *Health insurance* | 37 | 0.18 | 170 | 0.82 | 35 | 0.17 | 172 | 0.83 | 0.7055 | 0.935 | (0.560-1.561) | 0.7961 | 0.922 | (0.532-1.599) | 0.7722 |
| *Medical aid* | 7 | 0.23 | 24 | 0.77 | 1 | 0.03 | 30 | 0.97 | 0.0339 | 0.114 | (0.012-1.130) | 0.0627 | 0.048 | (0.002-1.456) | 0.0792 |
| **Hospital type at index date2** |  |  |  |  |  |  |  |  |  |  |  |  |  |  |  |
| *Tertiary and general hospital* | 32 | 0.16 | 164 | 0.84 | 29 | 0.15 | 167 | 0.85 | 0.5775 | 0.890 | (0.513-1.545) | 0.6773 | 0.870 | (0.472-1.605) | 0.6547 |
| *Others* | 12 | 0.29 | 30 | 0.71 | 7 | 0.17 | 35 | 0.83 | 0.0588 | 0.500 | (0.167-1.498) | 0.2092 | 0.237 | (0.059-0.949) | 0.0424 |
| **History of COPD exacerbation** |  |  |  |  |  |  |  |  |  |  |  |  |  |  |  |
| *none* | 13 | 0.10 | 118 | 0.90 | 11 | 0.08 | 120 | 0.92 | 0.5637 | 0.832 | (0.354-1.953) | 0.6706 | 0.817 | (0.175-3.821) | 0.7955 |
| *1 moderate* | 24 | 0.28 | 61 | 0.72 | 17 | 0.20 | 68 | 0.80 | 0.0896 | 0.635 | (0.308-1.313) | 0.2174 | 0.719 | (0.380-1.362) | 0.3077 |
| *≥ 2 moderate OR ≥ 1 severe* | 7 | 0.32 | 15 | 0.68 | 8 | 0.36 | 14 | 0.64 | 0.7055 | 1.224 | (0.315-4.753) | 0.7592 | 1.362 | (0.316-5.874) | 0.6650 |
| **History of Pneumonia** |  |  |  |  |  |  |  |  |  |  |  |  |  |  |  |
| *No* | 31 | 0.18 | 144 | 0.82 | 23 | 0.13 | 152 | 0.87 | 0.0881 | 0.703 | (0.389-1.270) | 0.2409 | 0.657 | (0.318-1.359) | 0.2561 |
| *Yes* | 13 | 0.21 | 50 | 0.79 | 13 | 0.21 | 50 | 0.79 | 1.0000 | 1.000 | (0.412-2.428) | 1.0000 | 1.000 | (0.291-3.442) | 1.0000 |
| **History of Asthma** |  |  |  |  |  |  |  |  |  |  |  |  |  |  |  |
| *No* | 7 | 0.18 | 31 | 0.82 | 4 | 0.11 | 34 | 0.89 | 0.0833 | 0.521 | (0.131-2.080) | 0.3461 | 0.729 | (0.331-1.608) | 0.4236 |
| *Yes* | 37 | 0.19 | 163 | 0.82 | 32 | 0.16 | 168 | 0.84 | 0.3841 | 0.839 | (0.497-1.418) | 0.5103 | 0.811 | (0.439-1.497) | 0.5009 |
| **mCCI** |  |  |  |  |  |  |  |  |  |  |  |  |  |  |  |
| *0, 1* | 27 | 0.23 | 89 | 0.77 | 23 | 0.20 | 93 | 0.80 | 0.3458 | 0.815 | (0.431-1.541) | 0.5264 | 0.767 | (0.395-1.489) | 0.4300 |
| *2 +* | 17 | 0.14 | 105 | 0.86 | 13 | 0.11 | 109 | 0.89 | 0.3458 | 0.737 | (0.337-1.609) | 0.4401 | 0.767 | (0.387-1.522) | 0.4456 |
| **mCCI category** |  |  |  |  |  |  |  |  |  |  |  |  |  |  |  |
| *Congestive heart failure* |  |  |  |  |  |  |  |  |  |  |  |  |  |  |  |
| *No* | 35 | 0.17 | 168 | 0.83 | 32 | 0.16 | 171 | 0.84 | 0.5775 | 0.898 | (0.529-1.525) | 0.6896 | 0.879 | (0.494-1.563) | 0.6592 |
| *Yes* | 9 | 0.26 | 26 | 0.74 | 4 | 0.11 | 31 | 0.89 | 0.0588 | 0.373 | (0.096-1.445) | 0.1481 | NA | NA | NA |
| *Chronic pulmonary disease* |  |  |  |  |  |  |  |  |  |  |  |  |  |  |  |
| *No* | 2 | 0.18 | 9 | 0.82 | 1 | 0.09 | 10 | 0.91 | 0.3173 | 0.450 | (0.021-9.567) | 0.5734 | NA | NA | NA |
| *Yes* | 42 | 0.19 | 185 | 0.81 | 35 | 0.15 | 192 | 0.85 | 0.2367 | 0.803 | (0.489-1.318) | 0.3839 | 0.770 | (0.423-1.401) | 0.3906 |
| *Mild liver disease* |  |  |  |  |  |  |  |  |  |  |  |  |  |  |  |
| *No* | 33 | 0.20 | 128 | 0.80 | 24 | 0.15 | 137 | 0.85 | 0.0606 | 0.679 | (0.379-1.219) | 0.1936 | 0.618 | (0.319-1.195) | 0.1512 |
| *Yes* | 11 | 0.14 | 66 | 0.86 | 12 | 0.16 | 65 | 0.84 | 0.7815 | 1.108 | (0.447-2.744) | 0.8229 | 1.102 | (0.465-2.611) | 0.8234 |
| *Diabetes with chronic complications* |  |  |  |  |  |  |  |  |  |  |  |  |  |  |  |
| *No* | 42 | 0.22 | 153 | 0.78 | 33 | 0.17 | 162 | 0.83 | 0.1172 | 0.742 | (0.445-1.237) | 0.2511 | 0.701 | (0.399-1.232) | 0.2150 |
| *Yes* | 2 | 0.05 | 41 | 0.95 | 3 | 0.07 | 40 | 0.93 | 0.5637 | 1.538 | (0.226-10.471) | 0.6532 | 2.685 | (0.550-13.121) | 0.2158 |
| *Any malignancy, including lymphoma and leukemia* |  |  |  |  |  |  |  |  |  |  |  |  |  |  |  |
| *No* | 41 | 0.21 | 159 | 0.80 | 32 | 0.16 | 168 | 0.84 | 0.1172 | 0.739 | (0.441-1.236) | 0.2476 | 0.699 | (0.386-1.268) | 0.2375 |
| *Yes* | 3 | 0.08 | 35 | 0.92 | 4 | 0.11 | 34 | 0.89 | 0.5637 | 1.373 | (0.265-7.107) | 0.6986 | 1.671 | (0.809-3.451) | 0.1600 |
| **Index year** |  |  |  |  |  |  |  |  |  |  |  |  |  |  |  |
| *2017* | 26 | 0.19 | 108 | 0.81 | 16 | 0.12 | 118 | 0.88 | 0.0124 | 0.563 | (0.284-1.116) | 0.0993 | 0.499 | (0.237-1.051) | 0.0670 |
| *2018* | 18 | 0.17 | 86 | 0.83 | 20 | 0.19 | 84 | 0.81 | 0.6547 | 1.138 | (0.556-2.328) | 0.7218 | 1.183 | (0.556-2.519) | 0.6596 |

6.5.1.2 Severe exacerbation

| **SEVERE COPD EXACERBATION** |  | | | | | | | | | | | | | | |
| --- | --- | --- | --- | --- | --- | --- | --- | --- | --- | --- | --- | --- | --- | --- | --- |
|  | **Pre-BV** | | | | **Post-BV** | | | | **Mcnemar p-value** | **Crude OR** | | | **Adjusted OR** | | |
|  | **Event** | | **No Event** | | **Event** | | **No Event** | |  |  |  |  |  |  |  |
|  | n | % | n | % | n | % | n | % |  | OR | 95% CI | p-value | OR | 95% CI | p-value |
| **Overall** | 44 | 0.18 | 194 | 0.82 | 43 | 0.18 | 195 | 0.82 | 0.8964 | 0.972 | (0.609-1.553) | 0.9059 | 0.969 | (0.577-1.626) | 0.9039 |
| **COPD medication during pre and post-BV period** |  |  |  |  |  |  |  |  |  |  |  |  |  |  |  |
| *ICS/LABA* | 22 | 0.19 | 91 | 0.81 | 19 | 0.17 | 94 | 0.83 | 0.5637 | 0.836 | (0.420-1.665) | 0.6075 | 0.812 | (0.382-1.723) | 0.5838 |
| *LAMA* | 0 | 0.00 | 24 | 1.00 | 2 | 0.08 | 22 | 0.92 | NA | NA | NA | NA | 1.017 | (0.603-1.716) | 0.9730 |
| *Triple* | 16 | 0.24 | 51 | 0.76 | 15 | 0.22 | 52 | 0.78 | 0.8348 | 0.919 | (0.403-2.097) | 0.8395 | 0.904 | (0.324-2.520) | 0.8446 |
| *LABA/LAMA* | 6 | 0.18 | 28 | 0.82 | 7 | 0.21 | 27 | 0.79 | 0.7055 | 1.210 | (0.337-4.338) | 0.7634 | 1.260 | (0.399-3.975) | 0.6851 |
| **Age at index date 1** |  |  |  |  |  |  |  |  |  |  |  |  |  |  |  |
| *40 to < 65* | 13 | 0.19 | 54 | 0.81 | 9 | 0.13 | 58 | 0.87 | 0.3173 | 0.645 | (0.249-1.669) | 0.3602 | 0.642 | (0.235-1.751) | 0.3809 |
| *65 to < 75* | 14 | 0.13 | 95 | 0.87 | 20 | 0.18 | 89 | 0.82 | 0.2207 | 1.525 | (0.718-3.240) | 0.2695 | 1.246 | (0.711-2.184) | 0.4381 |
| *75+* | 17 | 0.27 | 45 | 0.73 | 14 | 0.23 | 48 | 0.77 | 0.4913 | 0.772 | (0.334-1.787) | 0.5399 | 0.688 | (0.254-1.866) | 0.4565 |
| **Sex** |  |  |  |  |  |  |  |  |  |  |  |  |  |  |  |
| *Male* | 32 | 0.17 | 158 | 0.83 | 36 | 0.19 | 154 | 0.81 | 0.5465 | 1.154 | (0.679-1.961) | 0.5942 | 1.177 | (0.670-2.068) | 0.5697 |
| *Female* | 12 | 0.25 | 36 | 0.75 | 7 | 0.15 | 41 | 0.85 | 0.1967 | 0.512 | (0.175-1.497) | 0.2157 | 0.411 | (0.091-1.845) | 0.2395 |
| **Insurance type at index date2** |  |  |  |  |  |  |  |  |  |  |  |  |  |  |  |
| *Health insurance* | 36 | 0.17 | 171 | 0.83 | 37 | 0.18 | 170 | 0.82 | 0.8886 | 1.034 | (0.621-1.721) | 0.8978 | 1.039 | (0.589-1.831) | 0.8947 |
| *Medical aid* | 8 | 0.26 | 23 | 0.74 | 6 | 0.19 | 25 | 0.81 | 0.4795 | 0.690 | (0.193-2.461) | 0.5557 | 0.773 | (0.283-2.111) | 0.6039 |
| **Hospital type at index date2** |  |  |  |  |  |  |  |  |  |  |  |  |  |  |  |
| *Tertiary and general hospital* | 40 | 0.20 | 156 | 0.80 | 38 | 0.19 | 158 | 0.81 | 0.7773 | 0.938 | (0.569-1.547) | 0.8010 | 0.929 | (0.535-1.613) | 0.7918 |
| *Others* | 4 | 0.10 | 38 | 0.90 | 5 | 0.12 | 37 | 0.88 | 0.7389 | 1.284 | (0.301-5.474) | 0.7297 | 1.358 | (0.310-5.941) | 0.6779 |
| **History of COPD exacerbation** |  |  |  |  |  |  |  |  |  |  |  |  |  |  |  |
| *none* | 24 | 0.18 | 107 | 0.82 | 14 | 0.11 | 117 | 0.89 | 0.0588 | 0.533 | (0.260-1.095) | 0.0861 | 0.480 | (0.231-0.997) | 0.0491 |
| *1 moderate* | 12 | 0.14 | 73 | 0.86 | 20 | 0.24 | 65 | 0.76 | 0.0881 | 1.872 | (0.836-4.192) | 0.1258 | 2.125 | (0.848-5.329) | 0.1066 |
| *≥ 2 moderate OR ≥ 1 severe* | 8 | 0.36 | 14 | 0.64 | 9 | 0.41 | 13 | 0.59 | 0.7389 | 1.212 | (0.324-4.534) | 0.7654 | 1.199 | (0.254-5.661) | 0.8099 |
| **History of Pneumonia** |  |  |  |  |  |  |  |  |  |  |  |  |  |  |  |
| *No* | 22 | 0.13 | 153 | 0.87 | 25 | 0.14 | 150 | 0.86 | 0.6015 | 1.159 | (0.622-2.158) | 0.6399 | 1.174 | (0.617-2.236) | 0.6226 |
| *Yes* | 22 | 0.35 | 41 | 0.65 | 18 | 0.29 | 45 | 0.71 | 0.4328 | 0.745 | (0.344-1.617) | 0.4510 | 0.691 | (0.285-1.675) | 0.4073 |
| **History of Asthma** |  |  |  |  |  |  |  |  |  |  |  |  |  |  |  |
| *No* | 2 | 0.05 | 36 | 0.95 | 3 | 0.08 | 35 | 0.92 | 0.6547 | 1.543 | (0.222-10.702) | 0.6527 | 1.305 | (0.589-2.891) | 0.5019 |
| *Yes* | 42 | 0.21 | 158 | 0.79 | 40 | 0.20 | 160 | 0.80 | 0.7855 | 0.940 | (0.576-1.535) | 0.8051 | 0.933 | (0.540-1.614) | 0.8043 |
| **mCCI** |  |  |  |  |  |  |  |  |  |  |  |  |  |  |  |
| *0, 1* | 16 | 0.14 | 100 | 0.86 | 22 | 0.19 | 94 | 0.81 | 0.2568 | 1.463 | (0.717-2.985) | 0.2932 | 1.553 | (0.705-3.421) | 0.2716 |
| *2 +* | 28 | 0.23 | 94 | 0.77 | 21 | 0.17 | 101 | 0.83 | 0.2087 | 0.698 | (0.368-1.325) | 0.2688 | 0.655 | (0.316-1.357) | 0.2522 |
| **mCCI category** |  |  |  |  |  |  |  |  |  |  |  |  |  |  |  |
| *Congestive heart failure* |  |  |  |  |  |  |  |  |  |  |  |  |  |  |  |
| *No* | 34 | 0.17 | 169 | 0.83 | 35 | 0.17 | 168 | 0.83 | 0.8864 | 1.036 | (0.614-1.746) | 0.8953 | 1.041 | (0.577-1.878) | 0.8934 |
| *Yes* | 10 | 0.29 | 25 | 0.71 | 8 | 0.23 | 27 | 0.77 | 0.5271 | 0.741 | (0.239-2.300) | 0.5939 | 0.632 | (0.152-2.633) | 0.5180 |
| *Chronic pulmonary disease* |  |  |  |  |  |  |  |  |  |  |  |  |  |  |  |
| *No* | 1 | 0.09 | 10 | 0.91 | 1 | 0.09 | 10 | 0.91 | 1.0000 | 1.000 | (0.031-32.014) | 1.0000 | 1.000 | (0.030-33.514) | 1.0000 |
| *Yes* | 43 | 0.19 | 184 | 0.81 | 42 | 0.19 | 185 | 0.81 | 0.8946 | 0.971 | (0.604-1.562) | 0.9046 | 0.968 | (0.567-1.652) | 0.9039 |
| *Mild liver disease* |  |  |  |  |  |  |  |  |  |  |  |  |  |  |  |
| *No* | 27 | 0.17 | 134 | 0.83 | 29 | 0.18 | 132 | 0.82 | 0.7518 | 1.090 | (0.609-1.953) | 0.7698 | 1.103 | (0.579-2.102) | 0.7645 |
| *Yes* | 17 | 0.22 | 60 | 0.78 | 14 | 0.18 | 63 | 0.82 | 0.4913 | 0.784 | (0.349-1.761) | 0.5515 | NA | NA | NA |
| *Diabetes with chronic complications* |  |  |  |  |  |  |  |  |  |  |  |  |  |  |  |
| *No* | 37 | 0.19 | 158 | 0.81 | 39 | 0.20 | 156 | 0.80 | 0.7855 | 1.068 | (0.644-1.770) | 0.7990 | 1.077 | (0.613-1.893) | 0.7945 |
| *Yes* | 7 | 0.16 | 36 | 0.84 | 4 | 0.09 | 39 | 0.91 | 0.1797 | 0.527 | (0.135-2.064) | 0.3494 | 1.024 | (0.389-2.697) | 0.9801 |
| *Any malignancy, including lymphoma and leukemia* |  |  |  |  |  |  |  |  |  |  |  |  |  |  |  |
| *No* | 35 | 0.18 | 165 | 0.83 | 37 | 0.19 | 163 | 0.82 | 0.7728 | 1.070 | (0.640-1.791) | 0.7954 | 1.083 | (0.588-1.995) | 0.7969 |
| *Yes* | 9 | 0.24 | 29 | 0.76 | 6 | 0.16 | 32 | 0.84 | 0.3657 | 0.604 | (0.181-2.013) | 0.4017 | 0.553 | (0.112-2.736) | 0.4573 |
| **Index year** |  |  |  |  |  |  |  |  |  |  |  |  |  |  |  |
| *2017* | 18 | 0.13 | 116 | 0.87 | 25 | 0.19 | 109 | 0.81 | 0.2230 | 1.478 | (0.758-2.884) | 0.2496 | 1.553 | (0.734-3.283) | 0.2472 |
| *2018* | 26 | 0.25 | 78 | 0.75 | 18 | 0.17 | 86 | 0.83 | 0.1167 | 0.628 | (0.316-1.247) | 0.1814 | 0.735 | (0.412-1.311) | 0.2939 |

6.5.1.3 Moderate-to-severe exacerbation

| **MODERATE TO SEVERE COPD EXACERBATION** |  | | | | | | | | | | | | | | | | |
| --- | --- | --- | --- | --- | --- | --- | --- | --- | --- | --- | --- | --- | --- | --- | --- | --- | --- |
|  | **Pre-BV** | | | | **Post-BV** | | | | **Mcnemar p-value** | **Crude OR** | | | | **Adjusted OR** | | | |
|  | **Event** | | **No Event** | | **Event** | | **No Event** | |  |  |  |  |  |  |  |  |  |
|  | n | % | n | % | n | % | n | % |  | OR | 95% CI | p-value | OR | | 95% CI | p-value |  |
| **Overall** | 81 | 0.34 | 157 | 0.66 | 69 | 0.29 | 169 | 0.71 | 0.1336 | 0.791 | (0.536-1.169) | 0.2390 | 0.761 | | (0.491-1.179) | 0.2204 |  |
| **COPD medication during pre and post-BV period** |  |  |  |  |  |  |  |  |  |  |  |  |  | |  |  |  |
| *ICS/LABA* | 32 | 0.28 | 81 | 0.72 | 32 | 0.28 | 81 | 0.72 | 1.0000 | 1.000 | (0.556-1.800) | 1.0000 | 1.000 | | (0.516-1.938) | 1.0000 |  |
| *LAMA* | 1 | 0.04 | 23 | 0.96 | 3 | 0.13 | 21 | 0.88 | 0.1573 | 3.286 | (0.264-40.920) | 0.3393 | 1.392 | | (0.514-3.768) | 0.4993 |  |
| *Triple* | 36 | 0.54 | 31 | 0.46 | 24 | 0.36 | 43 | 0.64 | 0.0186 | 0.481 | (0.236-0.979) | 0.0436 | 0.406 | | (0.181-0.909) | 0.0289 |  |
| *LABA/LAMA* | 12 | 0.35 | 22 | 0.65 | 10 | 0.29 | 24 | 0.71 | 0.4142 | 0.764 | (0.261-2.236) | 0.6133 | 0.790 | | (0.299-2.089) | 0.6257 |  |
| **Age at index date 1** |  |  |  |  |  |  |  |  |  |  |  |  |  | |  |  |  |
| *40 to < 65* | 25 | 0.37 | 42 | 0.63 | 17 | 0.25 | 50 | 0.75 | 0.0736 | 0.571 | (0.267-1.221) | 0.1458 | 0.569 | | (0.260-1.243) | 0.1545 |  |
| *65 to < 75* | 32 | 0.29 | 77 | 0.71 | 34 | 0.31 | 75 | 0.69 | 0.6831 | 1.091 | (0.606-1.963) | 0.7698 | 1.108 | | (0.561-2.191) | 0.7652 |  |
| *75+* | 24 | 0.39 | 38 | 0.61 | 18 | 0.29 | 44 | 0.71 | 0.1797 | 0.648 | (0.300-1.400) | 0.2644 | 0.554 | | (0.211-1.460) | 0.2277 |  |
| **Sex** |  |  |  |  |  |  |  |  |  |  |  |  |  | |  |  |  |
| *Male* | 65 | 0.34 | 125 | 0.66 | 58 | 0.31 | 132 | 0.69 | 0.3072 | 0.845 | (0.547-1.304) | 0.4451 | 0.819 | | (0.504-1.333) | 0.4198 |  |
| *Female* | 16 | 0.33 | 32 | 0.67 | 11 | 0.23 | 37 | 0.77 | 0.2253 | 0.595 | (0.233-1.515) | 0.2692 | 0.456 | | (0.139-1.499) | 0.1909 |  |
| **Insurance type at index date2** |  |  |  |  |  |  |  |  |  |  |  |  |  | |  |  |  |
| *Health insurance* | 68 | 0.33 | 139 | 0.67 | 62 | 0.30 | 145 | 0.70 | 0.4142 | 0.874 | (0.575-1.329) | 0.5270 | 0.853 | | (0.534-1.362) | 0.5027 |  |
| *Medical aid* | 13 | 0.42 | 18 | 0.58 | 7 | 0.23 | 24 | 0.77 | 0.0578 | 0.404 | (0.125-1.300) | 0.1236 | NA | | NA | NA |  |
| **Hospital type at index date2** |  |  |  |  |  |  |  |  |  |  |  |  |  | |  |  |  |
| *Tertiary and general hospital* | 66 | 0.34 | 130 | 0.66 | 57 | 0.29 | 139 | 0.71 | 0.2164 | 0.808 | (0.525-1.243) | 0.3300 | 0.780 | | (0.481-1.266) | 0.3131 |  |
| *Others* | 15 | 0.36 | 27 | 0.64 | 12 | 0.29 | 30 | 0.71 | 0.3657 | 0.720 | (0.276-1.880) | 0.4932 | 0.640 | | (0.207-1.977) | 0.4293 |  |
| **History of COPD exacerbation** |  |  |  |  |  |  |  |  |  |  |  |  |  | |  |  |  |
| *none* | 35 | 0.27 | 96 | 0.73 | 23 | 0.18 | 108 | 0.82 | 0.0285 | 0.584 | (0.320-1.066) | 0.0793 | 0.538 | | (0.275-1.053) | 0.0702 |  |
| *1 moderate* | 34 | 0.40 | 51 | 0.60 | 33 | 0.39 | 52 | 0.61 | 0.8415 | 0.952 | (0.508-1.784) | 0.8764 | 0.942 | | (0.451-1.964) | 0.8709 |  |
| *≥ 2 moderate OR ≥ 1 severe* | 12 | 0.55 | 10 | 0.45 | 13 | 0.59 | 9 | 0.41 | 0.7389 | 1.204 | (0.329-4.404) | 0.7692 | 1.198 | | (0.248-5.785) | 0.8140 |  |
| **History of Pneumonia** |  |  |  |  |  |  |  |  |  |  |  |  |  | |  |  |  |
| *No* | 52 | 0.30 | 123 | 0.70 | 43 | 0.25 | 132 | 0.75 | 0.1495 | 0.771 | (0.478-1.242) | 0.2828 | 0.740 | | (0.436-1.255) | 0.2620 |  |
| *Yes* | 29 | 0.46 | 34 | 0.54 | 26 | 0.41 | 37 | 0.59 | 0.5485 | 0.824 | (0.399-1.701) | 0.5950 | 0.777 | | (0.318-1.902) | 0.5754 |  |
| **History of Asthma** |  |  |  |  |  |  |  |  |  |  |  |  |  | |  |  |  |
| *No* | 8 | 0.21 | 30 | 0.79 | 7 | 0.18 | 31 | 0.82 | 0.7055 | 0.847 | (0.259-2.772) | 0.7778 | 0.767 | | (0.256-2.298) | 0.6269 |  |
| *Yes* | 73 | 0.37 | 127 | 0.64 | 62 | 0.31 | 138 | 0.69 | 0.1451 | 0.782 | (0.514-1.189) | 0.2477 | 0.752 | | (0.468-1.207) | 0.2358 |  |
| **mCCI** |  |  |  |  |  |  |  |  |  |  |  |  |  | |  |  |  |
| *0, 1* | 38 | 0.33 | 78 | 0.67 | 40 | 0.34 | 76 | 0.66 | 0.7237 | 1.080 | (0.621-1.878) | 0.7825 | 1.101 | | (0.593-2.047) | 0.7583 |  |
| *2 +* | 43 | 0.35 | 79 | 0.65 | 29 | 0.24 | 93 | 0.76 | 0.0133 | 0.573 | (0.325-1.009) | 0.0538 | 0.507 | | (0.259-0.989) | 0.0463 |  |
| **mCCI category** |  |  |  |  |  |  |  |  |  |  |  |  |  | |  |  |  |
| *Congestive heart failure* |  |  |  |  |  |  |  |  |  |  |  |  |  | |  |  |  |
| *No* | 62 | 0.31 | 141 | 0.69 | 58 | 0.29 | 145 | 0.71 | 0.5791 | 0.910 | (0.592-1.399) | 0.6648 | 0.894 | | (0.540-1.479) | 0.6599 |  |
| *Yes* | 19 | 0.54 | 16 | 0.46 | 11 | 0.31 | 24 | 0.69 | 0.0209 | 0.386 | (0.138-1.077) | 0.0680 | 0.301 | | (0.079-1.142) | 0.0760 |  |
| *Chronic pulmonary disease* |  |  |  |  |  |  |  |  |  |  |  |  |  | |  |  |  |
| *No* | 3 | 0.27 | 8 | 0.73 | 2 | 0.18 | 9 | 0.82 | 0.5637 | 0.593 | (0.053-6.642) | 0.6399 | 0.449 | | (0.024-8.349) | 0.4894 |  |
| *Yes* | 78 | 0.34 | 149 | 0.66 | 67 | 0.30 | 160 | 0.70 | 0.1590 | 0.800 | (0.537-1.191) | 0.2708 | 0.769 | | (0.491-1.204) | 0.2499 |  |
| *Mild liver disease* |  |  |  |  |  |  |  |  |  |  |  |  |  | |  |  |  |
| *No* | 54 | 0.34 | 107 | 0.66 | 48 | 0.30 | 113 | 0.70 | 0.3763 | 0.842 | (0.523-1.354) | 0.4749 | 0.817 | | (0.472-1.416) | 0.4696 |  |
| *Yes* | 27 | 0.35 | 50 | 0.65 | 21 | 0.27 | 56 | 0.73 | 0.1573 | 0.694 | (0.344-1.401) | 0.3040 | 0.625 | | (0.282-1.386) | 0.2437 |  |
| *Diabetes with chronic complications* |  |  |  |  |  |  |  |  |  |  |  |  |  | |  |  |  |
| *No* | 72 | 0.37 | 123 | 0.63 | 62 | 0.32 | 133 | 0.68 | 0.1814 | 0.796 | (0.522-1.215) | 0.2892 | 0.763 | | (0.473-1.233) | 0.2681 |  |
| *Yes* | 9 | 0.21 | 34 | 0.79 | 7 | 0.16 | 36 | 0.84 | 0.4795 | 0.735 | (0.235-2.295) | 0.5876 | NA | | NA | NA |  |
| *Any malignancy, including lymphoma and leukemia* |  |  |  |  |  |  |  |  |  |  |  |  |  | |  |  |  |
| *No* | 70 | 0.35 | 130 | 0.65 | 60 | 0.30 | 140 | 0.70 | 0.1736 | 0.796 | (0.521-1.215) | 0.2886 | 0.759 | | (0.471-1.221) | 0.2536 |  |
| *Yes* | 11 | 0.29 | 27 | 0.71 | 9 | 0.24 | 29 | 0.76 | 0.5271 | 0.762 | (0.260-2.230) | 0.6107 | 0.696 | | (0.165-2.930) | 0.6128 |  |
| **Index year** |  |  |  |  |  |  |  |  |  |  |  |  |  | |  |  |  |
| *2017* | 41 | 0.31 | 93 | 0.69 | 37 | 0.28 | 97 | 0.72 | 0.5050 | 0.865 | (0.507-1.477) | 0.5931 | 0.839 | | (0.439-1.606) | 0.5947 |  |
| *2018* | 40 | 0.38 | 64 | 0.62 | 32 | 0.31 | 72 | 0.69 | 0.1306 | 0.711 | (0.397-1.275) | 0.2493 | 0.664 | | (0.347-1.272) | 0.2146 |  |

6.5.2. Frequency of antibiotics & OCS-used COPD AE

6.5.2.1. Moderate exacerbation

| **MODERATE COPD EXACERBATION** | **All** | **Pre-BV** | **Post-BV** | **Relative frequency** | **p-value** |
| --- | --- | --- | --- | --- | --- |
|  |  |  |  |  |  |
| ***Number of event per person per year*** | 0.38 | 0.39 | 0.36 | 0.90 | 0.7332 |
| *Total event* | 179 | 94 | 85 |  |  |
| *Patients with event* | 80 | 44 | 36 |  |  |
| *observational period (year)* |  |  |  |  |  |
| *Mean* | 365.04 | 365.08 | 365.00 |  |  |
| *SD* | 0.20 | 0.28 | 0.00 |  |  |
| *Median* | 365 | 365 | 365 |  |  |
| *Min* | 365 | 365 | 365 |  |  |
| *Max* | 366 | 366 | 365 |  |  |
| *P25* | 365 | 365 | 365 |  |  |
| *P75* | 365 | 365 | 365 |  |  |
| **COPD medication during pre and post-BV period** |  |  |  |  |  |
| *ICS/LABA* | 0.22 | 0.17 | 0.27 | 1.63 | 0.2942 |
| *LAMA* | 0.06 | 0.08 | 0.04 | 0.50 | 0.6484 |
| *Triple* | 0.70 | 0.87 | 0.54 | 0.62 | 0.2768 |
| *LABA/LAMA* | 0.47 | 0.44 | 0.50 | 1.13 | 0.8616 |
| **Age at index date 1** |  |  |  |  |  |
| *40 to < 65* | 0.54 | 0.60 | 0.48 | 0.80 | 0.6623 |
| *65 to < 75* | 0.33 | 0.31 | 0.35 | 1.12 | 0.7594 |
| *75+* | 0.28 | 0.32 | 0.24 | 0.75 | 0.7190 |
| **Sex** |  |  |  |  |  |
| *Male* | 0.35 | 0.37 | 0.33 | 0.89 | 0.7083 |
| *Female* | 0.49 | 0.50 | 0.48 | 0.96 | 0.9490 |
| **Insurance type at index date2** |  |  |  |  |  |
| *Health insurance* | 0.37 | 0.36 | 0.39 | 1.07 | 0.8332 |
| *Medical aid* | 0.39 | 0.61 | 0.16 | 0.26 | 0.2280 |
| **Hospital type at index date2** |  |  |  |  |  |
| *Tertiary and general hospital* | 0.30 | 0.27 | 0.34 | 1.29 | 0.4192 |
| *Others* | 0.71 | 1.00 | 0.43 | 0.43 | 0.1962 |
| **History of COPD exacerbation** |  |  |  |  |  |
| *none* | 0.12 | 0.12 | 0.12 | 1.00 | 1.0000 |
| *1 moderate* | 0.63 | 0.74 | 0.52 | 0.70 | 0.3727 |
| *≥ 2 moderate OR ≥ 1 severe* | 0.91 | 0.68 | 1.14 | 1.67 | 0.4034 |
| **History of Pneumonia** |  |  |  |  |  |
| *No* | 0.30 | 0.32 | 0.28 | 0.88 | 0.7126 |
| *Yes* | 0.59 | 0.60 | 0.57 | 0.95 | 0.9137 |
| **History of Asthma** |  |  |  |  |  |
| *No* | 0.22 | 0.21 | 0.24 | 1.12 | 0.8705 |
| *Yes* | 0.41 | 0.43 | 0.38 | 0.88 | 0.6964 |
| **mCCI** |  |  |  |  |  |
| *0, 1* | 0.53 | 0.61 | 0.46 | 0.75 | 0.4336 |
| *2 +* | 0.23 | 0.19 | 0.26 | 1.39 | 0.4626 |
| **mCCI category** |  |  |  |  |  |
| *Congestive heart failure* |  |  |  |  |  |
| *No* | 0.40 | 0.41 | 0.39 | 0.96 | 0.9092 |
| *Yes* | 0.23 | 0.31 | 0.14 | 0.45 | 0.1886 |
| *Dementia* |  |  |  |  |  |
| *No* | 0.38 | 0.40 | 0.36 | 0.89 | 0.7039 |
| *Yes* | 0.25 | 0.17 | 0.33 | 2.00 | 0.6634 |
| *Chronic pulmonary disease* |  |  |  |  |  |
| *No* | 0.18 | 0.18 | 0.18 | 1.00 | 1.0000 |
| *Yes* | 0.39 | 0.41 | 0.37 | 0.90 | 0.7320 |
| *Rheumatologic disease* |  |  |  |  |  |
| *No* | 0.38 | 0.41 | 0.35 | 0.86 | 0.6155 |
| *Yes* | 0.33 | 0.17 | 0.50 | 3.00 | 0.4170 |
| *Mild liver disease* |  |  |  |  |  |
| *No* | 0.41 | 0.48 | 0.34 | 0.71 | 0.3491 |
| *Yes* | 0.31 | 0.22 | 0.39 | 1.76 | 0.2694 |
| *Diabetes with chronic complications* |  |  |  |  |  |
| *No* | 0.43 | 0.47 | 0.40 | 0.84 | 0.5590 |
| *Yes* | 0.12 | 0.05 | 0.19 | 4.00 | 0.2554 |
| *Hemiplegia or paraplegia* |  |  |  |  |  |
| *No* | 0.38 | 0.40 | 0.36 | 0.89 | 0.7046 |
| *Yes* | 0.17 | NA | 0.33 | NA | NA |
| *Renal disease* |  |  |  |  |  |
| *No* | 0.37 | 0.40 | 0.35 | 0.87 | 0.6397 |
| *Yes* | 0.41 | 0.27 | 0.55 | 2.00 | 0.6244 |
| *Any malignancy, including lymphoma and leukemia* |  |  |  |  |  |
| *No* | 0.42 | 0.45 | 0.38 | 0.84 | 0.5826 |
| *Yes* | 0.17 | 0.11 | 0.24 | 2.25 | 0.4267 |
| *Moderate or severe liver disease* |  |  |  |  |  |
| *No* | 0.38 | 0.40 | 0.36 | 0.90 | 0.7331 |
| *Yes* | NA | NA | NA | NA | NA |
| *Metastatic solid tumor* |  |  |  |  |  |
| *No* | 0.38 | 0.40 | 0.35 | 0.89 | 0.7047 |
| *Yes* | 0.50 | NA | 1.00 | NA | NA |
| *HIV* |  |  |  |  |  |
| *No* | 0.38 | 0.39 | 0.36 | 0.90 | 0.7332 |
| *Yes* | NA | NA | NA | NA | NA |
| **Index year** |  |  |  |  |  |
| *2017* | 0.39 | 0.44 | 0.34 | 0.78 | 0.5395 |
| *2018* | 0.36 | 0.34 | 0.38 | 1.11 | 0.8005 |

6.5.2.2. Severe exacerbation

| **SEVERE COPD EXACERBATION** | **All** | **Pre-BV** | **Post-BV** | **Relative frequency** | **p-value** |
| --- | --- | --- | --- | --- | --- |
|  |  |  |  |  |  |
| ***Number of event per person per year*** | 0.28 | 0.29 | 0.28 | 0.99 | 0.9543 |
| *Total event* | 135 | 68 | 67 |  |  |
| *Patients with event* | 87 | 44 | 43 |  |  |
| *observational period (year)* |  |  |  |  |  |
| *Mean* | 365.04 | 365.08 | 365.00 |  |  |
| *SD* | 0.20 | 0.28 | 0.00 |  |  |
| *Median* | 365 | 365 | 365 |  |  |
| *Min* | 365 | 365 | 365 |  |  |
| *Max* | 366 | 366 | 365 |  |  |
| *P25* | 365 | 365 | 365 |  |  |
| *P75* | 365 | 365 | 365 |  |  |
| **COPD medication during pre and post-BV period** |  |  |  |  |  |
| *ICS/LABA* | 0.25 | 0.27 | 0.23 | 0.84 | 0.6003 |
| *LAMA* | 0.04 | NA | 0.08 | NA | NA |
| *Triple* | 0.38 | 0.37 | 0.39 | 1.04 | 0.9311 |
| *LABA/LAMA* | 0.37 | 0.35 | 0.38 | 1.08 | 0.9096 |
| **Age at index date 1** |  |  |  |  |  |
| *40 to < 65* | 0.30 | 0.31 | 0.28 | 0.90 | 0.8606 |
| *65 to < 75* | 0.18 | 0.17 | 0.20 | 1.22 | 0.5649 |
| *75+* | 0.44 | 0.47 | 0.42 | 0.90 | 0.7897 |
| **Sex** |  |  |  |  |  |
| *Male* | 0.25 | 0.23 | 0.27 | 1.18 | 0.5480 |
| *Female* | 0.41 | 0.50 | 0.31 | 0.62 | 0.4372 |
| **Insurance type at index date2** |  |  |  |  |  |
| *Health insurance* | 0.25 | 0.26 | 0.25 | 0.98 | 0.9426 |
| *Medical aid* | 0.48 | 0.48 | 0.48 | 1.00 | 1.0000 |
| **Hospital type at index date2** |  |  |  |  |  |
| *Tertiary and general hospital* | 0.32 | 0.32 | 0.32 | 0.98 | 0.9533 |
| *Others* | 0.12 | 0.12 | 0.12 | 1.00 | 1.0000 |
| **History of COPD exacerbation** |  |  |  |  |  |
| *none* | 0.18 | 0.23 | 0.12 | 0.53 | 0.0651 |
| *1 moderate* | 0.23 | 0.19 | 0.27 | 1.44 | 0.3044 |
| *≥ 2 moderate OR ≥ 1 severe* | 1.14 | 1.00 | 1.27 | 1.27 | 0.6543 |
| **History of Pneumonia** |  |  |  |  |  |
| *No* | 0.16 | 0.16 | 0.16 | 1.00 | 0.9997 |
| *Yes* | 0.63 | 0.64 | 0.62 | 0.97 | 0.9461 |
| **History of Asthma** |  |  |  |  |  |
| *No* | 0.08 | 0.08 | 0.08 | 1.00 | 1.0000 |
| *Yes* | 0.32 | 0.33 | 0.32 | 0.98 | 0.9534 |
| **mCCI** |  |  |  |  |  |
| *0, 1* | 0.27 | 0.22 | 0.32 | 1.42 | 0.4020 |
| *2 +* | 0.30 | 0.34 | 0.25 | 0.71 | 0.3027 |
| **mCCI category** |  |  |  |  |  |
| *Congestive heart failure* |  |  |  |  |  |
| *No* | 0.25 | 0.24 | 0.27 | 1.10 | 0.7360 |
| *Yes* | 0.46 | 0.54 | 0.37 | 0.68 | 0.5107 |
| *Dementia* |  |  |  |  |  |
| *No* | 0.29 | 0.29 | 0.28 | 0.99 | 0.9542 |
| *Yes* | 0.17 | 0.17 | 0.17 | 1.00 | 1.0000 |
| *Chronic pulmonary disease* |  |  |  |  |  |
| *No* | 0.09 | 0.09 | 0.09 | 1.00 | 1.0000 |
| *Yes* | 0.29 | 0.30 | 0.29 | 0.99 | 0.9541 |
| *Rheumatologic disease* |  |  |  |  |  |
| *No* | 0.29 | 0.30 | 0.29 | 0.97 | 0.9081 |
| *Yes* | 0.13 | 0.08 | 0.17 | 2.00 | 0.5741 |
| *Mild liver disease* |  |  |  |  |  |
| *No* | 0.26 | 0.24 | 0.29 | 1.18 | 0.6151 |
| *Yes* | 0.32 | 0.38 | 0.27 | 0.72 | 0.4461 |
| *Diabetes with chronic complications* |  |  |  |  |  |
| *No* | 0.31 | 0.30 | 0.32 | 1.07 | 0.8107 |
| *Yes* | 0.17 | 0.23 | 0.12 | 0.50 | 0.3009 |
| *Hemiplegia or paraplegia* |  |  |  |  |  |
| *No* | 0.28 | 0.28 | 0.28 | 1.00 | 0.9999 |
| *Yes* | 0.50 | 0.67 | 0.33 | 0.50 | 0.5805 |
| *Renal disease* |  |  |  |  |  |
| *No* | 0.28 | 0.28 | 0.29 | 1.02 | 0.9535 |
| *Yes* | 0.27 | 0.36 | 0.18 | 0.50 | 0.4580 |
| *Any malignancy, including lymphoma and leukemia* |  |  |  |  |  |
| *No* | 0.29 | 0.28 | 0.30 | 1.09 | 0.7635 |
| *Yes* | 0.26 | 0.34 | 0.18 | 0.54 | 0.2541 |
| *Moderate or severe liver disease* |  |  |  |  |  |
| *No* | 0.29 | 0.29 | 0.28 | 0.99 | 0.9543 |
| *Yes* | NA | NA | NA | NA | NA |
| *Metastatic solid tumor* |  |  |  |  |  |
| *No* | 0.29 | 0.29 | 0.28 | 0.99 | 0.9543 |
| *Yes* | NA | NA | NA | NA | NA |
| *HIV* |  |  |  |  |  |
| *No* | 0.28 | 0.29 | 0.28 | 0.99 | 0.9543 |
| *Yes* | NA | NA | NA | NA | NA |
| **Index year** |  |  |  |  |  |
| *2017* | 0.23 | 0.20 | 0.26 | 1.30 | 0.5132 |
| *2018* | 0.35 | 0.39 | 0.31 | 0.78 | 0.4750 |

6.5.2.3. Moderate-to-severe exacerbation

| **MODERATE TO SEVERE COPD EXACERBATION** | **All** | **Pre-BV** | **Post-BV** | **Relative frequency** | **p-value** |
| --- | --- | --- | --- | --- | --- |
|  |  |  |  |  |  |
| ***Number of event per person per year*** | 0.66 | 0.68 | 0.64 | 0.94 | 0.7592 |
| *Total event* | 314 | 162 | 152 |  |  |
| *Patients with event* | 150 | 81 | 69 |  |  |
| *observational period (year)* |  |  |  |  |  |
| *Mean* | 365.04 | 365.08 | 365.00 |  |  |
| *SD* | 0.20 | 0.28 | 0.00 |  |  |
| *Median* | 365 | 365 | 365 |  |  |
| *Min* | 365 | 365 | 365 |  |  |
| *Max* | 366 | 366 | 365 |  |  |
| *P25* | 365 | 365 | 365 |  |  |
| *P75* | 365 | 365 | 365 |  |  |
| **COPD medication during pre and post-BV period** |  |  |  |  |  |
| *ICS/LABA* | 0.47 | 0.44 | 0.50 | 1.14 | 0.6403 |
| *LAMA* | 0.10 | 0.08 | 0.13 | 1.50 | 0.7162 |
| *Triple* | 1.08 | 1.24 | 0.93 | 0.75 | 0.3674 |
| *LABA/LAMA* | 0.84 | 0.79 | 0.88 | 1.11 | 0.8485 |
| **Age at index date 1** |  |  |  |  |  |
| *40 to < 65* | 0.84 | 0.91 | 0.76 | 0.84 | 0.6615 |
| *65 to < 75* | 0.51 | 0.48 | 0.55 | 1.15 | 0.6007 |
| *75+* | 0.73 | 0.79 | 0.66 | 0.84 | 0.6510 |
| **Sex** |  |  |  |  |  |
| *Male* | 0.60 | 0.60 | 0.60 | 1.00 | 0.9984 |
| *Female* | 0.90 | 1.00 | 0.79 | 0.79 | 0.6243 |
| **Insurance type at index date2** |  |  |  |  |  |
| *Health insurance* | 0.63 | 0.62 | 0.64 | 1.03 | 0.8873 |
| *Medical aid* | 0.87 | 1.10 | 0.65 | 0.59 | 0.3621 |
| **Hospital type at index date2** |  |  |  |  |  |
| *Tertiary and general hospital* | 0.62 | 0.59 | 0.66 | 1.12 | 0.6012 |
| *Others* | 0.83 | 1.12 | 0.55 | 0.49 | 0.1904 |
| **History of COPD exacerbation** |  |  |  |  |  |
| *none* | 0.30 | 0.35 | 0.24 | 0.70 | 0.1950 |
| *1 moderate* | 0.86 | 0.93 | 0.79 | 0.85 | 0.5850 |
| *≥ 2 moderate OR ≥ 1 severe* | 2.05 | 1.68 | 2.41 | 1.43 | 0.3972 |
| **History of Pneumonia** |  |  |  |  |  |
| *No* | 0.46 | 0.48 | 0.44 | 0.92 | 0.7322 |
| *Yes* | 1.21 | 1.24 | 1.19 | 0.96 | 0.9032 |
| **History of Asthma** |  |  |  |  |  |
| *No* | 0.30 | 0.29 | 0.32 | 1.09 | 0.8845 |
| *Yes* | 0.73 | 0.76 | 0.70 | 0.93 | 0.7288 |
| **mCCI** |  |  |  |  |  |
| *0, 1* | 0.81 | 0.84 | 0.78 | 0.93 | 0.7922 |
| *2 +* | 0.52 | 0.53 | 0.51 | 0.95 | 0.8732 |
| **mCCI category** |  |  |  |  |  |
| *Congestive heart failure* |  |  |  |  |  |
| *No* | 0.66 | 0.65 | 0.66 | 1.02 | 0.9467 |
| *Yes* | 0.69 | 0.86 | 0.51 | 0.60 | 0.2703 |
| *Dementia* |  |  |  |  |  |
| *No* | 0.67 | 0.69 | 0.64 | 0.93 | 0.7352 |
| *Yes* | 0.42 | 0.33 | 0.50 | 1.50 | 0.6895 |
| *Chronic pulmonary disease* |  |  |  |  |  |
| *No* | 0.27 | 0.27 | 0.27 | 1.00 | 1.0000 |
| *Yes* | 0.68 | 0.70 | 0.66 | 0.94 | 0.7582 |
| *Rheumatologic disease* |  |  |  |  |  |
| *No* | 0.67 | 0.70 | 0.64 | 0.91 | 0.6411 |
| *Yes* | 0.46 | 0.25 | 0.67 | 2.67 | 0.3223 |
| *Mild liver disease* |  |  |  |  |  |
| *No* | 0.67 | 0.72 | 0.63 | 0.87 | 0.5833 |
| *Yes* | 0.63 | 0.60 | 0.66 | 1.11 | 0.7750 |
| *Diabetes with chronic complications* |  |  |  |  |  |
| *No* | 0.74 | 0.77 | 0.71 | 0.93 | 0.7279 |
| *Yes* | 0.29 | 0.28 | 0.30 | 1.08 | 0.8886 |
| *Hemiplegia or paraplegia* |  |  |  |  |  |
| *No* | 0.66 | 0.68 | 0.64 | 0.94 | 0.7591 |
| *Yes* | 0.67 | 0.67 | 0.67 | 1.00 | 1.0000 |
| *Renal disease* |  |  |  |  |  |
| *No* | 0.66 | 0.68 | 0.63 | 0.93 | 0.7294 |
| *Yes* | 0.68 | 0.64 | 0.73 | 1.14 | 0.8938 |
| *Any malignancy, including lymphoma and leukemia* |  |  |  |  |  |
| *No* | 0.70 | 0.73 | 0.68 | 0.94 | 0.7724 |
| *Yes* | 0.43 | 0.45 | 0.42 | 0.94 | 0.9188 |
| *Moderate or severe liver disease* |  |  |  |  |  |
| *No* | 0.66 | 0.68 | 0.64 | 0.94 | 0.7591 |
| *Yes* | NA | NA | NA | NA | NA |
| *Metastatic solid tumor* |  |  |  |  |  |
| *No* | 0.66 | 0.68 | 0.64 | 0.93 | 0.7359 |
| *Yes* | 0.50 | NA | 1.00 | NA | NA |
| *HIV* |  |  |  |  |  |
| *No* | 0.66 | 0.68 | 0.64 | 0.94 | 0.7592 |
| *Yes* | NA | NA | NA | NA | NA |
| **Index year** |  |  |  |  |  |
| *2017* | 0.62 | 0.64 | 0.60 | 0.94 | 0.8427 |
| *2018* | 0.71 | 0.73 | 0.68 | 0.93 | 0.8112 |

6.5.3. Incidence rate of antibiotics & OCS-used COPD AE

6.5.3.1. Moderate exacerbation

| **MODERATE COPD EXACERBATION** | **All** | **Pre-BV** | **Post-BV** | **p-value** | **IRR** | **IRR-pvalue** |
| --- | --- | --- | --- | --- | --- | --- |
|  |  |  |  |  |  |  |
| ***Incidence rate per 1,000 PYs*** | 186.58 | 209.05 | 164.92 | 0.2900 | 0.79 | 0.2914 |
| *Patients with event* | 80 | 44 | 36 |  |  |  |
| *Sum of person years (PYs)* | 428.76 | 210.48 | 218.29 |  |  |  |
| *Time to event (KM estimated)* |  |  |  |  |  |  |
| *Median* | NA | NA | NA |  |  |  |
| *SE* | NA | NA | NA |  |  |  |
| *Time to event (descriptive)* |  |  |  |  |  |  |
| *Mean* | 153.04 | 141.89 | 166.67 |  |  |  |
| *SD* | 115.82 | 117.50 | 113.89 |  |  |  |
| *Median* | 121.50 | 104.00 | 176.50 |  |  |  |
| *Min* | 3.00 | 3.00 | 4.00 |  |  |  |
| *Max* | 364.00 | 364.00 | 363.00 |  |  |  |
| *P25* | 40.50 | 38.00 | 59.50 |  |  |  |
| *P75* | 247.00 | 247.00 | 245.50 |  |  |  |
| **COPD medication during pre and post-BV period** |  |  |  |  |  |  |
| *ICS/LABA* | 138.08 | 112.18 | 164.98 | 0.3023 | 1.47 | 0.3062 |
| *LAMA* | 42.49 | 42.53 | 42.46 | 0.9991 | 1.00 | 0.9991 |
| *Triple* | 330.05 | 465.81 | 214.59 | 0.0208 | 0.46 | 0.0244 |
| *LABA/LAMA* | 201.43 | 245.96 | 160.69 | 0.4636 | 0.65 | 0.4672 |
| **Age at index date 1** |  |  |  |  |  |  |
| *40 to < 65* | 221.15 | 263.30 | 181.52 | 0.3455 | 0.69 | 0.3488 |
| *65 to < 75* | 209.08 | 215.94 | 202.34 | 0.8350 | 0.94 | 0.8350 |
| *75+* | 112.94 | 142.20 | 84.97 | 0.3594 | 0.60 | 0.3664 |
| **Sex** |  |  |  |  |  |  |
| *Male* | 196.75 | 220.81 | 173.43 | 0.3241 | 0.79 | 0.3256 |
| *Female* | 147.35 | 163.12 | 132.42 | 0.7073 | 0.81 | 0.7078 |
| **Insurance type at index date2** |  |  |  |  |  |  |
| *Health insurance* | 193.01 | 199.72 | 186.40 | 0.7696 | 0.93 | 0.7697 |
| *Medical aid* | 143.55 | 277.61 | 32.77 | 0.0122 | 0.12 | 0.0456 |
| **Hospital type at index date2** |  |  |  |  |  |  |
| *Tertiary and general hospital* | 170.33 | 180.87 | 160.04 | 0.6329 | 0.88 | 0.6331 |
| *Others* | 268.98 | 357.62 | 188.77 | 0.1705 | 0.53 | 0.1791 |
| **History of COPD exacerbation** |  |  |  |  |  |  |
| *none* | 96.56 | 104.88 | 88.28 | 0.6735 | 0.84 | 0.6741 |
| *1 moderate* | 279.47 | 343.98 | 220.97 | 0.1591 | 0.64 | 0.1627 |
| *≥ 2 moderate OR ≥ 1 severe* | 447.61 | 417.77 | 477.45 | 0.7962 | 1.14 | 0.7964 |
| **History of Pneumonia** |  |  |  |  |  |  |
| *No* | 169.45 | 198.48 | 141.55 | 0.2166 | 0.71 | 0.2194 |
| *Yes* | 236.17 | 239.47 | 232.95 | 0.9439 | 0.97 | 0.9439 |
| **History of Asthma** |  |  |  |  |  |  |
| *No* | 156.41 | 202.40 | 111.90 | 0.3347 | 0.55 | 0.3444 |
| *Yes* | 192.50 | 210.36 | 175.30 | 0.4494 | 0.83 | 0.4501 |
| **mCCI** |  |  |  |  |  |  |
| *0, 1* | 247.96 | 274.01 | 223.06 | 0.4677 | 0.81 | 0.4685 |
| *2 +* | 132.09 | 151.87 | 112.87 | 0.4182 | 0.74 | 0.4205 |
| **mCCI category** |  |  |  |  |  |  |
| *Congestive heart failure* |  |  |  |  |  |  |
| *No* | 182.40 | 192.22 | 172.75 | 0.6622 | 0.90 | 0.6624 |
| *Yes* | 211.58 | 317.00 | 121.02 | 0.0938 | 0.38 | 0.1091 |
| *Dementia* |  |  |  |  |  |  |
| *No* | 186.90 | 210.14 | 164.54 | 0.2812 | 0.78 | 0.2826 |
| *Yes* | 174.97 | 170.84 | 179.31 | 0.9727 | 1.05 | 0.9727 |
| *Chronic pulmonary disease* |  |  |  |  |  |  |
| *No* | 149.02 | 198.72 | 99.33 | 0.5598 | 0.50 | 0.5713 |
| *Yes* | 188.43 | 209.57 | 168.09 | 0.3341 | 0.80 | 0.3352 |
| *Rheumatologic disease* |  |  |  |  |  |  |
| *No* | 187.18 | 210.95 | 164.31 | 0.2773 | 0.78 | 0.2788 |
| *Yes* | 175.92 | 175.86 | 175.98 | 0.9994 | 1.00 | 0.9994 |
| *Mild liver disease* |  |  |  |  |  |  |
| *No* | 198.20 | 235.83 | 162.54 | 0.1624 | 0.69 | 0.1653 |
| *Yes* | 162.92 | 155.93 | 169.90 | 0.8371 | 1.09 | 0.8371 |
| *Diabetes with chronic complications* |  |  |  |  |  |  |
| *No* | 217.47 | 250.34 | 186.33 | 0.2024 | 0.74 | 0.2043 |
| *Yes* | 59.61 | 46.84 | 72.85 | 0.6248 | 1.56 | 0.6285 |
| *Hemiplegia or paraplegia* |  |  |  |  |  |  |
| *No* | 186.84 | 212.06 | 162.54 | 0.2386 | 0.77 | 0.2403 |
| *Yes* | 168.01 | NA | 337.57 | NA | NA | NA |
| *Renal disease* |  |  |  |  |  |  |
| *No* | 188.44 | 209.55 | 168.12 | 0.3347 | 0.80 | 0.3358 |
| *Yes* | 148.90 | 199.16 | 98.96 | 0.5563 | 0.50 | 0.5680 |
| *Any malignancy, including lymphoma and leukemia* |  |  |  |  |  |  |
| *No* | 205.14 | 235.62 | 175.97 | 0.2139 | 0.75 | 0.2159 |
| *Yes* | 96.01 | 82.26 | 109.77 | 0.7042 | 1.33 | 0.7057 |
| *Moderate or severe liver disease* |  |  |  |  |  |  |
| *No* | 187.46 | 210.05 | 165.68 | 0.2897 | 0.79 | 0.2910 |
| *Yes* | NA | NA | NA | NA | NA | NA |
| *Metastatic solid tumor* |  |  |  |  |  |  |
| *No* | 184.94 | 210.05 | 160.78 | 0.2363 | 0.77 | 0.2380 |
| *Yes* | 626.50 | NA | 1667.81 | NA | NA | NA |
| *HIV* |  |  |  |  |  |  |
| *No* | 186.58 | 209.05 | 164.92 | 0.2900 | 0.79 | 0.2914 |
| *Yes* | NA | NA | NA | NA | NA | NA |
| **Index year** |  |  |  |  |  |  |
| *2017* | 173.93 | 221.01 | 129.21 | 0.0862 | 0.58 | 0.0911 |
| *2018* | 202.89 | 193.89 | 211.73 | 0.7863 | 1.09 | 0.7864 |

6.5.3.2. Severe exacerbation

| **SEVERE COPD EXACERBATION** | **All** | **Pre-BV** | **Post-BV** | **p-value** | **IRR** | **IRR-pvalue** |
| --- | --- | --- | --- | --- | --- | --- |
|  |  |  |  |  |  |  |
| ***Incidence rate per 1,000 PYs*** | 202.11 | 203.19 | 201.02 | 0.9602 | 0.99 | 0.9602 |
| *Patients with event* | 87 | 44 | 43 |  |  |  |
| *Sum of person years (PYs)* | 430.46 | 216.55 | 213.91 |  |  |  |
| *Time to event (KM estimated)* |  |  |  |  |  |  |
| *Median* | NA | NA | NA |  |  |  |
| *SE* | NA | NA | NA |  |  |  |
| *Time to event (descriptive)* |  |  |  |  |  |  |
| *Mean* | 177.18 | 192.27 | 161.74 |  |  |  |
| *SD* | 115.64 | 125.96 | 103.23 |  |  |  |
| *Median* | 161.00 | 198.00 | 149.00 |  |  |  |
| *Min* | 2.00 | 6.00 | 2.00 |  |  |  |
| *Max* | 364.00 | 364.00 | 357.00 |  |  |  |
| *P25* | 70.00 | 69.00 | 70.00 |  |  |  |
| *P75* | 298.00 | 319.50 | 238.00 |  |  |  |
| **COPD medication during pre and post-BV period** |  |  |  |  |  |  |
| *ICS/LABA* | 202.80 | 217.40 | 188.17 | 0.6444 | 0.87 | 0.6448 |
| *LAMA* | 42.40 | NA | 86.01 | NA | NA | NA |
| *Triple* | 260.09 | 263.43 | 256.62 | 0.9418 | 0.97 | 0.9418 |
| *LABA/LAMA* | 209.93 | 195.48 | 224.14 | 0.8055 | 1.15 | 0.8057 |
| **Age at index date 1** |  |  |  |  |  |  |
| *40 to < 65* | 180.48 | 217.13 | 145.09 | 0.3482 | 0.67 | 0.3526 |
| *65 to < 75* | 167.41 | 135.15 | 201.00 | 0.2506 | 1.49 | 0.2547 |
| *75+* | 293.95 | 320.23 | 267.30 | 0.6159 | 0.83 | 0.6167 |
| **Sex** |  |  |  |  |  |  |
| *Male* | 196.61 | 182.06 | 211.65 | 0.5349 | 1.16 | 0.5353 |
| *Female* | 224.60 | 294.26 | 159.77 | 0.1904 | 0.54 | 0.1991 |
| **Insurance type at index date2** |  |  |  |  |  |  |
| *Health insurance* | 193.77 | 189.72 | 197.88 | 0.8572 | 1.04 | 0.8573 |
| *Medical aid* | 260.56 | 298.50 | 222.80 | 0.5862 | 0.75 | 0.5881 |
| **Hospital type at index date2** |  |  |  |  |  |  |
| *Tertiary and general hospital* | 222.10 | 225.78 | 218.35 | 0.8826 | 0.97 | 0.8826 |
| *Others* | 113.55 | 101.56 | 125.39 | 0.7527 | 1.23 | 0.7534 |
| **History of COPD exacerbation** |  |  |  |  |  |  |
| *none* | 156.13 | 200.03 | 113.45 | 0.0858 | 0.57 | 0.0917 |
| *1 moderate* | 209.20 | 153.15 | 268.07 | 0.1188 | 1.75 | 0.1252 |
| *≥ 2 moderate OR ≥ 1 severe* | 498.42 | 439.27 | 566.18 | 0.6010 | 1.29 | 0.6014 |
| **History of Pneumonia** |  |  |  |  |  |  |
| *No* | 143.14 | 132.78 | 153.68 | 0.6167 | 1.16 | 0.6171 |
| *Yes* | 391.77 | 432.50 | 351.33 | 0.5120 | 0.81 | 0.5131 |
| **History of Asthma** |  |  |  |  |  |  |
| *No* | 66.74 | 53.21 | 80.38 | 0.6479 | 1.51 | 0.6513 |
| *Yes* | 230.63 | 234.69 | 226.52 | 0.8726 | 0.97 | 0.8726 |
| **mCCI** |  |  |  |  |  |  |
| *0, 1* | 179.25 | 146.62 | 213.86 | 0.2473 | 1.46 | 0.2506 |
| *2 +* | 224.29 | 260.64 | 189.12 | 0.2639 | 0.73 | 0.2665 |
| **mCCI category** |  |  |  |  |  |  |
| *Congestive heart failure* |  |  |  |  |  |  |
| *No* | 186.27 | 180.96 | 191.73 | 0.8103 | 1.06 | 0.8104 |
| *Yes* | 299.86 | 348.82 | 255.11 | 0.5079 | 0.73 | 0.5095 |
| *Dementia* |  |  |  |  |  |  |
| *No* | 202.37 | 203.29 | 201.44 | 0.9664 | 0.99 | 0.9664 |
| *Yes* | 191.63 | 198.83 | 184.94 | 0.9592 | 0.93 | 0.9592 |
| *Chronic pulmonary disease* |  |  |  |  |  |  |
| *No* | 91.40 | 91.20 | 91.61 | 0.9975 | 1.00 | 0.9975 |
| *Yes* | 208.04 | 209.16 | 206.90 | 0.9602 | 0.99 | 0.9602 |
| *Rheumatologic disease* |  |  |  |  |  |  |
| *No* | 206.27 | 209.99 | 202.51 | 0.8679 | 0.96 | 0.8679 |
| *Yes* | 129.16 | 84.88 | 174.72 | 0.5435 | 2.06 | 0.5556 |
| *Mild liver disease* |  |  |  |  |  |  |
| *No* | 190.93 | 182.09 | 199.96 | 0.7261 | 1.10 | 0.7262 |
| *Yes* | 226.03 | 249.01 | 203.25 | 0.5728 | 0.82 | 0.5737 |
| *Diabetes with chronic complications* |  |  |  |  |  |  |
| *No* | 217.64 | 209.58 | 225.87 | 0.7442 | 1.08 | 0.7443 |
| *Yes* | 135.38 | 174.96 | 96.98 | 0.3368 | 0.55 | 0.3465 |
| *Hemiplegia or paraplegia* |  |  |  |  |  |  |
| *No* | 197.36 | 196.16 | 198.58 | 0.9551 | 1.01 | 0.9551 |
| *Yes* | 619.77 | 821.71 | 415.53 | 0.5665 | 0.51 | 0.5777 |
| *Renal disease* |  |  |  |  |  |  |
| *No* | 199.74 | 198.63 | 200.86 | 0.9597 | 1.01 | 0.9597 |
| *Yes* | 250.93 | 295.91 | 204.34 | 0.6821 | 0.69 | 0.6850 |
| *Any malignancy, including lymphoma and leukemia* |  |  |  |  |  |  |
| *No* | 199.76 | 191.63 | 208.12 | 0.7262 | 1.09 | 0.7263 |
| *Yes* | 214.21 | 265.47 | 166.10 | 0.3683 | 0.63 | 0.3736 |
| *Moderate or severe liver disease* |  |  |  |  |  |  |
| *No* | 203.05 | 204.13 | 201.96 | 0.9604 | 0.99 | 0.9604 |
| *Yes* | NA | NA | NA | NA | NA | NA |
| *Metastatic solid tumor* |  |  |  |  |  |  |
| *No* | 203.05 | 204.13 | 201.96 | 0.9604 | 0.99 | 0.9604 |
| *Yes* | NA | NA | NA | NA | NA | NA |
| *HIV* |  |  |  |  |  |  |
| *No* | 202.11 | 203.19 | 201.02 | 0.9602 | 0.99 | 0.9602 |
| *Yes* | NA | NA | NA | NA | NA | NA |
| **Index year** |  |  |  |  |  |  |
| *2017* | 175.01 | 144.29 | 206.70 | 0.2415 | 1.43 | 0.2449 |
| *2018* | 238.15 | 283.22 | 193.63 | 0.2109 | 0.68 | 0.2149 |

6.5.3.3. Moderate-to-severe exacerbation

| **MODERATE TO SEVERE COPD EXACERBATION** | **All** | **Pre-BV** | **Post-BV** | **p-value** | **IRR** | **IRR-pvalue** |
| --- | --- | --- | --- | --- | --- | --- |
|  |  |  |  |  |  |  |
| ***Incidence rate per 1,000 PYs*** | 382.89 | 419.50 | 347.30 | 0.2481 | 0.83 | 0.2490 |
| *Patients with event* | 150 | 81 | 69 |  |  |  |
| *Sum of person years (PYs)* | 391.76 | 193.09 | 198.67 |  |  |  |
| *Time to event (KM estimated)* |  |  |  |  |  |  |
| *Median* | NA | NA | NA |  |  |  |
| *SE* | NA | NA | NA |  |  |  |
| *Time to event (descriptive)* |  |  |  |  |  |  |
| *Mean* | 161.61 | 164.95 | 157.70 |  |  |  |
| *SD* | 117.85 | 125.06 | 109.57 |  |  |  |
| *Median* | 145.00 | 126.00 | 150.00 |  |  |  |
| *Min* | 2.00 | 3.00 | 2.00 |  |  |  |
| *Max* | 364.00 | 364.00 | 363.00 |  |  |  |
| *P25* | 43.00 | 41.00 | 63.00 |  |  |  |
| *P75* | 255.00 | 298.00 | 238.00 |  |  |  |
| **COPD medication during pre and post-BV period** |  |  |  |  |  |  |
| *ICS/LABA* | 337.59 | 331.61 | 343.80 | 0.8853 | 1.04 | 0.8852 |
| *LAMA* | 86.33 | 42.53 | 131.46 | 0.2926 | 3.09 | 0.3284 |
| *Triple* | 597.95 | 763.15 | 451.39 | 0.0437 | 0.59 | 0.0463 |
| *LABA/LAMA* | 396.35 | 463.32 | 337.76 | 0.4592 | 0.73 | 0.4604 |
| **Age at index date 1** |  |  |  |  |  |  |
| *40 to < 65* | 386.23 | 484.65 | 297.41 | 0.1164 | 0.61 | 0.1203 |
| *65 to < 75* | 355.98 | 341.59 | 370.68 | 0.7399 | 1.09 | 0.7400 |
| *75+* | 430.27 | 501.86 | 361.51 | 0.2901 | 0.72 | 0.2928 |
| **Sex** |  |  |  |  |  |  |
| *Male* | 394.85 | 419.59 | 370.37 | 0.4893 | 0.88 | 0.4897 |
| *Female* | 336.45 | 419.14 | 261.44 | 0.2235 | 0.62 | 0.2282 |
| **Insurance type at index date2** |  |  |  |  |  |  |
| *Health insurance* | 378.92 | 397.95 | 360.03 | 0.5682 | 0.90 | 0.5685 |
| *Medical aid* | 410.86 | 585.26 | 264.48 | 0.0816 | 0.45 | 0.0902 |
| **Hospital type at index date2** |  |  |  |  |  |  |
| *Tertiary and general hospital* | 378.64 | 409.54 | 348.22 | 0.3691 | 0.85 | 0.3698 |
| *Others* | 403.49 | 469.80 | 342.99 | 0.4148 | 0.73 | 0.4166 |
| **History of COPD exacerbation** |  |  |  |  |  |  |
| *none* | 250.01 | 307.77 | 194.48 | 0.0836 | 0.63 | 0.0872 |
| *1 moderate* | 505.96 | 525.90 | 486.94 | 0.7528 | 0.93 | 0.7528 |
| *≥ 2 moderate OR ≥ 1 severe* | 914.04 | 815.59 | 1028.65 | 0.5619 | 1.26 | 0.5621 |
| **History of Pneumonia** |  |  |  |  |  |  |
| *No* | 316.74 | 352.03 | 282.49 | 0.2845 | 0.80 | 0.2857 |
| *Yes* | 598.95 | 639.17 | 559.67 | 0.6226 | 0.88 | 0.6229 |
| **History of Asthma** |  |  |  |  |  |  |
| *No* | 215.27 | 231.32 | 199.45 | 0.7743 | 0.86 | 0.7746 |
| *Yes* | 419.15 | 460.56 | 379.02 | 0.2584 | 0.82 | 0.2592 |
| **mCCI** |  |  |  |  |  |  |
| *0, 1* | 418.70 | 400.76 | 437.29 | 0.7001 | 1.09 | 0.7001 |
| *2 +* | 350.42 | 437.58 | 270.51 | 0.0430 | 0.62 | 0.0453 |
| **mCCI category** |  |  |  |  |  |  |
| *Congestive heart failure* |  |  |  |  |  |  |
| *No* | 353.33 | 362.76 | 343.78 | 0.7686 | 0.95 | 0.7687 |
| *Yes* | 575.41 | 856.87 | 367.12 | 0.0220 | 0.43 | 0.0253 |
| *Dementia* |  |  |  |  |  |  |
| *No* | 382.33 | 419.80 | 345.92 | 0.2429 | 0.82 | 0.2438 |
| *Yes* | 404.37 | 407.87 | 400.93 | 0.9863 | 0.98 | 0.9863 |
| *Chronic pulmonary disease* |  |  |  |  |  |  |
| *No* | 249.32 | 298.08 | 200.19 | 0.6596 | 0.67 | 0.6628 |
| *Yes* | 390.09 | 426.18 | 355.09 | 0.2725 | 0.83 | 0.2733 |
| *Rheumatologic disease* |  |  |  |  |  |  |
| *No* | 386.75 | 428.82 | 346.01 | 0.2004 | 0.81 | 0.2014 |
| *Yes* | 318.00 | 268.04 | 369.69 | 0.6721 | 1.38 | 0.6738 |
| *Mild liver disease* |  |  |  |  |  |  |
| *No* | 386.35 | 412.84 | 360.33 | 0.4925 | 0.87 | 0.4929 |
| *Yes* | 375.73 | 433.48 | 320.78 | 0.2986 | 0.74 | 0.3008 |
| *Diabetes with chronic complications* |  |  |  |  |  |  |
| *No* | 428.87 | 469.88 | 389.41 | 0.2775 | 0.83 | 0.2783 |
| *Yes* | 201.73 | 225.82 | 177.40 | 0.6308 | 0.79 | 0.6321 |
| *Hemiplegia or paraplegia* |  |  |  |  |  |  |
| *No* | 377.30 | 414.37 | 341.31 | 0.2420 | 0.82 | 0.2429 |
| *Yes* | 832.48 | 821.71 | 843.53 | 0.9791 | 1.03 | 0.9791 |
| *Renal disease* |  |  |  |  |  |  |
| *No* | 383.27 | 413.34 | 354.06 | 0.3551 | 0.86 | 0.3556 |
| *Yes* | 375.17 | 542.40 | 211.86 | 0.2366 | 0.39 | 0.2612 |
| *Any malignancy, including lymphoma and leukemia* |  |  |  |  |  |  |
| *No* | 401.54 | 436.66 | 367.10 | 0.3232 | 0.84 | 0.3240 |
| *Yes* | 294.07 | 335.57 | 255.46 | 0.5427 | 0.76 | 0.5439 |
| *Moderate or severe liver disease* |  |  |  |  |  |  |
| *No* | 384.85 | 421.68 | 349.06 | 0.2478 | 0.83 | 0.2486 |
| *Yes* | NA | NA | NA | NA | NA | NA |
| *Metastatic solid tumor* |  |  |  |  |  |  |
| *No* | 381.89 | 421.68 | 343.30 | 0.2103 | 0.81 | 0.2112 |
| *Yes* | 626.50 | NA | 1667.81 | NA | NA | NA |
| *HIV* |  |  |  |  |  |  |
| *No* | 382.89 | 419.50 | 347.30 | 0.2481 | 0.83 | 0.2490 |
| *Yes* | NA | NA | NA | NA | NA | NA |
| **Index year** |  |  |  |  |  |  |
| *2017* | 349.89 | 370.81 | 329.30 | 0.6002 | 0.89 | 0.6005 |
| *2018* | 426.46 | 484.74 | 370.74 | 0.2566 | 0.76 | 0.2583 |

6.5.4. Time to first antibiotics & OCS-used COPD AE

6.5.4.1 Moderate exacerbation


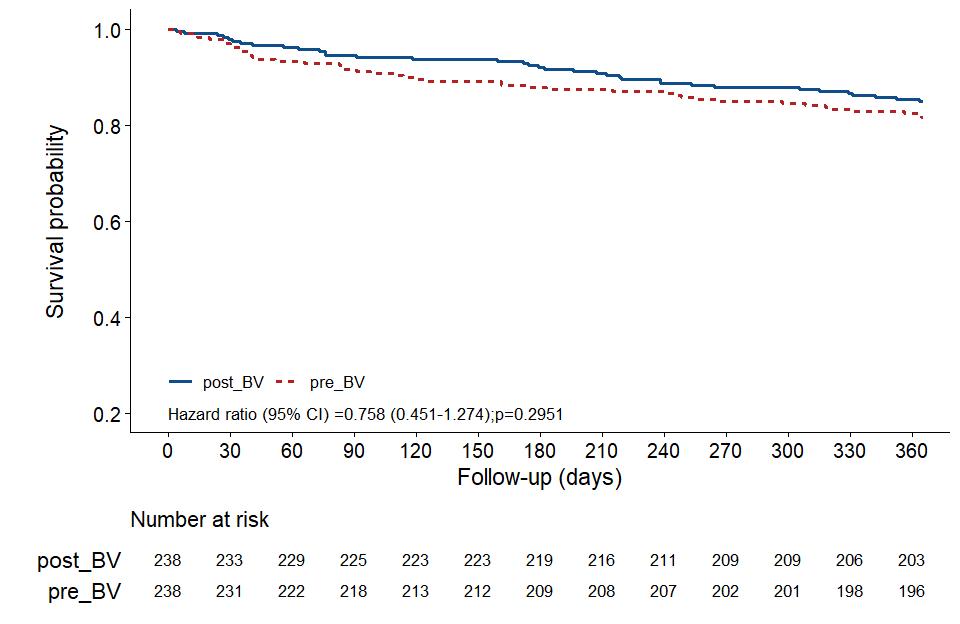


6.5.4.2. Severe exacerbation


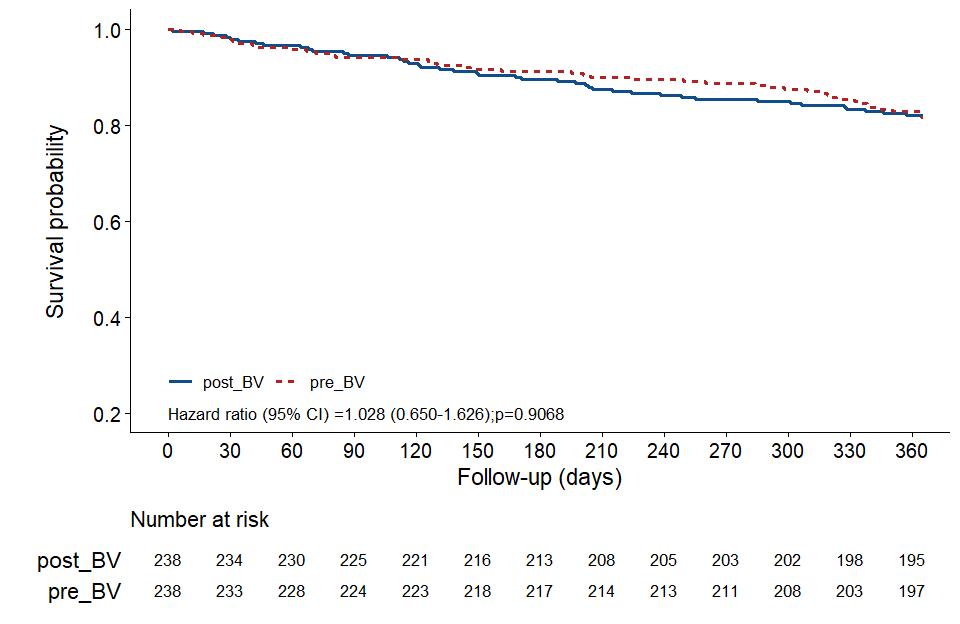


6.5.4.3. Moderate-to-severe exacerbation


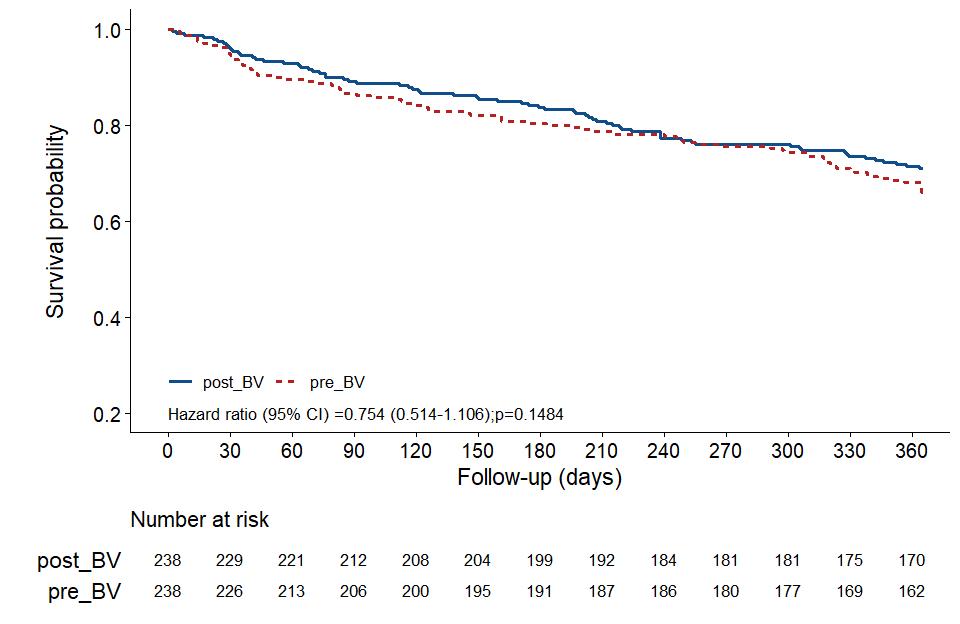


**7. Appendix 1 – Key definition**

## 7.1 Inclusion and exclusion criteria

**Table S7‑1 Relevant drug codes, diagnosis codes, and detailed operational definition for inclusion criteria**

| **Criteria** | **Class** | **Code** | **Detailed operational definition** |
| --- | --- | --- | --- |
| **Patients prescribed with ICS/LABA or LAMA during the selection period** | | | |
| **ICS/LABA** |  |  | ≥ 1 admission or out-patient visit with defined drug codes |
| salmeterol and fluticasone | Drug | 334500CSI |  |
| salmeterol and fluticasone | Drug | 334600CSI |  |
| salmeterol and fluticasone | Drug | 334700CSI |  |
| salmeterol and fluticasone | Drug | 407100CSI |  |
| salmeterol and fluticasone | Drug | 407200CSI |  |
| salmeterol and fluticasone | Drug | 407300CSI |  |
| salmeterol and fluticasone | Drug | 506400CSI |  |
| salmeterol and fluticasone | Drug | 506500CSI |  |
| salmeterol and fluticasone | Drug | 506600CSI |  |
| salmeterol and fluticasone | Drug | 543100CSI |  |
| salmeterol and fluticasone | Drug | 543200CSI |  |
| salmeterol and fluticasone | Drug | 543300CSI |  |
| salmeterol and fluticasone | Drug | 543400CSI |  |
| salmeterol and fluticasone | Drug | 543500CSI |  |
| salmeterol and fluticasone | Drug | 543600CSI |  |
| salmeterol and fluticasone | Drug | 544300CSI |  |
| salmeterol and fluticasone | Drug | 544400CSI |  |
| salmeterol and fluticasone | Drug | 544500CSI |  |
| formoterol and budesonide | Drug | 543800CSI |  |
| formoterol and budesonide | Drug | 543900CSI |  |
| formoterol and budesonide | Drug | 544000CSI |  |
| formoterol and budesonide | Drug | 544100CSI |  |
| formoterol and budesonide | Drug | 801100CSI |  |
| formoterol and budesonide | Drug | 391800CSI |  |
| formoterol and budesonide | Drug | 441700COS |  |
| formoterol and budesonide | Drug | 441700CSI |  |
| formoterol and budesonide | Drug | 453400CSI |  |
| formoterol and budesonide | Drug | 640400CSI |  |
| formoterol and beclometasone | Drug | 502000CSI |  |
| formoterol and beclometasone | Drug | 544200CSI |  |
| vilanterol and fluticasone furoate | Drug | 636700CSI |  |
| vilanterol and fluticasone furoate | Drug | 636800CSI |  |
| formoterol and fluticasone | Drug | 525700CSI |  |
| formoterol and fluticasone | Drug | 525800CSI |  |
| formoterol and fluticasone | Drug | 526200CSI |  |
| formoterol and fluticasone | Drug | 542800CSI |  |
| formoterol and fluticasone | Drug | 542900CSI |  |
| formoterol and fluticasone | Drug | 543000CSI |  |
| salmeterol and budesonide | Drug | 681000CSI |  |
| salmeterol and budesonide | Drug | 681100CSI |  |
| **LAMA** |  |  | ≥ 1 admission or out-patient visit with defined drug codes |
| tiotropium bromide | Drug | 457301CCH |  |
| tiotropium bromide | Drug | 457301CPK |  |
| tiotropium bromide | Drug | 457301CSI |  |
| tiotropium bromide | Drug | 457330CSI |  |
| tiotropium bromide | Drug | 503401CSI |  |
| tiotropium bromide | Drug | 503430CSI |  |
| aclidinium bromide | Drug | 633701CSI |  |
| aclidinium bromide | Drug | 633730CSI |  |
| glycopyrronium bromide | Drug | 635903CSI |  |
| umeclidinium bromide | Drug | 641101CSI |  |
| **Patients diagnosed with COPD during the selection period** | | | |
| **COPD** |  |  | ≥ 1 admissions or out-patient visits with defined diagnosis codes as any diagnosis in in-patient claims or primary-5^th^ diagnosis in out-patient claims |
| *Applied to the data from 2005/1/1 to 2007/12/31 (KCD-4 version)* |  |  |  |
| Emphysema | Diagnosis | J43 |  |
| Panlobular emphysema | Diagnosis | J43.1 |  |
| Centrilobular emphysema | Diagnosis | J43.2 |  |
| Other emphysema | Diagnosis | J43.8 |  |
| Emphysema, unspecified | Diagnosis | J43.9 |  |
| Other chronic obstructive pulmonary disease | Diagnosis | J44 |  |
| Chronic obstructive pulmonary disease with acute lower respiratory infection | Diagnosis | J44.0 |  |
| Chronic obstructive pulmonary disease with acute exacerbation, unspecified | Diagnosis | J44.1 |  |
| Other specified chronic obstructive pulmonary disease | Diagnosis | J44.8 |  |
| Chronic obstructive pulmonary disease, unspecified | Diagnosis | J44.9 |  |
| Applied to the data from 2008/1/1 to 2010/12/31 (KCD-5 version) |  |  |  |
| Emphysema | Diagnosis | J43 |  |
| Panlobular emphysema | Diagnosis | J43.1 |  |
| Centrilobular emphysema | Diagnosis | J43.2 |  |
| Other emphysema | Diagnosis | J43.8 |  |
| Emphysema, unspecified | Diagnosis | J43.9 |  |
| Other chronic obstructive pulmonary disease | Diagnosis | J44 |  |
| Chronic obstructive pulmonary disease with acute lower respiratory infection | Diagnosis | J44.0 |  |
| Chronic obstructive pulmonary disease with acute exacerbation, unspecified | Diagnosis | J44.1 |  |
| Other specified chronic obstructive pulmonary disease | Diagnosis | J44.8 |  |
| Chronic obstructive pulmonary disease, unspecified | Diagnosis | J44.9 |  |
| *Applied to the data from 2011/1/1 to 2015/12/31 (KCD-6 version)* |  |  |  |
| Emphysema | Diagnosis | J43 |  |
| Panlobular emphysema | Diagnosis | J43.1 |  |
| Centrilobular emphysema | Diagnosis | J43.2 |  |
| Other emphysema | Diagnosis | J43.8 |  |
| Emphysema, unspecified | Diagnosis | J43.9 |  |
| Other chronic obstructive pulmonary disease | Diagnosis | J44 |  |
| Chronic obstructive pulmonary disease with acute lower respiratory infection | Diagnosis | J44.0 |  |
| Chronic obstructive pulmonary disease with acute exacerbation, unspecified | Diagnosis | J44.1 |  |
| Other specified chronic obstructive pulmonary disease | Diagnosis | J44.8 |  |
| Chronic obstructive pulmonary disease, unspecified | Diagnosis | J44.9 |  |
| *Applied to the data from 2016/1/1 to 2019/12/31 (KCD-7 version)* |  |  |  |
| Emphysema | Diagnosis | J43 |  |
| Panlobular emphysema | Diagnosis | J43.1 |  |
| Centrilobular emphysema | Diagnosis | J43.2 |  |
| Other emphysema | Diagnosis | J43.8 |  |
| Emphysema, unspecified | Diagnosis | J43.9 |  |
| Other chronic obstructive pulmonary disease | Diagnosis | J44 |  |
| Chronic obstructive pulmonary disease with acute lower respiratory infection | Diagnosis | J44.0 |  |
| Chronic obstructive pulmonary disease with acute exacerbation, unspecified | Diagnosis | J44.1 |  |
| Other specified chronic obstructive pulmonary disease | Diagnosis | J44.8 |  |
| Chronic obstructive pulmonary disease, unspecified | Diagnosis | J44.9 |  |

**Table S7‑2 Relevant diagnosis codes, drug codes, and detailed operational definition for exclusion criteria**

| **Criteria** | **Class** | **Code** | **Detailed operational definition** |
| --- | --- | --- | --- |
| **Patients with prescribed with Uro-Vaxom® or Ismigen® anytime during the baseline period or follow-up period** | | | |
| **Uro-Vaxom®** |  |  | ≥ 1 admission or out-patient visit with defined drug codes |
| Other immunostimulants | Drug | 235101ACH |  |
| **Ismigen®** |  |  |  |
| Other immunostimulants | Drug | 186703ATL |  |
| **Patients diagnosed with lung cancer or had undergone lung transplantation during the baseline period or follow-up period** | | | |
| **Lung cancer** |  |  | ≥ 1 admission or out-patient visit with defined diagnosis codes as any diagnosis in in-patient claims or primary-5^th^ diagnosis in out-patient claims AND with special benefit codes for patients with cancers |
| *Applied to the data from 2005/1/1 to 2007/12/31 (KCD-4 version)* |  |  |  |
| Malignant neoplasm of main bronchus | Diagnosis | C34.0 |  |
| Malignant neoplasm upper lobe bronchus or lung | Diagnosis | C34.1 |  |
| Malignant neoplasm of middle lobe, bronchus or lung | Diagnosis | C34.2 |  |
| Malignant neoplasm of lower lobe, bronchus or lung | Diagnosis | C34.3 |  |
| Overlap malignant lesion bronchus or lung | Diagnosis | C34.8 |  |
| Malignant neoplasm of bronchus or lung | Diagnosis | C34.9 |  |
| *Applied to the data from 2008/1/1 to 2010/12/31 (KCD-5 version)* |  |  |  |
| Malignant neoplasm of main bronchus | Diagnosis | C34.0 |  |
| Malignant neoplasm upper lobe bronchus or lung | Diagnosis | C34.1 |  |
| Malignant neoplasm of middle lobe, bronchus or lung | Diagnosis | C34.2 |  |
| Malignant neoplasm of lower lobe, bronchus or lung | Diagnosis | C34.3 |  |
| Overlap malignant lesion bronchus or lung | Diagnosis | C34.8 |  |
| Malignant neoplasm of bronchus or lung | Diagnosis | C34.9 |  |
| *Applied to the data from 2011/1/1 to 2015/12/31 (KCD-6 version)* |  |  |  |
| Malignant neoplasm of hilus (of lung) | Diagnosis | C34.0 |  |
| Malignant neoplasm of main bronchus, right | Diagnosis | C34.00 |  |
| Malignant neoplasm of main bronchus, left | Diagnosis | C34.01 |  |
| Malignant neoplasm of main bronchus, unspecified side | Diagnosis | C34.09 |  |
| Malignant neoplasm of upper lobe, bronchus or lung | Diagnosis | C34.1 |  |
| Malignant neoplasm of upper lobe, bronchus or lung, right | Diagnosis | C34.10 |  |
| Malignant neoplasm of upper lobe, bronchus or lung, left | Diagnosis | C34.11 |  |
| Malignant neoplasm of upper lobe, bronchus or lung, unspecified side | Diagnosis | C34.19 |  |
| Malignant neoplasm of middle lobe, bronchus or lung | Diagnosis | C34.2 |  |
| Malignant neoplasm of lower lobe, bronchus or lung | Diagnosis | C34.3 |  |
| Malignant neoplasm of lower lobe, bronchus or lung, right | Diagnosis | C34.30 |  |
| Malignant neoplasm of lower lobe, bronchus or lung, left | Diagnosis | C34.31 |  |
| Malignant neoplasm of lower lobe, bronchus or lung, unspecified side | Diagnosis | C34.39 |  |
| Overlap malignant lesion bronchus or lung | Diagnosis | C34.8 |  |
| Malignant neoplasm of overlapping lesion of bronchus and lung, right | Diagnosis | C34.80 |  |
| Malignant neoplasm of overlapping lesion of bronchus and lung, left | Diagnosis | C34.81 |  |
| Malignant neoplasm of overlapping lesion of bronchus and lung, unspecified side | Diagnosis | C34.89 |  |
| Malignant neoplasm of bronchus or lung, unspecified | Diagnosis | C34.9 |  |
| Malignant neoplasm of bronchus or lung, unspecified, right | Diagnosis | C34.90 |  |
| Malignant neoplasm of bronchus or lung, unspecified, left | Diagnosis | C34.91 |  |
| Malignant neoplasm of bronchus or lung, unspecified, unspecified side | Diagnosis | C34.99 |  |
| *Applied to the data from 2016/1/1 to 2019/12/31 (KCD-7 version)* |  |  |  |
| Malignant neoplasm of bronchus and lung | Diagnosis | C34 |  |
| Malignant neoplasm of hilus (of lung) | Diagnosis | C34.0 |  |
| Malignant neoplasm of main bronchus, right | Diagnosis | C34.00 |  |
| Malignant neoplasm of main bronchus, left | Diagnosis | C34.01 |  |
| Malignant neoplasm of main bronchus, unspecified side | Diagnosis | C34.09 |  |
| Malignant neoplasm of upper lobe, bronchus or lung | Diagnosis | C34.1 |  |
| Malignant neoplasm of upper lobe, bronchus or lung, right | Diagnosis | C34.10 |  |
| Malignant neoplasm of upper lobe, bronchus or lung, left | Diagnosis | C34.11 |  |
| Malignant neoplasm of upper lobe, bronchus or lung, unspecified side | Diagnosis | C34.19 |  |
| Malignant neoplasm of middle lobe, bronchus or lung | Diagnosis | C34.2 |  |
| Malignant neoplasm of middle lobe, bronchus or lung, right | Diagnosis | C34.20 |  |
| Malignant neoplasm of middle lobe, bronchus or lung, left | Diagnosis | C34.21 |  |
| Malignant neoplasm of middle lobe, bronchus or lung, unspecified side | Diagnosis | C34.29 |  |
| Malignant neoplasm of lower lobe, bronchus or lung | Diagnosis | C34.3 |  |
| Malignant neoplasm of lower lobe, bronchus or lung, right | Diagnosis | C34.30 |  |
| Malignant neoplasm of lower lobe, bronchus or lung, left | Diagnosis | C34.31 |  |
| Malignant neoplasm of lower lobe, bronchus or lung, unspecified side | Diagnosis | C34.39 |  |
| Overlap malignant lesion bronchus or lung | Diagnosis | C34.8 |  |
| Malignant neoplasm of overlapping lesion of bronchus and lung, right | Diagnosis | C34.80 |  |
| Malignant neoplasm of overlapping lesion of bronchus and lung, left | Diagnosis | C34.81 |  |
| Malignant neoplasm of overlapping lesion of bronchus and lung, unspecified side | Diagnosis | C34.89 |  |
| Malignant neoplasm of bronchus or lung, unspecified | Diagnosis | C34.9 |  |
| Malignant neoplasm of bronchus or lung, unspecified, right | Diagnosis | C34.90 |  |
| Malignant neoplasm of bronchus or lung, unspecified, left | Diagnosis | C34.91 |  |
| Malignant neoplasm of bronchus or lung, unspecified, unspecified side | Diagnosis | C34.99 |  |
| **Special benefit codes for cancer patients** |  |  |  |
| Cancer benefit code | Benefit code | V193 |  |
| Cancer benefit code | Benefit code | V194 |  |
| Cancer benefit code | Benefit code | V027 |  |
| **Lung transplantation** |  |  | ≥ 1 admission or out-patient visit with defined diagnosis codes as any diagnosis in in-patient claims or primary-5th diagnosis in out-patient claims OR  ≥1 admission or outpatient claims with defined procedure codes |
| *Applied to the data from 2005/1/1 to 2007/12/31 (KCD-4 version)* |  |  |  |
| Lung transplant status | Diagnosis | Z94.2 |  |
| *Applied to the data from 2008/1/1 to 2010/12/31 (KCD-5 version)* |  |  |  |
| Lung transplant status | Diagnosis | Z94.2 |  |
| *Applied to the data from 2011/1/1 to 2015/12/31 (KCD-6 version)* |  |  |  |
| Lung transplant status | Diagnosis | Z94.2 |  |
| *Applied to the data from 2016/1/1 to 2019/12/31 (KCD-7 version)* |  |  |  |
| Lung transplant status | Diagnosis | Z94.2 |  |
| Lung transplantation | Procedure | Q8091.x |  |
| Lung transplantation | Procedure | Q8092.x |  |
| Lung transplantation | Procedure | Q8101.x |  |
| Lung transplantation | Procedure | Q8102.x |  |
| Lung transplantation | Procedure | Q8103.x |  |
| **Patients diagnosed with idiopathic pulmonary fibrosis (IPF) or interstitial lung disease (ILD) during the baseline period including the events occurred on the index date** | | | |
| **IPF** |  |  | ≥ 1 admission or out-patient visit with defined diagnosis codes as any diagnosis in in-patient claims or primary-5^th^ diagnosis in out-patient claims |
| *Applied to the data from 2005/1/1 to 2007/12/31 (KCD-4 version)* |  |  |  |
| Alveolar and parietoalveolar conditions | Diagnosis | J84.0 |  |
| Other interstitial pulmonary diseases with fibrosis | Diagnosis | J84.1 |  |
| Other specified interstitial pulmonary diseases | Diagnosis | J84.8 |  |
| Interstitial pulmonary disease, unspecified | Diagnosis | J84.9 |  |
| *Applied to the data from 2008/1/1 to 2010/12/31 (KCD-5 version)* |  |  |  |
| Alveolar and parietoalveolar conditions | Diagnosis | J84.0 |  |
| Other interstitial pulmonary diseases with fibrosis | Diagnosis | J84.1 |  |
| Other specified interstitial pulmonary diseases | Diagnosis | J84.8 |  |
| Interstitial pulmonary disease, unspecified | Diagnosis | J84.9 |  |
| *Applied to the data from 2011/1/1 to 2015/12/31 (KCD-6 version)* |  |  |  |
| Other interstitial pulmonary diseases | Diagnosis | J84 |  |
| Alveolar and parietoalveolar conditions | Diagnosis | J84.0 |  |
| Other interstitial pulmonary diseases with fibrosis | Diagnosis | J84.1 |  |
| Lymphoid interstitial pneumonia | Diagnosis | J84.10 |  |
| Other interstitial pulmonary disease with fibrosis | Diagnosis | J84.18 |  |
| Other specified interstitial pulmonary diseases | Diagnosis | J84.8 |  |
| Interstitial pulmonary disease, unspecified | Diagnosis | J84.9 |  |
| *Applied to the data from 2016/1/1 to 2019/12/31 (KCD-7 version)* |  |  |  |
| Other interstitial pulmonary diseases | Diagnosis | J84 |  |
| Alveolar and parietoalveolar conditions | Diagnosis | J84.0 |  |
| Other interstitial pulmonary diseases with fibrosis | Diagnosis | J84.1 |  |
| Lymphoid interstitial pneumonia | Diagnosis | J84.10 |  |
| Other interstitial pulmonary disease with fibrosis | Diagnosis | J84.18 |  |
| **ILD** |  |  | ≥ 1 admission or out-patient visit with defined diagnosis codes as any diagnosis in in-patient claims or primary-5^th^ diagnosis in out-patient claims |
| *Applied to the data from 2005/1/1 to 2007/12/31 (KCD-4 version)* |  |  |  |
| Acute drug-induced interstitial lung disorders | Diagnosis | J70.2 |  |
| Chronic drug-induced interstitial lung disorders | Diagnosis | J70.3 |  |
| Drug-induced interstitial lung disorders, unspecified | Diagnosis | J70.4 |  |
| *Applied to the data from 2008/1/1 to 2010/12/31 (KCD-5 version)* |  |  |  |
| Acute drug-induced interstitial lung disorders | Diagnosis | J70.2 |  |
| Chronic drug-induced interstitial lung disorders | Diagnosis | J70.3 |  |
| Drug-induced interstitial lung disorders, unspecified | Diagnosis | J70.4 |  |
| *Applied to the data from 2011/1/1 to 2015/12/31 (KCD-6 version)* |  |  |  |
| Acute drug-induced interstitial lung disorders | Diagnosis | J70.2 |  |
| Chronic drug-induced interstitial lung disorders | Diagnosis | J70.3 |  |
| Drug-induced interstitial lung disorders, unspecified | Diagnosis | J70.4 |  |
| *Applied to the data from 2016/1/1 to 2019/12/31 (KCD-7 version)* |  |  |  |
| Acute drug-induced interstitial lung disorders | Diagnosis | J70.2 |  |
| Chronic drug-induced interstitial lung disorders | Diagnosis | J70.3 |  |
| Drug-induced interstitial lung disorders, unspecified | Diagnosis | J70.4 |  |
| **Pateints prescribed with Broncho-Vaxom® during the 12-month period prior to the index date excluding the index date** | | | |
| **Broncho-Vaxom®** |  |  | ≥ 1 admission or out-patient visit with defined drug codes |
| Other immunostimulants | Drug | 186701ACH |  |
| Other immunostimulants | Drug | 186702ACH |  |
| **Patients had died during the 12-month period following the index date including the index date** | | | |
| All-cause death | Date | Date of death | Patients who had the date of death information |
| **Patients with prescribed triple combination therapy or LABA/LAMA during the 12-month period following the index date** | | | |
| **Triple combination therapy** |  |  | ≥ 1 admission or out-patient visit with defined drug codes  with the combination of ICS/LABA+LAMA issued on the same date in the same prescription |
| **ICS/LABA** |  |  |  |
| ipratropium bromide | Drug | 177101CLQ |  |
| salmeterol and fluticasone | Drug | 334500CSI |  |
| salmeterol and fluticasone | Drug | 334600CSI |  |
| salmeterol and fluticasone | Drug | 334700CSI |  |
| salmeterol and fluticasone | Drug | 407100CSI |  |
| salmeterol and fluticasone | Drug | 407200CSI |  |
| salmeterol and fluticasone | Drug | 407300CSI |  |
| salmeterol and fluticasone | Drug | 506400CSI |  |
| salmeterol and fluticasone | Drug | 506500CSI |  |
| salmeterol and fluticasone | Drug | 506600CSI |  |
| salmeterol and fluticasone | Drug | 543100CSI |  |
| salmeterol and fluticasone | Drug | 543200CSI |  |
| salmeterol and fluticasone | Drug | 543300CSI |  |
| salmeterol and fluticasone | Drug | 543400CSI |  |
| salmeterol and fluticasone | Drug | 543500CSI |  |
| salmeterol and fluticasone | Drug | 543600CSI |  |
| salmeterol and fluticasone | Drug | 544300CSI |  |
| salmeterol and fluticasone | Drug | 544400CSI |  |
| salmeterol and fluticasone | Drug | 544500CSI |  |
| formoterol and budesonide | Drug | 543800CSI |  |
| formoterol and budesonide | Drug | 543900CSI |  |
| formoterol and budesonide | Drug | 544000CSI |  |
| formoterol and budesonide | Drug | 544100CSI |  |
| formoterol and budesonide | Drug | 801100CSI |  |
| formoterol and budesonide | Drug | 391800CSI |  |
| formoterol and budesonide | Drug | 441700COS |  |
| formoterol and budesonide | Drug | 441700CSI |  |
| formoterol and budesonide | Drug | 453400CSI |  |
| formoterol and budesonide | Drug | 640400CSI |  |
| formoterol and beclometasone | Drug | 502000CSI |  |
| formoterol and beclometasone | Drug | 544200CSI |  |
| vilanterol and fluticasone furoate | Drug | 636700CSI |  |
| vilanterol and fluticasone furoate | Drug | 636800CSI |  |
| formoterol and fluticasone | Drug | 525700CSI |  |
| formoterol and fluticasone | Drug | 525800CSI |  |
| formoterol and fluticasone | Drug | 526200CSI |  |
| formoterol and fluticasone | Drug | 542800CSI |  |
| formoterol and fluticasone | Drug | 542900CSI |  |
| formoterol and fluticasone | Drug | 543000CSI |  |
| salmeterol and budesonide | Drug | 681000CSI |  |
| salmeterol and budesonide | Drug | 681100CSI |  |
| **LAMA** |  |  |  |
| tiotropium bromide | Drug | 457301CCH |  |
| tiotropium bromide | Drug | 457301CPK |  |
| tiotropium bromide | Drug | 457301CSI |  |
| tiotropium bromide | Drug | 457330CSI |  |
| tiotropium bromide | Drug | 503401CSI |  |
| tiotropium bromide | Drug | 503430CSI |  |
| aclidinium bromide | Drug | 633701CSI |  |
| aclidinium bromide | Drug | 633730CSI |  |
| glycopyrronium bromide | Drug | 635903CSI |  |
| umeclidinium bromide | Drug | 641101CSI |  |
| **LABA/LAMA** |  |  | ≥1 admission or out-patient visit with LABA/LAMA OR the combination of LABA + LAMA issued on the same date in the same prescription |
| vilanterol and umeclidinium bromide | Drug | 631200CSI |  |
| indacaterol and glycopyrronium bromide | Drug | 627500CSI |  |
| indacaterol and glycopyrronium bromide | Drug | 800100CSI |  |
| formoterol and aclidinium bromide | Drug | 635300CSI |  |
| olodaterol and tiotropium bromide | Drug | 643700CSI |  |
| **LABA** |  |  |  |
| salmeterol | Drug | 225801CSI |  |
| salmeterol | Drug | 225802CSI |  |
| formoterol | Drug | 163101ATB |  |
| formoterol | Drug | 163102CSI |  |
| formoterol | Drug | 163104ATB |  |
| formoterol | Drug | 163105CSI |  |
| formoterol | Drug | 163130ASY |  |
| formoterol | Drug | 163131ASY |  |
| indacaterol | Drug | 611901CSI |  |
| indacaterol | Drug | 611902CSI |  |
| **LAMA** |  |  |  |
| tiotropium bromide | Drug | 457301CCH |  |
| tiotropium bromide | Drug | 457301CPK |  |
| tiotropium bromide | Drug | 457301CSI |  |
| tiotropium bromide | Drug | 457330CSI |  |
| tiotropium bromide | Drug | 503401CSI |  |
| tiotropium bromide | Drug | 503430CSI |  |
| aclidinium bromide | Drug | 633701CSI |  |
| aclidinium bromide | Drug | 633730CSI |  |
| glycopyrronium bromide | Drug | 635903CSI |  |
| umeclidinium bromide | Drug | 641101CSI |  |
| **Patients with prescribed ICS/LABA combination therapy for LAMA-only group or LAMA monotherapy for ICS/LABA-only group during the 12-month period following the index date.** | | | |
| **LAMA** |  |  | ≥ 1 admission or out-patient visit with defined drug codes |
| tiotropium bromide | Drug | 457301CCH |  |
| tiotropium bromide | Drug | 457301CPK |  |
| tiotropium bromide | Drug | 457301CSI |  |
| tiotropium bromide | Drug | 457330CSI |  |
| tiotropium bromide | Drug | 503401CSI |  |
| tiotropium bromide | Drug | 503430CSI |  |
| aclidinium bromide | Drug | 633701CSI |  |
| aclidinium bromide | Drug | 633730CSI |  |
| glycopyrronium bromide | Drug | 635903CSI |  |
| umeclidinium bromide | Drug | 641101CSI |  |
| **ICS/LABA** |  |  | ≥ 1 admission or out-patient visit with defined drug codes |
| ipratropium bromide | Drug | 177101CLQ |  |
| salmeterol and fluticasone | Drug | 334500CSI |  |
| salmeterol and fluticasone | Drug | 334600CSI |  |
| salmeterol and fluticasone | Drug | 334700CSI |  |
| salmeterol and fluticasone | Drug | 407100CSI |  |
| salmeterol and fluticasone | Drug | 407200CSI |  |
| salmeterol and fluticasone | Drug | 407300CSI |  |
| salmeterol and fluticasone | Drug | 506400CSI |  |
| salmeterol and fluticasone | Drug | 506500CSI |  |
| salmeterol and fluticasone | Drug | 506600CSI |  |
| salmeterol and fluticasone | Drug | 543100CSI |  |
| salmeterol and fluticasone | Drug | 543200CSI |  |
| salmeterol and fluticasone | Drug | 543300CSI |  |
| salmeterol and fluticasone | Drug | 543400CSI |  |
| salmeterol and fluticasone | Drug | 543500CSI |  |
| salmeterol and fluticasone | Drug | 543600CSI |  |
| salmeterol and fluticasone | Drug | 544300CSI |  |
| salmeterol and fluticasone | Drug | 544400CSI |  |
| salmeterol and fluticasone | Drug | 544500CSI |  |
| formoterol and budesonide | Drug | 543800CSI |  |
| formoterol and budesonide | Drug | 543900CSI |  |
| formoterol and budesonide | Drug | 544000CSI |  |
| formoterol and budesonide | Drug | 544100CSI |  |
| formoterol and budesonide | Drug | 801100CSI |  |
| formoterol and budesonide | Drug | 391800CSI |  |
| formoterol and budesonide | Drug | 441700COS |  |
| formoterol and budesonide | Drug | 441700CSI |  |
| formoterol and budesonide | Drug | 453400CSI |  |
| formoterol and budesonide | Drug | 640400CSI |  |
| formoterol and beclometasone | Drug | 502000CSI |  |
| formoterol and beclometasone | Drug | 544200CSI |  |
| vilanterol and fluticasone furoate | Drug | 636700CSI |  |
| vilanterol and fluticasone furoate | Drug | 636800CSI |  |
| formoterol and fluticasone | Drug | 525700CSI |  |
| formoterol and fluticasone | Drug | 525800CSI |  |
| formoterol and fluticasone | Drug | 526200CSI |  |
| formoterol and fluticasone | Drug | 542800CSI |  |
| formoterol and fluticasone | Drug | 542900CSI |  |
| formoterol and fluticasone | Drug | 543000CSI |  |
| salmeterol and budesonide | Drug | 681000CSI |  |
| salmeterol and budesonide | Drug | 681100CSI |  |

## 7.2. Exposures

**Table S7‑3 Relevant drug codes for study drugs**

| **Criteria** | **Class** | **Code** |
| --- | --- | --- |
| **Broncho-Vaxom®** |  |  |
| Other immunostimulants | Drug | 186701ACH |
| Other immunostimulants | Drug | 186702ACH |

## 7.3. Primary outcomes

**Table S7‑4 Relevant diagnosis codes, drug codes, and detailed operational definition for COPD exacerbation**

| **Operational definition** | **Class** | | **Code** | **Detailed operational definition** |
| --- | --- | --- | --- | --- |
| Moderate COPD exacerbation |  | |  | ≥ 1 out-patient visit with defined diagnosis codes as primary-5^th^ diagnosis AND with defined drug codes for systemic steroid or antibiotics during 12 months after the index date |
| COPD |  | |  |  |
| *Applied to the data from 2005/1/1 to 2007/12/31 (KCD-4 version)* |  | |  |  |
| Emphysema | Diagnosis | | J43 |  |
| Panlobular emphysema | Diagnosis | | J43.1 |  |
| Centrilobular emphysema | Diagnosis | | J43.2 |  |
| Other emphysema | Diagnosis | | J43.8 |  |
| Emphysema, unspecified | Diagnosis | | J43.9 |  |
| Other chronic obstructive pulmonary disease | Diagnosis | | J44 |  |
| Chronic obstructive pulmonary disease with acute lower respiratory infection | Diagnosis | | J44.0 |  |
| Chronic obstructive pulmonary disease with acute exacerbation, unspecified | Diagnosis | | J44.1 |  |
| Other specified chronic obstructive pulmonary disease | Diagnosis | | J44.8 |  |
| Chronic obstructive pulmonary disease, unspecified | Diagnosis | | J44.9 |  |
| *Applied to the data from 2008/1/1 to 2010/12/31 (KCD-5 version)* |  | |  |  |
| Emphysema | Diagnosis | | J43 |  |
| Panlobular emphysema | Diagnosis | | J43.1 |  |
| Centrilobular emphysema | Diagnosis | | J43.2 |  |
| Other emphysema | Diagnosis | | J43.8 |  |
| Emphysema, unspecified | Diagnosis | | J43.9 |  |
| Other chronic obstructive pulmonary disease | Diagnosis | | J44 |  |
| Chronic obstructive pulmonary disease with acute lower respiratory infection | Diagnosis | | J44.0 |  |
| Chronic obstructive pulmonary disease with acute exacerbation, unspecified | Diagnosis | | J44.1 |  |
| Other specified chronic obstructive pulmonary disease | Diagnosis | | J44.8 |  |
| Chronic obstructive pulmonary disease, unspecified | Diagnosis | | J44.9 |  |
| *Applied to the data from 2011/1/1 to 2015/12/31 (KCD-6 version)* |  | |  |  |
| Emphysema | Diagnosis | | J43 |  |
| Panlobular emphysema | Diagnosis | | J43.1 |  |
| Centrilobular emphysema | Diagnosis | | J43.2 |  |
| Other emphysema | Diagnosis | | J43.8 |  |
| Emphysema, unspecified | Diagnosis | | J43.9 |  |
| Other chronic obstructive pulmonary disease | Diagnosis | | J44 |  |
| Chronic obstructive pulmonary disease with acute lower respiratory infection | Diagnosis | | J44.0 |  |
| Chronic obstructive pulmonary disease with acute exacerbation, unspecified | Diagnosis | | J44.1 |  |
| Other specified chronic obstructive pulmonary disease | Diagnosis | | J44.8 |  |
| Chronic obstructive pulmonary disease, unspecified | Diagnosis | | J44.9 |  |
| *Applied to the data from 2016/1/1 to 2019/12/31 (KCD-7 version)* |  | |  |  |
| Emphysema | Diagnosis | | J43 |  |
| Panlobular emphysema | Diagnosis | | J43.1 |  |
| Centrilobular emphysema | Diagnosis | | J43.2 |  |
| Other emphysema | Diagnosis | | J43.8 |  |
| Emphysema, unspecified | Diagnosis | | J43.9 |  |
| Other chronic obstructive pulmonary disease | Diagnosis | | J44 |  |
| Chronic obstructive pulmonary disease with acute lower respiratory infection | Diagnosis | | J44.0 |  |
| Chronic obstructive pulmonary disease with acute exacerbation, unspecified | Diagnosis | | J44.1 |  |
| Other specified chronic obstructive pulmonary disease | Diagnosis | | J44.8 |  |
| Chronic obstructive pulmonary disease, unspecified | Diagnosis | | J44.9 |  |
| Systemic steroid |  | |  |  |
| Refer to the attached file   | | | |  |
| Antibiotics |  | |  |  |
| Refer to the attached file   | | | |  |
| Severe COPD |  | |  | ≥ 1 ER or admission with defined diagnosis code as any diagnosis AND with defined drug codes for systemic steroid or antibiotics during 12 months after the index date |
| COPD |  | |  |  |
| *Applied to the data from 2005/1/1 to 2007/12/31 (KCD-4 version)* |  | |  |  |
| Emphysema | Diagnosis | | J43 |  |
| Panlobular emphysema | Diagnosis | | J43.1 |  |
| Centrilobular emphysema | Diagnosis | | J43.2 |  |
| Other emphysema | Diagnosis | | J43.8 |  |
| Emphysema, unspecified | Diagnosis | | J43.9 |  |
| Other chronic obstructive pulmonary disease | Diagnosis | | J44 |  |
| Chronic obstructive pulmonary disease with acute lower respiratory infection | Diagnosis | | J44.0 |  |
| Chronic obstructive pulmonary disease with acute exacerbation, unspecified | Diagnosis | | J44.1 |  |
| Other specified chronic obstructive pulmonary disease | Diagnosis | | J44.8 |  |
| Chronic obstructive pulmonary disease, unspecified | Diagnosis | | J44.9 |  |
| *Applied to the data from 2008/1/1 to 2010/12/31 (KCD-5 version)* |  | |  |  |
| Emphysema | Diagnosis | | J43 |  |
| Panlobular emphysema | Diagnosis | | J43.1 |  |
| Centrilobular emphysema | Diagnosis | | J43.2 |  |
| Other emphysema | Diagnosis | | J43.8 |  |
| Emphysema, unspecified | Diagnosis | | J43.9 |  |
| Other chronic obstructive pulmonary disease | Diagnosis | | J44 |  |
| Chronic obstructive pulmonary disease with acute lower respiratory infection | Diagnosis | | J44.0 |  |
| Chronic obstructive pulmonary disease with acute exacerbation, unspecified | Diagnosis | | J44.1 |  |
| Other specified chronic obstructive pulmonary disease | Diagnosis | | J44.8 |  |
| Chronic obstructive pulmonary disease, unspecified | Diagnosis | | J44.9 |  |
| *Applied to the data from 2011/1/1 to 2015/12/31 (KCD-6 version)* |  | |  |  |
| Emphysema | Diagnosis | | J43 |  |
| Panlobular emphysema | Diagnosis | | J43.1 |  |
| Centrilobular emphysema | Diagnosis | | J43.2 |  |
| Other emphysema | Diagnosis | | J43.8 |  |
| Emphysema, unspecified | Diagnosis | | J43.9 |  |
| Other chronic obstructive pulmonary disease | Diagnosis | | J44 |  |
| Chronic obstructive pulmonary disease with acute lower respiratory infection | Diagnosis | | J44.0 |  |
| Chronic obstructive pulmonary disease with acute exacerbation, unspecified | Diagnosis | | J44.1 |  |
| Other specified chronic obstructive pulmonary disease | Diagnosis | | J44.8 |  |
| Chronic obstructive pulmonary disease, unspecified | Diagnosis | | J44.9 |  |
| *Applied to the data from 2016/1/1 to 2019/12/31 (KCD-7 version)* |  | |  |  |
| Emphysema | Diagnosis | | J43 |  |
| Panlobular emphysema | Diagnosis | | J43.1 |  |
| Centrilobular emphysema | Diagnosis | | J43.2 |  |
| Other emphysema | Diagnosis | | J43.8 |  |
| Emphysema, unspecified | Diagnosis | | J43.9 |  |
| Other chronic obstructive pulmonary disease | Diagnosis | | J44 |  |
| Chronic obstructive pulmonary disease with acute lower respiratory infection | Diagnosis | | J44.0 |  |
| Chronic obstructive pulmonary disease with acute exacerbation, unspecified | Diagnosis | | J44.1 |  |
| Other specified chronic obstructive pulmonary disease | Diagnosis | | J44.8 |  |
| Chronic obstructive pulmonary disease, unspecified | Diagnosis | | J44.9 |  |
| Pneumonia |  | |  |  |
| *Applied to the data from 2005/1/1 to 2007/12/31 (KCD-4 version)* |  | |  |  |
| Viral pneumonia, not elsewhere classified | Diagnosis | | J12 |  |
| Adenoviral pneumonia | Diagnosis | | J12.0 |  |
| Respiratory syncytial virus pneumonia | Diagnosis | | J12.1 |  |
| Parainfluenza virus pneumonia | Diagnosis | | J12.2 |  |
| Other viral pneumonia | Diagnosis | | J12.8 |  |
| Viral pneumonia, unspecified | Diagnosis | | J12.9 |  |
| Pneumonia due to Streptococcus pneumoniae | Diagnosis | | J13 |  |
| Pneumonia due to Hemophilus influenzae | Diagnosis | | J14 |  |
| Bacterial pneumonia, not elsewhere classified | Diagnosis | | J15 |  |
| Pneumonia due to Klebsiella pneumoniae | Diagnosis | | J15.0 |  |
| Pneumonia due to Pseudomonas | Diagnosis | | J15.1 |  |
| Pneumonia due to staphylococcus | Diagnosis | | J15.2 |  |
| Pneumonia due to streptococcus, group B | Diagnosis | | J15.3 |  |
| Pneumonia due to other streptococci | Diagnosis | | J15.4 |  |
| Pneumonia due to Eschericha coli | Diagnosis | | J15.5 |  |
| Other aerobic pneumonia due to Gram-negative bacteria | Diagnosis | | J15.6 |  |
| Pneumonia due to Mycoplasma pneumoniae | Diagnosis | | J15.7 |  |
| Other bacterial pneumonia | Diagnosis | | J15.8 |  |
| Bacterial pneumonia | Diagnosis | | J15.9 |  |
| Pneumonia due to other infectious organisms, not elsewhere classified | Diagnosis | | J16 |  |
| Chlamydial pneumonia | Diagnosis | | J16.0 |  |
| Pneumonia due to other specified infectious organisms | Diagnosis | | J16.8 |  |
| Pneumonia in diseases classified elsewhere | Diagnosis | | J17 |  |
| Pneumonia in bacterial diseases classified elsewhere | Diagnosis | | J17.0 |  |
| Pneumonia in viral diseases classified elsewhere | Diagnosis | | J17.1 |  |
| Pneumonia in mycoses | Diagnosis | | J17.2 |  |
| Pneumonia in parasitic diseases | Diagnosis | | J17.3 |  |
| Pneumonia in other diseases classified elsewhere | Diagnosis | | J17.8 |  |
| Pneumonia, organism unspecified | Diagnosis | | J18 |  |
| Bronchopneumonia, unspecified | Diagnosis | | J18.0 |  |
| Lobar pneumonia, unspecified | Diagnosis | | J18.1 |  |
| Hypostatic pneumonia, unspecified | Diagnosis | | J18.2 |  |
| Other pneumonia, organism unspecified | Diagnosis | | J18.8 |  |
| Pneumonia | Diagnosis | | J18.9 |  |
| Pneumonitis due to food and vomit | Diagnosis | | J69.0 |  |
| Interstitial pulmonary disease, unspecified | Diagnosis | | J84.0 |  |
| Interstitial pulmonary disease, unspecified | Diagnosis | | J84.9 |  |
| Abscess of lung with pneumonia | Diagnosis | | J85.1 |  |
| Mycoplasma pneumoniae(M.pneumoniae) as the cause of diseases classified to other chapters | Diagnosis | | B96.0 |  |
| *Applied to the data from 2008/1/1 to 2010/12/31 (KCD-5 version)* |  | |  |  |
| Viral pneumonia, not elsewhere classified | Diagnosis | | J12 |  |
| Adenoviral pneumonia | Diagnosis | | J12.0 |  |
| Respiratory syncytial virus pneumonia | Diagnosis | | J12.1 |  |
| Parainfluenza virus pneumonia | Diagnosis | | J12.2 |  |
| Other viral pneumonia | Diagnosis | | J12.8 |  |
| Viral pneumonia, unspecified | Diagnosis | | J12.9 |  |
| Pneumonia due to Streptococcus pneumoniae | Diagnosis | | J13 |  |
| Pneumonia due to Hemophilus influenzae | Diagnosis | | J14 |  |
| Bacterial pneumonia, not elsewhere classified | Diagnosis | | J15 |  |
| Pneumonia due to Klebsiella pneumoniae | Diagnosis | | J15.0 |  |
| Pneumonia due to Pseudomonas | Diagnosis | | J15.1 |  |
| Pneumonia due to staphylococcus | Diagnosis | | J15.2 |  |
| Pneumonia due to streptococcus, group B | Diagnosis | | J15.3 |  |
| Pneumonia due to other streptococci | Diagnosis | | J15.4 |  |
| Pneumonia due to Eschericha coli | Diagnosis | | J15.5 |  |
| Other aerobic pneumonia due to Gram-negative bacteria | Diagnosis | | J15.6 |  |
| Pneumonia due to Mycoplasma pneumoniae | Diagnosis | | J15.7 |  |
| Other bacterial pneumonia | Diagnosis | | J15.8 |  |
| Bacterial pneumonia | Diagnosis | | J15.9 |  |
| Pneumonia due to other infectious organisms, not elsewhere classified | Diagnosis | | J16 |  |
| Chlamydial pneumonia | Diagnosis | | J16.0 |  |
| Pneumonia due to other specified infectious organisms | Diagnosis | | J16.8 |  |
| Pneumonia in diseases classified elsewhere | Diagnosis | | J17 |  |
| Pneumonia in bacterial diseases classified elsewhere | Diagnosis | | J17.0 |  |
| Pneumonia in viral diseases classified elsewhere | Diagnosis | | J17.1 |  |
| Pneumonia in mycoses | Diagnosis | | J17.2 |  |
| Pneumonia in parasitic diseases | Diagnosis | | J17.3 |  |
| Pneumonia in other diseases classified elsewhere | Diagnosis | | J17.8 |  |
| Pneumonia, organism unspecified | Diagnosis | | J18 |  |
| Bronchopneumonia, unspecified | Diagnosis | | J18.0 |  |
| Lobar pneumonia, unspecified | Diagnosis | | J18.1 |  |
| Hypostatic pneumonia, unspecified | Diagnosis | | J18.2 |  |
| Other pneumonia, organism unspecified | Diagnosis | | J18.8 |  |
| Pneumonia | Diagnosis | | J18.9 |  |
| Pneumonitis due to food and vomit | Diagnosis | | J69.0 |  |
| Interstitial pulmonary disease, unspecified | Diagnosis | | J84.0 |  |
| Interstitial pulmonary disease, unspecified | Diagnosis | | J84.9 |  |
| Abscess of lung with pneumonia | Diagnosis | | J85.1 |  |
| Mycoplasma pneumoniae(M.pneumoniae) as the cause of diseases classified to other chapters | Diagnosis | | B96.0 |  |
| *Applied to the data from 2011/1/1 to 2015/12/31 (KCD-6 version)* |  | |  |  |
| Viral pneumonia, not elsewhere classified | Diagnosis | | J12 |  |
| Adenoviral pneumonia | Diagnosis | | J12.0 |  |
| Respiratory syncytial virus pneumonia | Diagnosis | | J12.1 |  |
| Parainfluenza virus pneumonia | Diagnosis | | J12.2 |  |
| Human metapneumovirus pneumonia | Diagnosis | | J12.3 |  |
| Other viral pneumonia | Diagnosis | | J12.8 |  |
| Viral pneumonia, unspecified | Diagnosis | | J12.9 |  |
| Pneumonia due to streptococcus pneumonia | Diagnosis | | J13 |  |
| Pneumonia due to haemophilus influenzae | Diagnosis | | J14 |  |
| Bacterial pneumonia, not elsewhere classified | Diagnosis | | J15 |  |
| Pneumonia due to klebsiella pneumonia | Diagnosis | | J15.0 |  |
| Pneumonia due to pseudomonas | Diagnosis | | J15.1 |  |
| Pneumonia due to staphylococcus | Diagnosis | | J15.2 |  |
| Pneumonia due to streptococcus, group B | Diagnosis | | J15.3 |  |
| Pneumonia due to other streptococci | Diagnosis | | J15.4 |  |
| Pneumonia due to escherichia coli | Diagnosis | | J15.5 |  |
| Other aerobic pneumonia due to gram- negative bacteria | Diagnosis | | J15.6 |  |
| Pneumonia due to mycoplasma pneumonia | Diagnosis | | J15.7 |  |
| Other bacterial pneumonia | Diagnosis | | J15.8 |  |
| Bacterial pneumonia, unspecified | Diagnosis | | J15.9 |  |
| Pneumonia due to other infectious organisms, not elsewhere classified | Diagnosis | | J16 |  |
| Chlamydial pneumonia | Diagnosis | | J16.0 |  |
| Pneumonia due to other specified infectious organisms | Diagnosis | | J16.8 |  |
| Pneumonia in diseases classified elsewhere | Diagnosis | | J17 |  |
| Pneumonia in bacterial diseases classified elsewhere | Diagnosis | | J17.0 |  |
| Pneumonia in viral diseases classified elsewhere | Diagnosis | | J17.1 |  |
| Pneumonia in mycoses | Diagnosis | | J17.2 |  |
| Pneumonia in parasitic diseases | Diagnosis | | J17.3 |  |
| Pneumonia in other diseases classified elsewhere | Diagnosis | | J17.8 |  |
| Pneumonia, organism unspecified | Diagnosis | | J18 |  |
| Bronchopneumonia, unspecified | Diagnosis | | J18.0 |  |
| Lobar pneumonia, unspecified | Diagnosis | | J18.1 |  |
| Hypostatic pneumonia, unspecified | Diagnosis | | J18.2 |  |
| Other pneumonia, organism unspecified | Diagnosis | | J18.8 |  |
| Pneumonia, unspecified | Diagnosis | | J18.9 |  |
| Pneumonitis due to food and vomit | Diagnosis | | J69.0 |  |
| Interstitial pulmonary disease, unspecified | Diagnosis | | J84.0 |  |
| Interstitial pulmonary disease, unspecified | Diagnosis | | J84.9 |  |
| Abscess of lung with pneumonia | Diagnosis | | J85.1 |  |
| Mycoplasma pneumoniae(M.pneumoniae) as the cause of diseases classified to other chapters | Diagnosis | | B96.0 |  |
| *Applied to the data from 2016/1/1 to 2019/12/31 (KCD-7 version)* |  | |  |  |
| Viral pneumonia, not elsewhere classified | Diagnosis | | J12 |  |
| Respiratory syncytial virus pneumonia | Diagnosis | | J12.1 |  |
| Parainfluenza virus pneumonia | Diagnosis | | J12.2 |  |
| Human metapneumovirus pneumonia | Diagnosis | | J12.3 |  |
| Other viral pneumonia | Diagnosis | | J12.8 |  |
| Other viral pneumonia | Diagnosis | | J12.88 |  |
| Viral pneumonia, unspecified | Diagnosis | | J12.9 |  |
| Pneumonia due to streptococcus pneumoniae | Diagnosis | | J13 |  |
| Pneumonia due to haemophilus influenzae | Diagnosis | | J14 |  |
| Bacterial pneumonia, not elsewhere classified | Diagnosis | | J15 |  |
| Pneumonia due to klebsiella pneumoniae | Diagnosis | | J15.0 |  |
| Pneumonia due to pseudomonas | Diagnosis | | J15.1 |  |
| Pneumonia due to staphylococcus | Diagnosis | | J15.2 |  |
| Pneumonia due to streptococcus, group B | Diagnosis | | J15.3 |  |
| Pneumonia due to other streptococci | Diagnosis | | J15.4 |  |
| Pneumonia due to escherichia coli | Diagnosis | | J15.5 |  |
| Other aerobic pneumonia due to gram- negative bacteria | Diagnosis | | J15.6 |  |
| Pneumonia due to mycoplasma pneumoniae | Diagnosis | | J15.7 |  |
| Other bacterial pneumonia | Diagnosis | | J15.8 |  |
| Bacterial pneumonia, unspecified | Diagnosis | | J15.9 |  |
| Pneumonia due to other infectious organisms, not elsewhere classified | Diagnosis | | J16 |  |
| Chlamydial pneumonia | Diagnosis | | J16.0 |  |
| Pneumonia due to other specified infectious organisms | Diagnosis | | J16.8 |  |
| Pneumonia in diseases classified elsewhere | Diagnosis | | J17 |  |
| Pneumonia in bacterial diseases classified elsewhere | Diagnosis | | J17.0 |  |
| Pneumonia in viral diseases classified elsewhere | Diagnosis | | J17.1 |  |
| Pneumonia in mycoses | Diagnosis | | J17.2 |  |
| Pneumonia in parasitic diseases | Diagnosis | | J17.3 |  |
| Pneumonia in other diseases classified elsewhere | Diagnosis | | J17.8 |  |
| Pneumonia, organism unspecified | Diagnosis | | J18 |  |
| Bronchopneumonia, unspecified | Diagnosis | | J18.0 |  |
| Lobar pneumonia, unspecified | Diagnosis | | J18.1 |  |
| Hypostatic pneumonia, unspecified | Diagnosis | | J18.2 |  |
| Other pneumonia, organism unspecified | Diagnosis | | J18.8 |  |
| Interstitial pulmonary disease, unspecified | Diagnosis | | J84.0 |  |
| Interstitial pulmonary disease, unspecified | Diagnosis | | J84.9 |  |
| Abscess of lung with pneumonia | Diagnosis | | J85.1 |  |
| Mycoplasma pneumoniae(M.pneumoniae) as the cause of diseases classified to other chapters | Diagnosis | | B96.0 |  |
| Pulmonary thromboembolism |  | |  |  |
| *Applied to the data from 2005/1/1 to 2007/12/31 (KCD-4 version)* |  | |  |  |
| Pulmonary embolism with mention of acute cor pulmonale | Diagnosis | | I26.0 |  |
| Pulmonary embolism without mention of acute cor pulmonale | Diagnosis | | I26.9 |  |
| *Applied to the data from 2008/1/1 to 2010/12/31 (KCD-5 version)* |  | |  |  |
| Pulmonary embolism with mention of acute cor pulmonale | Diagnosis | | I26.0 |  |
| Pulmonary embolism without mention of acute cor pulmonale | Diagnosis | | I26.9 |  |
| *Applied to the data from 2011/1/1 to 2015/12/31 (KCD-6 version)* |  | |  |  |
| Pulmonary embolism | Diagnosis | | I26 |  |
| Pulmonary embolism with mention of acute cor pulmonale | Diagnosis | | I26.0 |  |
| Pulmonary embolism without mention of acute cor pulmonale | Diagnosis | | I26.9 |  |
| *Applied to the data from 2016/1/1 to 2019/12/31 (KCD-7 version)* |  | |  |  |
| Pulmonary embolism | Diagnosis | | I26 |  |
| Pulmonary embolism with mention of acute cor pulmonale | Diagnosis | | I26.0 |  |
| Pulmonary embolism without mention of acute cor pulmonale | Diagnosis | | I26.9 |  |
| Dyspnea |  | |  |  |
| *Applied to the data from 2005/1/1 to 2007/12/31 (KCD-4 version)* |  | |  |  |
| Dyspnea | Diagnosis | | R06.0 |  |
| *Applied to the data from 2008/1/1 to 2010/12/31 (KCD-5 version)* |  | |  |  |
| Dyspnea | Diagnosis | | R06.0 |  |
| *Applied to the data from 2011/1/1 to 2015/12/31 (KCD-6 version)* |  | |  |  |
| Dyspnea | Diagnosis | | R06.0 |  |
| *Applied to the data from 2016/1/1 to 2019/12/31 (KCD-7 version)* |  | |  |  |
| Acute respiratory disease |  | |  |  |
| *Applied to the data from 2005/1/1 to 2007/12/31 (KCD-4 version)* |  | |  |  |
| Adult respiratory distress syndrome | Diagnosis | | J80 |  |
| *Applied to the data from 2008/1/1 to 2010/12/31 (KCD-5 version)* |  | |  |  |
| Adult respiratory distress syndrome | Diagnosis | | J80 |  |
| *Applied to the data from 2011/1/1 to 2015/12/31 (KCD-6 version)* |  | |  |  |
| Adult respiratory distress syndrome | Diagnosis | | J80 |  |
| *Applied to the data from 2016/1/1 to 2019/12/31 (KCD-7 version)* |  | |  |  |
| Adult respiratory distress syndrome | Diagnosis | | J80 |  |
| Systemic steroid |  | |  |  |
| Refer to the attached file   | | | |  |
| Antibiotics |  | |  |  |
| Refer to the attached file   | | | |  |
| ER visits |  |  | |  |
| ER visit | Procedure | AC101.x | |  |
| ER visit | Procedure | AC103.x | |  |
| ER visit | Procedure | AC105.x | |  |
| ER visit | Procedure | V1.x | |  |
| ER visit | Procedure | V2.x | |  |
| ER visit | Procedure | V3.x | |  |
| ER visit | Procedure | V4.x | |  |
| ER visit | Procedure | V5.x | |  |
| ER visit | Specialty code | 24 | |  |
| Moderate to Severe COPD exacerbation | Moderate COPD exacerbation + Severe COPD exacerbation | | | Each definition is described as above |

**Table S7‑5 Operational definition for COPD exacerbation**

| **Criteria** | **Operational definition** |
| --- | --- |
| COPD exacerbation |  |
| Moderate COPD exacerbation | - ≥ 1 Outpatient claim(s) with   1) ICD-10 codes for COPD recorded as primary ~ 5th diagnosis AND  2) Systemic steroids and/or antibiotics prescriptions observed |
| Severe COPD exacerbation | - ≥ 1 Inpatient claim(s) or ER visit(s) with   1) ICD-10 codes for COPD or diseases due to COPD worsening as any diagnosis AND  2) Systemic steroids and/or antibiotics prescriptions observed |
| Moderate to severe COPD exacerbation | - Composition event of moderate and severe COPD exacerbation |
| Antibiotics-used COPD exacerbation |  |
| Antibiotics-used  moderate COPD exacerbation | - ≥ 1 Outpatient claim(s) with   1) ICD-10 codes for COPD recorded as primary ~ 5th diagnosis AND  2) Antibiotics prescriptions observed |
| Antibiotics-used  severe COPD exacerbation | - ≥ 1 Inpatient claim(s) or ER visit(s) with   1) ICD-10 codes for COPD or diseases due to COPD worsening as any diagnosis AND  2) Antibiotics prescriptions observed |
| Antibiotics-used  moderate to severe COPD exacerbation | - Composition event of antibotics-used moderate and antibotics-used severe COPD exacerbation |
| Oral corticosteroid (OCS)-used  COPD exacerbation |  |
| OCS-used  moderate COPD exacerbation | - ≥ 1 Outpatient claim(s) with   1) ICD-10 codes for COPD recorded as primary ~ 5th diagnosis AND  2) OCS prescriptions observed |
| OCS-used  severe COPD exacerbation | - ≥ 1 Inpatient claim(s) or ER visit(s) with   1) ICD-10 codes for COPD or diseases due to COPD worsening as any diagnosis AND  2) OCS prescriptions observed |
| OCS-used  moderate to severe COPD exacerbation | - Composition event of OCS-used moderate and OCS-used severe COPD exacerbation |
| Antibiotics and OCS-used  COPD exacerbation |  |
| Antibiotics and OCS-used  moderate COPD exacerbation | - ≥ 1 Outpatient claim(s) with   1) ICD-10 codes for COPD recorded as primary ~ 5th diagnosis AND  2) OCS and antibiotics prescriptions observed |
| Antibiotics and OCS-used  severe COPD exacerbation | - ≥ 1 Inpatient claim(s) or ER visit(s) with   1) ICD-10 codes for COPD or diseases due to COPD worsening as any diagnosis AND  2) OCS and antibiotics prescriptions observed |
| Antibiotics and OCS-used  moderate to severe COPD exacerbation | Composition event of Antibiotics and OCS-used moderate and Antibiotics and OCS-used severe COPD exacerbation |
| Diagnosis codes and HIRA molecule codes for Moderate COPD exacerbation | - See Appendix 7.3, Table 7-4 |
| Diagnosis codes and HIRA molecule codes for Severe COPD exacerbation | - See Appendix 7.3, Table 7-4 |

## 7.4. Covariate(s)

Table S7‑6 Relevant diagnosis codes, drug codes, and detailed operational definition for history of pneumonia

| **Operational definition** | **Class** | **Code** | **Detailed operational definition** |
| --- | --- | --- | --- |
| Pneumonia |  |  | ≥ 1 admission or out-patient visit with defined diagnosis code as any diagnosis in in-patients claims or primary-5^th^ diagnosis in out-patient claims, AND with defined diagnostic test codes, AND with antibiotics during 12 months before the index date |
| *Applied to the data from 2004/1/1 to 2007/12/31 (KCD-4 version)* |  |  |  |
| Viral pneumonia, not elsewhere classified | Diagnosis | J12 |  |
| Adenoviral pneumonia | Diagnosis | J12.0 |  |
| Respiratory syncytial virus pneumonia | Diagnosis | J12.1 |  |
| Parainfluenza virus pneumonia | Diagnosis | J12.2 |  |
| Other viral pneumonia | Diagnosis | J12.8 |  |
| Viral pneumonia, unspecified | Diagnosis | J12.9 |  |
| Pneumonia due to Streptococcus pneumoniae | Diagnosis | J13 |  |
| Pneumonia due to Hemophilus influenzae | Diagnosis | J14 |  |
| Bacterial pneumonia, not elsewhere classified | Diagnosis | J15 |  |
| Pneumonia due to Klebsiella pneumoniae | Diagnosis | J15.0 |  |
| Pneumonia due to Pseudomonas | Diagnosis | J15.1 |  |
| Pneumonia due to staphylococcus | Diagnosis | J15.2 |  |
| Pneumonia due to streptococcus, group B | Diagnosis | J15.3 |  |
| Pneumonia due to other streptococci | Diagnosis | J15.4 |  |
| Pneumonia due to Eschericha coli | Diagnosis | J15.5 |  |
| Other aerobic pneumonia due to Gram-negative bacteria | Diagnosis | J15.6 |  |
| Pneumonia due to Mycoplasma pneumoniae | Diagnosis | J15.7 |  |
| Other bacterial pneumonia | Diagnosis | J15.8 |  |
| Bacterial pneumonia | Diagnosis | J15.9 |  |
| Pneumonia due to other infectious organisms, not elsewhere classified | Diagnosis | J16 |  |
| Chlamydial pneumonia | Diagnosis | J16.0 |  |
| Pneumonia due to other specified infectious organisms | Diagnosis | J16.8 |  |
| Pneumonia in diseases classified elsewhere | Diagnosis | J17 |  |
| Pneumonia in bacterial diseases classified elsewhere | Diagnosis | J17.0 |  |
| Pneumonia in viral diseases classified elsewhere | Diagnosis | J17.1 |  |
| Pneumonia in mycoses | Diagnosis | J17.2 |  |
| Pneumonia in parasitic diseases | Diagnosis | J17.3 |  |
| Pneumonia in other diseases classified elsewhere | Diagnosis | J17.8 |  |
| Pneumonia, organism unspecified | Diagnosis | J18 |  |
| Bronchopneumonia, unspecified | Diagnosis | J18.0 |  |
| Lobar pneumonia, unspecified | Diagnosis | J18.1 |  |
| Hypostatic pneumonia, unspecified | Diagnosis | J18.2 |  |
| Other pneumonia, organism unspecified | Diagnosis | J18.8 |  |
| Pneumonia | Diagnosis | J18.9 |  |
| Pneumonitis due to food and vomit | Diagnosis | J69.0 |  |
| Interstitial pulmonary disease, unspecified | Diagnosis | J84.0 |  |
| Interstitial pulmonary disease, unspecified | Diagnosis | J84.9 |  |
| Abscess of lung with pneumonia | Diagnosis | J85.1 |  |
| Mycoplasma pneumoniae(M.pneumoniae) as the cause of diseases classified to other chapters | Diagnosis | B96.0 |  |
| *Applied to the data from 2008/1/1 to 2010/12/31 (KCD-5 version)* |  |  |  |
| Viral pneumonia, not elsewhere classified | Diagnosis | J12 |  |
| Adenoviral pneumonia | Diagnosis | J12.0 |  |
| Respiratory syncytial virus pneumonia | Diagnosis | J12.1 |  |
| Parainfluenza virus pneumonia | Diagnosis | J12.2 |  |
| Other viral pneumonia | Diagnosis | J12.8 |  |
| Viral pneumonia, unspecified | Diagnosis | J12.9 |  |
| Pneumonia due to Streptococcus pneumoniae | Diagnosis | J13 |  |
| Pneumonia due to Hemophilus influenzae | Diagnosis | J14 |  |
| Bacterial pneumonia, not elsewhere classified | Diagnosis | J15 |  |
| Pneumonia due to Klebsiella pneumoniae | Diagnosis | J15.0 |  |
| Pneumonia due to Pseudomonas | Diagnosis | J15.1 |  |
| Pneumonia due to staphylococcus | Diagnosis | J15.2 |  |
| Pneumonia due to streptococcus, group B | Diagnosis | J15.3 |  |
| Pneumonia due to other streptococci | Diagnosis | J15.4 |  |
| Pneumonia due to Eschericha coli | Diagnosis | J15.5 |  |
| Other aerobic pneumonia due to Gram-negative bacteria | Diagnosis | J15.6 |  |
| Pneumonia due to Mycoplasma pneumoniae | Diagnosis | J15.7 |  |
| Other bacterial pneumonia | Diagnosis | J15.8 |  |
| Bacterial pneumonia | Diagnosis | J15.9 |  |
| Pneumonia due to other infectious organisms, not elsewhere classified | Diagnosis | J16 |  |
| Chlamydial pneumonia | Diagnosis | J16.0 |  |
| Pneumonia due to other specified infectious organisms | Diagnosis | J16.8 |  |
| Pneumonia in diseases classified elsewhere | Diagnosis | J17 |  |
| Pneumonia in bacterial diseases classified elsewhere | Diagnosis | J17.0 |  |
| Pneumonia in viral diseases classified elsewhere | Diagnosis | J17.1 |  |
| Pneumonia in mycoses | Diagnosis | J17.2 |  |
| Pneumonia in parasitic diseases | Diagnosis | J17.3 |  |
| Pneumonia in other diseases classified elsewhere | Diagnosis | J17.8 |  |
| Pneumonia, organism unspecified | Diagnosis | J18 |  |
| Bronchopneumonia, unspecified | Diagnosis | J18.0 |  |
| Lobar pneumonia, unspecified | Diagnosis | J18.1 |  |
| Hypostatic pneumonia, unspecified | Diagnosis | J18.2 |  |
| Other pneumonia, organism unspecified | Diagnosis | J18.8 |  |
| Pneumonia | Diagnosis | J18.9 |  |
| Pneumonitis due to food and vomit | Diagnosis | J69.0 |  |
| Interstitial pulmonary disease, unspecified | Diagnosis | J84.0 |  |
| Interstitial pulmonary disease, unspecified | Diagnosis | J84.9 |  |
| Abscess of lung with pneumonia | Diagnosis | J85.1 |  |
| Mycoplasma pneumoniae(M.pneumoniae) as the cause of diseases classified to other chapters | Diagnosis | B96.0 |  |
| *Applied to the data from 2011/1/1 to 2015/12/31 (KCD-6 version)* |  |  |  |
| Viral pneumonia, not elsewhere classified | Diagnosis | J12 |  |
| Adenoviral pneumonia | Diagnosis | J12.0 |  |
| Respiratory syncytial virus pneumonia | Diagnosis | J12.1 |  |
| Parainfluenza virus pneumonia | Diagnosis | J12.2 |  |
| Human metapneumovirus pneumonia | Diagnosis | J12.3 |  |
| Other viral pneumonia | Diagnosis | J12.8 |  |
| Viral pneumonia, unspecified | Diagnosis | J12.9 |  |
| Pneumonia due to streptococcus pneumonia | Diagnosis | J13 |  |
| Pneumonia due to haemophilus influenzae | Diagnosis | J14 |  |
| Bacterial pneumonia, not elsewhere classified | Diagnosis | J15 |  |
| Pneumonia due to klebsiella pneumonia | Diagnosis | J15.0 |  |
| Pneumonia due to pseudomonas | Diagnosis | J15.1 |  |
| Pneumonia due to staphylococcus | Diagnosis | J15.2 |  |
| Pneumonia due to streptococcus, group B | Diagnosis | J15.3 |  |
| Pneumonia due to other streptococci | Diagnosis | J15.4 |  |
| Pneumonia due to escherichia coli | Diagnosis | J15.5 |  |
| Other aerobic pneumonia due to gram- negative bacteria | Diagnosis | J15.6 |  |
| Pneumonia due to mycoplasma pneumonia | Diagnosis | J15.7 |  |
| Other bacterial pneumonia | Diagnosis | J15.8 |  |
| Bacterial pneumonia, unspecified | Diagnosis | J15.9 |  |
| Pneumonia due to other infectious organisms, not elsewhere classified | Diagnosis | J16 |  |
| Chlamydial pneumonia | Diagnosis | J16.0 |  |
| Pneumonia due to other specified infectious organisms | Diagnosis | J16.8 |  |
| Pneumonia in diseases classified elsewhere | Diagnosis | J17 |  |
| Pneumonia in bacterial diseases classified elsewhere | Diagnosis | J17.0 |  |
| Pneumonia in viral diseases classified elsewhere | Diagnosis | J17.1 |  |
| Pneumonia in mycoses | Diagnosis | J17.2 |  |
| Pneumonia in parasitic diseases | Diagnosis | J17.3 |  |
| Pneumonia in other diseases classified elsewhere | Diagnosis | J17.8 |  |
| Pneumonia, organism unspecified | Diagnosis | J18 |  |
| Bronchopneumonia, unspecified | Diagnosis | J18.0 |  |
| Lobar pneumonia, unspecified | Diagnosis | J18.1 |  |
| Hypostatic pneumonia, unspecified | Diagnosis | J18.2 |  |
| Other pneumonia, organism unspecified | Diagnosis | J18.8 |  |
| Pneumonia, unspecified | Diagnosis | J18.9 |  |
| Pneumonitis due to food and vomit | Diagnosis | J69.0 |  |
| Interstitial pulmonary disease, unspecified | Diagnosis | J84.0 |  |
| Interstitial pulmonary disease, unspecified | Diagnosis | J84.9 |  |
| Abscess of lung with pneumonia | Diagnosis | J85.1 |  |
| Mycoplasma pneumoniae(M.pneumoniae) as the cause of diseases classified to other chapters | Diagnosis | B96.0 |  |
| *Applied to the data from 2016/1/1 to 2019/12/31 (KCD-7 version)* |  |  |  |
| Viral pneumonia, not elsewhere classified | Diagnosis | J12 |  |
| Respiratory syncytial virus pneumonia | Diagnosis | J12.1 |  |
| Parainfluenza virus pneumonia | Diagnosis | J12.2 |  |
| Human metapneumovirus pneumonia | Diagnosis | J12.3 |  |
| Other viral pneumonia | Diagnosis | J12.8 |  |
| Other viral pneumonia | Diagnosis | J12.88 |  |
| Viral pneumonia, unspecified | Diagnosis | J12.9 |  |
| Pneumonia due to streptococcus pneumoniae | Diagnosis | J13 |  |
| Pneumonia due to haemophilus influenzae | Diagnosis | J14 |  |
| Bacterial pneumonia, not elsewhere classified | Diagnosis | J15 |  |
| Pneumonia due to klebsiella pneumoniae | Diagnosis | J15.0 |  |
| Pneumonia due to pseudomonas | Diagnosis | J15.1 |  |
| Pneumonia due to staphylococcus | Diagnosis | J15.2 |  |
| Pneumonia due to streptococcus, group B | Diagnosis | J15.3 |  |
| Pneumonia due to other streptococci | Diagnosis | J15.4 |  |
| Pneumonia due to escherichia coli | Diagnosis | J15.5 |  |
| Other aerobic pneumonia due to gram- negative bacteria | Diagnosis | J15.6 |  |
| Pneumonia due to mycoplasma pneumoniae | Diagnosis | J15.7 |  |
| Other bacterial pneumonia | Diagnosis | J15.8 |  |
| Bacterial pneumonia, unspecified | Diagnosis | J15.9 |  |
| Pneumonia due to other infectious organisms, not elsewhere classified | Diagnosis | J16 |  |
| Chlamydial pneumonia | Diagnosis | J16.0 |  |
| Pneumonia due to other specified infectious organisms | Diagnosis | J16.8 |  |
| Pneumonia in diseases classified elsewhere | Diagnosis | J17 |  |
| Pneumonia in bacterial diseases classified elsewhere | Diagnosis | J17.0 |  |
| Pneumonia in viral diseases classified elsewhere | Diagnosis | J17.1 |  |
| Pneumonia in mycoses | Diagnosis | J17.2 |  |
| Pneumonia in parasitic diseases | Diagnosis | J17.3 |  |
| Pneumonia in other diseases classified elsewhere | Diagnosis | J17.8 |  |
| Pneumonia, organism unspecified | Diagnosis | J18 |  |
| Bronchopneumonia, unspecified | Diagnosis | J18.0 |  |
| Lobar pneumonia, unspecified | Diagnosis | J18.1 |  |
| Hypostatic pneumonia, unspecified | Diagnosis | J18.2 |  |
| Other pneumonia, organism unspecified | Diagnosis | J18.8 |  |
| Interstitial pulmonary disease, unspecified | Diagnosis | J84.0 |  |
| Interstitial pulmonary disease, unspecified | Diagnosis | J84.9 |  |
| Abscess of lung with pneumonia | Diagnosis | J85.1 |  |
| Mycoplasma pneumoniae(M.pneumoniae) as the cause of diseases classified to other chapters | Diagnosis | B96.0 |  |
| Chest-x ray |  |  |  |
| Chest-x ray | Procedure | G2101.x |  |
| Chest-x ray | Procedure | G2102.x |  |
| Chest-x ray | Procedure | G2103.x |  |
| Chest-x ray | Procedure | G2104.x |  |
| Chest-x ray | Procedure | G2105.x |  |
| Chest CT |  |  |  |
| Chest CT | Procedure | HA424.x |  |
| Chest CT | Procedure | HA434.x |  |
| Chest CT | Procedure | HA444.x |  |
| Chest CT | Procedure | HA454.x |  |
| Chest CT | Procedure | HA464.x |  |
| Chest CT | Procedure | HA474.x |  |
| Chest CT | Procedure | HA484.x |  |
| Chest CT | Procedure | HA834.x |  |
| Antibiotics |  |  |  |
| Refer to the attached file   | | |  |

**Table S7-7 Relevant diagnosis codes, drug codes, and detailed operational definition for history of asthma**

| **Operational definition** | **Class** | **Code** | **Detailed operational definition** |
| --- | --- | --- | --- |
| **Asthma** |  |  | ≥ 1 admission or out-patient visit with defined diagnosis code as any diagnosis in in-patient claims or primary-5^th^ diagnosis in out-patient claims during 12 months before the index date |
| *Applied to the data from 2005/1/1 to 2007/12/31 (KCD-4 version)* |  |  |  |
| Asthma | Diagnosis | J45 |  |
| Predominantly allergic asthma | Diagnosis | J45.0 |  |
| Nonallergic asthma | Diagnosis | J45.1 |  |
| Mixed asthma | Diagnosis | J45.8 |  |
| Asthma, unspecified | Diagnosis | J45.9 |  |
| Status asthmaticus | Diagnosis | J46 |  |
| *Applied to the data from 2008/1/1 to 2010/12/31 (KCD-5 version)* |  |  |  |
| Asthma | Diagnosis | J45 |  |
| Predominantly allergic asthma | Diagnosis | J45.0 |  |
| Nonallergic asthma | Diagnosis | J45.1 |  |
| Mixed asthma | Diagnosis | J45.8 |  |
| Asthma, unspecified | Diagnosis | J45.9 |  |
| Status asthmaticus | Diagnosis | J46 |  |
| *Applied to the data from 2011/1/1 to 2015/12/31 (KCD-6 version)* |  |  |  |
| Asthma | Diagnosis | J45 |  |
| Predominantly allergic asthma | Diagnosis | J45.0 |  |
| Nonallergic asthma | Diagnosis | J45.1 |  |
| Mixed asthma | Diagnosis | J45.8 |  |
| Asthma, unspecified | Diagnosis | J45.9 |  |
| Status asthmaticus | Diagnosis | J46 |  |
| With predominantly allergic asthma | Diagnosis | J46.0 |  |
| With nonallergic asthma | Diagnosis | J46.1 |  |
| With mixed asthma | Diagnosis | J46.8 |  |
| With unspecified asthma | Diagnosis | J46.9 |  |
| *Applied to the data from 2016/1/1 to 2019/12/31 (KCD-7 version)* |  |  |  |
| Asthma | Diagnosis | J45 |  |
| Predominantly allergic asthma | Diagnosis | J45.0 |  |
| Nonallergic asthma | Diagnosis | J45.1 |  |
| Mixed asthma | Diagnosis | J45.8 |  |
| Asthma, unspecified | Diagnosis | J45.9 |  |
| Status asthmaticus | Diagnosis | J46 |  |

**Table S7-8 Relevant diagnosis codes, drug codes, and detailed operational definition for Charlson Comorbidity Index (CCI)**

| **Operational definition** | **Class** | **Code** | **Detailed operational definition** |
| --- | --- | --- | --- |
| **Congestive heart failure (2 point)** |  |  | ≥1 admission or outpatient visit with defined diagnosis code as any diagnosis for in-patient claims or primary-5^th^ diagnosis in out-patient claims during 12 months before the index date |
| Refer to the attached file   | | |  |
| **Dementia (2 point)** |  |  | ≥1 admission or outpatient visit with defined diagnosis code as any diagnosis for in-patient claims or primary-5th diagnosis in out-patient claims during 12 months before the index date |
| Refer to the attached file   | | |  |
| **Chronic pulmonary disease (1 point)** |  |  | ≥1 admission or outpatient visit with defined diagnosis code as any diagnosis for in-patient claims or primary-5th diagnosis in out-patient claims during 12 months before the index date |
| Refer to the attached file   | | |  |
| **Rheumatologic disease (1 point)** |  |  | ≥1 admission or outpatient visit with defined diagnosis code as any diagnosis for in-patient claims or primary-5th diagnosis in out-patient claims during 12 months before the index date |
| Refer to the attached file   | | |  |
| **Mild liver disease (2 point)** |  |  | ≥1 admission or outpatient visit with defined diagnosis code as any diagnosis for in-patient claims or primary-5th diagnosis in out-patient claims during 12 months before the index date |
| Refer to the attached file   | | |  |
| **Diabetes with chronic complications (1 point)** |  |  | ≥1 admission or outpatient visit with defined diagnosis code as any diagnosis for in-patient claims or primary-5th diagnosis in out-patient claims during 12 months before the index date |
| Refer to the attached file   | | |  |
| **Hemiplegia or paraplegia (2 point)** |  |  | ≥1 admission or outpatient visit with defined diagnosis code as any diagnosis for in-patient claims or primary-5th diagnosis in out-patient claims during 12 months before the index date |
| Refer to the attached file   | | |  |
| **Renal disease (1 point)** |  |  | ≥1 admission or outpatient visit with defined diagnosis code as any diagnosis for in-patient claims or primary-5th diagnosis in out-patient claims during 12 months before the index date |
| Refer to the attached file   | | |  |
| **Any malignancy, including lymphoma and leukemia (2 point)** |  |  | ≥1 admission or outpatient visit with defined diagnosis code as any diagnosis for in-patient claims or primary-5th diagnosis in out-patient claims during 12 months before the index date |
| Refer to the attached file   | | |  |
| **Moderate or severe liver disease (4 point)** |  |  | ≥1 admission or outpatient visit with defined diagnosis code as any diagnosis for in-patient claims or primary-5th diagnosis in out-patient claims during 12 months before the index date |
| Refer to the attached file   | | |  |
| **Metastatic solid tumor (6 point)** |  |  | ≥1 admission or outpatient visit with defined diagnosis code as any diagnosis for in-patient claims or primary-5th diagnosis in out-patient claims during 12 months before the index date |
| Refer to the attached file   | | |  |
| HIV (4 point) |  |  | ≥1 admission or outpatient visit with defined diagnosis code as any diagnosis for in-patient claims or primary-5th diagnosis in out-patient claims during 12 months before the index date |
| Refer to the attached file   | | |  |
